# Decision Limits for Fetal Speckle-Tracking Outputs: Minimal Detectable Change and Threshold Instability in FetalHQ

**DOI:** 10.3390/biomedicines14102392

**Published:** 2026-10-19

**Authors:** Jakub Mlodawski, Anna Zmelonek-Znamirowska, Lukasz Pawlik, Marta Mlodawska, Grzegorz Swiercz

**Affiliations:** 1 Institute of Medical Sciences, Collegium Medicum, Jan Kochanowski University in Kielce, 25-516 Kielce, Poland; rjawka@poczta.fm (A.Z.-Z.); marta.mlodawska@ujk.edu.pl (M.M.); grzegorz.swiercz@ujk.edu.pl (G.S.); 2 Clinic of Obstetrics and Gynecology, Provincial Combined Hospital in Kielce, 25-736 Kielce, Poland; 3 Department of Information Systems, Kielce University of Technology, 25-314 Kielce, Poland; lpawlik@tu.kielce.pl

**Keywords:** fetal speckle-tracking echocardiography, FetalHQ, fetal echocardiography, reproducibility of results, observer variation, minimal detectable change, threshold instability

## Abstract

**Background**: Fetal Heart Quantification (FetalHQ) enables detailed assessment of fetal cardiac morphology and function using two-dimensional speckle-tracking echocardiography. However, previous studies have focused primarily on conventional reproducibility metrics such as the intraclass correlation coefficient (ICC). From a clinical perspective, it is more relevant to determine what magnitude of change exceeds measurement noise and whether a single result may lead to unstable classification relative to normative thresholds. The aim of this study was to evaluate the decision limits of selected FetalHQ parameters, including the minimal detectable change (MDC95), threshold instability, and the theoretical effect of averaging two independent analyses. **Materials and Methods**: This methodological study included 93 paired analyses (_1 and _2) obtained from the same recorded four-chamber cine loop under the assumption of zero biological variability. The intra-operator subset (*n* = 34) and inter-operator subset (*n* = 59) were analyzed separately. For key parameters, we determined the proportion of usable pairs after quality filtering, bias, Bland–Altman 95% limits of agreement, MDC95, classification flip rates for normalized outputs, and the width of the uncertainty zone (grey zone) around diagnostic thresholds. In addition, we estimated the effect of a mean-of-two workflow on measurement error reduction. **Results**: The best analytical performance was observed for left ventricular volumetric parameters, particularly LV EDV and LV ESV, which showed the highest proportion of usable pairs (88.2–94.9%) and relatively low MDC95 values. Functional and deformation metrics performed substantially worse, including LV EF, LV FRAC, LV GLS, and RV TAPSE, all of which were characterized by lower usability and wider limits of agreement, especially in the inter-operator analysis. Classification instability was greatest for normalized parameters. In the inter-operator analysis, classification flips occurred in 33.3% of cases for LV EF centile <5, 31.4% for RV TAPSE lateral centile <5, 35.7% for LV GLS centile >95, and 39.2% for LV GLS Z-score >2. Under the theoretical assumption of independent random measurement error, the estimated MDC95 for the mean of two analyses was approximately 29% lower than that for a single analysis; this reduction was mathematically derived rather than prospectively observed. **Conclusions**: FetalHQ showed the highest analytical stability for simpler LV volumetric parameters, whereas functional and deformation outputs remained substantially more susceptible to measurement noise and classification instability, particularly between operators. In practice, this means that a single measurement obtained near normative thresholds may be insufficient for reliable interpretation. A theoretical mean-of-two approach may reduce random measurement uncertainty, but this strategy was not prospectively validated and does not overcome the limitations of the least analytically stable parameters.

## 1. Introduction

FetalHQ software is an innovative fetal imaging technology based on two-dimensional speckle-tracking echocardiography (2D-STE). It enables functional assessment of the fetal heart on the basis of an entirely different imaging principle: the analysis of speckles, that is, fine patterns within the ultrasound image. What had long been regarded primarily as noise resulting from interference among waves reflected from tissue microstructures is now analyzed as a speckle pattern [1]. This pattern can currently be tracked frame by frame and used for functional cardiac assessment based on endocardial speckle-pattern tracking. The analysis includes motion vectors for thousands of pixels containing the speckle pattern and allows calculation of the fundamental parameter of this imaging approach, namely strain, which reflects the percentage of tissue shortening or lengthening, expressed as the relative change in vector length from its baseline value [2].

Speckle-pattern analysis allows the assessment of parameters such as GLS (global longitudinal strain), segmental strain, shortening fraction, and ejection fraction (EF, indirectly). In addition, it enables evaluation of TAPSE (tricuspid annular plane systolic excursion), MAPSE (mitral annular plane systolic excursion), ventricular shortening fraction (SF), and ventricular sphericity index (SI). FetalHQ itself allows each cardiac ventricle to be divided into 24 segments, and all parameters can be calculated separately for each segment and displayed against centile grids. As a result, the software generates an extremely large volume of data, and the examination, although performed with software guidance that leads the operator step by step through the successive stages of analysis after acquisition of the four-chamber view, becomes challenging to interpret. Previous studies have shown variable reproducibility and repeatability across parameters generated by FetalHQ [3,4]. Parameters related to chamber area generally perform well, and strain-related parameters show moderate repeatability, whereas secondary parameters derived mathematically from other measurable variables, such as the sphericity index (SI) or fractional shortening (FS), appear less robust [3,4,5]. It is also worth noting that reproducibility as measured by Intraclass Correlation Coefficient (ICC) differs substantially across published studies [6,7]. Fetal speckle tracking faces multiple challenges related to the specific nature of fetal imaging. The fetal cardiac ventricles are much smaller than those of adults, which limits the number of available pixels; acoustic shadowing is common; the entire fetus moves; fetal breathing-like movements and diaphragmatic motion are present; and, importantly, fetal cooperation during the examination remains beyond the scope of contemporary medicine.

In the present study, we did not focus on ICC-based evaluation of individual parameters generated by the software. Given the available literature suggesting variable repeatability across metrics, we instead focused on how large the typical difference is between two readings of the same cine loop, what magnitude of change must be observed to consider it real rather than attributable to measurement noise, and whether, because of the current level of measurement noise, two readings could shift a fetus from the “normal” category to the “abnormal” category despite the fact that nothing has objectively changed. Accordingly, we analyzed clinically relevant metrics, including feasibility/usable pairs, minimal detectable change (MDC95), Bland–Altman agreement, and threshold instability/classification flips. In addition, we defined the grey zone around each threshold as ±MDC95/2 and calculated a theoretical mean-of-two estimate to quantify the expected reduction in random measurement error under the assumption of independent measurement errors. Overall, the main objective was to estimate decision limits and threshold instability for selected key metrics, whereas the remaining analyses were supportive. Therefore, the aim of our study was not to provide a general assessment of the reproducibility of FetalHQ, but rather to evaluate analytical repeatability under conditions of zero biological variability.

## 2. Materials and Methods

### 2.1. Study Design and Study Population

We conducted a methodological study evaluating the analytical repeatability of outputs generated by Fetal Heart Quantification (FetalHQ) software in two-dimensional speckle-tracking (2D-STE) analysis under the assumption of zero biological variability. The analysis included 93 examinations with paired outputs labeled _1 and _2, obtained from the same recorded four-chamber view (4CV) cine loop.

The study material was derived from patients examined as part of routine second- and third-trimester screening at the Department of Obstetrics and Gynecology, Provincial Specialist Hospital in Kielce, Poland. The study was approved by the Bioethics Committee of Jan Kochanowski University in Kielce (No. 35/2024; 8 May 2024), and all patients provided informed consent for the ultrasound examination and participation in the study. We included only patients with a normal fetal cardiac appearance on ultrasound and without any other detectable abnormalities in fetal anatomy or growth.

### 2.2. Image Acquisition and FetalHQ Protocol

All examinations were performed using a Voluson Expert 22 system BT24 EC410 (GE) equipped with a C2-9-D transducer. Speckle-tracking analyses were performed using FetalHQ software (September 2024 version) according to the manufacturer’s instructions and with reference to ISUOG-recommended 4CV imaging standards, modified as required for FetalHQ analysis (maximal cardiac magnification, minimization of the insonation angle to achieve a high frame rate, and acquisition in a plane allowing reliable endocardial border tracking). Cine loops were approximately 5 s in duration.

Before FetalHQ analysis, each cine loop was assessed for technical suitability. An acceptable recording required an adequate four-chamber plane with both ventricles visualized without relevant foreshortening, sufficient cardiac magnification, a frame rate optimized by minimizing the insonation angle, absence of major fetal or transducer motion during the cardiac cycle selected for analysis, and continuous visualization of the ventricular endocardial borders. No fixed numerical minimum frame-rate threshold was prespecified; frame rate was optimized individually by minimizing the insonation angle while maintaining an adequate four-chamber plane and continuous endocardial visualization. Cine loops in which acoustic shadowing, motion, or loss of endocardial definition prevented reliable identification of the predefined anatomical landmarks or ventricular borders were considered technically inadequate for tracking.

Following initialization of the ventricular contours, tracking was visually reviewed throughout the complete selected cardiac cycle. Tracking was considered acceptable when the generated contour remained aligned with the visible endocardial border throughout the cycle without relevant loss of tracking or displacement into the ventricular cavity or myocardium. When localized mismatch occurred, the contour was manually corrected and tracking was reassessed. An analysis was not accepted when adequate tracking could not be achieved despite manual contour correction. The same technical acceptance criteria and correction procedure were applied by both operators.

In the standard FetalHQ workflow, the operator first performed morphometric measurements, including 4CV length and width for calculation of the global sphericity index. Cardiac-cycle selection was then performed manually using anatomical M-mode. The M-mode line was traced from the cardiac apex along the lateral wall of the right ventricle, passing through the tricuspid valve leaflet, while avoiding image drift. Within a single cardiac cycle, three temporal landmarks were manually identified: end-diastole at the beginning of the cycle (ED1), end-diastole at the end of the cycle (ED2), and the intervening end-systolic time point (ES), corresponding to maximal closure of the atrioventricular valves. Cardiac-cycle phase selection was therefore operator-dependent and was not automatically determined by the software or based solely on maximal or minimal ventricular dimensions.

**Figure 1:**
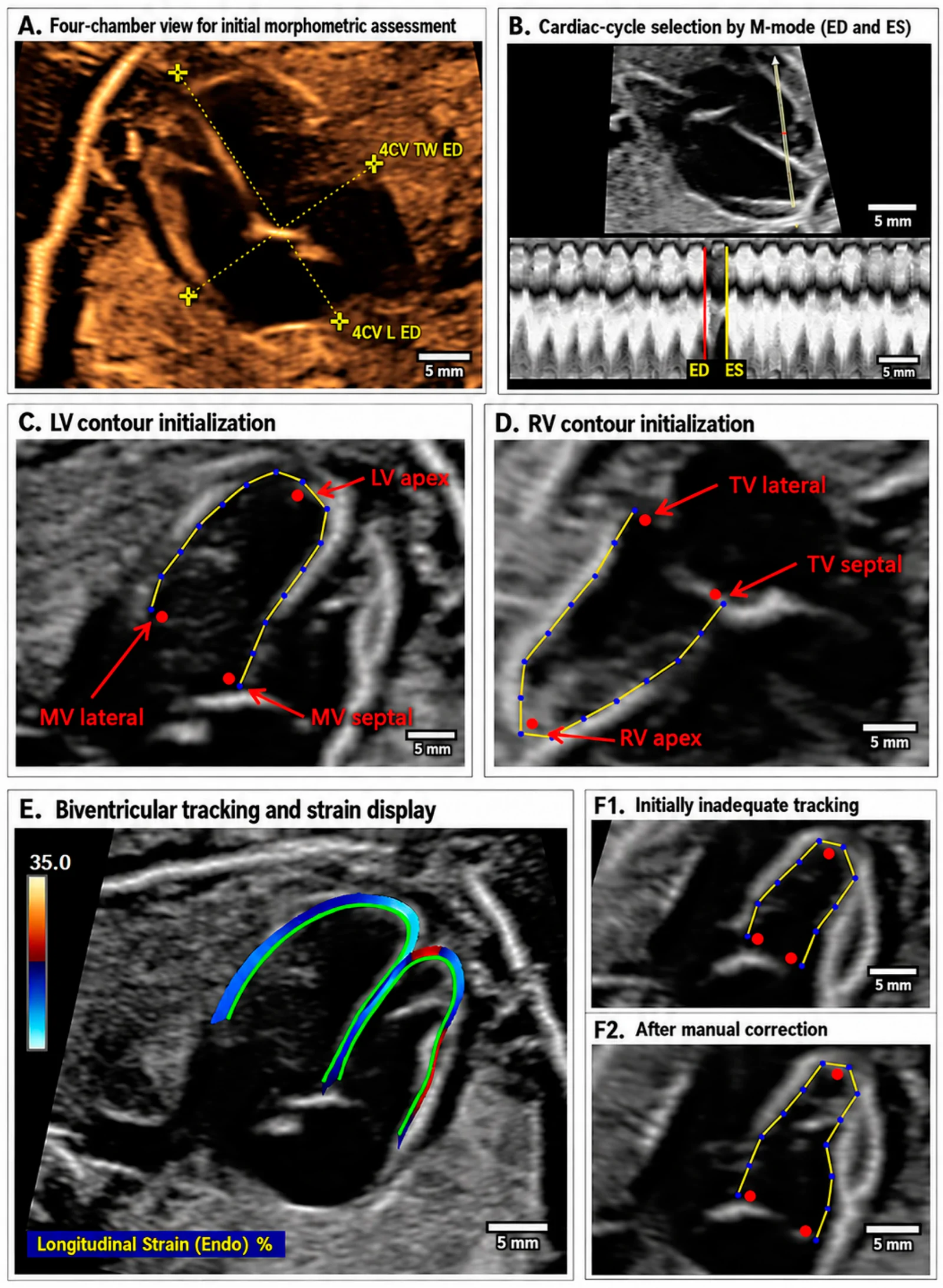
Visual workflow of FetalHQ ventricular analysis. (**A**) Representative four-chamber view used for the initial morphometric assessment, including four-chamber length and transverse width measurements at end-diastole. (**B**) Cardiac-cycle selection using anatomical M-mode, with identification of the selected end-diastolic (ED) and end-systolic (ES) time points. (**C**) Left ventricular (LV) contour initialization based on three predefined anatomical landmarks: the septal mitral valve insertion (MV septal), the lateral mitral valve insertion (MV lateral), and the LV endocardial apex (LV apex). (**D**) Right ventricular (RV) contour initialization based on the corresponding three anatomical landmarks: the septal tricuspid valve insertion (TV septal), the lateral tricuspid valve insertion (TV lateral), and the RV endocardial apex (RV apex). The septal and lateral tricuspid valve insertion points correspond to the annular landmarks used for calculation of RV TAPSE septal and RV TAPSE lateral, respectively. (**E**) Final biventricular endocardial tracking with the associated longitudinal strain display. (**F1**) Example of initially inadequate endocardial tracking, with contour propagation not fully aligned with the visible endocardial border. (**F2**) The same analysis after manual contour correction, demonstrating improved alignment of the tracking contour with the visible endocardial interface. Analyses in which satisfactory endocardial tracking could not be achieved despite manual contour correction were considered technically unacceptable. Abbreviations: LV, left ventricle; RV, right ventricle; MV, mitral valve; TV, tricuspid valve; ED, end-diastole; ES, end-systole; TAPSE, tricuspid annular plane systolic excursion.

Endocardial contouring was performed according to the same predefined protocol for both operators. For each ventricle, contour initialization was based on three anatomical landmarks: the septal atrioventricular valve insertion, the atrioventricular valve insertion on the free wall, and the endocardial apex. Accordingly, the left ventricular contour was initialized using the septal and lateral mitral valve insertion points and the LV apex, whereas the corresponding septal and lateral tricuspid valve insertion points and the RV apex were used for the right ventricle. The interventricular septal border formed part of the endocardial contour between the septal atrioventricular valve insertion and the apical point. In accordance with the predefined FetalHQ tracing protocol, papillary muscles and the right ventricular moderator band were included in the outline. Following initialization, the software generated the endocardial tracking, which was reviewed throughout the selected cardiac cycle by the operator. Manual contour correction was performed when the automatically generated tracking did not adequately follow the visible endocardial border. The same anatomical landmarks, contouring rules, and criteria for manual correction were applied by both operators. The algorithm subsequently divided each ventricular chamber into 24 segments and generated volumetric, functional, and strain-based outputs together with the corresponding normalized centiles and/or Z-scores. The principal steps of the FetalHQ analysis workflow are illustrated in Figure 1, including cardiac-cycle selection, LV and RV contour initialization, final biventricular tracking, and an example of initially inadequate tracking requiring manual contour correction.

RV TAPSE was derived by FetalHQ from speckle-tracked longitudinal displacement of the tricuspid annular insertion points between end-diastole and end-systole. RV TAPSE lateral represented systolic excursion of the lateral tricuspid annulus and was calculated from the change in distance between the RV apex and the lateral tricuspid valve insertion point. RV TAPSE septal represented the corresponding excursion of the septal tricuspid annulus and was calculated from the change in distance between the RV apex and the septal tricuspid valve insertion point. For both parameters, annular plane systolic excursion corresponds to the end-diastolic apex-to-annulus length minus the corresponding end-systolic length. Thus, these outputs were derived from the same tracked cardiac cycle and ventricular landmarks used for the FetalHQ analysis rather than from a separate conventional M-mode TAPSE measurement [8]. The definitions, derivation methods, cardiac-cycle timing, and principal operator-dependent components of the analyzed FetalHQ parameters are summarized in Table 1.

### 2.3. Definition of Intra-Operator and Inter-Operator Comparisons and Data Export

Two subsets were distinguished in the analysis:

- Intra-operator: the first 34 observations, representing repeat analysis of the same cine loop by a single ultrasonographer, yielding two outputs (_1 and _2);
- Inter-operator: the subsequent 59 observations, representing two independent readings of the same cine loop performed by two operators.

For intra-operator assessment, the same stored cine loop was analyzed twice by the same operator on separate occasions, with an interval of several days between analyses to avoid immediate-repetition bias. The exact interval was not prospectively recorded for individual cases. For inter-operator assessment, both operators analyzed the same stored cine loop, with the second operator blinded to the results of the first analysis. The exact cardiac cycle and end-diastolic frame were not fixed across repeated analyses. For each reading, the operator independently positioned the anatomical M-mode line and selected a suitable cardiac cycle within the same cine loop. Consequently, the selected cycle and frame could differ between repeated analyses.

Both operators were obstetricians with more than seven years of experience in prenatal diagnostics and second-trimester fetal screening. They were not dedicated fetal echocardiography specialists. Their experience specifically with FetalHQ was more limited. The local FetalHQ protocol was established on the basis of the manufacturer’s guidelines, original methodological publications, consensus documents, and educational materials, including Voluson Club resources. No formal manufacturer-certified FetalHQ training course was undertaken; instead, both operators standardized their analytical approach before the study analyses using the same manufacturer-provided and published methodological materials. Both operators followed the same predefined workflow for cardiac-cycle selection, landmark placement, endocardial contouring, manual correction, and tracking acceptance.

Raw outputs were exported from FetalHQ in CSV format and reshaped into a wide-format dataset, in which each metric was represented as a pair of variables with the suffixes _1 and _2. At the design stage, the 4CV GSI parameter (and its derived variables) was excluded because of the specific characteristics of this metric and the risk of export-related artifacts in this dataset.

### 2.4. Statistical Analysis

#### 2.4.1. General Assumptions and Data Description

Analyses were performed separately for the intra-operator and inter-operator subsets. Continuous variables were described as mean ± SD or as median and interquartile range (IQR) when distributions were clearly skewed, whereas categorical variables were reported as counts and percentages. Statistical significance was set at α = 0.05, and all tests were two-sided.

#### 2.4.2. Post-Export Numerical Quality Control and Definition of a Usable Pair

Technical image and tracking quality were assessed during the FetalHQ analysis itself, as described in Section 2.2. The quality-control procedure described below represents a separate post-export numerical plausibility screen and was not intended to substitute for assessment of image or tracking quality.

For each metric, a usable pair was defined as an observation meeting both of the following numerical criteria: (1) availability of both values (_1 and _2, with no missing data) and (2) values falling within the physiologic or logically admissible range for that metric. The post-export numerical plausibility rules included, among others, the following criteria: volumes and flow-related parameters >0; EF and FRAC within the range of 0% to 100%; raw strain values between −60% and +10%; TAPSE within the range of 0 to 10 mm; and normative scales within the ranges of 0 to 100 for centiles and −10 to +10 for Z-scores. For some parameters, normalized outputs remained available even when the corresponding raw metric was rejected; therefore, the number of usable pairs could differ between raw and normalized outputs. Raw and normalized variables were exported by FetalHQ as separate output fields and were subjected to scale-specific post-export plausibility criteria. Consequently, a normalized centile or Z-score could remain available and fall within the admissible normalized range even when the corresponding raw value was excluded by the plausibility limits defined for the raw metric. Such normalized outputs were retained for the threshold-instability analysis because they represented directly exported FetalHQ outputs; their retention should not be interpreted as restoring the validity of the corresponding excluded raw measurement.

For descriptive reporting of non-usable pairs, two categories were distinguished: (1) missing or incomplete paired output, defined as absence of one or both values required to form a pair, and (2) exclusion by post-export numerical quality control, defined as a complete paired output in which at least one value fell outside the prespecified plausibility range. The archived export did not contain reason codes allowing missing or incomplete outputs to be retrospectively subdivided into inadequate image acquisition, inadequate tracking, incomplete analysis, or software/export failure. Moreover, because the present study was based on previously stored cine loops selected for analysis, failures occurring before cine-loop storage were not represented in the analytical dataset.

Accordingly, numerical QC was applied only after completion of the image-level and tracking-level assessment. Values passing the numerical plausibility limits should therefore not be interpreted as having been accepted solely on the basis of their numerical range. Detailed post-export quality-control rules and preprocessing steps are provided in Supplement S2.

#### 2.4.3. Decision Limits: Bland–Altman Agreement and Minimal Detectable Change (MDC95)

For each metric, the difference was defined as Δ = value_2 − value_1, and the following were calculated: (1) bias, defined as the mean of Δ; (2) 95% limits of agreement (LoAs) according to the Bland–Altman method, calculated as bias ± 1.96 × SD(Δ); and (3) MDC95 (minimal detectable change), calculated as 1.96 × SD(Δ). In the present same-cine-loop design, MDC95 was interpreted as an analysis-level decision limit describing repeat-analysis measurement variability under conditions in which biological and repeat-acquisition variability were intentionally excluded, rather than as a threshold for clinically meaningful longitudinal change between separate examinations. In addition, we estimated the effect of a mean-of-two workflow (the average of two independent tracings) on reducing measurement uncertainty, using the conventional approximation: MDC95_mean-of-two = MDC95/√2. This calculation represents a theoretical approximation based on the assumption that the two measurement errors are independent and have comparable variance. The study did not prospectively compare a single-analysis workflow with an independently acquired two-analysis strategy.

#### 2.4.4. Threshold Instability of Normative Cutoffs and the Grey Zone

Threshold instability was analyzed for prespecified normalized outputs exported directly by FetalHQ. Normalized centiles and Z-scores were not recalculated by the investigators but were taken directly from the parameter-specific outputs generated by the software. According to the manufacturer’s documentation, FetalHQ provides Z-scores and percentiles for fetal cardiac measurements together with integrated nomograms based on published reference data [9]. The primary analysis included LV EDV centile (GA), LV EF centile (GA), RV TAPSE lateral centile (GA), LV GLS centile, and LV GLS Z-score. For outputs explicitly identified as gestational-age-dependent in the FetalHQ export, namely LV EDV centile (GA), LV EF centile (GA), and RV TAPSE lateral centile (GA), gestational age was incorporated by the software as part of the normalization process; no additional gestational-age adjustment was performed by the investigators. LV GLS was evaluated using the centile and Z-score outputs provided directly by the software. The investigators did not reconstruct or modify the underlying normative equations. Because the available manufacturer documentation does not provide a complete parameter-by-parameter mapping of every normalized output to its original reference cohort, we do not assume that all analyzed normative parameters derive from a single common reference population. The analysis of classification flips was prespecified and restricted to clinically typical cutoffs: <5 and >95 for centile-based metrics and <−2 and >2 for Z-score-based metrics. Threshold analyses were performed only for complete pairs with values within the admissible range of the normative scale. The grey-zone analysis in the main manuscript was limited to the thresholds considered most clinically relevant: LV EDV centile (GA) <5, LV EF centile (GA) <5, RV TAPSE lateral centile (GA) <5, LV GLS centile >95, and LV GLS Z-score >2. The grey zone around each threshold was then defined as ±(MDC95/2) on the same scale (centile or Z-score), and the proportion of cases in which the mean of the two readings fell within this zone was calculated. This interval was defined as an exploratory descriptive construct centered on the normative threshold, with a total width equal to one MDC95. The choice of ±MDC95/2 was intended to visualize the region around a cutoff in which repeat-analysis variability could plausibly contribute to threshold crossing. It does not represent a confidence interval, an individualized probability of misclassification, or a validated clinical decision boundary. Full results for all analyzed normative metrics and thresholds are provided in Supplement S3.

#### 2.4.5. Proportional Bias and Heteroscedasticity

To assess whether measurement error depended on the magnitude of the measured value, proportional bias was tested using linear regression of Δ ~ pair mean, whereas heteroscedasticity was assessed using Spearman correlation for |Δ| ~ pair mean.

#### 2.4.6. Software and Libraries

All analyses were performed in Python 3.12.14 [10] pandas 3.0.3 [11] and NumPy 2.5.0 [12] were used for tabular data processing and array-based operations; SciPy 1.18.0 [13] was used for statistical testing and correlation analyses, including Spearman correlation; regression models (Δ ~ pair mean) were estimated using statsmodels [14]; figures, including Bland–Altman plots, were generated using matplotlib [15]; and export of tables to XLSX files was performed using openpyxl 3.1.5. [16].

## 3. Results

The analysis included 93 examinations with paired measurements (_1 and _2) obtained from the same cine loop (repeat analysis/tracing without a biological variability component). The intra-operator subset included 34 observations, and the inter-operator subset included 59 observations.

Gestational age (clinical GA) was as follows:

- Intra-operator: mean 22.38 ± 4.05 weeks, range 16.71–35.43 weeks;
- Inter-operator: mean 22.51 ± 4.14 weeks, range 18.29–36.43 weeks.

For each parameter, paired measurements were classified as usable, missing/incomplete, or excluded by post-export numerical quality control. The distribution of these categories for the principal FetalHQ parameters is presented in Table 2.

The first 34 cases were treated as intra-operator repeats, whereas the remaining 59 were treated as inter-operator repeats.

The highest proportion of usable pairs was observed for LV EDV and ESV, whereas the lowest was observed for TAPSE, particularly septal TAPSE. In the intra-operator subset, four examinations had missing or incomplete paired output across the principal parameters; additional exclusions resulted from post-export numerical QC and were more frequent for functional parameters and TAPSE. In the inter-operator subset, three examinations had missing or incomplete paired output for most principal parameters, with one additional incomplete pair for septal TAPSE. The remaining non-usable pairs resulted from numerical QC exclusions. The available export did not permit reliable retrospective attribution of missing/incomplete outputs to tracking failure, incomplete analysis, or software/export failure.

Decision limits: Bland–Altman agreement and MDC95

For each metric, we calculated the difference Δ = _2 − _1, the bias (mean difference), the 95% Bland–Altman limits of agreement (LoAs), and MDC95 = 1.96 × SD(Δ), representing the magnitude of repeat-analysis variability under the same-cine-loop conditions of the present study. The results are summarized in Table 3.

LV volumetric metrics (EDV/ESV) were characterized by minimal bias and relatively narrow LoA, whereas contractility and deformation parameters (EF, FRAC, and GLS) showed substantially wider LoA and higher MDC95 values, particularly in the inter-operator analysis. TAPSE, especially septal TAPSE, was characterized by both lower usability (Table 2) and wide LoA. A complete summary of all parameters, including bias, LoA, Sw, MDC95, and the proportion of usable pairs, is provided in Supplement S1.

### 3.1. Threshold Instability of Normative Classification

For selected normative parameters, we assessed how often classification changed between _1 and _2 (classification flips) at predefined centile- and Z-score-based thresholds. These analyses were performed only for pairs with complete values and values within the admissible range of the normative scale (centiles 0–100; Z-scores −10 to +10). The results are presented in Table 4.

The greatest classification instability was observed for normalized functional parameters, particularly LV EF centile <5 and LV GLS Z-score >2, with a clearly higher proportion of classification flips in the inter-operator analysis than in the intra-operator analysis. In practical terms, this means that, near normative thresholds, some results may shift between the “normal” and “abnormal” categories solely because of measurement uncertainty. These findings motivated the exploratory grey-zone analysis and the theoretical evaluation of a mean-of-two approach presented in the following sections. The results of the threshold instability analysis for all normative parameters (centiles and Z-scores) are presented in Supplement S3.

### 3.2. Proportional Bias and Heteroscedasticity (Analysis of Δ Relative to the Pair Mean)

We assessed whether differences between repeated measurements (Δ = _2 − _1) showed proportional bias (i.e., a nonzero regression slope for Δ ~ pair mean) and whether heteroscedasticity was present (i.e., an increase in |Δ| with increasing pair mean). For the key parameters (LV EDV, LV EF, LV GLS, and RV TAPSE lateral), most metrics showed no significant proportional bias in either the intra-operator or inter-operator comparison (all *p* > 0.05), with the exception of a trend for LV EF in the intra-operator analysis (negative slope; *p* = 0.073). At the same time, LV EDV showed heteroscedasticity, with a positive correlation between |Δ| and the pair mean in both the inter-operator analysis (ρ = 0.500; *p* < 0.001) and the intra-operator analysis (ρ = 0.386; *p* = 0.035), suggesting greater dispersion of differences at higher EDV values (Table 5). No significant signals of heteroscedasticity were observed for LV EF, LV GLS, or RV TAPSE lateral.

### 3.3. Estimated Effect of Averaging 2 Independent Analyses (Mean-of-Two) on Decision Limits

For clinically key parameters, we calculated MDC95 for a single tracing and the theoretical estimate of MDC95 for the mean of two independent tracings (MDC95/√2), under the assumption of independent random measurement errors. The results are summarized in Table 6. By mathematical derivation, division of MDC95 by √2 yields an estimated reduction of approximately 29.3% for the mean of two independent analyses. This estimate was not derived from an observed comparison of single- versus two-analysis workflows or from a demonstrated reduction in classification flips. For example, the estimated inter-operator MDC95 for LV EF decreased from 40.1 to 28.4 percentage points, and for LV GLS, from 23.7 to 16.7 percentage points under this theoretical assumption.

### 3.4. Exploratory Grey Zone Around Normative Thresholds and Its Relationship with Classification Flips

To descriptively examine the relationship between repeat-analysis uncertainty and proximity to normative thresholds, we applied the prespecified exploratory grey zone of ±(MDC95/2) around each cutoff. We then calculated (i) the half-width of the grey zone, (ii) the proportion of cases in which the mean of the 2 readings fell within this zone, and (iii) the observed proportion of classification flips at the given threshold. The results are presented in Table 7.

In practical terms, the half-width of the grey zone was large on the centile scale (e.g., for LV EF centile [GA] <5, 26.44 centiles in the inter-operator analysis and 18.91 centiles in the intra-operator analysis), resulting in most cases falling within this zone (84.3% and 89.3%, respectively). For LV GLS Z-score >2, the half-width of the grey zone was 3.60 in the inter-operator analysis (78.4% of cases within the grey zone) and 2.53 in the intra-operator analysis (62.5% within the grey zone). For the threshold LV GLS Z-score >2, all observed classification flips occurred in cases located within the grey zone, whereas no flips were observed outside the grey zone.

In summary, the results of repeated analysis of the same cine loop showed a clear contrast between volumetric/geometric parameters and functional parameters. LV volumetric metrics (EDV/ESV) were characterized by high usability (Table 2) and relatively narrow limits of agreement with lower MDC95 values (Table 3), whereas tracking-based and/or derived functional parameters (EF, FRAC, GLS, and TAPSE) more often showed limited usability and wide decision limits, particularly in the inter-operator comparison. A direct consequence of these limitations was substantial threshold instability (Table 4), more pronounced in the inter-operator analysis, confirming the practical problem of classification flips near diagnostic thresholds. Analysis of the relationship between measurement error and measurement magnitude demonstrated heteroscedasticity for LV EDV (Table 5), whereas EF, GLS, and TAPSE showed no clear evidence of proportional bias. Under the theoretical assumption of independent random measurement error, averaging two analyses would be expected to reduce the estimated MDC95 by approximately 29.3% (Table 6). This reduction was mathematically derived and was not prospectively validated as a clinical strategy. At the same time, the defined grey zone (Table 7) indicates that a substantial proportion of normative results fall within the zone of uncertainty around the threshold, which illustrates the potential instability of single measurements obtained near normative thresholds. The grey-zone analysis should be interpreted as exploratory. Its width reflects the propagation of repeat-analysis variability onto the normalized centile or Z-score scale and, for several parameters, encompasses a large proportion of observations. It does not establish an individualized probability of misclassification or define a validated clinical decision interval. Prospective external validation is required before such zones can be used to guide clinical management or determine when repeat measurements should be obtained. For some parameters, the half-width of the grey zone is very wide, which may be biologically and clinically controversial because of the resulting ambiguity in interpretation. However, this is a consequence of the propagation of measurement error after transformation to the normative scale. Conceptually, this exploratory measure indicates that a single measurement near a normative threshold may yield an unstable classification.

A detailed fit-for-purpose ranking of the most and least actionable parameters, incorporating usability, MDC95/LoA, and the risk of classification flips, is provided in Supplement S6. Additional Bland–Altman plots and sensitivity analyses (as-is vs QC, transformations) are presented in Supplement S4 and S5.

## 4. Discussion

Our study shifts the emphasis away from the classical assessment of reproducibility and toward the level at which the clinical interpretability of the examination is determined, that is, from reliability to decision reliability. The key premise of this framework is the assumption of zero biological variability. Within this context, the method performs well for simpler volumetric metrics, but clearly less well for functional parameters and threshold-based classification. Therefore, the aim of our study was not to provide a general assessment of the reproducibility of FetalHQ, but rather to evaluate its analytical repeatability under conditions of zero biological variability.

A fundamental distinction should be made between the repeat-analysis setting evaluated here and full clinical test–retest reproducibility. In the present study, all repeated analyses were performed on the same previously acquired cine loop, although the exact frame and cardiac cycle were not necessarily identical between readings. Thus, the design captures uncertainty arising from the analytical workflow, including cardiac-cycle selection, M-mode line placement, endocardial contouring, manual correction, and tracking acceptance, while intentionally excluding variability associated with repeat image acquisition and biological change. Consequently, the present results should be interpreted as measures of same-cine-loop analytical repeatability rather than total reproducibility of a complete fetal cardiac examination.

The variability quantified in the present study should not be interpreted as intrinsic variability of the FetalHQ software algorithm alone. Consistent with our previously described pragmatic protocol [17], repeated analyses were performed on the same stored cine loop but did not require use of an identical frame or cardiac cycle. The operator independently positioned the anatomical M-mode line, selected the cardiac cycle, initialized the endocardial contours, performed manual corrections when required, and assessed tracking adequacy. Therefore, the observed variability reflects the complete operator–software analytical workflow, including both operator-dependent decisions and software-derived tracking and calculations. The present design does not allow these components to be separated quantitatively.

In our study, LV EDV and LV ESV showed the best performance: they had the highest proportion of usable pairs (88.2–94.9%), low bias, relatively narrow LoA, and low MDC95 values. This suggests comparatively greater analytical stability of ventricular volumetric and geometric measurements under the same-cine-loop conditions of the present study. In contrast, functional metrics such as EF, FRAC, GLS, and TAPSE performed substantially worse, with wider limits of agreement and higher MDC95 values. At these levels of uncertainty, the safe use of a single measurement is open to question. In clinical terms, for some outputs, classification flips across the centile threshold occurred in up to one-third of cases. This indicates that relying exclusively on a single reading, rather than on interpretation of the examination as a whole, may lead to error.

Accordingly, the MDC95 values reported here should be interpreted strictly within the same-cine-loop analytical setting. They quantify the magnitude of change required to exceed repeat-analysis variability when biological and repeat-acquisition variability are intentionally removed. They should not be used directly as thresholds for clinically meaningful change between separate fetal ultrasound examinations.

An additional finding of the present study is the limited analysis-level feasibility of several functional FetalHQ outputs. Even though all analyses were based on previously recorded cine loops selected as technically suitable for FetalHQ analysis, a relevant proportion of paired measurements for functional parameters, particularly TAPSE, could not be retained for the final paired analysis because of missing/incomplete output or failure of the post-export numerical plausibility criteria. Reduced usability should therefore not be regarded merely as missing data, but as a characteristic of the analytical workflow itself. Importantly, this limitation was observed under controlled same-cine-loop conditions, without repeat acquisition or biological variability. At the same time, these proportions should not be interpreted as overall clinical acquisition feasibility, because cine loops that failed before storage or selection for analysis were not captured in the present dataset.

To our knowledge, we did not identify any previous study that approached FetalHQ analysis in the same way as our group. Nevertheless, the available literature consistently shows substantial heterogeneity in repeated measurements, both when performed by the same operator and by 2 independent operators. In the study by Hata et al. [18], lower correlations for FS (fractional shortening) were observed mainly in the right ventricle. Our findings, which suggest uncertainty of a single measurement, may help explain why the assessment of physiologic fetal cardiac parameters during pregnancy differs substantially across studies. This issue was also discussed by Hata et al. [18]. Other studies likewise suggest discrepancies between reports, for example in the direction of change in FS with advancing gestational age [18,19].

In the study by Xiao et al. [20], lower LV GLS and 4CV GSI values were demonstrated in fetuses with FGR compared with controls. In contrast, another cohort showed no significant differences in global metrics (GSI, GLS, and EF) despite the presence of segmental changes [21]. Differences have also been reported for segmental FS. In one cohort, reduced FS was reported in LV segments 1–17 and RV segments 1–8 (*p* < 0.05), with no differences in the remaining segments [20]. In the second study, the authors emphasized segmental heterogeneity, indicating that some segments may show a different direction of change to others [21]. In other pregnancy complications, the literature also reports conflicting findings regarding the direction of strain changes in hypertensive disease of pregnancy [22,23] and differences in GSI in pregnancies complicated by hyperglycemia [24,25].

**Figure 2:**
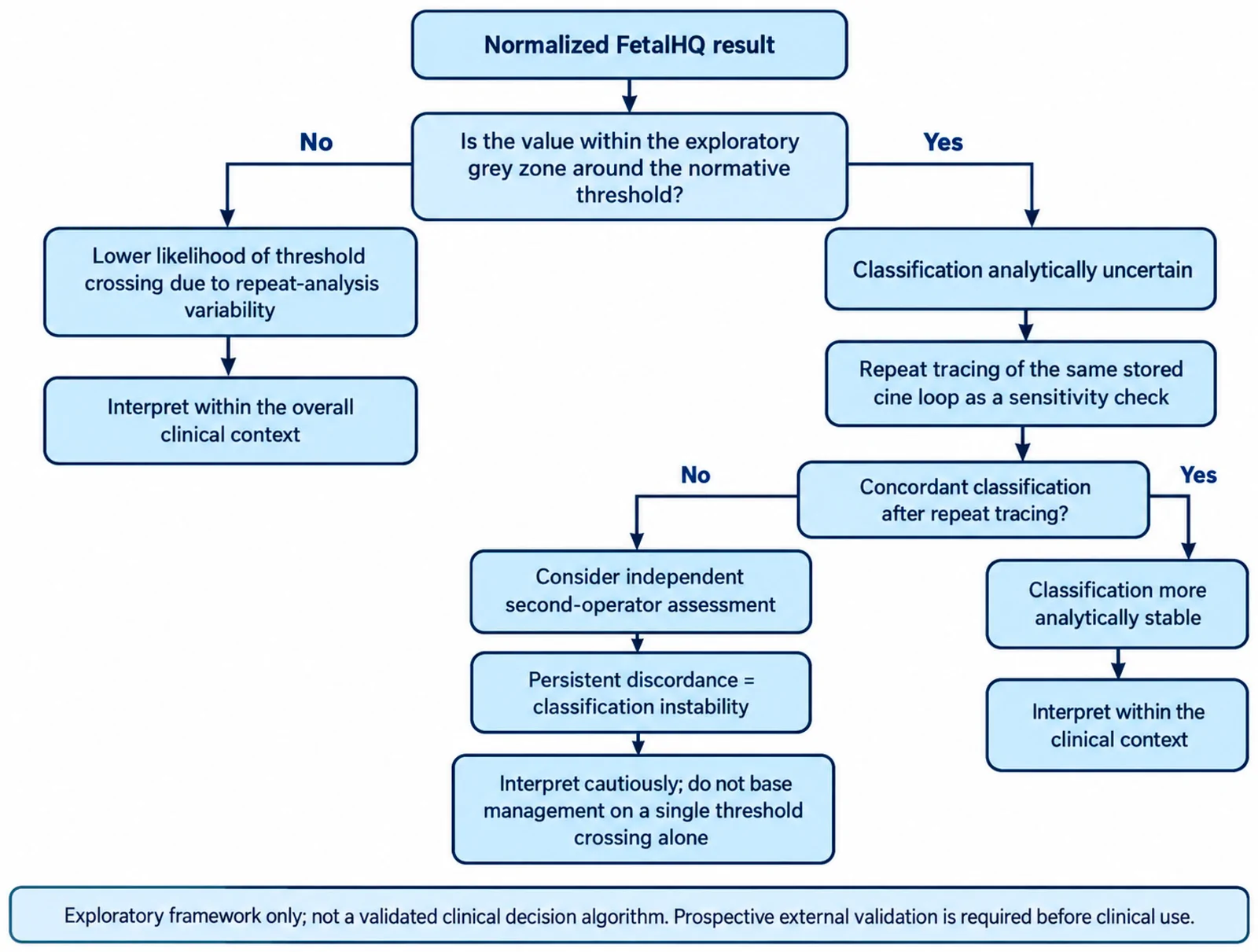
Exploratory interpretive framework for FetalHQ measurements near normative thresholds. Measurements falling within the exploratory grey zone are considered analytically uncertain with respect to threshold classification. Repeat tracing of the same stored cine loop and, in persistently discordant cases, independent second-operator assessment are presented as potential sensitivity checks. This schematic is intended to illustrate the implications of repeat-analysis variability and does not represent a validated clinical decision algorithm.

Based on our findings, it may be extrapolated that one partial explanation for the ambiguity of repeatability results and for so-called classification flips between physiologic and pathologic categories across studies is the lack of averaging, which is not routinely performed. Using the conventional theoretical approximation for averaging two independent measurements, the estimated MDC95 for the mean of two analyses equals MDC95/√2, corresponding to an expected reduction of approximately 29.3% in random measurement error. This reduction was not directly observed in a prospectively tested two-measurement protocol, and the present study did not demonstrate a corresponding reduction in classification flips. Moreover, the assumption of independent errors may not be fully satisfied because repeated analyses of the same cine loop can share common sources of error. Therefore, the mean-of-two results should be regarded as a theoretical estimate rather than a clinically validated strategy. However, if a given output already has wide LoA and a high MDC95, it may remain unstable even after averaging. In our study, this applies particularly to functional and deformation parameters such as EF, FRAC, and GLS, which may still retain relatively wide decision limits even when based on the mean of 2 measurements. From a practical perspective, a result close to a centile- or Z-score-based threshold should be interpreted cautiously because repeat analysis alone may alter classification. A repeat tracing or mean-of-two approach may theoretically reduce random measurement uncertainty; however, the present study did not prospectively test whether such a strategy reduces classification flips. Its clinical effectiveness therefore requires prospective validation. For practical interpretation, the present findings may be summarized in an exploratory stepwise framework rather than a validated clinical algorithm. First, measurements clearly outside the exploratory grey zone may be interpreted with lower concern for threshold instability attributable to repeat-analysis variability. Second, measurements falling within the grey zone should be regarded as analytically uncertain with respect to threshold classification. In such cases, a repeat tracing of the same cine loop may be considered as a sensitivity check. If repeated analyses remain discordant relative to the threshold, confirmation by an independent operator may provide additional information regarding operator-dependent variability. Importantly, this framework is intended only to illustrate how analytical uncertainty could be approached and should not be interpreted as a validated management pathway or as evidence that repeat tracing or second-operator assessment improves clinical outcomes (Figure 2). Awareness of these limitations may improve the rigor of future studies and support more reliable use of the method in clinical practice.

The principal clinical implication of the present study is therefore not simply that individual FetalHQ parameters differ in the width of their limits of agreement or MDC95. Rather, for several functional and normalized outputs, repeat analysis of the same stored cine loop was sufficient to change classification relative to a normative threshold despite the absence of biological change or repeat image acquisition. Under these same-cine-loop analytical conditions, LV volumetric parameters, particularly EDV and ESV, showed the greatest stability and usability. In contrast, EF, FRAC, GLS, and TAPSE showed lower usability, wider decision limits, and/or greater threshold instability and should therefore be interpreted with greater caution. Importantly, this relative ranking applies only to analytical repeatability of stored cine loops and should not be interpreted as evidence that any parameter has been validated for serial clinical follow-up between separate examinations.

Our study has several limitations. First, it was a single-center study. Second, although both operators had more than seven years of experience in prenatal diagnostics and second-trimester fetal screening, their specific experience with FetalHQ was more limited, which may have contributed to operator-dependent variability. The physicians performing the analyses learned the technique from the manufacturer’s official materials and repositories. At the same time, the software itself guides the user step by step through the analysis workflow. The reduced proportion of usable pairs for several functional metrics should be regarded as an analysis-level feasibility finding of the study rather than solely as missing data. At the same time, the resulting smaller effective sample sizes for these parameters remain a statistical limitation because they may reduce the precision and stability of the corresponding LoA and MDC95 estimates. The present study did not evaluate between-visit, between-machine, or repeat-acquisition variability. A separate clinical examination introduces additional sources of uncertainty that were intentionally absent from the present same-cine-loop design, including changes in fetal position, acquisition plane, cardiac magnification, insonation angle and frame rate, image quality, gestational age, and biological state. Therefore, the total test–retest uncertainty associated with serial clinical examinations is likely to be greater than the repeat-analysis uncertainty estimated in the present study. Although intra-operator analyses were performed on separate occasions with an interval of several days, the exact interval was not prospectively recorded. A residual recall effect cannot therefore be completely excluded. If present, such recall could have reduced the observed intra-operator differences and therefore made intra-operator repeatability appear more favorable than it would be under fully independent repeat-analysis conditions. However, the protocol was specifically designed to avoid immediate-repetition bias. Because repeat image acquisition was not part of the study design, the present data cannot quantify total between-examination test–retest variability in clinical practice.

## 5. Conclusions

FetalHQ showed the greatest same-cine-loop analytical stability for LV volumetric parameters, particularly EDV and ESV, whereas EF, FRAC, GLS, and TAPSE were more susceptible to measurement variability, reduced usability, and/or classification instability. Importantly, repeat analysis of the same stored cine loop was sufficient to move some normalized measurements across conventional normative thresholds despite the absence of biological change or repeat image acquisition. A theoretical mean-of-two approach may reduce random measurement uncertainty, but this strategy was not prospectively validated in the present study. These findings characterize same-cine-loop analytical repeatability and should not be interpreted as estimates of total test–retest reproducibility between separate fetal ultrasound examinations.

## Figures and Tables

**Table 1:** Definitions and operator-dependent components of the principal FetalHQ-derived parameters.

Parameter	Unit	Anatomical/Mathematical Definition	Cardiac-Cycle Timing	Measurement/Derivation Type	Operator-Dependent Steps
**LV EDV**	mL	Left ventricular end-diastolic volume calculated from the endocardial geometry of the LV using the FetalHQ volume model.	End-diastole (ED)	Geometry-derived	Cardiac-cycle selection; placement of anatomical landmarks; LV endocardial contour initialization; manual contour correction when required; tracking acceptance.
**LV ESV**	mL	Left ventricular end-systolic volume calculated from the endocardial geometry of the LV.	End-systole (ES)	Geometry-derived	Cardiac-cycle selection; placement of anatomical landmarks; LV endocardial contour initialization; manual contour correction when required; tracking acceptance.
**LV SV**	mL	Left ventricular stroke volume, calculated as EDV−ESV.	ED and ES of the selected cardiac cycle	Mathematically derived	No additional operator steps beyond those required for EDV and ESV determination.
**LV CO**	mL/min	Left ventricular cardiac output, calculated from stroke volume and fetal heart rate.	Selected cardiac cycle	Mathematically derived	No additional operator step beyond the source measurements.
**LV EF**	%	Left ventricular ejection fraction, calculated as (EDV−ESV)/EDV × 100.	ED and ES	Mathematically derived	No additional operator steps beyond those required for EDV and ESV determination.
**LV FRAC**	%	Left ventricular fractional area change, calculated from the change in ventricular area between end-diastole and end-systole.	ED and ES	Geometry-derived/mathematically derived	Cardiac-cycle selection; LV contour definition and tracking acceptance.
**LV GLS**	%	Left ventricular global longitudinal strain derived from longitudinal deformation of the tracked LV endocardial contour during the selected cardiac cycle.	Across the selected cardiac cycle	Speckle-tracking-derived	Cardiac-cycle selection; landmark placement; LV endocardial contour initialization; manual contour correction when required; tracking acceptance.
**RV TAPSE lateral**	mm	Systolic longitudinal excursion of the lateral tricuspid annular insertion, derived from the change in apex-to-lateral-annulus distance between ED and ES.	ED to ES	Tracking-derived	Cardiac-cycle selection; RV apical and lateral tricuspid annular landmark placement; contour/tracking assessment and correction when required.
**RV TAPSE septal**	mm	Systolic longitudinal excursion of the septal tricuspid annular insertion, derived from the change in apex-to-septal-annulus distance between ED and ES.	ED to ES	Tracking-derived	Cardiac-cycle selection; RV apical and septal tricuspid annular landmark placement; contour/tracking assessment and correction when required.

Note: The table summarizes the anatomical or mathematical basis of each output, the relevant cardiac-cycle phase, and the principal steps requiring operator input during FetalHQ analysis. Geometry- and tracking-derived parameters are generated by the software from the operator-defined cardiac cycle, anatomical landmarks, and ventricular contours, whereas mathematically derived parameters are calculated from these source measurements. Abbreviations: LV, left ventricle; RV, right ventricle; ED, end-diastole; ES, end-systole; EDV, end-diastolic volume; ESV, end-systolic volume; SV, stroke volume; CO, cardiac output; EF, ejection fraction; FRAC, fractional area change; GLS, global longitudinal strain; TAPSE, tricuspid annular plane systolic excursion. The operator-dependent steps listed above indicate that the variability quantified in this study reflects the complete operator–software analytical workflow rather than the intrinsic variability of the software algorithm alone.

**Table 2:** Breakdown of non-usable paired measurements by identifiable post-export cause.

Metric	Intra-Operator Usable Pairs, *n*/N (%)	Missing/Incomplete Output, *n*	Excluded by Numerical QC, *n*	Inter-Operator Usable Pairs, *n*/N (%)	Missing/Incomplete Output, *n*	Excluded by Numerical QC, *n*
LV EDV [mL]	30/34 (88.2%)	4	0	56/59 (94.9%)	3	0
LV ESV [mL]	30/34 (88.2%)	4	0	56/59 (94.9%)	3	0
LV SV [mL]	22/34 (64.7%)	4	8	45/59 (76.3%)	3	11
LV CO [mL/min]	22/34 (64.7%)	4	8	45/59 (76.3%)	3	11
LV EF [%]	22/34 (64.7%)	4	8	45/59 (76.3%)	3	11
LV FRAC [%]	22/34 (64.7%)	4	8	45/59 (76.3%)	3	11
LV GLS [%]	23/34 (67.6%)	4	7	50/59 (84.7%)	3	6
RV TAPSE lateral [mm]	17/34 (50.0%)	4	13	45/59 (76.3%)	3	11
RV TAPSE septal [mm]	14/34 (41.2%)	4	16	41/59 (69.5%)	4	14

Notes: Usable pairs were complete paired observations (_1 and _2) meeting the prespecified post-export numerical plausibility criteria. Missing/incomplete output indicates that one or both values required to form a paired result were absent from the archived FetalHQ export. The archived export did not encode the upstream reason for an absent output; therefore, inability to obtain satisfactory tracking, incomplete analysis, and software/export-related failure could not be distinguished retrospectively. Excluded by numerical QC indicates a complete paired output in which at least one value fell outside the prespecified numerical plausibility limits. Numerical plausibility limits for the key raw metrics were: volumes and flow-related parameters >0; EF and FRAC 0–100%; raw strain −60% to +10%; and TAPSE 0–10 mm. Four intra-operator cases and three inter-operator cases lacked a complete second FetalHQ output across the principal metrics. One additional inter-operator case had parameter-specific absence of septal TAPSE despite otherwise available paired outputs. Because the study started from previously stored cine loops, acquisition failures occurring before a cine loop was stored or selected were not captured; therefore, these data describe analysis-level feasibility rather than overall clinical acquisition feasibility. Abbreviations: LV, left ventricle; RV, right ventricle; EDV, end-diastolic volume; ESV, end-systolic volume; SV, stroke volume; CO, cardiac output; EF, ejection fraction; FRAC, fractional area change; GLS, global longitudinal strain; TAPSE, tricuspid annular plane systolic excursion; QC, quality control.

**Table 3:** Decision limits (bias, 95% LoA) and MDC95 for key parameters after quality-control filtering.

Metric	Intra-Bias	Intra-95% LoA	Intra-MDC95	Inter-Bias	Inter-95% LoA	Inter-MDC95
LV EDV [mL]	0.032	−0.664 to 0.728	0.696	−0.024	−0.401 to 0.353	0.377
LV ESV [mL]	−0.025	−0.433 to 0.382	0.408	−0.038	−0.501 to 0.426	0.464
LV SV [mL]	0.071	−0.392 to 0.533	0.462	0,027	−0.373 to 0.427	0.400
LV CO [mL/min]	11.800	−55.7 to 79.3	6.,500	3000	−47.5 to 53.6	50,600
LV EF [%]	−2400	−33.5 to 28.6	31.000	2700	−37.5 to 42.8	40,100
LV FRAC [%]	−2200	−25.9 to 21.5	23.700	1400	−29.2 to 31.9	30,500
LV GLS [%]	1700	−15.1 to 18.4	16.800	−0.600	−24.3 to 23.0	23,700
RV TAPSE lateral [mm]	0.010	−2.44 to 2.46	2450	0.580	−3.18 to 4.35	3760
RV TAPSE septal [mm]	−0.480	−4.77 to 3.81	4290	0.350	−2.83 to 3.53	3180

Bias was defined as value_2 − value_1. Bland–Altman 95% limits of agreement (LoAs) were calculated as bias ± 1.96 × SD(diff). MDC95 was calculated as 1.96 × SD(diff) and represents the magnitude of repeat-analysis variability under the same-cine-loop conditions used in this study. It should not be interpreted as a threshold for clinically meaningful longitudinal change between separate fetal examinations.

**Table 4:** Threshold instability (classification flips) for normalized outputs.

Metric	Threshold	Intra-Flips/*n* (%)	Inter-Flips/*n* (%)
LV EDV centile (GA)	<5	7/28 (25.0%)	9/51 (17.6%)
LV EDV centile (GA)	>95	0/28 (0.0%)	6/51 (11.8%)
LV EF centile (GA)	<5	5/28 (17.9%)	17/51 (33.3%)
LV EF centile (GA)	>95	0/28 (0.0%)	2/51 (3.9%)
RV TAPSE lateral centile (GA)	<5	7/28 (25.0%)	16/51 (31.4%)
RV TAPSE lateral centile (GA)	>95	1/28 (3.6%)	4/51 (7.8%)
LV GLS centile	<5	4/30 (13.3%)	11/56 (19.6%)
LV GLS centile	>95	4/30 (13.3%)	20/56 (35.7%)
LV GLS Z-score	<−2	2/24 (8.3%)	10/51 (19.6%)
LV GLS Z-score	>2	4/24 (16.7%)	20/51 (39.2%)

Flips indicate discordant classification between measurement 1 and measurement 2 at the specified threshold. GA-based centiles were analyzed for LV EDV, LV EF, and RV TAPSE lateral, whereas LV GLS was analyzed both as centile and as Z-score, according to the normalized outputs available in the FetalHQ export. Normalized outputs were exported as separate FetalHQ variables and were evaluated using their own admissible scale. Therefore, the denominator of a normalized parameter may differ from that of the corresponding raw metric.

**Table 5:** Proportional bias and heteroscedasticity for selected metrics.

Group	Metric	Usable *n*	Slope of Δ~Mean	*p*	Spearman ρ of |Δ|~Mean	*p*
Inter-operator	LV EDV [mL]	56	0.059	0.103	0.500	<0.001
Inter-operator	LV EF [%]	45	−0.198	0.416	−0.152	0.317
Inter-operator	LV GLS [%]	50	−0.050	0.793	−0.110	0.446
Inter-operator	RV TAPSE lateral [mm]	45	0.241	0.278	0.150	0.325
Intra-operator	LV EDV [mL]	30	0.120	0.184	0.386	0.035
Intra-operator	LV EF [%]	22	−0.427	0.073	−0.258	0.246
Intra-operator	LV GLS [%]	23	−0.221	0.305	0.125	0.568
Intra-operator	RV TAPSE lateral [mm]	17	0.001	0.998	0.273	0.289

Proportional bias was assessed using ordinary least squares regression of the difference (Δ = value_2 − value_1) on the mean of the pair. Heteroscedasticity was assessed using Spearman correlation between the absolute difference (|Δ|) and the mean of the pair.

**Table 6:** Estimated reduction in MDC95 when averaging two independent traces.

Metric	MDC95 Intra	MDC95 Inter	Estimated MDC95 for the Mean of Two Traces (Intra)	Estimated MDC95 for the Mean of Two Traces (Inter)
LV EDV [mL]	0.696	0.377	0.492	0.267
LV ESV [mL]	0.408	0.464	0.288	0.328
LV SV [mL]	0.462	0.400	0.327	0.283
LV CO [mL/min]	67.500	50.600	47.730	35.780
LV EF [%]	31.000	40.100	21.920	28.355
LV FRAC [%]	23.700	30.500	16.758	21.567
LV GLS [%]	16.800	23.700	11.879	16.758
RV TAPSE lateral [mm]	2450	3760	1732	2659

Estimated MDC95 for the mean of two traces was computed as MDC95/SQRT(2). This approximation assumes that averaging two independent repeat tracings reduces random measurement error by the square root of the number of repeats.

**Table 7:** Exploratory grey zone around selected normative thresholds.

Group	Metric	Threshold	Usable *n*	Flips *n* (%)	Grey-Zone Half-Width	Cases in Grey-Zone *n* (%)
Inter-operator	LV EDV centile (GA)	<5	51	9/51 (17.6%)	30,050	27/51 (52.9%)
Inter-operator	LV EF centile (GA)	<5	51	17/51 (33.3%)	26,440	43/51 (84.3%)
Inter-operator	RV TAPSE lateral centile (GA)	<5	51	16/51 (31.4%)	28,940	40/51 (78.4%)
Inter-operator	LV GLS centile	>95	56	20/56 (35.7%)	40,630	42/56 (75.0%)
Inter-operator	LV GLS Z-score	>2	51	20/51 (39.2%)	3600	40/51 (78.4%)
Intra-operator	LV EDV centile (GA)	<5	28	7/28 (25.0%)	25,870	21/28 (75.0%)
Intra-operator	LV EF centile (GA)	<5	28	5/28 (17.9%)	18,910	25/28 (89.3%)
Intra-operator	RV TAPSE lateral centile (GA)	<5	28	7/28 (25.0%)	25,440	24/28 (85.7%)
Intra-operator	LV GLS centile	>95	30	4/30 (13.3%)	31,900	23/30 (76.7%)
Intra-operator	LV GLS Z-score	>2	24	4/24 (16.7%)	2530	15/24 (62.5%)

Grey-zone was defined as ±(MDC95/2) around the specified threshold in the same scale (centile or Z-score). Cases in grey-zone were counted using the mean of the two available measurements. Grey-zone analysis was restricted to prespecified thresholds selected for clinical interpretability in the main manuscript; full threshold-level results are provided in Supplement S3. For some parameters, normalized outputs remained available even when the raw metric was rejected. The grey zone is an exploratory descriptive construct and should not be interpreted as a validated clinical decision interval or as an individualized probability of misclassification. Prospective external validation would be required before clinical application.

## Data Availability

The dataset supporting this study has been deposited in a public repository and is available at: https://doi.org/10.17605/OSF.IO/54XW7 (accessed on 26 March 2026).

## Supplementary Materials

The following supporting information can be downloaded at: https://www.mdpi.com/article/10.3390/biomedicines14102392/s1, S1: Full parameter atlas (Excel); S2: QC rules and preprocessing (PDF/Word); S3: Threshold-instability atlas (Excel); S4: Additional Bland–Altman plots (PDF); S5: Sensitivity analyses (Excel/PDF); and S6: Fit-for-purpose ranking (Excel). Supporting information is available at: https://doi.org/10.17605/OSF.IO/54XW7 (accessed on 26 March 2026).
