# Supplementary material for: Decision Limits for Fetal Speckle-Tracking Outputs: Minimal Detectable Change and Threshold Instability in FetalHQ
Source: Biomedicines. 2026 Oct 19;14(10):2392. doi: 10.3390/biomedicines14102392 (PMC13702384; doi:10.3390/biomedicines14102392)
Supplement: Supplementary file 1 [file biomedicines-14-02392-s001.zip › biomedicines-4577182-supplementary.pdf]

Combined Supplementary Materials

FetalHQ repeat-analysis study

Contents

S1. Full parameter atlas
S2. Quality-control rules and preprocessing
S3. Threshold-instability atlas
S4. Bland-Altman plots
S5. Sensitivity analyses
S6. Fit-for-purpose ranking

Supplementary File S1

Supplementary File S1. Full parameter atlas (Excel).

Dataset: repeat analyses performed on the same cine-loop; values are paired as *_1 and *_2.

Groups: first 34 rows = Intra-operator repeats; remaining 59 rows = Inter-operator repeats.

4CV GSI outputs were excluded (4CV GSI raw, % and Z-score).

Definitions:

- bias = mean(value_2 − value_1)

- LoA (95%) = bias ± 1.96 × SD(diff)

- Sw = SD(diff) / √2

- MDC95 = 1.96 × SD(diff)

Usable pairs apply QC plausibility filters determined from variable name patterns (see Supplementary File S2).

Supplementary File S1

Atlas: S1: Full parameter atlas (QC-filtered statistics)

parameter	qc_rule	intra_n_total	intra_n_complete	intra_n_usable	intra_usable_rate	intra_bias	intra_sd_diff	intra_sw	intra_loa_low	intra_loa_high	intra_loa_width	intra_mdc95	intra_mean	intra_rel_mdc95	intra_zero_diff_rate	inter_n_total	inter_n_complete	inter_n_usable	inter_usable_rate	inter_bias	inter_sd_diff	inter_sw	inter_loa_low	inter_loa_high	inter_loa_width	inter_mdc95	inter_mean	inter_rel_mdc95	inter_zero_diff_rate	possible_copy_forward_inter
LV_ Global Strain[%]	strain_raw	34	30	23	0.676471	1.684783	8.545042	6.042257	-15.0635	18.433065	33.496565	16.748282	-15.709783	1.066105	0	59	56	50	0.847458	-0.6018	12.066364	8.532208	-24.251874	23.048274	47.300147	23.650074	-16.7509	1.411869	0	FALSE
RV_ Global Strain[%]	strain_raw	34	30	22	0.647059	1.596818	12.689597	8.9729	-23.274791	26.468427	49.743219	24.871609	-11.3675	2.187958	0	59	56	52	0.881356	-2.136731	12.642469	8.939576	-26.91597	22.642508	49.558478	24.779239	-14.724712	1.682834	0	FALSE
LV_ Frac. Area change[%]	percent_0_100	34	30	22	0.647059	-2.209091	12.105958	8.560205	-25.936769	21.518587	47.455357	23.727678	30.712273	0.77258	0	59	56	45	0.762712	1.368667	15.572012	11.011076	-29.152478	31.889811	61.042289	30.521144	35.008111	0.871831	0	FALSE
RV_ Frac. Area change[%]	percent_0_100	34	30	18	0.529412	0.285	15.956293	11.282803	-30.989334	31.559334	62.548668	31.274334	26.156944	1.195642	0	59	56	43	0.728814	3.105581	15.898826	11.242167	-28.056117	34.26728	62.323397	31.161698	30.042093	1.037268	0	FALSE
LV_ EF[%]	percent_0_100	34	30	22	0.647059	-2.438182	15.832293	11.195122	-33.469477	28.593113	62.06259	31.031295	42.266364	0.734184	0	59	56	45	0.762712	2.663333	20.478679	14.480613	-37.474878	42.801544	80.276422	40.138211	47.164111	0.851033	0	FALSE
LV_ SV[ml]	positive	34	30	22	0.647059	0.070909	0.235936	0.166832	-0.391525	0.533344	0.924869	0.462435	0.271818	1.701264	0.1	59	56	45	0.762712	0.026889	0.20407	0.144299	-0.373089	0.426866	0.799955	0.399978	0.298556	1.339709	0.035714	FALSE
LV_ SV/KG[ml/kg]	none	34	30	30	0.882353	0.074	0.488132	0.345162	-0.882739	1.030739	1.913478	0.956739	0.238667	4.008684	0.066667	59	55	55	0.932203	0.024364	0.420136	0.297081	-0.799102	0.847829	1.646932	0.823466	0.423455	1.944638	0.036364	FALSE
LV_ CO[ml/min]	positive	34	30	22	0.647059	11.819545	34.43141	24.346683	-55.666018	79.305109	134.971127	67.485563	35.652045	1.892895	0	59	56	45	0.762712	3.031778	25.798591	18.242359	-47.53346	53.597016	101.130476	50.565238	40.405222	1.251453	0	FALSE
LV_ CO/KG[ml/min/kg]	none	34	30	30	0.882353	8.474333	72.653732	51.373947	-133.926982	150.875648	284.80263	142.401315	30.1135	4.72882	0	59	55	55	0.932203	2.534727	58.156658	41.122968	-111.452323	116.521778	227.974101	113.98705	57.504091	1.982242	0	FALSE
LV_ Frac. Area change.Lt FRAC % (AC)[%]	centile	34	30	30	0.882353	-5.727667	19.582037	13.846591	-44.10846	32.653126	76.761586	38.380793	9.383833	4.090097	0.266667	59	56	56	0.949153	0.879643	26.974039	19.073526	-51.989473	53.748758	105.738231	52.869116	13.16375	4.016266	0.107143	FALSE
LV_ Frac. Area change.Lt FRAC % (AUA)[%]	centile	34	30	30	0.882353	-5.49	18.974729	13.417159	-42.680469	31.700469	74.380937	37.190469	9.088333	4.092111	0.333333	59	54	54	0.915254	0.674444	27.673936	19.568428	-53.566469	54.915358	108.481828	54.240914	12.909815	4.201525	0.166667	FALSE
LV_ Frac. Area change.Lt FRAC % (BPD)[%]	centile	34	30	30	0.882353	-5.717333	19.848228	14.034817	-44.61986	33.185194	77.805054	38.902527	9.352667	4.159512	0.333333	59	55	55	0.932203	1.944545	26.353469	18.634717	-49.708254	53.597345	103.305599	51.6528	12.620455	4.092784	0.163636	FALSE
LV_ Frac. Area change.Lt FRAC % (EFW)[%]	centile	34	30	30	0.882353	-5.617	19.098795	13.504887	-43.050638	31.816638	74.867276	37.433638	8.862833	4.223665	0.433333	59	55	55	0.932203	1.844545	25.937051	18.340265	-48.992075	52.681166	101.673242	50.836621	12.095364	4.202984	0.236364	FALSE
LV_ Frac. Area change.Lt FRAC % (FL)[%]	centile	34	29	29	0.852941	-2.428276	11.740528	8.301807	-25.43971	20.583158	46.022868	23.011434	7.921724	2.904852	0.37931	59	55	55	0.932203	1.093818	27.419778	19.388711	-52.648946	54.836582	107.485528	53.742764	13.077273	4.109631	0.163636	FALSE
LV_ Frac. Area change.Lt FRAC % (GA)[%]	centile	34	28	28	0.823529	-5.686429	21.561681	15.246411	-47.947323	36.574466	84.521788	42.260894	10.202143	4.142355	0.25	59	51	51	0.864407	0.113529	28.144181	19.900942	-55.049066	55.276125	110.325191	55.162596	14.29049	3.860091	0.156863	FALSE
LV_ Frac. Area change.Lt FRAC % (HC)[%]	centile	34	30	30	0.882353	-5.717667	19.580801	13.845717	-44.096036	32.660702	76.756738	38.378369	9.290167	4.131074	0.3	59	55	55	0.932203	1.929636	26.23569	18.551435	-49.492317	53.351589	102.843906	51.421953	12.607545	4.078665	0.163636	FALSE
RV_ Frac. Area change.Rt FRAC % (AC)[%]	centile	34	30	30	0.882353	5.394	31.081617	21.978022	-55.525969	66.313969	121.839937	60.919969	6.743667	9.033657	0.466667	59	56	56	0.949153	6.553571	30.153609	21.321821	-52.547502	65.654644	118.202146	59.101073	11.851607	4.986756	0.214286	FALSE
RV_ Frac. Area change.Rt FRAC % (AUA)[%]	centile	34	30	30	0.882353	5.436667	30.609094	21.643898	-54.557158	65.430492	119.98765	59.993825	6.486667	9.248791	0.5	59	54	54	0.915254	6.911296	30.791711	21.773028	-53.440458	67.26305	120.703508	60.351754	11.93287	5.057606	0.259259	FALSE
RV_ Frac. Area change.Rt FRAC % (BPD)[%]	centile	34	30	30	0.882353	5.301333	30.996572	21.917886	-55.451948	66.054615	121.506563	60.753281	6.646667	9.140413	0.5	59	55	55	0.932203	6.941091	31.150845	22.026974	-54.114565	67.996747	122.111313	61.055656	12.156364	5.022526	0.254545	FALSE
RV_ Frac. Area change.Rt FRAC % (EFW)[%]	centile	34	30	30	0.882353	5.235	30.368554	21.47381	-54.287366	64.757366	119.044731	59.522366	6.501833	9.154705	0.466667	59	55	55	0.932203	6.678182	29.963417	21.187336	-52.050116	65.40648	117.456596	58.728298	11.757818	4.99483	0.236364	FALSE
RV_ Frac. Area change.Rt FRAC % (FL)[%]	centile	34	29	29	0.852941	5.550345	30.670839	21.687558	-54.5645	65.66519	120.22969	60.114845	6.621379	9.078901	0.482759	59	55	55	0.932203	6.620727	30.274446	21.407266	-52.717188	65.958642	118.67583	59.337915	11.712364	5.066263	0.290909	FALSE
RV_ Frac. Area change.Rt FRAC % (GA)[%]	centile	34	28	28	0.823529	6.515	33.290491	23.539932	-58.734363	71.764363	130.498726	65.249363	7.696071	8.478269	0.357143	59	51	51	0.864407	6.308235	31.865858	22.532565	-56.148847	68.765318	124.914165	62.457082	13.486471	4.631092	0.215686	FALSE
RV_ Frac. Area change.Rt FRAC % (HC)[%]	centile	34	30	30	0.882353	5.208333	30.472375	21.547223	-54.517522	64.934188	119.45171	59.725855	6.498167	9.191185	0.5	59	55	55	0.932203	6.711636	31.247808	22.095537	-54.534067	67.95734	122.491407	61.245704	11.923818	5.136417	0.236364	FALSE
LV_ EF.Lt EF % (AC)[%]	centile	34	19	19	0.558824	-4.552105	12.636727	8.935515	-29.32009	20.215879	49.535969	24.767984	6.384474	3.879409	0.368421	59	28	28	0.474576	-3.156429	28.368726	20.059719	-58.759132	52.446275	111.205407	55.602704	14.407857	3.859193	0.178571	FALSE
LV_ EF.Lt EF % (AUA)[%]	centile	34	30	30	0.882353	-5.102667	16.411541	11.604712	-37.269286	27.063953	64.333239	32.166619	7.295333	4.409205	0.4	59	54	54	0.915254	0.943889	25.993799	18.380392	-50.003958	51.891735	101.895693	50.947847	11.680833	4.361662	0.185185	FALSE
LV_ EF.Lt EF % (BPD)[%]	centile	34	30	30	0.882353	-5.397667	17.438743	12.331053	-39.577603	28.78227	68.359872	34.179936	7.4945	4.560669	0.466667	59	51	51	0.864407	3.072549	26.328841	18.617302	-48.53198	54.677078	103.209058	51.604529	11.582157	4.45552	0.235294	FALSE
LV_ EF.Lt EF % (EFW)[%]	centile	34	16	16	0.470588	-4.1625	12.491519	8.832838	-28.645877	20.320877	48.966754	24.483377	6.60125	3.7089	0.4375	59	19	19	0.322034	-0.09	32.180315	22.754919	-63.163417	62.983417	126.146833	63.073417	16.734474	3.769071	0.210526	FALSE
LV_ EF.Lt EF % (FL)[%]	centile	34	17	17	0.5	-1.348235	9.398487	6.645734	-19.769269	17.072799	36.842068	18.421034	7.960588	2.314029	0.235294	59	33	33	0.559322	0.64	30.74369	21.739072	-59.617632	60.897632	120.515264	60.257632	15.441515	3.902313	0.181818	FALSE
LV_ EF.Lt EF % (GA)[%]	centile	34	28	28	0.823529	-5.836429	19.296058	13.644373	-43.656701	31.983844	75.640546	37.820273	8.511786	4.443283	0.321429	59	51	51	0.864407	1.451569	26.975524	19.074576	-51.420459	54.323596	105.744056	52.872028	13.757745	3.843074	0.156863	FALSE
LV_ EF.Lt EF % (HC)[%]	centile	34	13	13	0.382353	-4.966923	12.460226	8.81071	-29.388965	19.455119	48.844085	24.422042	10.755	2.270762	0.384615	59	14	14	0.237288	7.119286	26.651082	18.84516	-45.116834	59.355405	104.47224	52.23612	13.593214	3.842809	0.285714	FALSE
LV_ SV.Lt SV % (AC)[%]	centile	34	19	19	0.558824	4.52	19.872849	14.052226	-34.430784	43.470784	77.901568	38.950784	12.030526	3.237662	0.157895	59	28	28	0.474576	-3.996786	28.714626	20.304307	-60.277452	52.283881	112.561333	56.280667	20.579107	2.734845	0.071429	FALSE
LV_ SV.Lt SV % (AUA)[%]	centile	34	30	30	0.882353	2.920333	15.708701	11.107729	-27.86872	33.709386	61.578106	30.789053	5.9535	5.171589	0.466667	59	54	54	0.915254	0.893148	32.176203	22.752012	-62.17221	63.958507	126.130717	63.065358	13.601574	4.636622	0.351852	FALSE
LV_ SV.Lt SV % (BPD)[%]	centile	34	30	30	0.882353	-0.139	23.20094	16.405542	-45.612841	45.334841	90.947683	45.473841	13.234833	3.435921	0.566667	59	51	51	0.864407	-0.520392	24.722849	17.481694	-48.977176	47.936391	96.913567	48.456783	19.207255	2.522838	0.607843	FALSE
LV_ SV.Lt SV % (EFW)[%]	centile	34	16	16	0.470588	6.15125	18.822681	13.309646	-30.741205	43.043705	73.784911	36.892455	12.700625	2.904775	0.0625	59	19	19	0.322034	-2.717895	27.858822	19.699162	-57.321186	51.885397	109.206583	54.603291	24.167368	2.259381	0.105263	FALSE
LV_ SV.Lt SV % (FL)[%]	centile	34	17	17	0.5	6.661765	15.675518	11.084265	-24.06225	37.385779	61.448029	30.724015	11.645588	2.638254	0.058824	59	33	33	0.559322	0.384545	30.529332	21.587498	-59.452945	60.222036	119.674981	59.837491	20.773788	2.880432	0.090909	FALSE
LV_ SV.Lt SV % (GA)[%]	centile	34	28	28	0.823529	3.007857	16.525087	11.685001	-29.381312	35.397027	64.778339	32.38917	7.9075	4.096006	0.178571	59	51	51	0.864407	2.832157	34.878387	24.662744	-65.529481	71.193795	136.723277	68.361638	16.926078	4.038835	0.098039	FALSE
LV_ SV.Lt SV % (HC)[%]	centile	34	13	13	0.382353	2.873077	28.651406	20.259604	-53.28368	59.029834	112.313513	56.156757	12.991154	4.322692	0.230769	59	14	14	0.237288	-4.757143	28.998756	20.505217	-61.594704	52.080418	113.675122	56.837561	24.911429	2.281586	0.142857	FALSE
LV_ SV/KG.Lt SV/KG % (AC)[%]	centile	34	19	19	0.558824	6.178947	18.575729	13.135024	-30.229482	42.587376	72.816858	36.408429	8.936316	4.07421	0.105263	59	27	27	0.457627	-2.17037	26.884313	19.01008	-54.863623	50.522883	105.386506	52.693253	19.695926	2.675338	0.074074	FALSE
LV_ SV/KG.Lt SV/KG % (AUA)[%]	centile	34	30	30	0.882353	0.655667	20.500789	14.496247	-39.52588	40.837213	80.363094	40.181547	9.547167	4.20874	0.1	59	53	53	0.898305	0.302453	34.590647	24.459281	-67.495215	68.100121	135.595336	67.797668	20.321792	3.336205	0.09434	FALSE
LV_ SV/KG.Lt SV/KG % (BPD)[%]	centile	34	30	30	0.882353	0.661667	20.362415	14.398402	-39.248666	40.571999	79.820666	39.910333	9.265833	4.307258	0.1	59	51	51	0.864407	2.916471	37.880402	26.785489	-71.329118	77.162059	148.491177	74.245589	20.873137	3.556992	0.098039	FALSE
LV_ SV/KG.Lt SV/KG % (EFW)[%]	centile	34	16	16	0.470588	6.975	19.845381	14.032803	-31.921947	45.871947	77.793893	38.896947	10.63	3.659167	0.0625	59	19	19	0.322034	-1.533684	30.705127	21.711804	-61.715734	58.648365	120.364099	60.18205	23.143684	2.600366	0.105263	FALSE
LV_ SV/KG.Lt SV/KG % (FL)[%]	centile	34	17	17	0.5	8.209412	19.680499	13.916214	-30.364366	46.783189	77.147555	38.573778	10.861765	3.551336	0.058824	59	32	32	0.542373	1.92	31.410107	22.2103	-59.64381	63.48381	123.12762	61.56381	21.16375	2.908927	0.09375	FALSE
LV_ SV/KG.Lt SV/KG % (GA)[%]	centile	34	28	28	0.823529	0.202143	21.355045	15.100297	-41.653746	42.058032	83.711777	41.855889	10.4475	4.006307	0.071429	59	50	50	0.847458	3.4464	36.684012	25.939513	-68.454263	75.347063	143.801326	71.900663	22.2106	3.237223	0.1	FALSE
LV_ SV/KG.Lt SV/KG % (HC)[%]	centile	34	13	13	0.382353	5.799231	27.621553	19.531387	-48.339013	59.937474	108.276487	54.138243	15.348846	3.527187	0	59	14	14	0.237288	-3.821429	23.271766	16.455624	-49.43409	41.791233	91.225324	45.612662	21.701429	2.101828	0.071429	FALSE
LV_ CO.Lt CO % (AC)[%]	centile	34	19	19	0.558824	5.166316	18.891242	13.358126	-31.860519	42.193151	74.05367	37.026835	9.516842	3.890664	0	59	28	28	0.474576	-5.687857	25.365132	17.935857	-55.403516	44.027802	99.431317	49.715659	17.318214	2.870715	0.071429	FALSE
LV_ CO.Lt CO % (AUA)[%]	centile	34	30	30	0.882353	2.866	14.752048	10.431273	-26.048014	31.780014	57.828028	28.914014	4.485333	6.446347	0.466667	59	54	54	0.915254	0.323333	26.221567	18.541448	-51.070939	51.717606	102.788544	51.394272	9.492778	5.414039	0.37037	FALSE
LV_ CO.Lt CO % (BPD)[%]	centile	34	30	30	0.882353	1.889667	16.286877	11.516561	-30.032612	33.811945	63.844557	31.922278	5.7165	5.584235	0.266667	59	51	51	0.864407	3.606275	32.090883	22.691681	-59.291856	66.504405	125.796261	62.89813	14.079804	4.467259	0.215686	FALSE
LV_ CO.Lt CO % (EFW)[%]	centile	34	16	16	0.470588	6.76625	18.245946	12.901832	-28.995804	42.528304	71.524108	35.762054	9.671875	3.697531	0	59	19	19	0.322034	-4.796842	26.668279	18.857321	-57.066668	47.472984	104.539652	52.269826	21.078947	2.479717	0.105263	FALSE
LV_ CO.Lt CO % (FL)[%]	centile	34	17	17	0.5	7.017647	15.724601	11.118972	-23.80257	37.837865	61.640435	30.820217	8.712941	3.537292	0	59	33	33	0.559322	-1.653636	27.538013	19.472315	-55.628141	52.320868	107.949009	53.974505	16.629848	3.24564	0.090909	FALSE
LV_ CO.Lt CO % (GA)[%]	centile	34	28	28	0.823529	3.506071	16.035046	11.33849	-27.92262	34.934762	62.857382	31.428691	5.391964	5.828802	0.25	59	51	51	0.864407	2.158235	31.265423	22.107993	-59.121994	63.438464	122.560458	61.280229	13.111667	4.673718	0.098039	FALSE
LV_ CO.Lt CO % (HC)[%]	centile	34	13	13	0.382353	3.84	22.588752	15.97266	-40.433955	48.113955	88.547909	44.273955	9.934615	4.456534	0	59	14	14	0.237288	-2.327143	19.473468	13.769821	-40.49514	35.840854	76.335993	38.167997	20.945714	1.822234	0.071429	FALSE
LV_ CO/KG.Lt CO/KG % (AC)[%]	centile	34	19	19	0.558824	6.57	17.817403	12.598807	-28.35211	41.49211	69.84422	34.92211	6.638684	5.260396	0	59	27	27	0.457627	-3.624074	25.46718	18.008016	-53.539747	46.291599	99.831346	49.915673	15.893519	3.140631	0.074074	FALSE
LV_ CO/KG.Lt CO/KG % (AUA)[%]	centile	34	30	30	0.882353	2.135667	18.189513	12.861928	-33.515779	37.787112	71.302891	35.651446	6.479167	5.502474	0.033333	59	53	53	0.898305	0.230943	32.493048	22.976055	-63.455431	63.917318	127.372749	63.686374	16.575849	3.842118	0.075472	FALSE
LV_ CO/KG.Lt CO/KG % (BPD)[%]	centile	34	30	30	0.882353	2.165	18.151932	12.835354	-33.412787	37.742787	71.155575	35.577787	6.273833	5.670821	0.033333	59	51	51	0.864407	1.903922	35.756306	25.283527	-68.178439	71.986282	140.164721	70.08236	17.177059	4.079998	0.078431	FALSE
LV_ CO/KG.Lt CO/KG % (EFW)[%]	centile	34	16	16	0.470588	7.57375	19.142929	13.536095	-29.946392	45.093892	75.040283	37.520142	7.87125	4.766732	0	59	19	19	0.322034	-3.713158	29.932988	21.165819	-62.381814	54.955498	117.337313	58.668656	19.777632	2.966415	0.105263	FALSE
LV_ CO/KG.Lt CO/KG % (FL)[%]	centile	34	17	17	0.5	8.27	18.878732	13.349279	-28.732315	45.272315	74.00463	37.002315	8.045	4.599418	0	59	32	32	0.542373	0.031562	29.645505	20.962538	-58.073627	58.136752	116.210379	58.10519	17.153281	3.38741	0.09375	FALSE
LV_ CO/KG.Lt CO/KG % (GA)[%]	centile	34	28	28	0.823529	1.550357	18.950801	13.40024	-35.593213	38.693927	74.28714	37.14357	7.235893	5.133239	0	59	50	50	0.847458	2.4102	35.008604	24.754821	-66.206663	71.027063	137.233727	68.616863	18.6395	3.681261	0.08	FALSE
LV_ CO/KG.Lt CO/KG % (HC)[%]	centile	34	13	13	0.382353	5.301538	27.473458	19.426668	-48.546439	59.149516	107.695956	53.847978	11.944615	4.508138	0	59	14	14	0.237288	-2.06	21.878524	15.470453	-44.941907	40.821907	85.763814	42.881907	19.537857	2.194811	0.071429	FALSE
LV_ Frac. Area change.Lt FRAC ZS (AC)[]	none	34	30	30	0.882353	2.100333	11.724793	8.290681	-20.880261	25.080927	45.961188	22.980594	-5.1735	4.441982	0.033333	59	56	56	0.949153	-0.001964	3.720598	2.63086	-7.294336	7.290408	14.584744	7.292372	-3.519196	2.07217	0	FALSE
LV_ Frac. Area change.Lt FRAC ZS(AUA)[]	none	34	30	30	0.882353	2.194667	12.134028	8.580053	-21.588027	25.977361	47.565388	23.782694	-5.344333	4.450077	0	59	54	54	0.915254	-0.033704	3.865168	2.733087	-7.609433	7.542026	15.15146	7.57573	-3.672778	2.06267	0	FALSE
LV_ Frac. Area change.Lt FRAC ZS(BPD)[]	none	34	30	30	0.882353	2.177	12.070698	8.535272	-21.481567	25.835567	47.317134	23.658567	-5.340167	4.430305	0	59	55	55	0.932203	0.229455	3.47791	2.459254	-6.587249	7.046158	13.633407	6.816703	-3.603818	1.891523	0	FALSE
LV_ Frac. Area change.Lt FRAC ZS (EFW)[]	none	34	30	30	0.882353	2.336	12.89116	9.115427	-22.930673	27.602673	50.533347	25.266673	-5.719667	4.417508	0	59	55	55	0.932203	0.261818	3.685496	2.606039	-6.961754	7.485391	14.447145	7.223572	-3.887273	1.858262	0	FALSE
LV_ Frac. Area change.Lt FRAC ZS(FL)[]	none	34	29	29	0.852941	2.463448	12.493311	8.834105	-22.023442	26.950338	48.97378	24.48689	-5.538966	4.420842	0	59	55	55	0.932203	0.055091	3.875727	2.740553	-7.541335	7.651517	15.192851	7.596426	-3.719727	2.0422	0	FALSE
LV_ Frac. Area change.Lt FRAC ZS(GA)[]	none	34	28	28	0.823529	1.885357	12.61147	8.917656	-22.833123	26.603838	49.436961	24.71848	-5.096964	4.849648	0	59	51	51	0.864407	0.127843	3.78231	2.674497	-7.285485	7.541171	14.826655	7.413328	-3.607451	2.055004	0	FALSE
LV_ Frac. Area change.Lt FRAC ZS(HC)[]	none	34	30	30	0.882353	2.193333	12.111731	8.564287	-21.54566	25.932326	47.477986	23.738993	-5.321333	4.461099	0	59	55	55	0.932203	0.231636	3.439311	2.43196	-6.509413	6.972685	13.482098	6.741049	-3.591455	1.876969	0	FALSE
RV_ Frac. Area change.Rt FRAC ZS (AC)[]	none	34	30	30	0.882353	1.687667	6.565365	4.642414	-11.180448	14.555781	25.736229	12.868115	-6.2925	2.044992	0	59	56	56	0.949153	0.506071	5.202276	3.678565	-9.69039	10.702533	20.392923	10.196462	-4.784107	2.13132	0	FALSE
RV_ Frac. Area change.Rt FRAC ZS(AUA)[]	none	34	30	30	0.882353	1.750667	6.695643	4.734535	-11.372793	14.874127	26.24692	13.12346	-6.46	2.031495	0	59	54	54	0.915254	0.661111	5.341831	3.777245	-9.808877	11.1311	20.939977	10.469989	-4.960556	2.110648	0	FALSE
RV_ Frac. Area change.Rt FRAC ZS(BPD)[]	none	34	30	30	0.882353	1.761667	6.698787	4.736758	-11.367956	14.891289	26.259244	13.129622	-6.4145	2.046866	0	59	55	55	0.932203	0.650727	5.258076	3.718021	-9.655101	10.956555	20.611656	10.305828	-4.873909	2.114489	0	FALSE
RV_ Frac. Area change.Rt FRAC ZS (EFW)[]	none	34	30	30	0.882353	1.681667	6.506645	4.600893	-11.071357	14.434691	25.506048	12.753024	-6.289833	2.027562	0	59	55	55	0.932203	0.643818	5.102697	3.608151	-9.357467	10.645104	20.002571	10.001285	-4.754455	2.103561	0	FALSE
RV_ Frac. Area change.Rt FRAC ZS(FL)[]	none	34	29	29	0.852941	1.884828	6.863644	4.853329	-11.567915	15.33757	26.905485	13.452743	-6.514828	2.064942	0	59	55	55	0.932203	0.449455	5.40272	3.8203	-10.139876	11.038785	21.178661	10.58933	-4.973636	2.129092	0	FALSE
RV_ Frac. Area change.Rt FRAC ZS(GA)[]	none	34	28	28	0.823529	0.895357	5.873946	4.153507	-10.617577	12.408291	23.025868	11.512934	-5.69625	2.021143	0	59	51	51	0.864407	0.821373	4.683059	3.311423	-8.357423	10.000168	18.357591	9.178795	-4.421275	2.076052	0	FALSE
RV_ Frac. Area change.Rt FRAC ZS(HC)[]	none	34	30	30	0.882353	1.761333	6.728204	4.757559	-11.425947	14.948613	26.37456	13.18728	-6.446667	2.045597	0	59	55	55	0.932203	0.650364	5.293105	3.742791	-9.724123	11.02485	20.748972	10.374486	-4.924818	2.106572	0	FALSE
LV_ EF.Lt EF ZS (AC)[]	none	34	19	19	0.558824	-1.077895	5.863923	4.14642	-12.571184	10.415395	22.986579	11.493289	-4.893684	2.348596	0	59	28	28	0.474576	-1.051786	5.766422	4.077476	-12.353973	10.250401	22.604374	11.302187	-4.805893	2.351735	0	FALSE
LV_ EF.Lt EF ZS(AUA)[]	none	34	30	30	0.882353	5.871	31.7518	22.451913	-56.362527	68.104527	124.467054	62.233527	-8.098833	7.684258	0	59	54	54	0.915254	0.100556	5.191019	3.670605	-10.073841	10.274953	20.348794	10.174397	-4.566944	2.227835	0	FALSE
LV_ EF.Lt EF ZS(BPD)[]	none	34	30	30	0.882353	6.143333	33.217893	23.488598	-58.963738	71.250404	130.214142	65.107071	-8.426333	7.726619	0	59	51	51	0.864407	0.555686	4.948477	3.499102	-9.14333	10.254702	19.398032	9.699016	-4.758039	2.038448	0	FALSE
LV_ EF.Lt EF ZS (EFW)[]	none	34	16	16	0.470588	-1.230625	6.573856	4.648418	-14.115382	11.654132	25.769515	12.884757	-5.125937	2.513639	0	59	19	19	0.322034	-0.268421	4.916158	3.476249	-9.904092	9.36725	19.271341	9.635671	-5.24	1.838868	0	FALSE
LV_ EF.Lt EF ZS(FL)[]	none	34	17	17	0.5	9.164118	41.856586	29.597076	-72.874791	91.203026	164.077817	82.038908	-10.246176	8.006783	0	59	33	33	0.559322	-0.17	5.67688	4.01416	-11.296684	10.956684	22.253368	11.126684	-4.760758	2.337167	0	FALSE
LV_ EF.Lt EF ZS(GA)[]	none	34	28	28	0.823529	5.362857	32.441632	22.939698	-58.222741	68.948455	127.171196	63.585598	-7.689643	8.268992	0	59	51	51	0.864407	0.326667	4.999831	3.535414	-9.473002	10.126336	19.599338	9.799669	-4.34098	2.257478	0.019608	FALSE
LV_ EF.Lt EF ZS(HC)[]	none	34	13	13	0.382353	0.506923	3.435834	2.429502	-6.227312	7.241158	13.46847	6.734235	-4.141923	1.625872	0	59	14	14	0.237288	0.887143	4.706453	3.327965	-8.337505	10.111791	18.449295	9.224648	-4.862857	1.89696	0	FALSE
LV_ SV.Lt SV ZS (AC)[]	none	34	19	19	0.558824	-0.079474	1.694453	1.198159	-3.400602	3.241655	6.642257	3.321128	-2.238158	1.483867	0.105263	59	28	28	0.474576	-0.45	2.352148	1.66322	-5.06021	4.16021	9.22042	4.61021	-1.855	2.485288	0	FALSE
LV_ SV.Lt SV ZS(AUA)[]	none	34	30	30	0.882353	0.901	5.194971	3.673399	-9.281142	11.083142	20.364285	10.182142	-5.926833	1.717973	0.066667	59	54	54	0.915254	-0.140556	4.834072	3.418205	-9.615336	9.334225	18.949562	9.474781	-5.225278	1.813259	0.037037	FALSE
LV_ SV.Lt SV ZS(BPD)[]	none	34	30	30	0.882353	-6.924667	58.388874	41.287169	-121.366859	107.517526	228.884385	114.442193	6.512667	17.572248	0.066667	59	51	51	0.864407	3.42098	10.340119	7.311569	-16.845654	23.687614	40.533268	20.266634	-3.277353	6.183842	0.039216	FALSE
LV_ SV.Lt SV ZS (EFW)[]	none	34	16	16	0.470588	-0.019375	1.575256	1.113875	-3.106878	3.068128	6.175005	3.087503	-1.815938	1.700225	0.0625	59	19	19	0.322034	0.070526	1.647235	1.164771	-3.158054	3.299107	6.457161	3.228581	-1.355789	2.381329	0	FALSE
LV_ SV.Lt SV ZS(FL)[]	none	34	17	17	0.5	0.53	2.526643	1.786606	-4.42222	5.48222	9.904441	4.95222	-2.319118	2.13539	0.058824	59	33	33	0.559322	-0.108182	2.306046	1.630621	-4.628031	4.411668	9.039699	4.519849	-1.669545	2.707234	0.030303	FALSE
LV_ SV.Lt SV ZS(GA)[]	none	34	28	28	0.823529	0.096071	2.535454	1.792837	-4.873419	5.065562	9.938981	4.96949	-3.07625	1.615438	0.071429	59	51	51	0.864407	0.004314	2.62673	1.857378	-5.144077	5.152704	10.296781	5.14839	-2.315882	2.223079	0.039216	FALSE
LV_ SV.Lt SV ZS(HC)[]	none	34	13	13	0.382353	0.068462	2.491883	1.762027	-4.815629	4.952552	9.768181	4.884091	-2.860385	1.707494	0	59	14	14	0.237288	-0.71	4.898744	3.463935	-10.311538	8.891538	19.203076	9.601538	-2.435	3.943137	0	FALSE
LV_ SV/KG.Lt SV/KG ZS (AC)[]	none	34	19	19	0.558824	-0.011053	1.634135	1.155508	-3.213958	3.191853	6.405811	3.202906	-2.267632	1.412445	0.105263	59	27	27	0.457627	-0.136667	2.049989	1.449561	-4.154645	3.881311	8.035956	4.017978	-1.669074	2.40731	0	FALSE
LV_ SV/KG.Lt SV/KG ZS(AUA)[]	none	34	30	30	0.882353	0.380333	2.525997	1.786149	-4.57062	5.331287	9.901906	4.950953	-2.492833	1.986075	0.066667	59	53	53	0.898305	0.03283	2.075786	1.467802	-4.03571	4.10137	8.13708	4.06854	-1.598302	2.545539	0.037736	FALSE
LV_ SV/KG.Lt SV/KG ZS(BPD)[]	none	34	30	30	0.882353	0.394667	2.617535	1.850877	-4.735701	5.525035	10.260736	5.130368	-2.580333	1.988258	0.066667	59	51	51	0.864407	0.166667	2.339129	1.654014	-4.418027	4.75136	9.169388	4.584694	-1.620392	2.829373	0.039216	FALSE
LV_ SV/KG.Lt SV/KG ZS (EFW)[]	none	34	16	16	0.470588	-0.050625	1.773807	1.254271	-3.527288	3.426038	6.953325	3.476663	-2.177812	1.596401	0.0625	59	19	19	0.322034	0.048947	1.999147	1.41361	-3.86938	3.967275	7.836655	3.918328	-1.609737	2.434142	0	FALSE
LV_ SV/KG.Lt SV/KG ZS(FL)[]	none	34	17	17	0.5	0.438824	2.726394	1.927852	-4.904908	5.782556	10.687464	5.343732	-2.51	2.128977	0.058824	59	32	32	0.542373	0.098125	2.043472	1.444953	-3.90708	4.10333	8.010409	4.005205	-1.58125	2.532936	0.03125	FALSE

parameter	qc_rule	intra_n_total	intra_n_complete	intra_n_usable	intra_usable_rate	intra_bias	intra_sd_diff	intra_sw	intra_loa_low	intra_loa_high	intra_loa_width	intra_mdc95	intra_mean	intra_rel_mdc95	intra_zero_diff_rate	inter_n_total	inter_n_complete	inter_n_usable	inter_usable_rate	inter_bias	inter_sd_diff	inter_sw	inter_loa_low	inter_loa_high	inter_loa_width	inter_mdc95	inter_mean	inter_rel_mdc95	inter_zero_diff_rate	possible_copy_forward_inter
LV_ SV/KG.Lt SV/KG ZS(GA)[]	none	34	28	28	0.823529	0.101786	2.274005	1.607964	-4.355263	4.558835	8.914098	4.457049	-2.304821	1.933794	0.071429	59	50	50	0.847458	0.2658	2.104855	1.488357	-3.859715	4.391315	8.25103	4.125515	-1.4493	2.846557	0.04	FALSE
LV_ SV/KG.Lt SV/KG ZS(HC)[]	none	34	13	13	0.382353	0.216923	1.565926	1.107277	-2.852291	3.286137	6.138428	3.069214	-1.798462	1.706578	0	59	14	14	0.237288	0.068571	2.115739	1.496053	-4.078277	4.21542	8.293697	4.146849	-1.522857	2.723071	0	FALSE
LV_ CO.Lt CO ZS (AC)[]	none	34	19	19	0.558824	-0.147368	2.021789	1.429621	-4.110075	3.815338	7.925414	3.962707	-2.380526	1.664635	0	59	28	28	0.474576	-0.534286	2.106601	1.489592	-4.663223	3.594651	8.257874	4.128937	-1.946071	2.121678	0	FALSE
LV_ CO.Lt CO ZS(AUA)[]	none	34	30	30	0.882353	0.847	4.188119	2.961447	-7.361713	9.055713	16.417426	8.208713	-5.720167	1.435048	0	59	54	54	0.915254	-0.151296	4.008629	2.834529	-8.00821	7.705617	15.713827	7.856914	-5.167315	1.520502	0	FALSE
LV_ CO.Lt CO ZS(BPD)[]	none	34	30	30	0.882353	1.166667	4.482239	3.169421	-7.618521	9.951855	17.570376	8.785188	-4.784	1.836369	0	59	51	51	0.864407	0.102745	3.359371	2.375434	-6.481622	6.687112	13.168734	6.584367	-3.669804	1.794201	0	FALSE
LV_ CO.Lt CO ZS (EFW)[]	none	34	16	16	0.470588	-0.15375	2.258223	1.596805	-4.579867	4.272367	8.852235	4.426117	-2.121875	2.085946	0	59	19	19	0.322034	-0.053684	1.593006	1.126426	-3.175977	3.068608	6.244585	3.122292	-1.538947	2.028849	0	FALSE
LV_ CO.Lt CO ZS(FL)[]	none	34	17	17	0.5	0.363529	2.700808	1.909759	-4.930054	5.657112	10.587166	5.293583	-2.533529	2.08941	0	59	33	33	0.559322	-0.277879	2.115774	1.496078	-4.424795	3.869038	8.293833	4.146917	-1.87197	2.215269	0	FALSE
LV_ CO.Lt CO ZS(GA)[]	none	34	28	28	0.823529	0.097857	2.71249	1.91802	-5.218623	5.414338	10.632961	5.316481	-3.623214	1.467338	0	59	51	51	0.864407	-0.08098	2.634334	1.862755	-5.244274	5.082313	10.326587	5.163294	-2.86402	1.802814	0	FALSE
LV_ CO.Lt CO ZS(HC)[]	none	34	13	13	0.382353	0.015385	2.376112	1.680165	-4.641796	4.672565	9.314361	4.65718	-2.355385	1.977248	0	59	14	14	0.237288	-0.043571	2.50328	1.770086	-4.95	4.862857	9.812856	4.906428	-1.778214	2.759188	0	FALSE
LV_ CO/KG.Lt CO/KG ZS (AC)[]	none	34	19	19	0.558824	-0.123684	2.169706	1.534214	-4.376308	4.12894	8.505248	4.252624	-2.580263	1.648136	0	59	27	27	0.457627	-0.263333	1.938584	1.370786	-4.062958	3.536291	7.599249	3.799625	-1.897963	2.001949	0	FALSE
LV_ CO/KG.Lt CO/KG ZS(AUA)[]	none	34	30	30	0.882353	0.302667	2.586165	1.828695	-4.766216	5.371549	10.137766	5.068883	-2.737333	1.851759	0	59	53	53	0.898305	-0.002075	1.953569	1.381382	-3.831071	3.82692	7.657991	3.828995	-1.843679	2.076823	0	FALSE
LV_ CO/KG.Lt CO/KG ZS(BPD)[]	none	34	30	30	0.882353	0.316	2.644721	1.8701	-4.867653	5.499653	10.367306	5.183653	-2.819333	1.83861	0	59	51	51	0.864407	0.092549	2.189014	1.547867	-4.197919	4.383017	8.580936	4.290468	-1.87451	2.288848	0	FALSE
LV_ CO/KG.Lt CO/KG ZS (EFW)[]	none	34	16	16	0.470588	-0.161875	2.357581	1.667062	-4.782734	4.458984	9.241719	4.620859	-2.529062	1.827104	0	59	19	19	0.322034	-0.116316	1.955599	1.382818	-3.949291	3.716659	7.66595	3.832975	-1.836053	2.087617	0	FALSE
LV_ CO/KG.Lt CO/KG ZS(FL)[]	none	34	17	17	0.5	0.264706	3.026971	2.140391	-5.668157	6.197568	11.865725	5.932863	-2.767647	2.143649	0	59	32	32	0.542373	-0.069687	1.916656	1.355281	-3.826334	3.686959	7.513292	3.756646	-1.802969	2.083589	0	FALSE
LV_ CO/KG.Lt CO/KG ZS(GA)[]	none	34	28	28	0.823529	0.033929	2.380923	1.683567	-4.632681	4.700538	9.333219	4.666609	-2.536964	1.839446	0	59	50	50	0.847458	0.1926	1.986359	1.404568	-3.700664	4.085864	7.786529	3.893264	-1.6581	2.348028	0	FALSE
LV_ CO/KG.Lt CO/KG ZS(HC)[]	none	34	13	13	0.382353	0.02	2.398659	1.696108	-4.681372	4.721372	9.402745	4.701372	-2.140769	2.196114	0	59	14	14	0.237288	0.098571	2.080998	1.471488	-3.980184	4.177327	8.157511	4.078756	-1.627857	2.505598	0	FALSE
LV_ ED Area[cm¸]	none	34	30	30	0.882353	0.010667	0.274904	0.194386	-0.528144	0.549478	1.077622	0.538811	0.914333	0.589294	0.033333	59	56	56	0.949153	-0.041429	0.202452	0.143156	-0.438235	0.355378	0.793614	0.396807	0.927857	0.427659	0	FALSE
LV_ ED Length[cm]	positive	34	30	30	0.882353	-0.020333	0.271858	0.192233	-0.553175	0.512508	1.065683	0.532842	1.3085	0.407216	0	59	56	56	0.949153	-0.066964	0.218972	0.154837	-0.49615	0.362221	0.858371	0.429185	1.351518	0.317558	0.035714	FALSE
LV_ ES Area[cm¸]	none	34	30	30	0.882353	-0.030333	0.192488	0.13611	-0.40761	0.346943	0.754553	0.377276	0.719833	0.524116	0	59	56	56	0.949153	-0.0475	0.246291	0.174154	-0.53023	0.43523	0.965459	0.48273	0.66875	0.721839	0.071429	FALSE
LV_ ES Length[cm]	positive	34	30	30	0.882353	-0.028667	0.197392	0.139577	-0.415555	0.358222	0.773777	0.386889	1.161	0.333237	0.033333	59	56	56	0.949153	-0.046429	0.223505	0.158042	-0.484497	0.39164	0.876138	0.438069	1.161786	0.377065	0.071429	FALSE
LV_ EDV[ml]	positive	34	30	30	0.882353	0.031667	0.355082	0.251081	-0.664294	0.727628	1.391922	0.695961	0.618167	1.125847	0	59	56	56	0.949153	-0.023929	0.192256	0.135946	-0.400751	0.352894	0.753645	0.376822	0.611964	0.615759	0.017857	FALSE
LV_ ESV[ml]	positive	34	30	30	0.882353	-0.025333	0.207941	0.147037	-0.432898	0.382231	0.815129	0.407565	0.438667	0.929099	0.033333	59	56	56	0.949153	-0.037857	0.23648	0.167216	-0.501357	0.425643	0.927	0.4635	0.376429	1.231309	0.107143	FALSE
RV_ ED Area[cm¸]	none	34	30	30	0.882353	0.001	0.31744	0.224464	-0.621182	0.623182	1.244363	0.622182	0.795833	0.781799	0.033333	59	56	56	0.949153	-0.01625	0.202642	0.14329	-0.413429	0.380929	0.794357	0.397179	0.870089	0.45648	0.035714	FALSE
RV_ ED Length[cm]	positive	34	30	30	0.882353	-0.051	0.249072	0.176121	-0.539181	0.437181	0.976363	0.488181	1.137167	0.429296	0.033333	59	56	56	0.949153	-0.038571	0.249344	0.176313	-0.527286	0.450143	0.977429	0.488714	1.202143	0.406536	0.035714	FALSE
RV_ ES Area[cm¸]	none	34	30	30	0.882353	-0.031	0.246553	0.17434	-0.514245	0.452245	0.96649	0.483245	0.6465	0.747478	0	59	56	56	0.949153	-0.049286	0.194187	0.137311	-0.429892	0.331321	0.761213	0.380606	0.671429	0.566861	0.035714	FALSE
RV_ ES Length[cm]	positive	34	30	30	0.882353	-0.046333	0.273767	0.193582	-0.582916	0.490249	1.073165	0.536583	1.0285	0.521714	0	59	56	56	0.949153	-0.073214	0.213628	0.151058	-0.491924	0.345496	0.83742	0.41871	1.026071	0.408071	0.053571	FALSE
LV_ EDV.Lt EDV % (AC)[%]	centile	34	19	19	0.558824	1.708947	30.380157	21.482015	-57.83616	61.254054	119.090214	59.545107	30.810263	1.932639	0.105263	59	28	28	0.474576	-8.782143	30.228077	21.374478	-68.029175	50.464889	118.494063	59.247032	38.926429	1.522026	0.035714	FALSE
LV_ EDV.Lt EDV % (AUA)[%]	centile	34	30	30	0.882353	-1.245333	28.518723	20.165782	-57.14203	54.651364	111.793394	55.896697	20.69	2.701629	0.1	59	54	54	0.915254	-9.743704	34.071442	24.092147	-76.523729	57.036322	133.560052	66.780026	32.777593	2.037368	0.185185	FALSE
LV_ EDV.Lt EDV % (BPD)[%]	centile	34	30	30	0.882353	-3.079667	31.787649	22.477262	-65.383458	59.224125	124.607583	62.303792	26.570833	2.344819	0.066667	59	51	51	0.864407	-8.824902	35.306637	24.965562	-78.02591	60.376106	138.402016	69.201008	40.085	1.726357	0.117647	FALSE
LV_ EDV.Lt EDV % (EFW)[%]	centile	34	16	16	0.470588	-2.1675	31.246763	22.094798	-63.411155	59.076155	122.48731	61.243655	34.2	1.79075	0	59	19	19	0.322034	-6.204211	28.6534	20.261013	-62.364874	49.956453	112.321326	56.160663	44.412632	1.26452	0	FALSE
LV_ EDV.Lt EDV % (FL)[%]	centile	34	17	17	0.5	1.511765	35.223892	24.907053	-67.527063	70.550593	138.077656	69.038828	31.934118	2.161914	0	59	33	33	0.559322	-5.512424	27.896983	19.726146	-60.190511	49.165663	109.356174	54.678087	41.995	1.302014	0	FALSE
LV_ EDV.Lt EDV % (GA)[%]	centile	34	28	28	0.823529	-7.674643	26.402301	18.669246	-59.423153	44.073867	103.497021	51.74851	21.833036	2.370193	0.035714	59	51	51	0.864407	-5.391176	30.659306	21.679403	-65.483416	54.701063	120.184479	60.092239	37.318137	1.610269	0.078431	FALSE
LV_ EDV.Lt EDV % (HC)[%]	centile	34	13	13	0.382353	-3.271538	40.064745	28.330053	-81.798439	75.255362	157.053801	78.526901	41.545769	1.89013	0	59	14	14	0.237288	-12.672857	40.772959	28.830835	-92.587856	67.242142	159.829997	79.914999	54.624286	1.462994	0	FALSE
LV_ EDV.Lt EDV ZS (AC)[]	none	34	19	19	0.558824	0.004737	1.306775	0.924029	-2.556541	2.566015	5.122556	2.561278	-0.999737	2.561952	0	59	28	28	0.474576	-0.566071	1.350019	0.954607	-3.212108	2.079965	5.292074	2.646037	-0.647679	4.085417	0	FALSE
LV_ EDV.Lt EDV ZS(AUA)[]	none	34	30	30	0.882353	-0.152	2.141342	1.514158	-4.349031	4.045031	8.394062	4.197031	-2.003667	2.094675	0	59	54	54	0.915254	-0.575741	2.334797	1.650951	-5.151943	4.000462	9.152405	4.576202	-1.482685	3.086429	0.018519	FALSE
LV_ EDV.Lt EDV ZS(BPD)[]	none	34	30	30	0.882353	0.020667	1.889734	1.336244	-3.683213	3.724546	7.407759	3.703879	-1.503	2.464324	0	59	51	51	0.864407	-0.379804	2.568218	1.816004	-5.413511	4.653903	10.067413	5.033707	-0.738137	6.819472	0.019608	FALSE
LV_ EDV.Lt EDV ZS (EFW)[]	none	34	16	16	0.470588	-0.02125	1.144517	0.809295	-2.264503	2.222003	4.486505	2.243253	-0.493125	4.549055	0	59	19	19	0.322034	-0.183684	1.04627	0.739824	-2.234373	1.867004	4.101377	2.050688	-0.183421	11.180224	0	FALSE
LV_ EDV.Lt EDV ZS(FL)[]	none	34	17	17	0.5	0.123529	1.277458	0.903299	-2.380289	2.627347	5.007636	2.503818	-0.741176	3.378167	0	59	33	33	0.559322	-0.25697	1.127421	0.797207	-2.466715	1.952775	4.41949	2.209745	-0.335152	6.593272	0	FALSE
LV_ EDV.Lt EDV ZS(GA)[]	none	34	28	28	0.823529	-0.256071	1.222558	0.864479	-2.652284	2.140141	4.792426	2.396213	-1.357321	1.765398	0	59	51	51	0.864407	-0.327451	1.724058	1.219093	-3.706604	3.051702	6.758307	3.379153	-0.767647	4.401962	0.019608	FALSE
LV_ EDV.Lt EDV ZS(HC)[]	none	34	13	13	0.382353	-0.120769	1.457958	1.030932	-2.978367	2.736828	5.715195	2.857597	-0.291923	9.788871	0	59	14	14	0.237288	-0.524286	1.591075	1.12506	-3.642792	2.594221	6.237013	3.118506	0.208571	14.951743	0	FALSE
4CV Trv. Width ED[mm]	positive	34	30	30	0.882353	0.123	0.433837	0.306769	-0.727321	0.973321	1.700642	0.850321	21.681833	0.039218	0.9	59	56	56	0.949153	0	0	0	0	0	0	0	21.502679	0	1	TRUE
4CV Length ED[mm]	positive	34	30	30	0.882353	0.047667	1.07952	0.763336	-2.068192	2.163526	4.231718	2.115859	26.6835	0.079295	0.9	59	56	56	0.949153	0	0	0	0	0	0	0	26.374821	0	1	TRUE
4CV Area (2Dist)[mm¸]	none	34	30	30	0.882353	3.778667	25.796759	18.241063	-46.782981	54.340314	101.123295	50.561647	484.057	0.104454	0.9	59	56	56	0.949153	0	0	0	0	0	0	0	476.532143	0	1	TRUE
4CV Trv. Width ED.4CV TW ED % (AC)[%]	centile	34	30	30	0.882353	0.402667	5.805764	4.105295	-10.976631	11.781964	22.758595	11.379298	47.156333	0.24131	0.9	59	56	56	0.949153	0	0	0	0	0	0	0	53.874821	0	1	TRUE
4CV Trv. Width ED.4CV TW ED % (AUA)[%]	centile	34	30	30	0.882353	0.432	8.261049	5.841444	-15.759656	16.623656	32.383311	16.191656	42.775	0.378531	0.9	59	54	54	0.915254	-0.048148	0.353815	0.250185	-0.741626	0.64533	1.386956	0.693478	55.41	0.012515	0.981481	FALSE
4CV Trv. Width ED.4CV TW ED % (BPD)[%]	centile	34	30	30	0.882353	-0.241333	7.774635	5.497497	-15.479617	14.99695	30.476568	15.238284	40.945667	0.372159	0.9	59	55	55	0.932203	0	0	0	0	0	0	0	47.601273	0	1	TRUE
4CV Trv. Width ED.4CV TW ED % (EFW)[%]	centile	34	30	30	0.882353	0.565	8.254458	5.836783	-15.613737	16.743737	32.357475	16.178737	44.178833	0.36621	0.9	59	55	55	0.932203	0	0	0	0	0	0	0	53.002545	0	1	TRUE
4CV Trv. Width ED.4CV TW ED % (FL)[%]	centile	34	29	29	0.852941	-0.088966	9.071403	6.414451	-17.868915	17.690984	35.5599	17.77995	38.673793	0.459742	0.896552	59	56	56	0.949153	0	0	0	0	0	0	0	49.5225	0	1	TRUE
4CV Trv. Width ED.4CV TW ED % (GA)[%]	centile	34	28	28	0.823529	-1.041429	6.268409	4.432435	-13.327511	11.244653	24.572164	12.286082	38.681786	0.317619	0.928571	59	51	51	0.864407	0	0	0	0	0	0	0	43.75	0	1	TRUE
4CV Trv. Width ED.4CV TW ED % (HC)[%]	centile	34	30	30	0.882353	0.187667	7.343196	5.192423	-14.204997	14.58033	28.785327	14.392663	40.1525	0.35845	0.9	59	55	55	0.932203	0	0	0	0	0	0	0	49.144182	0	1	TRUE
4CV Length ED.4CV L ED % (AC)[%]	centile	34	30	30	0.882353	-1.304333	6.790015	4.801266	-14.612763	12.004097	26.61686	13.30843	42.017167	0.316738	0.9	59	56	56	0.949153	0	0	0	0	0	0	0	45.200357	0	1	TRUE
4CV Length ED.4CV L ED % (AUA)[%]	centile	34	30	30	0.882353	-1.597333	6.017138	4.254759	-13.390923	10.196256	23.587179	11.79359	40.262	0.292921	0.9	59	54	54	0.915254	-0.036852	0.270805	0.191488	-0.567629	0.493925	1.061554	0.530777	45.421574	0.011686	0.981481	FALSE
4CV Length ED.4CV L ED % (BPD)[%]	centile	34	30	30	0.882353	-1.474333	4.890322	3.45798	-11.059365	8.110699	19.170064	9.585032	37.7765	0.25373	0.9	59	55	55	0.932203	0	0	0	0	0	0	0	40.787455	0	1	TRUE
4CV Length ED.4CV L ED % (EFW)[%]	centile	34	30	30	0.882353	-1.463667	6.13079	4.335123	-13.480015	10.552681	24.032696	12.016348	39.444167	0.304642	0.9	59	55	55	0.932203	0	0	0	0	0	0	0	41.148364	0	1	TRUE
4CV Length ED.4CV L ED % (FL)[%]	centile	34	29	29	0.852941	-2.405172	7.579969	5.359847	-17.261911	12.451566	29.713477	14.856739	39.375	0.377314	0.896552	59	56	56	0.949153	0	0	0	0	0	0	0	42.593571	0	1	TRUE
4CV Length ED.4CV L ED % (GA)[%]	centile	34	28	28	0.823529	-0.059643	1.992078	1.408612	-3.964115	3.84483	7.808945	3.904472	36.004107	0.108445	0.928571	59	51	51	0.864407	0	0	0	0	0	0	0	36.936078	0	1	TRUE
4CV Length ED.4CV L ED % (HC)[%]	centile	34	30	30	0.882353	-0.751333	6.257118	4.424451	-13.015285	11.512618	24.527904	12.263952	36.266333	0.338164	0.9	59	55	55	0.932203	0	0	0	0	0	0	0	38.994364	0	1	TRUE
4CV Area (2Dist).4CV A % (AC)[%]	centile	34	30	30	0.882353	-1.4	5.064931	3.581447	-11.327264	8.527264	19.854529	9.927264	52.408333	0.189421	0.9	59	56	56	0.949153	0	0	0	0	0	0	0	59.330893	0	1	TRUE
4CV Area (2Dist).4CV A % (AUA)[%]	centile	34	30	30	0.882353	-1.204667	5.432562	3.841401	-11.852488	9.443155	21.295643	10.647821	35.109667	0.303273	0.9	59	54	54	0.915254	-0.009074	0.066681	0.04715	-0.139768	0.12162	0.261388	0.130694	46.205093	0.002829	0.981481	FALSE
4CV Area (2Dist).4CV A % (BPD)[%]	centile	34	30	30	0.882353	-1.165667	5.144593	3.637777	-11.249069	8.917736	20.166805	10.083403	34.6545	0.29097	0.9	59	55	55	0.932203	0	0	0	0	0	0	0	39.815455	0	1	TRUE
4CV Area (2Dist).4CV A % (EFW)[%]	centile	34	30	30	0.882353	-1.016333	3.777993	2.671444	-8.4212	6.388533	14.809732	7.404866	36.978167	0.20025	0.9	59	55	55	0.932203	0	0	0	0	0	0	0	43.531636	0	1	TRUE
4CV Area (2Dist).4CV A % (FL)[%]	centile	34	29	29	0.852941	-1.852414	8.295042	5.86548	-18.110696	14.405868	32.516564	16.258282	36.492414	0.445525	0.896552	59	56	56	0.949153	0	0	0	0	0	0	0	44.356786	0	1	TRUE
4CV Area (2Dist).4CV A % (HC)[%]	centile	34	30	30	0.882353	-1.025	4.602462	3.254432	-10.045825	7.995825	18.04165	9.020825	32.416167	0.278282	0.9	59	55	55	0.932203	0	0	0	0	0	0	0	40.843636	0	1	TRUE
4CV Trv. Width ED.4CV TW ED ZS (AC)[]	none	34	30	30	0.882353	-0.002667	0.211268	0.149389	-0.416751	0.411418	0.828169	0.414085	-0.105	3.943663	0.9	59	56	56	0.949153	0	0	0	0	0	0	0	0.180179	0	1	TRUE
4CV Trv. Width ED.4CV TW ED ZS(AUA)[]	none	34	30	30	0.882353	-0.049333	0.582539	0.411917	-1.191109	1.092442	2.283551	1.141776	-0.368	3.102651	0.9	59	54	54	0.915254	-0.007037	0.051711	0.036566	-0.108391	0.094317	0.202709	0.101354	0.247222	0.409973	0.981481	FALSE
4CV Trv. Width ED.4CV TW ED ZS(BPD)[]	none	34	30	30	0.882353	-0.044	0.50513	0.357181	-1.034054	0.946054	1.980108	0.990054	-0.508333	1.947647	0.9	59	55	55	0.932203	0	0	0	0	0	0	0	-0.076727	0	1	TRUE
4CV Trv. Width ED.4CV TW ED ZS (EFW)[]	none	34	30	30	0.882353	-0.033	0.44495	0.314627	-0.905102	0.839102	1.744203	0.872102	-0.213833	4.078418	0.9	59	55	55	0.932203	0	0	0	0	0	0	0	0.166364	0	1	TRUE
4CV Trv. Width ED.4CV TW ED ZS(FL)[]	none	34	29	29	0.852941	-0.068276	0.641711	0.453758	-1.32603	1.189478	2.515508	1.257754	-0.431724	2.913328	0.896552	59	56	56	0.949153	0	0	0	0	0	0	0	-0.031786	0	1	TRUE
4CV Trv. Width ED.4CV TW ED ZS(GA)[]	none	34	28	28	0.823529	-0.053929	0.369217	0.261076	-0.777593	0.669736	1.44733	0.723665	-0.587321	1.232145	0.928571	59	51	51	0.864407	0	0	0	0	0	0	0	-0.169608	0	1	TRUE
4CV Trv. Width ED.4CV TW ED ZS(HC)[]	none	34	30	30	0.882353	-0.029667	0.469405	0.33192	-0.949701	0.890368	1.840068	0.920034	-0.5795	1.587635	0.9	59	55	55	0.932203	0	0	0	0	0	0	0	0.018	0	1	TRUE
4CV Length ED.4CV L ED ZS (AC)[]	none	34	30	30	0.882353	-0.041	0.198917	0.140655	-0.430877	0.348877	0.779754	0.389877	-0.295167	1.320871	0.9	59	56	56	0.949153	0	0	0	0	0	0	0	-0.115357	0	1	TRUE
4CV Length ED.4CV L ED ZS(AUA)[]	none	34	30	30	0.882353	-0.067	0.225666	0.15957	-0.509305	0.375305	0.884611	0.442305	-0.396833	1.114588	0.9	59	54	54	0.915254	-0.004074	0.029938	0.02117	-0.062753	0.054605	0.117358	0.058679	-0.130741	0.448819	0.981481	FALSE
4CV Length ED.4CV L ED ZS(BPD)[]	none	34	30	30	0.882353	-0.059667	0.194165	0.137295	-0.440229	0.320896	0.761125	0.380563	-0.4545	0.837321	0.9	59	55	55	0.932203	0	0	0	0	0	0	0	-0.292	0	1	TRUE
4CV Length ED.4CV L ED ZS (EFW)[]	none	34	30	30	0.882353	-0.053667	0.208384	0.14735	-0.4621	0.354767	0.816867	0.408433	-0.4055	1.007234	0.9	59	55	55	0.932203	0	0	0	0	0	0	0	-0.267091	0	1	TRUE
4CV Length ED.4CV L ED ZS(FL)[]	none	34	29	29	0.852941	-0.084483	0.253456	0.17922	-0.581256	0.412291	0.993547	0.496774	-0.410862	1.209101	0.896552	59	56	56	0.949153	0	0	0	0	0	0	0	-0.252143	0	1	TRUE
4CV Length ED.4CV L ED ZS(GA)[]	none	34	28	28	0.823529	-0.025357	0.16827	0.118985	-0.355166	0.304451	0.659617	0.329809	-0.576607	0.571981	0.928571	59	51	51	0.864407	0	0	0	0	0	0	0	-0.454118	0	1	TRUE
4CV Length ED.4CV L ED ZS(HC)[]	none	34	30	30	0.882353	-0.050667	0.245173	0.173364	-0.531206	0.429873	0.961079	0.480539	-0.551333	0.871595	0.9	59	55	55	0.932203	0	0	0	0	0	0	0	-0.389091	0	1	TRUE
4CV Area (2Dist).4CV A ZS (AC)[]	none	34	30	30	0.882353	-0.051667	0.201393	0.142406	-0.446397	0.343064	0.789461	0.394731	0.0925	4.267357	0.9	59	56	56	0.949153	0	0	0	0	0	0	0	0.439821	0	1	TRUE
4CV Area (2Dist).4CV A ZS(AUA)[]	none	34	30	30	0.882353	-0.089333	0.371706	0.262836	-0.817876	0.63921	1.457086	0.728543	-0.649	1.122562	0.9	59	54	54	0.915254	-0.009444	0.069402	0.049075	-0.145473	0.126584	0.272057	0.136028	-0.180463	0.753774	0.981481	FALSE
4CV Area (2Dist).4CV A ZS(BPD)[]	none	34	30	30	0.882353	-0.046	0.180146	0.127382	-0.399085	0.307085	0.70617	0.353085	-0.540333	0.653458	0.9	59	55	55	0.932203	0	0	0	0	0	0	0	-0.340909	0	1	TRUE
4CV Area (2Dist).4CV A ZS (EFW)[]	none	34	30	30	0.882353	-0.041333	0.150602	0.106492	-0.336513	0.253846	0.590359	0.29518	-0.431	0.684872	0.9	59	55	55	0.932203	0	0	0	0	0	0	0	-0.168	0	1	TRUE
4CV Area (2Dist).4CV A ZS(FL)[]	none	34	29	29	0.852941	-0.105517	0.45957	0.324965	-1.006274	0.795239	1.801513	0.900756	-0.503793	1.787949	0.896552	59	56	56	0.949153	0	0	0	0	0	0	0	-0.191429	0	1	TRUE
4CV Area (2Dist).4CV A ZS(HC)[]	none	34	30	30	0.882353	-0.061667	0.236703	0.167374	-0.525604	0.402271	0.927875	0.463937	-0.779167	0.595428	0.9	59	55	55	0.932203	0	0	0	0	0	0	0	-0.454	0	1	TRUE
RV_ FreeWall Strain[%]	strain_raw	34	30	22	0.647059	2.939091	14.162485	10.014389	-24.819379	30.697561	55.51694	27.75847	-14.962273	1.855231	0	59	56	50	0.847458	-3.139	13.419364	9.488923	-29.440954	23.162954	52.603908	26.301954	-18.0679	1.455728	0.017857	FALSE
LV_ MAPSE septal[mm]	positive	34	30	20	0.588235	-0.265	1.665209	1.177481	-3.52881	2.99881	6.527619	3.26381	2.6625	1.225844	0.033333	59	56	42	0.711864	-0.030952	1.766696	1.249243	-3.493676	3.431771	6.925447	3.462724	3.039286	1.139322	0.017857	FALSE
LV_ MAPSE lateral[mm]	positive	34	30	20	0.588235	-0.42	1.651984	1.168129	-3.65789	2.81789	6.475779	3.23789	2.24	1.445486	0.033333	59	56	42	0.711864	-0.004762	1.581903	1.118574	-3.105291	3.095767	6.201058	3.100529	2.204762	1.406287	0.035714	FALSE
RV_ TAPSE septal[mm]	tapse_mm	34	30	12	0.352941	-0.55	2.318895	1.639706	-5.095034	3.995034	9.090067	4.545034	1.875	2.424018	0.066667	59	55	39	0.661017	0.387179	1.596077	1.128597	-2.741132	3.515491	6.256623	3.128312	2.170513	1.441278	0.036364	FALSE
RV_ TAPSE lateral[mm]	tapse_mm	34	30	16	0.470588	-0.0625	1.252132	0.885391	-2.516678	2.391678	4.908356	2.454178	2.36875	1.036064	0.033333	59	56	45	0.762712	0.584444	1.919512	1.3573	-3.177799	4.346687	7.524486	3.762243	2.621111	1.435362	0.017857	FALSE
LV_ MAPSE septal.MAPSE sept. % (AC)[%]	centile	34	30	30	0.882353	-1.918333	38.963201	27.551144	-78.286208	74.449541	152.735749	76.367875	29.7335	2.568412	0.033333	59	56	56	0.949153	-0.1275	42.055918	29.738025	-82.5571	82.3021	164.8592	82.4296	42.542321	1.937591	0.053571	FALSE
LV_ MAPSE septal.MAPSE sept. % (AUA)[%]	centile	34	30	30	0.882353	1.258667	6.478281	4.580837	-11.438765	13.956098	25.394863	12.697431	2.659667	4.774069	0.9	59	54	54	0.915254	0.662037	4.979938	3.521348	-9.098641	10.422715	19.521356	9.760678	0.4525	21.570559	0.888889	FALSE
LV_ MAPSE septal.MAPSE sept. % (BPD)[%]	centile	34	30	30	0.882353	-1.445667	38.851575	27.472212	-77.594753	74.70342	152.298173	76.149086	28.673833	2.6557	0.033333	59	55	55	0.932203	1.712909	40.056795	28.324432	-76.79841	80.224228	157.022637	78.511319	41.628455	1.886001	0.090909	FALSE
LV_ MAPSE septal.MAPSE sept. % (EFW)[%]	centile	34	30	30	0.882353	-1.561333	38.337645	27.108809	-76.703117	73.580451	150.283568	75.141784	29.521	2.545367	0.033333	59	55	55	0.932203	1.241455	39.901877	28.214888	-76.966225	79.449134	156.415359	78.207679	42.113636	1.857063	0.054545	FALSE
LV_ MAPSE septal.MAPSE sept. % (FL)[%]	centile	34	29	29	0.852941	1.713448	35.51311	25.111561	-67.892248	71.319144	139.211392	69.605696	27.878103	2.496787	0.034483	59	56	56	0.949153	-0.604643	42.203054	29.842066	-83.322629	82.113343	165.435972	82.717986	41.321429	2.001818	0.089286	FALSE
LV_ MAPSE septal.MAPSE sept. % (GA)[%]	centile	34	28	28	0.823529	-1.615714	39.831828	28.165355	-79.686096	76.454668	156.140764	78.070382	30.919643	2.524944	0.035714	59	51	51	0.864407	-0.455098	43.607769	30.835349	-85.926325	85.016129	170.942454	85.471227	41.193039	2.074895	0.058824	FALSE
LV_ MAPSE septal.MAPSE sept. % (HC)[%]	centile	34	30	30	0.882353	-1.284	38.915995	27.517764	-77.559351	74.991351	152.550702	76.275351	28.791333	2.649247	0.033333	59	55	55	0.932203	1.281636	40.631401	28.730739	-78.35591	80.919183	159.275092	79.637546	42.369727	1.879586	0.090909	FALSE
LV_ MAPSE lateral.MAPSE lat. % (AC)[%]	centile	34	30	30	0.882353	-8.347667	36.289332	25.660432	-79.474756	62.779423	142.25418	71.12709	17.6545	4.028836	0.066667	59	56	56	0.949153	-0.569821	36.252013	25.634044	-71.623767	70.484124	142.107891	71.053946	17.015268	4.175893	0.142857	FALSE
LV_ MAPSE lateral.MAPSE lat. % (AUA)[%]	centile	34	30	30	0.882353	-8.561	36.787023	26.012353	-80.663565	63.541565	144.20513	72.102565	17.377167	4.14927	0.066667	59	54	54	0.915254	-0.436296	37.043353	26.193606	-73.041268	72.168675	145.209944	72.604972	17.533704	4.140881	0.111111	FALSE
LV_ MAPSE lateral.MAPSE lat. % (BPD)[%]	centile	34	30	30	0.882353	-8.330333	36.409292	25.745257	-79.692546	63.031879	142.724426	71.362213	17.235167	4.1405	0.1	59	55	55	0.932203	0.267636	35.930972	25.407034	-70.157069	70.692342	140.849411	70.424706	16.367091	4.302824	0.145455	FALSE
LV_ MAPSE lateral.MAPSE lat. % (EFW)[%]	centile	34	30	30	0.882353	-9.089667	36.571492	25.85995	-80.769792	62.590458	143.36025	71.680125	18.5945	3.85491	0.033333	59	55	55	0.932203	-0.019636	35.548412	25.136523	-69.694524	69.655252	139.349776	69.674888	18.343455	3.798351	0.090909	FALSE
LV_ MAPSE lateral.MAPSE lat. % (FL)[%]	centile	34	29	29	0.852941	-6.17	34.749817	24.571832	-74.279642	61.939642	136.219284	68.109642	16.546034	4.116373	0.068966	59	56	56	0.949153	-0.727143	36.209974	25.604318	-71.698692	70.244406	141.943097	70.971549	17.017679	4.17046	0.125	FALSE

parameter	qc_rule	intra_n_total	intra_n_complete	intra_n_usable	intra_usable_rate	intra_bias	intra_sd_diff	intra_sw	intra_loa_low	intra_loa_high	intra_loa_width	intra_mdc95	intra_mean	intra_rel_mdc95	intra_zero_diff_rate	inter_n_total	inter_n_complete	inter_n_usable	inter_usable_rate	inter_bias	inter_sd_diff	inter_sw	inter_loa_low	inter_loa_high	inter_loa_width	inter_mdc95	inter_mean	inter_rel_mdc95	inter_zero_diff_rate	possible_copy_forward_inter
LV_ MAPSE lateral.MAPSE lat. % (GA)[%]	centile	34	28	28	0.823529	-8.798214	36.694502	25.946931	-80.719439	63.12301	143.842449	71.921225	18.056964	3.983019	0.107143	59	51	51	0.864407	-0.519412	37.725143	26.675704	-74.460692	73.421868	147.882559	73.94128	15.925	4.643094	0.156863	FALSE
LV_ MAPSE lateral.MAPSE lat. % (HC)[%]	centile	34	30	30	0.882353	-8.490667	36.732254	25.973626	-80.485884	63.50455	143.990434	71.995217	16.907333	4.258224	0.133333	59	55	55	0.932203	0.281091	36.453407	25.776451	-71.167587	71.729769	142.897356	71.448678	15.974909	4.472556	0.145455	FALSE
RV_ TAPSE septal.TAPSE sept. % (AC)[%]	centile	34	30	30	0.882353	-1.5	35.040611	24.777454	-70.179598	67.179598	137.359197	68.679598	10.474667	6.556734	0.133333	59	55	55	0.932203	10.869818	40.86162	28.893529	-69.218957	90.958594	160.177551	80.088775	22.114364	3.621573	0.090909	FALSE
RV_ TAPSE septal.TAPSE sept. % (AUA)[%]	centile	34	30	30	0.882353	-1.120667	34.304999	24.257297	-68.358464	66.11713	134.475594	67.237797	10.048	6.69166	0.133333	59	53	53	0.898305	11.750943	40.880132	28.906619	-68.374115	91.876002	160.250117	80.125059	22.2	3.609237	0.09434	FALSE
RV_ TAPSE septal.TAPSE sept. % (BPD)[%]	centile	34	30	30	0.882353	-1.197333	33.452417	23.654431	-66.764071	64.369405	131.133476	65.566738	9.654	6.791665	0.166667	59	54	54	0.915254	11.796481	40.432732	28.590259	-67.451674	91.044637	158.496311	79.248156	20.165093	3.929967	0.092593	FALSE
RV_ TAPSE septal.TAPSE sept. % (EFW)[%]	centile	34	30	30	0.882353	-0.495667	33.94562	24.003178	-67.029081	66.037748	133.066829	66.533415	10.1285	6.568931	0.1	59	54	54	0.915254	11.525556	40.397018	28.565005	-67.6526	90.703711	158.35631	79.178155	22.03537	3.59323	0.092593	FALSE
RV_ TAPSE septal.TAPSE sept. % (FL)[%]	centile	34	29	29	0.852941	-1.137586	34.076689	24.095858	-67.927896	65.652724	133.58062	66.79031	9.787414	6.824102	0.137931	59	55	55	0.932203	11.034364	39.664841	28.047278	-66.708724	88.777452	155.486176	77.743088	20.506818	3.791085	0.090909	FALSE
RV_ TAPSE septal.TAPSE sept. % (GA)[%]	centile	34	28	28	0.823529	-1.731429	34.721747	24.551983	-69.786053	66.323196	136.109248	68.054624	10.034643	6.781968	0.107143	59	50	50	0.847458	11.466	41.308277	29.209363	-69.498223	92.430223	161.928446	80.964223	21.742	3.723863	0.08	FALSE
RV_ TAPSE septal.TAPSE sept. % (HC)[%]	centile	34	30	30	0.882353	-1.100333	34.035553	24.06677	-67.810017	65.60935	133.419366	66.709683	9.826833	6.788523	0.166667	59	54	54	0.915254	11.787963	40.437152	28.593384	-67.468854	91.04478	158.513635	79.256817	21.153981	3.746662	0.092593	FALSE
RV_ TAPSE lateral.TAPSE lat. % (AC)[%]	centile	34	30	30	0.882353	-5.029333	26.256592	18.566214	-56.492254	46.433587	102.925841	51.46292	10.481333	4.909959	0.1	59	56	56	0.949153	5.767143	31.21043	22.069107	-55.4053	66.939586	122.344887	61.172443	17.914286	3.41473	0.071429	FALSE
RV_ TAPSE lateral.TAPSE lat. % (AUA)[%]	centile	34	30	30	0.882353	-4.871333	25.776623	18.226825	-55.393515	45.650848	101.044363	50.522182	9.248333	5.462842	0.133333	59	54	54	0.915254	7.289444	30.023735	21.229986	-51.557075	66.135964	117.693039	58.84652	17.619167	3.339915	0.074074	FALSE
RV_ TAPSE lateral.TAPSE lat. % (BPD)[%]	centile	34	30	30	0.882353	-4.663	25.433285	17.984048	-54.512238	45.186238	99.698476	49.849238	8.9585	5.564463	0.133333	59	55	55	0.932203	5.551455	29.573482	20.911609	-52.412569	63.515478	115.928048	57.964024	16.350455	3.545102	0.072727	FALSE
RV_ TAPSE lateral.TAPSE lat. % (EFW)[%]	centile	34	30	30	0.882353	-4.717	25.683507	18.160982	-55.056675	45.622675	100.679349	50.339675	9.8035	5.134868	0.066667	59	55	55	0.932203	6.071091	31.019413	21.934038	-54.726959	66.869141	121.5961	60.79805	17.978818	3.381649	0.072727	FALSE
RV_ TAPSE lateral.TAPSE lat. % (FL)[%]	centile	34	29	29	0.852941	-4.642759	25.23596	17.844519	-54.105241	44.819723	98.924964	49.462482	9.244138	5.350686	0.068966	59	56	56	0.949153	6.320536	30.61146	21.645571	-53.677926	66.318998	119.996924	59.998462	16.824018	3.566239	0.071429	FALSE
RV_ TAPSE lateral.TAPSE lat. % (GA)[%]	centile	34	28	28	0.823529	-5.501071	25.961482	18.35754	-56.385575	45.383433	101.769008	50.884504	9.125179	5.576275	0.178571	59	51	51	0.864407	8.437451	29.525954	20.878002	-49.433418	66.30832	115.741739	57.870869	15.485196	3.737174	0.058824	FALSE
RV_ TAPSE lateral.TAPSE lat. % (HC)[%]	centile	34	30	30	0.882353	-4.659	25.494159	18.027093	-54.627553	45.309553	99.937105	49.968553	8.679833	5.756856	0.133333	59	55	55	0.932203	5.757636	29.921633	21.15779	-52.888765	64.404038	117.292802	58.646401	16.835182	3.483562	0.072727	FALSE
LV_ MAPSE septal.MAPSE sept. ZS (AC)[]	none	34	30	30	0.882353	0.112	2.978625	2.106206	-5.726106	5.950106	11.676212	5.838106	-1.206333	4.839546	0.033333	59	56	56	0.949153	-0.195714	2.217446	1.567971	-4.541909	4.150481	8.69239	4.346195	-0.549286	7.912449	0.017857	FALSE
LV_ MAPSE septal.MAPSE sept. ZS(AUA)[]	none	34	30	30	0.882353	0.211667	3.02671	2.140207	-5.720685	6.144018	11.864703	5.932351	-8.618833	0.688301	0.033333	59	54	54	0.915254	-0.191481	2.236239	1.58126	-4.574509	4.191546	8.766056	4.383028	-8.282037	0.529221	0.018519	FALSE
LV_ MAPSE septal.MAPSE sept. ZS(BPD)[]	none	34	30	30	0.882353	0.119	3.0556	2.160636	-5.869976	6.107976	11.977952	5.988976	-1.308167	4.578145	0.033333	59	55	55	0.932203	-0.078727	2.078977	1.470059	-4.153522	3.996067	8.149589	4.074795	-0.599727	6.794413	0.018182	FALSE
LV_ MAPSE septal.MAPSE sept. ZS (EFW)[]	none	34	30	30	0.882353	0.097667	2.888038	2.042151	-5.562888	5.758221	11.321109	5.660554	-1.193167	4.744144	0.033333	59	55	55	0.932203	-0.066182	1.969358	1.392547	-3.926124	3.79376	7.719885	3.859942	-0.538727	7.164928	0.018182	FALSE
LV_ MAPSE septal.MAPSE sept. ZS(FL)[]	none	34	29	29	0.852941	0.248276	2.994717	2.117585	-5.621369	6.117921	11.73929	5.869645	-1.381034	4.25018	0.034483	59	56	56	0.949153	-0.206429	2.28634	1.616687	-4.687655	4.274798	8.962453	4.481226	-0.643571	6.96306	0.017857	FALSE
LV_ MAPSE septal.MAPSE sept. ZS(GA)[]	none	34	28	28	0.823529	0.088929	3.09957	2.191727	-5.986229	6.164086	12.150315	6.075158	-1.149821	5.283566	0.035714	59	51	51	0.864407	-0.201765	2.324977	1.644007	-4.75872	4.355191	9.11391	4.556955	-0.642255	7.095244	0	FALSE
LV_ MAPSE septal.MAPSE sept. ZS(HC)[]	none	34	30	30	0.882353	0.119667	3.123971	2.208981	-6.003317	6.24265	12.245967	6.122984	-1.313833	4.660396	0.033333	59	55	55	0.932203	-0.082182	2.138863	1.512405	-4.274354	4.10999	8.384344	4.192172	-0.563636	7.437724	0.018182	FALSE
LV_ MAPSE lateral.MAPSE lat. ZS (AC)[]	none	34	30	30	0.882353	0.143	3.189877	2.255584	-6.10916	6.39516	12.50432	6.25216	-2.4135	2.590495	0.033333	59	56	56	0.949153	-0.329821	2.2278	1.575292	-4.696309	4.036667	8.732976	4.366488	-1.997589	2.185879	0.035714	FALSE
LV_ MAPSE lateral.MAPSE lat. ZS(AUA)[]	none	34	30	30	0.882353	0.137667	3.205499	2.26663	-6.145112	6.420445	12.565557	6.282779	-2.459833	2.554148	0.033333	59	54	54	0.915254	-0.343889	2.336256	1.651982	-4.922951	4.235173	9.158124	4.579062	-1.984537	2.30737	0.018519	FALSE
LV_ MAPSE lateral.MAPSE lat. ZS(BPD)[]	none	34	30	30	0.882353	0.167667	3.250541	2.29848	-6.203394	6.538728	12.742122	6.371061	-2.495833	2.552679	0.033333	59	55	55	0.932203	-0.245273	2.191877	1.549891	-4.541352	4.050806	8.592158	4.296079	-2.068636	2.076769	0.036364	FALSE
LV_ MAPSE lateral.MAPSE lat. ZS (EFW)[]	none	34	30	30	0.882353	0.077667	2.809576	1.98667	-5.429102	5.584435	11.013536	5.506768	-2.149167	2.562281	0.033333	59	55	55	0.932203	-0.202364	1.930774	1.365263	-3.986681	3.581954	7.568635	3.784317	-1.734273	2.182077	0.036364	FALSE
LV_ MAPSE lateral.MAPSE lat. ZS(FL)[]	none	34	29	29	0.852941	0.222759	3.024211	2.13844	-5.704694	6.150211	11.854905	5.927453	-2.438276	2.431002	0.034483	59	56	56	0.949153	-0.325357	2.201841	1.556937	-4.640966	3.990252	8.631218	4.315609	-1.971071	2.189474	0.035714	FALSE
LV_ MAPSE lateral.MAPSE lat. ZS(GA)[]	none	34	28	28	0.823529	-0.0675	3.107404	2.197267	-6.158012	6.023012	12.181024	6.090512	-2.351607	2.589936	0.035714	59	51	51	0.864407	-0.339412	2.281588	1.613327	-4.811325	4.132502	8.943827	4.471913	-2.13951	2.090158	0.039216	FALSE
LV_ MAPSE lateral.MAPSE lat. ZS(HC)[]	none	34	30	30	0.882353	0.16	3.392079	2.398562	-6.488475	6.808475	13.29695	6.648475	-2.600667	2.55645	0.033333	59	55	55	0.932203	-0.272727	2.320024	1.640504	-4.819973	4.274519	9.094492	4.547246	-2.175818	2.089902	0.036364	FALSE
RV_ TAPSE septal.TAPSE sept. ZS (AC)[]	none	34	30	30	0.882353	0.171333	4.20205	2.971298	-8.064685	8.407352	16.472037	8.236018	-2.918667	2.821843	0.066667	59	55	55	0.932203	0.327091	2.705888	1.913352	-4.976449	5.630631	10.60708	5.30354	-1.766636	3.002055	0.036364	FALSE
RV_ TAPSE septal.TAPSE sept. ZS(AUA)[]	none	34	30	30	0.882353	0.165	4.144888	2.930878	-7.95898	8.28898	16.24796	8.12398	-2.9485	2.755293	0.066667	59	53	53	0.898305	0.456792	2.680326	1.895276	-4.796646	5.710231	10.506876	5.253438	-1.761415	2.98251	0.037736	FALSE
RV_ TAPSE septal.TAPSE sept. ZS(BPD)[]	none	34	30	30	0.882353	0.206333	4.515891	3.193217	-8.644813	9.05748	17.702293	8.851147	-3.326167	2.661065	0.066667	59	54	54	0.915254	0.452963	2.930394	2.072102	-5.29061	6.196536	11.487145	5.743573	-2.068889	2.776163	0.037037	FALSE
RV_ TAPSE septal.TAPSE sept. ZS (EFW)[]	none	34	30	30	0.882353	0.152667	4.063641	2.873428	-7.81207	8.117403	15.929473	7.964737	-2.811	2.833417	0.066667	59	54	54	0.915254	0.417593	2.5507	1.803617	-4.58178	5.416965	9.998745	4.999372	-1.697685	2.944817	0.037037	FALSE
RV_ TAPSE septal.TAPSE sept. ZS(FL)[]	none	34	29	29	0.852941	0.08069	4.069344	2.877461	-7.895225	8.056605	15.95183	7.975915	-2.924138	2.727612	0.068966	59	55	55	0.932203	0.319091	2.634325	1.862749	-4.844186	5.482368	10.326554	5.163277	-1.860091	2.77582	0.036364	FALSE
RV_ TAPSE septal.TAPSE sept. ZS(GA)[]	none	34	28	28	0.823529	-0.141071	4.176345	2.953122	-8.326707	8.044564	16.371271	8.185635	-2.885893	2.836431	0.071429	59	50	50	0.847458	0.4924	2.783583	1.96829	-4.963422	5.948222	10.911644	5.455822	-1.815	3.005963	0.02	FALSE
RV_ TAPSE septal.TAPSE sept. ZS(HC)[]	none	34	30	30	0.882353	0.184667	4.219128	2.983374	-8.084824	8.454157	16.538981	8.269491	-3.028	2.731007	0.066667	59	54	54	0.915254	0.418519	2.709611	1.915985	-4.89232	5.729357	10.621677	5.310838	-1.865	2.847635	0.037037	FALSE
RV_ TAPSE lateral.TAPSE lat. ZS (AC)[]	none	34	30	30	0.882353	-0.062	2.543907	1.798814	-5.048058	4.924058	9.972117	4.986058	-2.613667	1.907687	0.033333	59	56	56	0.949153	0.331071	1.836089	1.298311	-3.267664	3.929807	7.19747	3.598735	-1.88875	1.905353	0.017857	FALSE
RV_ TAPSE lateral.TAPSE lat. ZS(AUA)[]	none	34	30	30	0.882353	-0.068333	2.518279	1.780693	-5.004161	4.867494	9.871656	4.935828	-2.659167	1.856156	0.033333	59	54	54	0.915254	0.412222	1.791946	1.267097	-3.099992	3.924436	7.024428	3.512214	-1.912222	1.836719	0.018519	FALSE
RV_ TAPSE lateral.TAPSE lat. ZS(BPD)[]	none	34	30	30	0.882353	-0.065	2.538318	1.794862	-5.040104	4.910104	9.950208	4.975104	-2.7405	1.8154	0.033333	59	55	55	0.932203	0.382	1.808797	1.279012	-3.163242	3.927242	7.090484	3.545242	-1.977727	1.792584	0.018182	FALSE
RV_ TAPSE lateral.TAPSE lat. ZS (EFW)[]	none	34	30	30	0.882353	-0.075333	2.480221	1.753781	-4.936566	4.785899	9.722465	4.861233	-2.580333	1.883955	0.033333	59	55	55	0.932203	0.374909	1.757804	1.242955	-3.070386	3.820204	6.89059	3.445295	-1.848727	1.863604	0.018182	FALSE
RV_ TAPSE lateral.TAPSE lat. ZS(FL)[]	none	34	29	29	0.852941	-0.097241	2.480741	1.754149	-4.959494	4.765012	9.724506	4.862253	-2.608966	1.863671	0.034483	59	56	56	0.949153	0.318036	1.751596	1.238565	-3.115093	3.751164	6.866257	3.433128	-1.935982	1.773326	0.017857	FALSE
RV_ TAPSE lateral.TAPSE lat. ZS(GA)[]	none	34	28	28	0.823529	-0.364286	2.346794	1.659434	-4.964001	4.23543	9.199431	4.599715	-2.653214	1.733639	0.035714	59	51	51	0.864407	0.442157	1.899741	1.34332	-3.281336	4.16565	7.446986	3.723493	-2.06951	1.799215	0	FALSE
RV_ TAPSE lateral.TAPSE lat. ZS(HC)[]	none	34	30	30	0.882353	-0.064	2.530003	1.788982	-5.022806	4.894806	9.917611	4.958806	-2.735	1.813092	0.033333	59	55	55	0.932203	0.382	1.800794	1.273354	-3.147557	3.911557	7.059114	3.529557	-1.940818	1.818592	0.018182	FALSE
LV_ FreeWall Strain[%]	strain_raw	34	30	23	0.676471	1.878696	10.397304	7.352004	-18.50002	22.257411	40.757432	20.378716	-14.919348	1.365925	0	59	56	50	0.847458	-0.143	12.713921	8.990099	-25.062284	24.776284	49.838569	24.919284	-15.3463	1.623798	0	FALSE
LV_ Sept.Wall Strain[%]	strain_raw	34	30	24	0.705882	0.476667	9.244297	6.536705	-17.642156	18.595489	36.237645	18.118823	-15.003333	1.207653	0	59	56	48	0.813559	-0.584375	12.762096	9.024164	-25.598082	24.429332	50.027415	25.013707	-18.062812	1.384818	0	FALSE
RV_ Sept.Wall Strain[%]	strain_raw	34	30	22	0.647059	0.500455	9.374992	6.629121	-17.87453	18.875439	36.74997	18.374985	-12.381591	1.484057	0	59	56	50	0.847458	-1.5914	9.712862	6.86803	-20.628609	17.445809	38.074419	19.037209	-15.4133	1.235116	0	FALSE
LV_ Basal-Lat.Wall FS[%]	percent_0_100	34	30	21	0.617647	-2.397619	11.121786	7.86429	-24.19632	19.401082	43.597402	21.798701	15.555952	1.401309	0	59	56	43	0.728814	0.184186	9.998623	7.070094	-19.413115	19.781487	39.194602	19.597301	15.474651	1.266413	0	FALSE
RV_ Basal-Lat.Wall FS[%]	percent_0_100	34	30	17	0.5	-0.648824	11.115002	7.859493	-22.434227	21.13658	43.570806	21.785403	18.417941	1.182836	0	59	56	45	0.762712	4.310444	12.874957	9.103969	-20.924471	29.54536	50.469831	25.234915	19.038778	1.325448	0	FALSE
LV_ Basal-Sep.Wall FS[%]	percent_0_100	34	30	20	0.588235	-2.382	9.114999	6.445278	-20.247398	15.483398	35.730796	17.865398	17.522	1.019598	0	59	56	42	0.711864	0.34	11.444836	8.092721	-22.091879	22.771879	44.863758	22.431879	20.470476	1.095816	0	FALSE
RV_ Basal-Sep.Wall FS[%]	percent_0_100	34	30	14	0.411765	2.885714	18.995629	13.431938	-34.345719	40.117148	74.462867	37.231434	14.295	2.604507	0	59	56	40	0.677966	2.52475	10.987092	7.769047	-19.00995	24.05945	43.0694	21.5347	16.602625	1.297066	0	FALSE
LV_ Basal-Apical FS[%]	percent_0_100	34	30	21	0.617647	-1.538571	10.383607	7.342319	-21.890442	18.813299	40.703741	20.351871	16.334048	1.245978	0	59	56	44	0.745763	0.032727	11.331825	8.012811	-22.177651	22.243105	44.420756	22.210378	18.286136	1.214602	0	FALSE
RV_ Basal-Apical FS[%]	percent_0_100	34	30	16	0.470588	-0.490625	14.865156	10.511253	-29.626331	28.645081	58.271413	29.135706	16.405938	1.775925	0	59	56	45	0.762712	3.815333	11.352881	8.027699	-18.436314	26.066981	44.503294	22.251647	17.910556	1.242376	0	FALSE
LV_ Global Strain.Lt GLS %[%]	centile	34	30	30	0.882353	0.937333	32.554858	23.019761	-62.870189	64.744855	127.615044	63.807522	81.413333	0.783748	0.333333	59	56	56	0.949153	0.481964	41.456059	29.31386	-80.771911	81.73584	162.507751	81.253876	73.939196	1.098928	0.196429	FALSE
RV_ Global Strain.Rt GLS %[%]	centile	34	30	30	0.882353	-1.213667	36.027948	25.475607	-71.828446	69.401112	141.229558	70.614779	88.759833	0.795571	0.2	59	56	56	0.949153	-9.690714	35.350691	24.996713	-78.978069	59.59664	138.574709	69.287354	80.089107	0.865128	0.125	FALSE
RV_ FreeWall Strain.Rt FWS %[%]	centile	34	30	30	0.882353	2.079333	32.546533	23.013875	-61.711872	65.870539	127.582411	63.791206	83.873333	0.760566	0.2	59	56	56	0.949153	-11.788393	33.425235	23.63521	-77.301853	53.725068	131.026921	65.513461	76.732054	0.853795	0.107143	FALSE
LV_ FreeWall Strain.Lt FWS %[%]	centile	34	30	30	0.882353	6.683	38.658553	27.335725	-69.087763	82.453763	151.541527	75.770763	81.8255	0.926004	0.1	59	56	56	0.949153	-0.966786	37.482322	26.504004	-74.432136	72.498564	146.9307	73.46535	81.688571	0.899334	0.107143	FALSE
LV_ Sept.Wall Strain.Lt SWS %[%]	centile	34	30	30	0.882353	-0.676667	27.989097	19.79128	-55.535296	54.181963	109.717259	54.85863	77.567333	0.707239	0.066667	59	56	56	0.949153	-0.107679	40.632221	28.731319	-79.746833	79.531476	159.278308	79.639154	69.353125	1.148314	0.089286	FALSE
RV_ Sept.Wall Strain.Rt SWS %[%]	centile	34	30	30	0.882353	1.638333	36.110826	25.53421	-69.138885	72.415551	141.554436	70.777218	80.074833	0.883888	0	59	56	56	0.949153	-4.255536	35.517714	25.114816	-73.870255	65.359184	139.229438	69.614719	66.797054	1.042182	0.071429	FALSE
LV_ Basal-Lat.Wall FS.Lt Basal-Lat FS % (AC)[%]	centile	34	30	30	0.882353	-1.856	39.822554	28.158798	-79.908207	76.196207	156.104414	78.052207	27.539667	2.834174	0.033333	59	56	56	0.949153	0.650179	43.549351	30.794042	-84.70655	86.006907	170.713457	85.356728	30.650089	2.784877	0.125	FALSE
LV_ Basal-Lat.Wall FS.Lt Basal-Lat FS % (AUA)[%]	centile	34	30	30	0.882353	-2.047333	39.596287	27.998803	-79.656057	75.56139	155.217447	77.608723	27.901333	2.781542	0.033333	59	54	54	0.915254	0.590741	44.584906	31.526289	-86.795675	87.977156	174.772831	87.386415	32.525185	2.686731	0.111111	FALSE
LV_ Basal-Lat.Wall FS.Lt Basal-Lat FS % (BPD)[%]	centile	34	30	30	0.882353	-1.84	39.774159	28.124578	-79.797352	76.117352	155.914705	77.957352	28.111333	2.773165	0.033333	59	55	55	0.932203	1.880182	42.40164	29.982487	-81.227033	84.987397	166.21443	83.107215	31.353182	2.650679	0.109091	FALSE
LV_ Basal-Lat.Wall FS.Lt Basal-Lat FS % (EFW)[%]	centile	34	30	30	0.882353	-1.793667	39.413806	27.869769	-79.044725	75.457392	154.502118	77.251059	28.328833	2.726941	0.033333	59	55	55	0.932203	2.168	42.585919	30.112792	-81.300401	85.636401	166.936802	83.468401	31.586182	2.642561	0.127273	FALSE
LV_ Basal-Lat.Wall FS.Lt Basal-Lat FS % (FL)[%]	centile	34	29	29	0.852941	0.557586	38.565758	27.270109	-75.031299	76.146472	151.177771	75.588886	26.439138	2.858977	0.034483	59	55	55	0.932203	2.232545	41.406952	29.279136	-78.92508	83.390171	162.315251	81.157625	30.624455	2.650092	0.127273	FALSE
LV_ Basal-Lat.Wall FS.Lt Basal-Lat FS % (GA)[%]	centile	34	28	28	0.823529	-2.642143	39.866024	28.189536	-80.779549	75.495263	156.274813	78.137406	29.996786	2.604859	0.035714	59	51	51	0.864407	-1.958039	40.565495	28.684137	-81.466409	77.550331	159.01674	79.50837	29.39902	2.704457	0.078431	FALSE
LV_ Basal-Lat.Wall FS.Lt Basal-Lat FS % (HC)[%]	centile	34	30	30	0.882353	-1.931	39.904462	28.216715	-80.143745	76.281745	156.42549	78.212745	27.858167	2.807534	0.033333	59	55	55	0.932203	2.114909	42.352087	29.947448	-80.895181	85.124999	166.02018	83.01009	30.844	2.691288	0.109091	FALSE
RV_ Basal-Lat.Wall FS.Rt Basal-Lat FS % (AC)[%]	centile	34	30	30	0.882353	-1.876	37.705814	26.662037	-75.779396	72.027396	147.806792	73.903396	17.588	4.201922	0.133333	59	56	56	0.949153	9.186607	33.364417	23.592206	-56.207651	74.580865	130.788516	65.394258	24.452232	2.674368	0.053571	FALSE
RV_ Basal-Lat.Wall FS.Rt Basal-Lat FS % (AUA)[%]	centile	34	30	30	0.882353	-1.667333	37.288682	26.36708	-74.75315	71.418483	146.171633	73.085817	16.951667	4.311424	0.133333	59	54	54	0.915254	11.133704	31.534045	22.297937	-50.673025	72.940432	123.613457	61.806728	23.671296	2.611041	0.055556	FALSE
RV_ Basal-Lat.Wall FS.Rt Basal-Lat FS % (BPD)[%]	centile	34	30	30	0.882353	-1.706333	37.107476	26.238948	-74.436987	71.024321	145.461308	72.730654	16.6185	4.376487	0.166667	59	55	55	0.932203	9.696	32.583823	23.040243	-54.168294	73.560294	127.728588	63.864294	23.126909	2.761471	0.054545	FALSE
RV_ Basal-Lat.Wall FS.Rt Basal-Lat FS % (EFW)[%]	centile	34	30	30	0.882353	-2.011667	35.621854	25.188455	-71.830501	67.807168	139.63767	69.818835	15.8105	4.415979	0.2	59	55	55	0.932203	9.709091	32.299485	22.839185	-53.597901	73.016082	126.613983	63.306991	21.321091	2.969219	0.072727	FALSE
RV_ Basal-Lat.Wall FS.Rt Basal-Lat FS % (FL)[%]	centile	34	29	29	0.852941	-1.87931	37.66438	26.632738	-75.701495	71.942874	147.644369	73.822184	17.25069	4.279376	0.206897	59	55	55	0.932203	8.145273	31.566554	22.320925	-53.725174	70.015719	123.740893	61.870446	22.437909	2.757407	0.054545	FALSE
RV_ Basal-Lat.Wall FS.Rt Basal-Lat FS % (GA)[%]	centile	34	28	28	0.823529	-2.002143	38.459375	27.194885	-77.382518	73.378232	150.76075	75.380375	17.941429	4.20147	0.142857	59	51	51	0.864407	11.083137	32.760776	23.165367	-53.127984	75.294258	128.422242	64.211121	22.525294	2.850623	0.058824	FALSE
RV_ Basal-Lat.Wall FS.Rt Basal-Lat FS % (HC)[%]	centile	34	30	30	0.882353	-1.624	36.945411	26.124351	-74.037005	70.789005	144.826011	72.413005	16.716	4.331958	0.133333	59	55	55	0.932203	9.549455	32.332951	22.862849	-53.82313	72.922039	126.745169	63.372585	23.497273	2.697019	0.054545	FALSE
LV_ Basal-Sep.Wall FS.Lt Basal-Sep FS % (AC)[%]	centile	34	30	30	0.882353	-0.425	33.097651	23.403573	-65.296395	64.446395	129.742791	64.871395	37.5765	1.726382	0	59	56	56	0.949153	-0.130357	42.825435	30.282156	-84.06821	83.807496	167.875706	83.937853	50.352143	1.667017	0.089286	FALSE
LV_ Basal-Sep.Wall FS.Lt Basal-Sep FS % (AUA)[%]	centile	34	30	30	0.882353	-0.058667	32.70529	23.126133	-64.161036	64.043702	128.204738	64.102369	36.936667	1.735467	0	59	54	54	0.915254	-1.322037	42.501498	30.053097	-84.624972	81.980898	166.60587	83.302935	51.310278	1.623514	0.092593	FALSE
LV_ Basal-Sep.Wall FS.Lt Basal-Sep FS % (BPD)[%]	centile	34	30	30	0.882353	-0.011667	33.051291	23.370792	-64.792198	64.768864	129.561062	64.780531	36.7385	1.763287	0	59	55	55	0.932203	1.554182	40.849824	28.885188	-78.511474	81.619837	160.131311	80.065655	50.176364	1.595685	0.090909	FALSE
LV_ Basal-Sep.Wall FS.Lt Basal-Sep FS % (EFW)[%]	centile	34	30	30	0.882353	-0.101333	33.175657	23.458732	-65.125622	64.922955	130.048577	65.024289	35.596667	1.826696	0	59	55	55	0.932203	1.387091	40.56924	28.686785	-78.128619	80.902801	159.031421	79.51571	48.300273	1.646279	0.090909	FALSE
LV_ Basal-Sep.Wall FS.Lt Basal-Sep FS % (FL)[%]	centile	34	29	29	0.852941	2.192069	30.733555	21.731905	-58.045699	62.429837	120.475536	60.237768	35.267414	1.708029	0	59	55	55	0.932203	1.099636	41.348237	29.237619	-79.942908	82.14218	162.085088	81.042544	49.077636	1.651313	0.090909	FALSE
LV_ Basal-Sep.Wall FS.Lt Basal-Sep FS % (GA)[%]	centile	34	28	28	0.823529	-0.312857	34.102668	24.114228	-67.154087	66.528373	133.68246	66.84123	39.232143	1.703736	0	59	51	51	0.864407	-0.555882	43.553307	30.796839	-85.920365	84.8086	170.728965	85.364482	49.083824	1.739157	0.098039	FALSE
LV_ Basal-Sep.Wall FS.Lt Basal-Sep FS % (HC)[%]	centile	34	30	30	0.882353	0.139667	32.847653	23.226798	-64.241734	64.521067	128.762801	64.3814	36.8205	1.748521	0	59	55	55	0.932203	1.604909	40.655852	28.748029	-78.080562	81.29038	159.370942	79.685471	50.242455	1.586019	0.090909	FALSE
RV_ Basal-Sep.Wall FS.Rt Basal-Sep FS % (AC)[%]	centile	34	30	30	0.882353	2.883	42.08405	29.757917	-79.601738	85.367738	164.969477	82.484738	14.6785	5.619426	0.033333	59	56	56	0.949153	10.07875	40.353802	28.534447	-69.014701	89.172201	158.186902	79.093451	27.800982	2.844988	0.035714	FALSE
RV_ Basal-Sep.Wall FS.Rt Basal-Sep FS % (AUA)[%]	centile	34	30	30	0.882353	3.034333	41.715036	29.496985	-78.727137	84.795804	163.522941	81.76147	14.280167	5.725526	0.033333	59	54	54	0.915254	11.763148	40.563962	28.683053	-67.742217	91.268514	159.010731	79.505366	27.665278	2.873832	0.037037	FALSE
RV_ Basal-Sep.Wall FS.Rt Basal-Sep FS % (BPD)[%]	centile	34	30	30	0.882353	3.022667	41.594895	29.412032	-78.503327	84.548661	163.051988	81.525994	14.156333	5.758977	0.033333	59	55	55	0.932203	11.771455	40.014194	28.294308	-66.656366	90.199275	156.855641	78.427821	26.923545	2.912983	0.036364	FALSE
RV_ Basal-Sep.Wall FS.Rt Basal-Sep FS % (EFW)[%]	centile	34	30	30	0.882353	3.037667	41.507442	29.350194	-78.31692	84.392254	162.709174	81.354587	14.057167	5.78741	0.033333	59	55	55	0.932203	11.846364	39.785653	28.132705	-66.133517	89.826244	155.959761	77.979881	26.585364	2.933188	0.036364	FALSE
RV_ Basal-Sep.Wall FS.Rt Basal-Sep FS % (FL)[%]	centile	34	29	29	0.852941	2.537586	42.266533	29.886952	-80.304819	85.379992	165.684811	82.842406	14.611897	5.669518	0.034483	59	55	55	0.932203	10.812182	40.354109	28.534664	-68.281872	89.906236	158.188108	79.094054	27.435545	2.882904	0.036364	FALSE

parameter	qc_rule	intra_n_total	intra_n_complete	intra_n_usable	intra_usable_rate	intra_bias	intra_sd_diff	intra_sw	intra_loa_low	intra_loa_high	intra_loa_width	intra_mdc95	intra_mean	intra_rel_mdc95	intra_zero_diff_rate	inter_n_total	inter_n_complete	inter_n_usable	inter_usable_rate	inter_bias	inter_sd_diff	inter_sw	inter_loa_low	inter_loa_high	inter_loa_width	inter_mdc95	inter_mean	inter_rel_mdc95	inter_zero_diff_rate	possible_copy_forward_inter
RV_ Basal-Sep.Wall FS.Rt Basal-Sep FS % (GA)[%]	centile	34	28	28	0.823529	3.060714	43.365145	30.663788	-81.93497	88.056399	169.991369	84.995684	15.447857	5.502102	0.035714	59	51	51	0.864407	11.626863	40.293688	28.49194	-67.348766	90.602491	157.951257	78.975629	28.096373	2.810883	0.039216	FALSE
RV_ Basal-Sep.Wall FS.Rt Basal-Sep FS % (HC)[%]	centile	34	30	30	0.882353	3.029	41.687259	29.477344	-78.678028	84.736028	163.414056	81.707028	14.2235	5.744509	0.033333	59	55	55	0.932203	11.594	40.054125	28.322543	-66.912084	90.100084	157.012168	78.506084	27.099909	2.896913	0.036364	FALSE
LV_ Basal-Apical FS.Lt BASE-AP FS %[%]	centile	34	30	30	0.882353	-1.418667	35.601246	25.173883	-71.197109	68.359776	139.556885	69.778443	22.538	3.096035	0.1	59	56	56	0.949153	-0.782679	42.694748	30.189746	-84.464384	82.899027	167.363411	83.681705	31.782589	2.632942	0.142857	FALSE
RV_ Basal-Apical FS.Rt BASE-AP FS %[%]	centile	34	30	30	0.882353	0.417333	42.3159	29.92186	-82.52183	83.356497	165.878326	82.939163	14.608333	5.677524	0.166667	59	56	56	0.949153	10.919107	37.930426	26.820862	-63.424528	85.262743	148.687271	74.343635	25.445982	2.921626	0.089286	FALSE
LV_ Global Strain.Lt GLS ZS[]	zscore	34	30	24	0.705882	0.27375	2.578666	1.823392	-4.780436	5.327936	10.108372	5.054186	2.283958	2.212906	0	59	56	51	0.864407	0.023922	3.668626	2.594111	-7.166586	7.214429	14.381016	7.190508	1.809216	3.974378	0	FALSE
RV_ Global Strain.Rt GLS ZS[]	zscore	34	30	25	0.735294	0.4204	3.472431	2.45538	-6.385565	7.226365	13.611931	6.805965	3.2262	2.109592	0	59	56	53	0.898305	-0.540566	3.078094	2.176541	-6.57363	5.492498	12.066129	6.033064	2.093491	2.881821	0.017857	FALSE
RV_ FreeWall Strain.Rt FWS ZS[]	zscore	34	30	25	0.735294	0.6264	2.990011	2.114257	-5.234022	6.486822	11.720843	5.860422	2.78	2.108065	0	59	56	53	0.898305	-0.449057	2.836785	2.00591	-6.009155	5.111041	11.120196	5.560098	1.899434	2.927239	0.017857	FALSE
LV_ FreeWall Strain.Lt FWS ZS[]	zscore	34	30	27	0.794118	0.168519	2.694956	1.905622	-5.113596	5.450633	10.564229	5.282115	2.303148	2.293432	0	59	56	55	0.932203	0.232364	2.640299	1.866973	-4.942622	5.407349	10.349971	5.174986	2.130182	2.429363	0	FALSE
LV_ Sept.Wall Strain.Lt SWS ZS[]	zscore	34	30	29	0.852941	-0.041034	2.87976	2.036298	-5.685364	5.603295	11.288659	5.644329	1.946379	2.899912	0.033333	59	56	56	0.949153	0.177143	2.662298	1.882529	-5.04096	5.395246	10.436206	5.218103	1.444821	3.61159	0	FALSE
RV_ Sept.Wall Strain.Rt SWS ZS[]	zscore	34	30	29	0.852941	-0.041724	2.683496	1.897518	-5.301376	5.217928	10.519304	5.259652	1.629138	3.228488	0	59	56	56	0.949153	-0.048214	1.794717	1.269056	-3.565859	3.46943	7.035289	3.517645	0.926429	3.796995	0	FALSE
LV_ Basal-Lat.Wall FS.Lt Basal-Lat FS ZS (AC)[]	none	34	30	30	0.882353	0.572667	4.781562	3.381075	-8.799195	9.944529	18.743724	9.371862	-1.959	4.784003	0	59	56	56	0.949153	-0.379107	2.857249	2.02038	-5.979316	5.221102	11.200417	5.600209	-1.435625	3.900885	0	FALSE
LV_ Basal-Lat.Wall FS.Lt Basal-Lat FS ZS(AUA)[]	none	34	30	30	0.882353	0.563333	4.758077	3.364469	-8.762498	9.889165	18.651663	9.325831	-1.912	4.877527	0	59	54	54	0.915254	-0.382037	2.898491	2.049543	-6.06308	5.299005	11.362085	5.681042	-1.312315	4.329024	0	FALSE
LV_ Basal-Lat.Wall FS.Lt Basal-Lat FS ZS(BPD)[]	none	34	30	30	0.882353	0.580667	4.832939	3.417404	-8.891895	10.053228	18.945122	9.472561	-1.914667	4.947368	0	59	55	55	0.932203	-0.268	2.77478	1.962066	-5.706569	5.170569	10.877138	5.438569	-1.366	3.981383	0	FALSE
LV_ Basal-Lat.Wall FS.Lt Basal-Lat FS ZS (EFW)[]	none	34	30	30	0.882353	0.575	4.799814	3.393981	-8.832636	9.982636	18.815273	9.407636	-1.8855	4.989465	0	59	55	55	0.932203	-0.269455	2.758361	1.950456	-5.675843	5.136934	10.812776	5.406388	-1.335636	4.047799	0	FALSE
LV_ Basal-Lat.Wall FS.Lt Basal-Lat FS ZS(FL)[]	none	34	29	29	0.852941	0.744828	4.666204	3.299505	-8.400933	9.890588	18.29152	9.14576	-2.03069	4.503771	0	59	55	55	0.932203	-0.279636	2.747062	1.942466	-5.663877	5.104605	10.768482	5.384241	-1.424727	3.779138	0	FALSE
LV_ Basal-Lat.Wall FS.Lt Basal-Lat FS ZS(GA)[]	none	34	28	28	0.823529	0.260357	3.915108	2.768399	-7.413254	7.933969	15.347223	7.673612	-1.479107	5.188003	0	59	51	51	0.864407	-0.31	2.273137	1.607351	-4.765349	4.145349	8.910697	4.455349	-1.306569	3.409961	0	FALSE
LV_ Basal-Lat.Wall FS.Lt Basal-Lat FS ZS(HC)[]	none	34	30	30	0.882353	0.577667	4.823653	3.410838	-8.876694	10.032027	18.908721	9.454361	-1.9325	4.892295	0	59	55	55	0.932203	-0.268364	2.774182	1.961643	-5.705761	5.169034	10.874795	5.437398	-1.396909	3.892449	0	FALSE
RV_ Basal-Lat.Wall FS.Rt Basal-Lat FS ZS (AC)[]	none	34	30	30	0.882353	0.493333	3.852707	2.724276	-7.057973	8.04464	15.102613	7.551306	-2.686667	2.81066	0	59	56	56	0.949153	0.466964	2.48529	1.757365	-4.404204	5.338132	9.742336	4.871168	-1.812411	2.687673	0	FALSE
RV_ Basal-Lat.Wall FS.Rt Basal-Lat FS ZS(AUA)[]	none	34	30	30	0.882353	0.475667	3.780003	2.672866	-6.93314	7.884473	14.817613	7.408807	-2.697833	2.746206	0	59	54	54	0.915254	0.563333	2.398075	1.695695	-4.136895	5.263561	9.400456	4.700228	-1.850556	2.539901	0	FALSE
RV_ Basal-Lat.Wall FS.Rt Basal-Lat FS ZS(BPD)[]	none	34	30	30	0.882353	0.480333	3.802559	2.688816	-6.972683	7.93335	14.906033	7.453017	-2.752167	2.708054	0	59	55	55	0.932203	0.542364	2.394061	1.692857	-4.149997	5.234724	9.38472	4.69236	-1.866455	2.51405	0	FALSE
RV_ Basal-Lat.Wall FS.Rt Basal-Lat FS ZS (EFW)[]	none	34	30	30	0.882353	0.507	3.857928	2.727967	-7.054539	8.068539	15.123077	7.561539	-2.8725	2.632389	0	59	55	55	0.932203	0.545818	2.423627	1.713763	-4.20449	5.296126	9.500616	4.750308	-2.004909	2.369338	0	FALSE
RV_ Basal-Lat.Wall FS.Rt Basal-Lat FS ZS(FL)[]	none	34	29	29	0.852941	0.446207	3.876618	2.741183	-7.151964	8.044378	15.196343	7.598171	-2.736552	2.776549	0	59	55	55	0.932203	0.394727	2.402977	1.699161	-4.315108	5.104563	9.419671	4.709835	-1.919545	2.45362	0	FALSE
RV_ Basal-Lat.Wall FS.Rt Basal-Lat FS ZS(GA)[]	none	34	28	28	0.823529	0.028571	3.49673	2.472562	-6.82502	6.882163	13.707183	6.853592	-2.530714	2.708165	0	59	51	51	0.864407	0.572353	2.482867	1.755652	-4.294067	5.438773	9.73284	4.86642	-1.924804	2.528268	0	FALSE
RV_ Basal-Lat.Wall FS.Rt Basal-Lat FS ZS(HC)[]	none	34	30	30	0.882353	0.457333	3.713261	2.625672	-6.820659	7.735326	14.555985	7.277992	-2.676667	2.719051	0	59	55	55	0.932203	0.536182	2.326741	1.645254	-4.024231	5.096594	9.120825	4.560413	-1.804818	2.526799	0	FALSE
LV_ Basal-Sep.Wall FS.Lt Basal-Sep FS ZS (AC)[]	none	34	30	30	0.882353	0.441667	4.517109	3.194079	-8.411868	9.295201	17.707068	8.853534	-1.033833	8.563793	0	59	56	56	0.949153	-0.195179	2.757598	1.949916	-5.60007	5.209713	10.809783	5.404892	-0.232232	23.273659	0	FALSE
LV_ Basal-Sep.Wall FS.Lt Basal-Sep FS ZS(AUA)[]	none	34	30	30	0.882353	0.447333	4.55327	3.219648	-8.477076	9.371743	17.848819	8.924409	-1.073667	8.312086	0	59	54	54	0.915254	-0.158519	2.731805	1.931678	-5.512857	5.19582	10.708677	5.354338	-0.17963	29.807657	0	FALSE
LV_ Basal-Sep.Wall FS.Lt Basal-Sep FS ZS(BPD)[]	none	34	30	30	0.882353	0.447667	4.597078	3.250625	-8.562605	9.457939	18.020544	9.010272	-1.0945	8.232318	0	59	55	55	0.932203	-0.052364	2.605599	1.842437	-5.159338	5.054611	10.213949	5.106975	-0.230727	22.134248	0	FALSE
LV_ Basal-Sep.Wall FS.Lt Basal-Sep FS ZS (EFW)[]	none	34	30	30	0.882353	0.467	4.591176	3.246452	-8.531705	9.465705	17.99741	8.998705	-1.1725	7.674802	0	59	55	55	0.932203	-0.045273	2.594998	1.834941	-5.131469	5.040923	10.172392	5.086196	-0.353727	14.378863	0	FALSE
LV_ Basal-Sep.Wall FS.Lt Basal-Sep FS ZS(FL)[]	none	34	29	29	0.852941	0.633103	4.561463	3.225441	-8.307364	9.573571	17.880934	8.940467	-1.230345	7.266635	0	59	55	55	0.932203	-0.146	2.779371	1.965312	-5.593567	5.301567	10.895134	5.447567	-0.336818	16.173613	0	FALSE
LV_ Basal-Sep.Wall FS.Lt Basal-Sep FS ZS(GA)[]	none	34	28	28	0.823529	0.465714	4.76395	3.368621	-8.871627	9.803056	18.674683	9.337342	-0.929643	10.044009	0	59	51	51	0.864407	-0.21451	2.861865	2.023644	-5.823766	5.394746	11.218512	5.609256	-0.354706	15.813823	0	FALSE
LV_ Basal-Sep.Wall FS.Lt Basal-Sep FS ZS(HC)[]	none	34	30	30	0.882353	0.442333	4.541045	3.211004	-8.458114	9.342781	17.800895	8.900448	-1.086833	8.18934	0	59	55	55	0.932203	-0.049455	2.56772	1.815652	-5.082186	4.983277	10.065463	5.032731	-0.216909	23.202031	0	FALSE
RV_ Basal-Sep.Wall FS.Rt Basal-Sep FS ZS (AC)[]	none	34	30	30	0.882353	0.183667	4.193731	2.965416	-8.036047	8.40338	16.439427	8.219713	-2.3945	3.432747	0	59	56	56	0.949153	0.38375	2.676605	1.892646	-4.862397	5.629897	10.492293	5.246147	-1.407589	3.727044	0	FALSE
RV_ Basal-Sep.Wall FS.Rt Basal-Sep FS ZS(AUA)[]	none	34	30	30	0.882353	0.197333	4.252085	3.006678	-8.136753	8.53142	16.668173	8.334087	-2.478	3.363231	0	59	54	54	0.915254	0.507963	2.70599	1.913424	-4.795778	5.811704	10.607481	5.303741	-1.445463	3.669233	0	FALSE
RV_ Basal-Sep.Wall FS.Rt Basal-Sep FS ZS(BPD)[]	none	34	30	30	0.882353	0.200667	4.260088	3.012337	-8.149106	8.550439	16.699546	8.349773	-2.518	3.316034	0	59	55	55	0.932203	0.474909	2.722314	1.924967	-4.860826	5.810644	10.67147	5.335735	-1.487455	3.587158	0	FALSE
RV_ Basal-Sep.Wall FS.Rt Basal-Sep FS ZS (EFW)[]	none	34	30	30	0.882353	0.209	4.271671	3.020528	-8.163476	8.581476	16.744951	8.372476	-2.5145	3.329678	0	59	55	55	0.932203	0.471636	2.698147	1.907878	-4.816732	5.760005	10.576738	5.288369	-1.499455	3.526862	0	FALSE
RV_ Basal-Sep.Wall FS.Rt Basal-Sep FS ZS(FL)[]	none	34	29	29	0.852941	0.047931	4.227239	2.98911	-8.237458	8.33332	16.570778	8.285389	-2.406724	3.4426	0	59	55	55	0.932203	0.406364	2.703568	1.911711	-4.892629	5.705357	10.597986	5.298993	-1.450273	3.653791	0	FALSE
RV_ Basal-Sep.Wall FS.Rt Basal-Sep FS ZS(GA)[]	none	34	28	28	0.823529	-0.084643	4.235798	2.995162	-8.386808	8.217522	16.60433	8.302165	-2.27625	3.647299	0	59	51	51	0.864407	0.533333	2.724504	1.926515	-4.806695	5.873361	10.680056	5.340028	-1.421961	3.755398	0	FALSE
RV_ Basal-Sep.Wall FS.Rt Basal-Sep FS ZS(HC)[]	none	34	30	30	0.882353	0.199333	4.27234	3.021001	-8.174453	8.57312	16.747573	8.373786	-2.509333	3.337056	0	59	55	55	0.932203	0.474909	2.71664	1.920955	-4.849705	5.799523	10.649229	5.324614	-1.475818	3.607907	0	FALSE
LV_ Basal-Apical FS.Lt BASE-AP FS ZS[]	zscore	34	30	28	0.823529	-0.611071	2.987363	2.112384	-6.466302	5.244159	11.710462	5.855231	-2.253036	2.598818	0	59	56	55	0.932203	-0.305636	3.381576	2.391135	-6.933525	6.322252	13.255777	6.627889	-1.617	4.09888	0	FALSE
RV_ Basal-Apical FS.Rt BASE-AP FS ZS[]	zscore	34	30	25	0.735294	-0.676	3.718837	2.629615	-7.964921	6.612921	14.577842	7.288921	-2.6608	2.739372	0	59	56	53	0.898305	0.496038	2.780803	1.966324	-4.954335	5.946411	10.900746	5.450373	-1.68217	3.240085	0	FALSE
LV_ ED Area.Lt ED Area % (AC)[%]	centile	34	30	30	0.882353	-3.566333	32.195871	22.765919	-66.670241	59.537575	126.207816	63.103908	39.258833	1.607381	0.033333	59	56	56	0.949153	-7.010536	26.833852	18.974398	-59.604885	45.583814	105.188698	52.594349	52.104018	1.009411	0	FALSE
LV_ ED Area.Lt ED Area % (AUA)[%]	centile	34	30	30	0.882353	-3.467667	29.795134	21.068341	-61.86613	54.930796	116.796926	58.398463	25.882167	2.25632	0.033333	59	54	54	0.915254	-8.104815	28.529577	20.173458	-64.022787	47.813157	111.835944	55.917972	39.657222	1.410032	0	FALSE
LV_ ED Area.Lt ED Area % (BPD)[%]	centile	34	30	30	0.882353	-2.522667	25.797714	18.241738	-53.086185	48.040852	101.127037	50.563519	23.703667	2.133152	0.033333	59	55	55	0.932203	-6.422182	26.714092	18.889715	-58.781802	45.937438	104.719239	52.35962	36.732	1.42545	0	FALSE
LV_ ED Area.Lt ED Area % (EFW)[%]	centile	34	30	30	0.882353	-3.723	28.820956	20.379493	-60.212074	52.766074	112.978148	56.489074	27.596167	2.04699	0.033333	59	55	55	0.932203	-6.261818	26.039127	18.412444	-57.298508	44.774872	102.07338	51.03669	39.691273	1.285842	0	FALSE
LV_ ED Area.Lt ED Area % (FL)[%]	centile	34	29	29	0.852941	-3.38	35.018339	24.761705	-72.015945	65.255945	137.271889	68.635945	31.865862	2.153902	0.034483	59	55	55	0.932203	-6.571818	27.285533	19.293786	-60.051463	46.907827	106.95929	53.479645	43.784273	1.221435	0	FALSE
LV_ ED Area.Lt ED Area % (GA)[%]	centile	34	28	28	0.823529	-8.981071	26.784954	18.939822	-61.479581	43.517438	104.997018	52.498509	26.17875	2.005386	0.035714	59	51	51	0.864407	-6.208431	29.39213	20.783374	-63.817006	51.400143	115.217149	57.608575	40.019314	1.439519	0	FALSE
LV_ ED Area.Lt ED Area % (HC)[%]	centile	34	30	30	0.882353	-3.144333	28.356591	20.051138	-58.723252	52.434585	111.157837	55.578919	23.1535	2.400454	0.033333	59	55	55	0.932203	-7.492545	29.807918	21.077381	-65.916065	50.930974	116.847039	58.42352	38.326636	1.524358	0	FALSE
LV_ ED Length.Lt ED Length % (AC)[%]	centile	34	30	30	0.882353	-1.431	22.878704	16.177687	-46.27326	43.41126	89.68452	44.84226	13.923167	3.220694	0	59	56	56	0.949153	-6.108929	21.928048	15.505471	-49.087902	36.870045	85.957947	42.978973	21.448214	2.003849	0.035714	FALSE
LV_ ED Length.Lt ED Length % (AUA)[%]	centile	34	30	30	0.882353	-1.361	20.364583	14.399935	-41.275582	38.553582	79.829164	39.914582	11.879833	3.35986	0	59	54	54	0.915254	-6.713148	21.106154	14.924305	-48.081211	34.654914	82.736125	41.368062	21.048056	1.96541	0.037037	FALSE
LV_ ED Length.Lt ED Length % (BPD)[%]	centile	34	30	30	0.882353	-0.764333	19.949962	14.106753	-39.866259	38.337592	78.203851	39.101926	11.550167	3.385399	0	59	55	55	0.932203	-5.915636	18.573914	13.13374	-42.320507	30.489235	72.809742	36.404871	19.277091	1.888504	0.036364	FALSE
LV_ ED Length.Lt ED Length % (EFW)[%]	centile	34	30	30	0.882353	-1.383667	20.470964	14.475157	-41.506756	38.739423	80.246178	40.123089	13.324167	3.011302	0	59	55	55	0.932203	-6.036182	20.099564	14.212538	-45.431327	33.358963	78.79029	39.395145	21.233364	1.855342	0.036364	FALSE
LV_ ED Length.Lt ED Length % (FL)[%]	centile	34	29	29	0.852941	-1.475862	20.279192	14.339554	-41.223079	38.271355	79.494434	39.747217	12.391034	3.20774	0	59	55	55	0.932203	-5.872545	20.943893	14.809569	-46.922575	35.177485	82.10006	41.05003	20.359727	2.016237	0.036364	FALSE
LV_ ED Length.Lt ED Length % (GA)[%]	centile	34	28	28	0.823529	-1.697857	19.720407	13.944434	-40.349856	36.954141	77.303997	38.651999	11.384286	3.395206	0	59	51	51	0.864407	-4.166471	17.279194	12.218235	-38.03369	29.700749	67.73444	33.86722	18.740294	1.807187	0.039216	FALSE
LV_ ED Length.Lt ED Length % (HC)[%]	centile	34	30	30	0.882353	-0.767667	19.965113	14.117467	-39.899288	38.363954	78.263242	39.131621	10.851833	3.605992	0	59	55	55	0.932203	-6.183273	20.290179	14.347323	-45.952023	33.585477	79.5375	39.76875	19.984364	1.989993	0.036364	FALSE
RV_ ED Area.Rt ED Area % (AC)[%]	centile	34	30	30	0.882353	-6.261333	32.884707	23.252999	-70.715359	58.192692	128.90805	64.454025	47.171	1.366391	0.066667	59	56	56	0.949153	-0.945714	26.697556	18.878023	-53.272924	51.381495	104.654419	52.32721	63.776429	0.820479	0.107143	FALSE
RV_ ED Area.Rt ED Area % (AUA)[%]	centile	34	30	30	0.882353	-10.203	35.249871	24.925423	-79.292746	58.886746	138.179493	69.089746	32.644167	2.11645	0.066667	59	54	54	0.915254	-1.73963	26.638424	18.83621	-53.95094	50.471681	104.422621	52.211311	56.957222	0.916676	0.055556	FALSE
RV_ ED Area.Rt ED Area % (BPD)[%]	centile	34	30	30	0.882353	-7.325667	32.412273	22.918938	-70.853721	56.202388	127.056109	63.528055	27.7365	2.290414	0.033333	59	55	55	0.932203	-3.766182	27.399627	19.374462	-57.469451	49.937087	107.406538	53.703269	48.951091	1.09708	0.072727	FALSE
RV_ ED Area.Rt ED Area % (EFW)[%]	centile	34	30	30	0.882353	-7.394333	31.715182	22.42602	-69.556089	54.767423	124.323512	62.161756	37.192833	1.671337	0.066667	59	55	55	0.932203	-2.738727	22.720264	16.065653	-47.270446	41.792991	89.063437	44.531718	55.541364	0.801776	0.054545	FALSE
RV_ ED Area.Rt ED Area % (FL)[%]	centile	34	29	29	0.852941	-8.166552	35.58064	25.159312	-77.904607	61.571503	139.47611	69.738055	31.234655	2.232714	0.034483	59	55	55	0.932203	-3.970909	28.172507	19.920971	-59.189023	51.247205	110.436228	55.218114	51.201818	1.07844	0.054545	FALSE
RV_ ED Area.Rt ED Area % (GA)[%]	centile	34	28	28	0.823529	-12.311071	35.472943	25.083159	-81.83804	57.215898	139.053938	69.526969	30.459107	2.282633	0.071429	59	51	51	0.864407	-2.991373	29.438763	20.816349	-60.691348	54.708603	115.399952	57.699976	50.596275	1.1404	0.058824	FALSE
RV_ ED Area.Rt ED Area % (HC)[%]	centile	34	30	30	0.882353	-10.522667	36.862569	26.065772	-82.773301	61.727968	144.501269	72.250635	27.172333	2.658978	0.033333	59	55	55	0.932203	-2.142182	28.551098	20.188675	-58.102333	53.81797	111.920303	55.960152	52.350909	1.068943	0.109091	FALSE
RV_ ED Length.Rt ED Length % (AC)[%]	centile	34	30	30	0.882353	-1.913333	24.930928	17.628828	-50.777952	46.951285	97.729237	48.864618	18.585667	2.629156	0.033333	59	56	56	0.949153	-6.560893	25.629836	18.123031	-56.795371	43.673586	100.468957	50.234478	29.233125	1.718409	0.053571	FALSE
RV_ ED Length.Rt ED Length % (AUA)[%]	centile	34	30	30	0.882353	-1.450333	22.885356	16.182391	-46.305631	43.404965	89.710596	44.855298	15.303833	2.930984	0.033333	59	54	54	0.915254	-5.341481	25.315586	17.900823	-54.96003	44.277067	99.237098	49.618549	25.387778	1.954427	0.055556	FALSE
RV_ ED Length.Rt ED Length % (BPD)[%]	centile	34	30	30	0.882353	-1.076667	21.406469	15.136659	-43.033345	40.880012	83.913357	41.956678	14.566333	2.880387	0.033333	59	55	55	0.932203	-5.748364	24.700631	17.465983	-54.1616	42.664872	96.826472	48.413236	23.903273	2.025381	0.054545	FALSE
RV_ ED Length.Rt ED Length % (EFW)[%]	centile	34	30	30	0.882353	-1.449667	23.016464	16.275097	-46.561935	43.662602	90.224537	45.112269	16.1575	2.792033	0.033333	59	55	55	0.932203	-5.517818	24.570286	17.373816	-53.675578	42.639942	96.315521	48.15776	25.512	1.887651	0.036364	FALSE
RV_ ED Length.Rt ED Length % (FL)[%]	centile	34	29	29	0.852941	-1.673448	23.865584	16.875517	-48.449994	45.103097	93.553091	46.776546	16.184655	2.890179	0.034483	59	55	55	0.932203	-4.580727	23.707006	16.763385	-51.04646	41.885005	92.931465	46.465732	24.831455	1.871245	0.036364	FALSE
RV_ ED Length.Rt ED Length % (GA)[%]	centile	34	28	28	0.823529	-1.953571	23.129645	16.355129	-47.287675	43.380532	90.668207	45.334103	16.026071	2.828772	0.035714	59	51	51	0.864407	-5.308235	23.818868	16.842483	-51.993216	41.376745	93.369961	46.68498	25.719608	1.815151	0.039216	FALSE
RV_ ED Length.Rt ED Length % (HC)[%]	centile	34	30	30	0.882353	-1.317	22.846531	16.154937	-46.0962	43.4622	89.5584	44.7792	14.392167	3.111359	0.033333	59	55	55	0.932203	-6.044909	25.365535	17.936142	-55.761357	43.671539	99.432896	49.716448	24.587	2.022062	0.054545	FALSE
LV_ ED Area.Lt ED Area ZS (AC)[]	none	34	30	30	0.882353	-0.109333	1.272399	0.899722	-2.603236	2.384569	4.987805	2.493902	-0.383333	6.505832	0.033333	59	56	56	0.949153	-0.265357	1.105385	0.781625	-2.431912	1.901198	4.33311	2.166555	0.162143	13.362012	0	FALSE
LV_ ED Area.Lt ED Area ZS(AUA)[]	none	34	30	30	0.882353	-0.085	1.242436	0.878535	-2.520174	2.350174	4.870348	2.435174	-0.9445	2.578268	0.033333	59	54	54	0.915254	-0.312407	1.102642	0.779685	-2.473585	1.84877	4.322355	2.161178	-0.451389	4.78784	0	FALSE
LV_ ED Area.Lt ED Area ZS(BPD)[]	none	34	30	30	0.882353	-0.067667	1.165142	0.82388	-2.351345	2.216012	4.567358	2.283679	-1.010833	2.259204	0.033333	59	55	55	0.932203	-0.235818	1.034578	0.731557	-2.26359	1.791954	4.055544	2.027772	-0.568273	3.568308	0	FALSE
LV_ ED Area.Lt ED Area ZS (EFW)[]	none	34	30	30	0.882353	-0.077	1.1187	0.791041	-2.269653	2.115653	4.385306	2.192653	-0.818167	2.679959	0.033333	59	55	55	0.932203	-0.211273	0.9541	0.674651	-2.08131	1.658764	3.740074	1.870037	-0.397273	4.707186	0	FALSE
LV_ ED Area.Lt ED Area ZS(FL)[]	none	34	29	29	0.852941	-0.074138	1.280476	0.905433	-2.58387	2.435594	5.019465	2.509732	-0.723276	3.469952	0.034483	59	55	55	0.932203	-0.242727	1.106435	0.782368	-2.411339	1.925885	4.337224	2.168612	-0.256091	8.468134	0	FALSE
LV_ ED Area.Lt ED Area ZS(GA)[]	none	34	28	28	0.823529	-0.218929	1.258825	0.890123	-2.686225	2.248368	4.934593	2.467296	-0.996964	2.474809	0.035714	59	51	51	0.864407	-0.213922	1.120436	0.792268	-2.409976	1.982133	4.392108	2.196054	-0.468529	4.687121	0	FALSE
LV_ ED Area.Lt ED Area ZS(HC)[]	none	34	30	30	0.882353	-0.064667	1.286427	0.909641	-2.586064	2.45673	5.042794	2.521397	-1.112667	2.266085	0.033333	59	55	55	0.932203	-0.285091	1.232821	0.871736	-2.70142	2.131238	4.832658	2.416329	-0.556727	4.340239	0	FALSE
LV_ ED Length.Lt ED Length ZS (AC)[]	none	34	30	30	0.882353	-0.144	1.539634	1.088686	-3.161683	2.873683	6.035366	3.017683	-1.811	1.666307	0	59	56	56	0.949153	-0.382679	1.251426	0.884892	-2.835474	2.070117	4.90559	2.452795	-1.382946	1.773601	0.035714	FALSE
LV_ ED Length.Lt ED Length ZS(AUA)[]	none	34	30	30	0.882353	-0.141667	1.425855	1.008232	-2.936343	2.653009	5.589352	2.794676	-1.8145	1.540191	0	59	54	54	0.915254	-0.354074	1.142573	0.807921	-2.593518	1.88537	4.478887	2.239444	-1.344444	1.665702	0.037037	FALSE
LV_ ED Length.Lt ED Length ZS(BPD)[]	none	34	30	30	0.882353	-0.133333	1.404543	0.993162	-2.886237	2.61957	5.505807	2.752904	-1.85	1.488056	0	59	55	55	0.932203	-0.346545	1.121084	0.792726	-2.543871	1.85078	4.394651	2.197326	-1.442545	1.523228	0.036364	FALSE
LV_ ED Length.Lt ED Length ZS (EFW)[]	none	34	30	30	0.882353	-0.136667	1.291501	0.913229	-2.668009	2.394675	5.062684	2.531342	-1.596667	1.585392	0	59	55	55	0.932203	-0.309818	1.010738	0.714699	-2.290864	1.671228	3.962092	1.981046	-1.228364	1.612752	0.036364	FALSE
LV_ ED Length.Lt ED Length ZS(FL)[]	none	34	29	29	0.852941	-0.124483	1.404614	0.993212	-2.877526	2.62856	5.506086	2.753043	-1.697414	1.621904	0	59	55	55	0.932203	-0.293091	1.064705	0.75286	-2.379912	1.79373	4.173642	2.086821	-1.332727	1.565827	0.036364	FALSE
LV_ ED Length.Lt ED Length ZS(GA)[]	none	34	28	28	0.823529	-0.187857	1.439077	1.017581	-3.008449	2.632734	5.641183	2.820591	-1.848214	1.526117	0	59	51	51	0.864407	-0.30451	1.114306	0.787933	-2.488549	1.879529	4.368078	2.184039	-1.465784	1.490014	0.039216	FALSE
LV_ ED Length.Lt ED Length ZS(HC)[]	none	34	30	30	0.882353	-0.134333	1.489035	1.052907	-3.052842	2.784175	5.837017	2.918509	-1.9655	1.484868	0	59	55	55	0.932203	-0.375455	1.221871	0.863994	-2.770322	2.019413	4.789736	2.394868	-1.493727	1.603283	0.036364	FALSE
RV_ ED Area.Rt ED Area ZS (AC)[]	none	34	30	30	0.882353	-0.432333	1.657563	1.172074	-3.681157	2.81649	6.497647	3.248823	-0.019167	169.503824	0.033333	59	56	56	0.949153	-0.246607	1.471531	1.04053	-3.130809	2.637594	5.768403	2.884202	1.002054	2.878291	0.035714	FALSE
RV_ ED Area.Rt ED Area ZS(AUA)[]	none	34	30	30	0.882353	-0.363	1.528922	1.081111	-3.359686	2.633686	5.993372	2.996686	-0.7345	4.0799	0.033333	59	54	54	0.915254	-0.175556	1.380684	0.976291	-2.881697	2.530586	5.412283	2.706141	0.316111	8.560728	0.037037	FALSE
RV_ ED Area.Rt ED Area ZS(BPD)[]	none	34	30	30	0.882353	-0.282	1.366822	0.966489	-2.960971	2.396971	5.357943	2.678971	-0.941	2.846941	0.033333	59	55	55	0.932203	-0.159091	1.284098	0.907995	-2.675924	2.357742	5.033665	2.516833	-0.079909	31.496199	0.036364	FALSE
RV_ ED Area.Rt ED Area ZS (EFW)[]	none	34	30	30	0.882353	-0.258667	1.300636	0.919688	-2.807913	2.290579	5.098492	2.549246	-0.468333	5.44323	0.033333	59	55	55	0.932203	-0.093455	1.029599	0.728037	-2.111469	1.92456	4.03603	2.018015	0.269636	7.484209	0.036364	FALSE
RV_ ED Area.Rt ED Area ZS(FL)[]	none	34	29	29	0.852941	-0.242759	1.452048	1.026753	-3.088772	2.603255	5.692026	2.846013	-0.81069	3.510608	0.034483	59	55	55	0.932203	-0.170545	1.238683	0.875881	-2.598364	2.257273	4.855636	2.427818	0.059636	40.710364	0.036364	FALSE
RV_ ED Area.Rt ED Area ZS(GA)[]	none	34	28	28	0.823529	-0.470357	1.519787	1.074652	-3.449139	2.508425	5.957564	2.978782	-0.878393	3.391173	0.035714	59	51	51	0.864407	-0.120392	1.402411	0.991654	-2.869117	2.628333	5.49745	2.748725	0.127255	21.60015	0.039216	FALSE
RV_ ED Area.Rt ED Area ZS(HC)[]	none	34	30	30	0.882353	-0.378	1.752758	1.239387	-3.813406	3.057406	6.870812	3.435406	-1.291333	2.660356	0.033333	59	55	55	0.932203	-0.359273	2.028516	1.434377	-4.335164	3.616619	7.951783	3.975891	0.061818	64.315889	0.036364	FALSE
RV_ ED Length.Rt ED Length ZS (AC)[]	none	34	30	30	0.882353	-0.378	1.188176	0.840167	-2.706824	1.950824	4.657649	2.328824	-1.501667	1.550826	0.033333	59	56	56	0.949153	-0.224643	1.344183	0.950481	-2.859242	2.409957	5.269199	2.634599	-0.962321	2.737754	0.035714	FALSE
RV_ ED Length.Rt ED Length ZS(AUA)[]	none	34	30	30	0.882353	-0.391333	1.220415	0.862963	-2.783346	2.000679	4.784025	2.392013	-1.738	1.376302	0.033333	59	54	54	0.915254	-0.184444	1.399019	0.989256	-2.926523	2.557634	5.484156	2.742078	-1.166852	2.34998	0.037037	FALSE

parameter	qc_rule	intra_n_total	intra_n_complete	intra_n_usable	intra_usable_rate	intra_bias	intra_sd_diff	intra_sw	intra_loa_low	intra_loa_high	intra_loa_width	intra_mdc95	intra_mean	intra_rel_mdc95	intra_zero_diff_rate	inter_n_total	inter_n_complete	inter_n_usable	inter_usable_rate	inter_bias	inter_sd_diff	inter_sw	inter_loa_low	inter_loa_high	inter_loa_width	inter_mdc95	inter_mean	inter_rel_mdc95	inter_zero_diff_rate	possible_copy_forward_inter
RV_ ED Length.Rt ED Length ZS(BPD)[]	none	34	30	30	0.882353	-0.399667	1.218689	0.861743	-2.788297	1.988964	4.777262	2.388631	-1.825167	1.308719	0.033333	59	55	55	0.932203	-0.233273	1.439823	1.018108	-3.055325	2.58878	5.644105	2.822052	-1.273727	2.215586	0.036364	FALSE
RV_ ED Length.Rt ED Length ZS (EFW)[]	none	34	30	30	0.882353	-0.358667	1.193834	0.844168	-2.698581	1.981248	4.679829	2.339915	-1.646	1.421576	0.033333	59	55	55	0.932203	-0.204545	1.33	0.940452	-2.811345	2.402254	5.213599	2.6068	-1.133182	2.300425	0.036364	FALSE
RV_ ED Length.Rt ED Length ZS(FL)[]	none	34	29	29	0.852941	-0.34069	1.192096	0.842939	-2.677197	1.995818	4.673015	2.336508	-1.658966	1.408412	0.034483	59	55	55	0.932203	-0.180909	1.292791	0.914141	-2.714779	2.352961	5.06774	2.53387	-1.155364	2.193137	0.036364	FALSE
RV_ ED Length.Rt ED Length ZS(GA)[]	none	34	28	28	0.823529	-0.428571	1.165195	0.823917	-2.712354	1.855211	4.567564	2.283782	-1.638214	1.394068	0.035714	59	51	51	0.864407	-0.182157	1.327493	0.938679	-2.784043	2.419729	5.203772	2.601886	-1.088137	2.391138	0.039216	FALSE
RV_ ED Length.Rt ED Length ZS(HC)[]	none	34	30	30	0.882353	-0.393333	1.240148	0.876917	-2.824024	2.037357	4.861381	2.430691	-1.836667	1.323425	0.033333	59	55	55	0.932203	-0.245091	1.457998	1.03096	-3.102767	2.612586	5.715353	2.857676	-1.252364	2.281826	0.036364	FALSE
4CV Circ (2Dist)[mm]	positive	34	30	30	0.882353	0.257667	2.19268	1.550459	-4.039986	4.55532	8.595306	4.297653	76.210833	0.056392	0.9	59	56	56	0.949153	0	0	0	0	0	0	0	75.403214	0	1	TRUE
4CV Circ (2Dist).4CV C % (AC)[%]	centile	34	30	30	0.882353	-0.356333	1.11317	0.78713	-2.538147	1.82548	4.363628	2.181814	47.5305	0.045903	0.9	59	56	56	0.949153	0	0	0	0	0	0	0	49.7875	0	1	TRUE
4CV Circ (2Dist).4CV C % (AUA)[%]	centile	34	30	30	0.882353	-1.433667	5.725463	4.048514	-12.655574	9.788241	22.443815	11.221908	38.182833	0.293899	0.9	59	54	54	0.915254	-0.02037	0.149691	0.105848	-0.313765	0.273024	0.586789	0.293394	47.878704	0.006128	0.981481	FALSE
4CV Circ (2Dist).4CV C % (BPD)[%]	centile	34	30	30	0.882353	-1.520333	6.390768	4.518956	-14.046239	11.005573	25.051812	12.525906	36.151833	0.346481	0.9	59	55	55	0.932203	0	0	0	0	0	0	0	42.523091	0	1	TRUE
4CV Circ (2Dist).4CV C % (EFW)[%]	centile	34	30	30	0.882353	-1.363333	4.545386	3.214073	-10.27229	7.545623	17.817913	8.908956	39.391667	0.226163	0.9	59	55	55	0.932203	0	0	0	0	0	0	0	44.799636	0	1	TRUE
4CV Circ (2Dist).4CV C % (FL)[%]	centile	34	29	29	0.852941	-2.087931	8.455995	5.979291	-18.661681	14.485819	33.147501	16.57375	38.008793	0.43605	0.896552	59	56	56	0.949153	0	0	0	0	0	0	0	45.059821	0	1	TRUE
4CV Circ (2Dist).4CV C % (GA)[%]	centile	34	28	28	0.823529	-0.823214	3.870891	2.737134	-8.410161	6.763733	15.173894	7.586947	34.69125	0.218699	0.928571	59	51	51	0.864407	0	0	0	0	0	0	0	38.368824	0	1	TRUE
4CV Circ (2Dist).4CV C % (HC)[%]	centile	34	30	30	0.882353	-1.025333	3.937224	2.784038	-8.742293	6.691627	15.43392	7.71696	34.745667	0.222098	0.9	59	55	55	0.932203	0	0	0	0	0	0	0	41.788364	0	1	TRUE
4CV Circ (2Dist).4CV C ZS (AC)[]	none	34	30	30	0.882353	-0.009333	0.029235	0.020673	-0.066635	0.047968	0.114603	0.057302	-0.062333	0.919276	0.9	59	56	56	0.949153	0	0	0	0	0	0	0	-0.005	0	1	TRUE
4CV Circ (2Dist).4CV C ZS(AUA)[]	none	34	30	30	0.882353	-0.076	0.267383	0.189068	-0.600071	0.448071	1.048142	0.524071	-0.487333	1.075385	0.9	59	54	54	0.915254	-0.006481	0.047629	0.033679	-0.099834	0.086871	0.186706	0.093353	-0.065093	1.434154	0.981481	FALSE
4CV Circ (2Dist).4CV C ZS(BPD)[]	none	34	30	30	0.882353	-0.062	0.223304	0.1579	-0.499677	0.375677	0.875353	0.437677	-0.509333	0.859313	0.9	59	55	55	0.932203	0	0	0	0	0	0	0	-0.251455	0	1	TRUE
4CV Circ (2Dist).4CV C ZS (EFW)[]	none	34	30	30	0.882353	-0.052667	0.172725	0.122135	-0.391208	0.285875	0.677083	0.338542	-0.380667	0.889339	0.9	59	55	55	0.932203	0	0	0	0	0	0	0	-0.145273	0	1	TRUE
4CV Circ (2Dist).4CV C ZS(FL)[]	none	34	29	29	0.852941	-0.090345	0.347927	0.246022	-0.772282	0.591593	1.363875	0.681938	-0.435517	1.565811	0.896552	59	56	56	0.949153	0	0	0	0	0	0	0	-0.18375	0	1	TRUE
4CV Circ (2Dist).4CV C ZS(GA)[]	none	34	28	28	0.823529	-0.040357	0.148236	0.104819	-0.3309	0.250186	0.581086	0.290543	-0.650179	0.446866	0.928571	59	51	51	0.864407	0	0	0	0	0	0	0	-0.430392	0	1	TRUE
4CV Circ (2Dist).4CV C ZS(HC)[]	none	34	30	30	0.882353	-0.053	0.167562	0.118484	-0.381421	0.275421	0.656842	0.328421	-0.643833	0.510102	0.9	59	55	55	0.932203	0	0	0	0	0	0	0	-0.303091	0	1	TRUE

Supplementary File S1

CopyForwardFlags: Parameters with 100% zero differences in Inter-operator complete pairs

parameter	inter_n_complete	inter_zero_diff_rate
4CV Trv. Width ED[mm]	56	1
4CV Length ED[mm]	56	1
4CV Area (2Dist)[mm¸]	56	1
4CV Trv. Width ED.4CV TW ED % (AC)[%]	56	1
4CV Trv. Width ED.4CV TW ED % (BPD)[%]	55	1
4CV Trv. Width ED.4CV TW ED % (EFW)[%]	55	1
4CV Trv. Width ED.4CV TW ED % (FL)[%]	56	1
4CV Trv. Width ED.4CV TW ED % (GA)[%]	51	1
4CV Trv. Width ED.4CV TW ED % (HC)[%]	55	1
4CV Length ED.4CV L ED % (AC)[%]	56	1
4CV Length ED.4CV L ED % (BPD)[%]	55	1
4CV Length ED.4CV L ED % (EFW)[%]	55	1
4CV Length ED.4CV L ED % (FL)[%]	56	1
4CV Length ED.4CV L ED % (GA)[%]	51	1
4CV Length ED.4CV L ED % (HC)[%]	55	1
4CV Area (2Dist).4CV A % (AC)[%]	56	1
4CV Area (2Dist).4CV A % (BPD)[%]	55	1
4CV Area (2Dist).4CV A % (EFW)[%]	55	1
4CV Area (2Dist).4CV A % (FL)[%]	56	1
4CV Area (2Dist).4CV A % (HC)[%]	55	1
4CV Trv. Width ED.4CV TW ED ZS (AC)[]	56	1
4CV Trv. Width ED.4CV TW ED ZS(BPD)[]	55	1
4CV Trv. Width ED.4CV TW ED ZS (EFW)[]	55	1
4CV Trv. Width ED.4CV TW ED ZS(FL)[]	56	1

parameter	inter_n_complete	inter_zero_diff_rate
4CV Trv. Width ED.4CV TW ED ZS(GA)[]	51	1
4CV Trv. Width ED.4CV TW ED ZS(HC)[]	55	1
4CV Length ED.4CV L ED ZS (AC)[]	56	1
4CV Length ED.4CV L ED ZS(BPD)[]	55	1
4CV Length ED.4CV L ED ZS (EFW)[]	55	1
4CV Length ED.4CV L ED ZS(FL)[]	56	1
4CV Length ED.4CV L ED ZS(GA)[]	51	1
4CV Length ED.4CV L ED ZS(HC)[]	55	1
4CV Area (2Dist).4CV A ZS (AC)[]	56	1
4CV Area (2Dist).4CV A ZS(BPD)[]	55	1
4CV Area (2Dist).4CV A ZS (EFW)[]	55	1
4CV Area (2Dist).4CV A ZS(FL)[]	56	1
4CV Area (2Dist).4CV A ZS(HC)[]	55	1
4CV Circ (2Dist)[mm]	56	1
4CV Circ (2Dist).4CV C % (AC)[%]	56	1
4CV Circ (2Dist).4CV C % (BPD)[%]	55	1
4CV Circ (2Dist).4CV C % (EFW)[%]	55	1
4CV Circ (2Dist).4CV C % (FL)[%]	56	1
4CV Circ (2Dist).4CV C % (GA)[%]	51	1
4CV Circ (2Dist).4CV C % (HC)[%]	55	1
4CV Circ (2Dist).4CV C ZS (AC)[]	56	1
4CV Circ (2Dist).4CV C ZS(BPD)[]	55	1
4CV Circ (2Dist).4CV C ZS (EFW)[]	55	1
4CV Circ (2Dist).4CV C ZS(FL)[]	56	1
4CV Circ (2Dist).4CV C ZS(GA)[]	51	1
4CV Circ (2Dist).4CV C ZS(HC)[]	55	1

Supplementary File S2. Quality-control rules and preprocessing

Dataset and pairing. All outputs were exported with paired columns ending in "_1" and "_2", representing two repeat analyses performed on the same cine-loop. No biological variability is expected between repeats.

Groups. The first 34 rows were treated as intra-operator repeats (same operator repeated the tracing). The remaining 59 rows were treated as inter-operator repeats (two operators traced independently).

Excluded outputs. All 4CV GSI-related outputs were excluded (4CV GSI raw, centile, and Z-score) due to known parameter-specific export behavior and non-comparability across tracing instances.

Definitions

- Difference: Δ = value_2 − value_1.
- Bias: mean(Δ).
- SD of differences: SD(diff) = standard deviation of Δ.
- Within-subject SD: Sw = SD(diff) / √2.
- Bland–Altman limits of agreement: 95% LoA = bias ± 1.96 × SD(diff).
- Minimal detectable change: MDC95 = 1.96 × SD(diff).
- Usable pair: A complete pair meeting Post-export numerical plausibility limits defined below.

  For reporting of non-usable pairs, observations were separated into (1) missing/incomplete paired output and (2) complete paired output excluded by numerical QC. The exported dataset did not contain reason codes for missing values; therefore, missing/incomplete outputs could not be retrospectively subdivided into acquisition failure, inadequate tracking, incomplete analysis, or software/export failure.

Post-export numerical plausibility limits

QC limits were applied uniformly using rule-based parsing of variable names. The intent was to remove obviously implausible values and prevent inflation/deflation of decision limits by non-physiologic exports.

These numerical rules were applied after completion of FetalHQ image analysis and were intended only to identify missing or physiologically/logically implausible exported values; they did not replace technical assessment of image or tracking quality.

QC rule	Name pattern (examples)	Accepted range / constraint
Centile outputs	"% (GA)[%]", "% (AC)[%]",	0–100

	"%[%]", "Centiles[%]"	
Z-scores	"ZS[]"	−10 to +10
Percent scale (EF/FRAC etc.)	"EF[%]", "Frac. Area change[%]", "FRAC"	0–100
Raw strain	"Global Strain[%]" (without "% (…)[%]" and without "ZS[]")	−60% to +10%
TAPSE (mm)	"TAPSE … [mm]"	>=0 and ≤10 mm
Physical measures (volumes/lengths/flow)	[ml], [ml/min], [cm], [mm], [g]	>0
Doppler indices (if present)	PI, RI, S/D	>0

Threshold-instability analysis

For normalized outputs (centiles and Z-scores), binary classification was assigned at standard thresholds and compared between repeats (flip = discordant classification). Cohen's kappa was computed as an agreement measure beyond chance.

Centiles: thresholds <5 and >95. Z-scores: two-sided thresholds |Z|>1.645 and |Z|>2.0; additionally, one-sided thresholds Z>2 and Z<−2 were reported for completeness.

Grey-zone definition

Grey-zone around a diagnostic threshold was defined as ±(MDC95/2) expressed in the same scale as the threshold (centile or Z-score). The proportion of cases falling within the grey-zone was calculated using the mean of the two measurements.

Supplementary File S3

ThresholdAtlas: S3: Threshold instability atlas (flips and kappa)

group	parameter	scale	threshold	thr_value	usable_n	flips	flip_rate	kappa
Inter-operator	4CV Area (2Dist).4CV A % (AC)[%]	centile	<5	5	56	0	0	1
Inter-operator	4CV Area (2Dist).4CV A % (AC)[%]	centile	>95	95	56	0	0	1
Intra-operator	4CV Area (2Dist).4CV A % (AC)[%]	centile	<5	5	30	0	0	
Intra-operator	4CV Area (2Dist).4CV A % (AC)[%]	centile	>95	95	30	0	0	1
Inter-operator	4CV Area (2Dist).4CV A % (AUA)[%]	centile	<5	5	54	0	0	1
Inter-operator	4CV Area (2Dist).4CV A % (AUA)[%]	centile	>95	95	54	0	0	1
Intra-operator	4CV Area (2Dist).4CV A % (AUA)[%]	centile	<5	5	30	0	0	1
Intra-operator	4CV Area (2Dist).4CV A % (AUA)[%]	centile	>95	95	30	1	0.033333	0.651163
Inter-operator	4CV Area (2Dist).4CV A % (BPD)[%]	centile	<5	5	55	0	0	1
Inter-operator	4CV Area (2Dist).4CV A % (BPD)[%]	centile	>95	95	55	0	0	1
Intra-operator	4CV Area (2Dist).4CV A % (BPD)[%]	centile	<5	5	30	1	0.033333	0.83871
Intra-operator	4CV Area (2Dist).4CV A % (BPD)[%]	centile	>95	95	30	0	0	
Inter-operator	4CV Area (2Dist).4CV A % (EFW)[%]	centile	<5	5	55	0	0	1
Inter-operator	4CV Area (2Dist).4CV A % (EFW)[%]	centile	>95	95	55	0	0	1
Intra-operator	4CV Area (2Dist).4CV A % (EFW)[%]	centile	<5	5	30	0	0	1
Intra-operator	4CV Area (2Dist).4CV A % (EFW)[%]	centile	>95	95	30	0	0	1
Inter-operator	4CV Area (2Dist).4CV A % (FL)[%]	centile	<5	5	56	0	0	1
Inter-operator	4CV Area (2Dist).4CV A % (FL)[%]	centile	>95	95	56	0	0	1
Intra-operator	4CV Area (2Dist).4CV A % (FL)[%]	centile	<5	5	29	1	0.034483	0.888031
Intra-operator	4CV Area (2Dist).4CV A % (FL)[%]	centile	>95	95	29	1	0.034483	0.650602
Inter-operator	4CV Area (2Dist).4CV A % (HC)[%]	centile	<5	5	55	0	0	1
Inter-operator	4CV Area (2Dist).4CV A % (HC)[%]	centile	>95	95	55	0	0	1
Intra-operator	4CV Area (2Dist).4CV A % (HC)[%]	centile	<5	5	30	0	0	1
Intra-operator	4CV Area (2Dist).4CV A % (HC)[%]	centile	>95	95	30	1	0.033333	0.651163
Inter-operator	4CV Circ (2Dist).4CV C % (AC)[%]	centile	<5	5	56	0	0	
Inter-operator	4CV Circ (2Dist).4CV C % (AC)[%]	centile	>95	95	56	0	0	
Intra-operator	4CV Circ (2Dist).4CV C % (AC)[%]	centile	<5	5	30	0	0	
Intra-operator	4CV Circ (2Dist).4CV C % (AC)[%]	centile	>95	95	30	0	0	
Inter-operator	4CV Circ (2Dist).4CV C % (AUA)[%]	centile	<5	5	54	0	0	1
Inter-operator	4CV Circ (2Dist).4CV C % (AUA)[%]	centile	>95	95	54	0	0	1
Intra-operator	4CV Circ (2Dist).4CV C % (AUA)[%]	centile	<5	5	30	0	0	1
Intra-operator	4CV Circ (2Dist).4CV C % (AUA)[%]	centile	>95	95	30	1	0.033333	0.651163
Inter-operator	4CV Circ (2Dist).4CV C % (BPD)[%]	centile	<5	5	55	0	0	1
Inter-operator	4CV Circ (2Dist).4CV C % (BPD)[%]	centile	>95	95	55	0	0	1
Intra-operator	4CV Circ (2Dist).4CV C % (BPD)[%]	centile	<5	5	30	1	0.033333	0.83871
Intra-operator	4CV Circ (2Dist).4CV C % (BPD)[%]	centile	>95	95	30	0	0	
Inter-operator	4CV Circ (2Dist).4CV C % (EFW)[%]	centile	<5	5	55	0	0	1

group	parameter	scale	threshold	thr_value	usable_n	flips	flip_rate	kappa
Inter-operator	4CV Circ (2Dist).4CV C % (EFW)[%]	centile	>95	95	55	0	0	1
Intra-operator	4CV Circ (2Dist).4CV C % (EFW)[%]	centile	<5	5	30	0	0	1
Intra-operator	4CV Circ (2Dist).4CV C % (EFW)[%]	centile	>95	95	30	0	0	1
Inter-operator	4CV Circ (2Dist).4CV C % (FL)[%]	centile	<5	5	56	0	0	1
Inter-operator	4CV Circ (2Dist).4CV C % (FL)[%]	centile	>95	95	56	0	0	1
Intra-operator	4CV Circ (2Dist).4CV C % (FL)[%]	centile	<5	5	29	1	0.034483	0.868778
Intra-operator	4CV Circ (2Dist).4CV C % (FL)[%]	centile	>95	95	29	1	0.034483	0
Inter-operator	4CV Circ (2Dist).4CV C % (GA)[%]	centile	<5	5	51	0	0	1
Inter-operator	4CV Circ (2Dist).4CV C % (GA)[%]	centile	>95	95	51	0	0	1
Intra-operator	4CV Circ (2Dist).4CV C % (GA)[%]	centile	<5	5	28	0	0	1
Intra-operator	4CV Circ (2Dist).4CV C % (GA)[%]	centile	>95	95	28	0	0	1
Inter-operator	4CV Circ (2Dist).4CV C % (HC)[%]	centile	<5	5	55	0	0	1
Inter-operator	4CV Circ (2Dist).4CV C % (HC)[%]	centile	>95	95	55	0	0	1
Intra-operator	4CV Circ (2Dist).4CV C % (HC)[%]	centile	<5	5	30	0	0	1
Intra-operator	4CV Circ (2Dist).4CV C % (HC)[%]	centile	>95	95	30	0	0	1
Inter-operator	4CV Length ED.4CV L ED % (AC)[%]	centile	<5	5	56	0	0	1
Inter-operator	4CV Length ED.4CV L ED % (AC)[%]	centile	>95	95	56	0	0	1
Intra-operator	4CV Length ED.4CV L ED % (AC)[%]	centile	<5	5	30	0	0	1
Intra-operator	4CV Length ED.4CV L ED % (AC)[%]	centile	>95	95	30	0	0	
Inter-operator	4CV Length ED.4CV L ED % (AUA)[%]	centile	<5	5	54	0	0	1
Inter-operator	4CV Length ED.4CV L ED % (AUA)[%]	centile	>95	95	54	1	0.018519	0.878924
Intra-operator	4CV Length ED.4CV L ED % (AUA)[%]	centile	<5	5	30	1	0.033333	0.83871
Intra-operator	4CV Length ED.4CV L ED % (AUA)[%]	centile	>95	95	30	0	0	
Inter-operator	4CV Length ED.4CV L ED % (BPD)[%]	centile	<5	5	55	0	0	1
Inter-operator	4CV Length ED.4CV L ED % (BPD)[%]	centile	>95	95	55	0	0	1
Intra-operator	4CV Length ED.4CV L ED % (BPD)[%]	centile	<5	5	30	1	0.033333	0.782609
Intra-operator	4CV Length ED.4CV L ED % (BPD)[%]	centile	>95	95	30	0	0	
Inter-operator	4CV Length ED.4CV L ED % (EFW)[%]	centile	<5	5	55	0	0	1
Inter-operator	4CV Length ED.4CV L ED % (EFW)[%]	centile	>95	95	55	0	0	1
Intra-operator	4CV Length ED.4CV L ED % (EFW)[%]	centile	<5	5	30	1	0.033333	0.83871
Intra-operator	4CV Length ED.4CV L ED % (EFW)[%]	centile	>95	95	30	0	0	
Inter-operator	4CV Length ED.4CV L ED % (FL)[%]	centile	<5	5	56	0	0	1
Inter-operator	4CV Length ED.4CV L ED % (FL)[%]	centile	>95	95	56	0	0	1
Intra-operator	4CV Length ED.4CV L ED % (FL)[%]	centile	<5	5	29	1	0.034483	0.868778
Intra-operator	4CV Length ED.4CV L ED % (FL)[%]	centile	>95	95	29	0	0	
Inter-operator	4CV Length ED.4CV L ED % (GA)[%]	centile	<5	5	51	0	0	1
Inter-operator	4CV Length ED.4CV L ED % (GA)[%]	centile	>95	95	51	0	0	1
Intra-operator	4CV Length ED.4CV L ED % (GA)[%]	centile	<5	5	28	1	0.035714	0.9
Intra-operator	4CV Length ED.4CV L ED % (GA)[%]	centile	>95	95	28	0	0	
Inter-operator	4CV Length ED.4CV L ED % (HC)[%]	centile	<5	5	55	0	0	1

group	parameter	scale	threshold	thr_value	usable_n	flips	flip_rate	kappa
Inter-operator	4CV Length ED.4CV L ED % (HC)[%]	centile	>95	95	55	0	0	1
Intra-operator	4CV Length ED.4CV L ED % (HC)[%]	centile	<5	5	30	1	0.033333	0.901961
Intra-operator	4CV Length ED.4CV L ED % (HC)[%]	centile	>95	95	30	0	0	1
Inter-operator	4CV Trv. Width ED.4CV TW ED % (AC)[%]	centile	<5	5	56	0	0	1
Inter-operator	4CV Trv. Width ED.4CV TW ED % (AC)[%]	centile	>95	95	56	0	0	1
Intra-operator	4CV Trv. Width ED.4CV TW ED % (AC)[%]	centile	<5	5	30	0	0	1
Intra-operator	4CV Trv. Width ED.4CV TW ED % (AC)[%]	centile	>95	95	30	0	0	
Inter-operator	4CV Trv. Width ED.4CV TW ED % (AUA)[%]	centile	<5	5	54	0	0	1
Inter-operator	4CV Trv. Width ED.4CV TW ED % (AUA)[%]	centile	>95	95	54	0	0	1
Intra-operator	4CV Trv. Width ED.4CV TW ED % (AUA)[%]	centile	<5	5	30	0	0	1
Intra-operator	4CV Trv. Width ED.4CV TW ED % (AUA)[%]	centile	>95	95	30	1	0.033333	0.782609
Inter-operator	4CV Trv. Width ED.4CV TW ED % (BPD)[%]	centile	<5	5	55	0	0	1
Inter-operator	4CV Trv. Width ED.4CV TW ED % (BPD)[%]	centile	>95	95	55	0	0	1
Intra-operator	4CV Trv. Width ED.4CV TW ED % (BPD)[%]	centile	<5	5	30	1	0.033333	0.911243
Intra-operator	4CV Trv. Width ED.4CV TW ED % (BPD)[%]	centile	>95	95	30	1	0.033333	0
Inter-operator	4CV Trv. Width ED.4CV TW ED % (EFW)[%]	centile	<5	5	55	0	0	1
Inter-operator	4CV Trv. Width ED.4CV TW ED % (EFW)[%]	centile	>95	95	55	0	0	1
Intra-operator	4CV Trv. Width ED.4CV TW ED % (EFW)[%]	centile	<5	5	30	0	0	1
Intra-operator	4CV Trv. Width ED.4CV TW ED % (EFW)[%]	centile	>95	95	30	1	0.033333	0.782609
Inter-operator	4CV Trv. Width ED.4CV TW ED % (FL)[%]	centile	<5	5	56	0	0	1
Inter-operator	4CV Trv. Width ED.4CV TW ED % (FL)[%]	centile	>95	95	56	0	0	1
Intra-operator	4CV Trv. Width ED.4CV TW ED % (FL)[%]	centile	<5	5	29	0	0	1
Intra-operator	4CV Trv. Width ED.4CV TW ED % (FL)[%]	centile	>95	95	29	1	0.034483	0.781955
Inter-operator	4CV Trv. Width ED.4CV TW ED % (GA)[%]	centile	<5	5	51	0	0	1
Inter-operator	4CV Trv. Width ED.4CV TW ED % (GA)[%]	centile	>95	95	51	0	0	1
Intra-operator	4CV Trv. Width ED.4CV TW ED % (GA)[%]	centile	<5	5	28	1	0.035714	0.909091
Intra-operator	4CV Trv. Width ED.4CV TW ED % (GA)[%]	centile	>95	95	28	1	0.035714	0.78125
Inter-operator	4CV Trv. Width ED.4CV TW ED % (HC)[%]	centile	<5	5	55	0	0	1
Inter-operator	4CV Trv. Width ED.4CV TW ED % (HC)[%]	centile	>95	95	55	0	0	1
Intra-operator	4CV Trv. Width ED.4CV TW ED % (HC)[%]	centile	<5	5	30	0	0	1
Intra-operator	4CV Trv. Width ED.4CV TW ED % (HC)[%]	centile	>95	95	30	1	0.033333	0.651163
Inter-operator	LV 24-Segment FS Centiles[%] Segment 1	centile	<5	5	56	22	0.392857	0.10073
Inter-operator	LV 24-Segment FS Centiles[%] Segment 1	centile	>95	95	56	5	0.089286	-0.044776
Intra-operator	LV 24-Segment FS Centiles[%] Segment 1	centile	<5	5	30	14	0.466667	-0.088083
Intra-operator	LV 24-Segment FS Centiles[%] Segment 1	centile	>95	95	30	2	0.066667	0
Inter-operator	LV 24-Segment FS Centiles[%] Segment 10	centile	<5	5	56	26	0.464286	0.056995
Inter-operator	LV 24-Segment FS Centiles[%] Segment 10	centile	>95	95	56	4	0.071429	0.464115
Intra-operator	LV 24-Segment FS Centiles[%] Segment 10	centile	<5	5	30	14	0.466667	0.032258
Intra-operator	LV 24-Segment FS Centiles[%] Segment 10	centile	>95	95	30	0	0	
Inter-operator	LV 24-Segment FS Centiles[%] Segment 11	centile	<5	5	56	25	0.446429	0.118388

group	parameter	scale	threshold	thr_value	usable_n	flips	flip_rate	kappa
Inter-operator	LV 24-Segment FS Centiles[%] Segment 11	centile	>95	95	56	2	0.035714	0.65
Intra-operator	LV 24-Segment FS Centiles[%] Segment 11	centile	<5	5	30	12	0.4	0.104478
Intra-operator	LV 24-Segment FS Centiles[%] Segment 11	centile	>95	95	30	0	0	
Inter-operator	LV 24-Segment FS Centiles[%] Segment 12	centile	<5	5	56	24	0.428571	0.158949
Inter-operator	LV 24-Segment FS Centiles[%] Segment 12	centile	>95	95	56	1	0.017857	0.791045
Intra-operator	LV 24-Segment FS Centiles[%] Segment 12	centile	<5	5	30	12	0.4	0.104478
Intra-operator	LV 24-Segment FS Centiles[%] Segment 12	centile	>95	95	30	0	0	
Inter-operator	LV 24-Segment FS Centiles[%] Segment 13	centile	<5	5	56	26	0.464286	0.09
Inter-operator	LV 24-Segment FS Centiles[%] Segment 13	centile	>95	95	56	0	0	1
Intra-operator	LV 24-Segment FS Centiles[%] Segment 13	centile	<5	5	30	10	0.333333	0.264706
Intra-operator	LV 24-Segment FS Centiles[%] Segment 13	centile	>95	95	30	0	0	
Inter-operator	LV 24-Segment FS Centiles[%] Segment 14	centile	<5	5	56	26	0.464286	0.085427
Inter-operator	LV 24-Segment FS Centiles[%] Segment 14	centile	>95	95	56	0	0	1
Intra-operator	LV 24-Segment FS Centiles[%] Segment 14	centile	<5	5	30	9	0.3	0.314721
Intra-operator	LV 24-Segment FS Centiles[%] Segment 14	centile	>95	95	30	0	0	
Inter-operator	LV 24-Segment FS Centiles[%] Segment 15	centile	<5	5	56	23	0.410714	0.188917
Inter-operator	LV 24-Segment FS Centiles[%] Segment 15	centile	>95	95	56	1	0.017857	0.658537
Intra-operator	LV 24-Segment FS Centiles[%] Segment 15	centile	<5	5	30	10	0.333333	0.253731
Intra-operator	LV 24-Segment FS Centiles[%] Segment 15	centile	>95	95	30	0	0	
Inter-operator	LV 24-Segment FS Centiles[%] Segment 16	centile	<5	5	56	26	0.464286	0.080808
Inter-operator	LV 24-Segment FS Centiles[%] Segment 16	centile	>95	95	56	1	0.017857	0.658537
Intra-operator	LV 24-Segment FS Centiles[%] Segment 16	centile	<5	5	30	9	0.3	0.341463
Intra-operator	LV 24-Segment FS Centiles[%] Segment 16	centile	>95	95	30	0	0	
Inter-operator	LV 24-Segment FS Centiles[%] Segment 17	centile	<5	5	56	26	0.464286	0.071429
Inter-operator	LV 24-Segment FS Centiles[%] Segment 17	centile	>95	95	56	2	0.035714	0
Intra-operator	LV 24-Segment FS Centiles[%] Segment 17	centile	<5	5	30	8	0.266667	0.425837
Intra-operator	LV 24-Segment FS Centiles[%] Segment 17	centile	>95	95	30	0	0	
Inter-operator	LV 24-Segment FS Centiles[%] Segment 18	centile	<5	5	56	26	0.464286	0.071429
Inter-operator	LV 24-Segment FS Centiles[%] Segment 18	centile	>95	95	56	1	0.017857	0
Intra-operator	LV 24-Segment FS Centiles[%] Segment 18	centile	<5	5	30	5	0.166667	0.637681
Intra-operator	LV 24-Segment FS Centiles[%] Segment 18	centile	>95	95	30	0	0	
Inter-operator	LV 24-Segment FS Centiles[%] Segment 19	centile	<5	5	56	24	0.428571	0.142857
Inter-operator	LV 24-Segment FS Centiles[%] Segment 19	centile	>95	95	56	1	0.017857	0
Intra-operator	LV 24-Segment FS Centiles[%] Segment 19	centile	<5	5	30	6	0.2	0.577465
Intra-operator	LV 24-Segment FS Centiles[%] Segment 19	centile	>95	95	30	0	0	
Inter-operator	LV 24-Segment FS Centiles[%] Segment 2	centile	<5	5	56	18	0.321429	0.213729
Inter-operator	LV 24-Segment FS Centiles[%] Segment 2	centile	>95	95	56	3	0.053571	-0.02439
Intra-operator	LV 24-Segment FS Centiles[%] Segment 2	centile	<5	5	30	13	0.433333	0.010152
Intra-operator	LV 24-Segment FS Centiles[%] Segment 2	centile	>95	95	30	1	0.033333	0
Inter-operator	LV 24-Segment FS Centiles[%] Segment 20	centile	<5	5	56	21	0.375	0.253807

group	parameter	scale	threshold	thr_value	usable_n	flips	flip_rate	kappa
Inter-operator	LV 24-Segment FS Centiles[%] Segment 20	centile	>95	95	56	1	0.017857	0
Intra-operator	LV 24-Segment FS Centiles[%] Segment 20	centile	<5	5	30	6	0.2	0.577465
Intra-operator	LV 24-Segment FS Centiles[%] Segment 20	centile	>95	95	30	0	0	
Inter-operator	LV 24-Segment FS Centiles[%] Segment 21	centile	<5	5	56	21	0.375	0.261307
Inter-operator	LV 24-Segment FS Centiles[%] Segment 21	centile	>95	95	56	0	0	
Intra-operator	LV 24-Segment FS Centiles[%] Segment 21	centile	<5	5	30	6	0.2	0.577465
Intra-operator	LV 24-Segment FS Centiles[%] Segment 21	centile	>95	95	30	0	0	
Inter-operator	LV 24-Segment FS Centiles[%] Segment 22	centile	<5	5	56	19	0.339286	0.331658
Inter-operator	LV 24-Segment FS Centiles[%] Segment 22	centile	>95	95	56	0	0	
Intra-operator	LV 24-Segment FS Centiles[%] Segment 22	centile	<5	5	30	9	0.3	0.4
Intra-operator	LV 24-Segment FS Centiles[%] Segment 22	centile	>95	95	30	0	0	
Inter-operator	LV 24-Segment FS Centiles[%] Segment 23	centile	<5	5	56	19	0.339286	0.331658
Inter-operator	LV 24-Segment FS Centiles[%] Segment 23	centile	>95	95	56	0	0	
Intra-operator	LV 24-Segment FS Centiles[%] Segment 23	centile	<5	5	30	8	0.266667	0.466667
Intra-operator	LV 24-Segment FS Centiles[%] Segment 23	centile	>95	95	30	0	0	
Inter-operator	LV 24-Segment FS Centiles[%] Segment 24	centile	<5	5	56	18	0.321429	0.363636
Inter-operator	LV 24-Segment FS Centiles[%] Segment 24	centile	>95	95	56	0	0	
Intra-operator	LV 24-Segment FS Centiles[%] Segment 24	centile	<5	5	30	8	0.266667	0.466667
Intra-operator	LV 24-Segment FS Centiles[%] Segment 24	centile	>95	95	30	0	0	
Inter-operator	LV 24-Segment FS Centiles[%] Segment 3	centile	<5	5	56	18	0.321429	0.181818
Inter-operator	LV 24-Segment FS Centiles[%] Segment 3	centile	>95	95	56	3	0.053571	-0.02439
Intra-operator	LV 24-Segment FS Centiles[%] Segment 3	centile	<5	5	30	13	0.433333	0.010152
Intra-operator	LV 24-Segment FS Centiles[%] Segment 3	centile	>95	95	30	2	0.066667	0
Inter-operator	LV 24-Segment FS Centiles[%] Segment 4	centile	<5	5	56	20	0.357143	0.130435
Inter-operator	LV 24-Segment FS Centiles[%] Segment 4	centile	>95	95	56	3	0.053571	-0.02439
Intra-operator	LV 24-Segment FS Centiles[%] Segment 4	centile	<5	5	30	8	0.266667	0.428571
Intra-operator	LV 24-Segment FS Centiles[%] Segment 4	centile	>95	95	30	2	0.066667	0
Inter-operator	LV 24-Segment FS Centiles[%] Segment 5	centile	<5	5	56	19	0.339286	0.186544
Inter-operator	LV 24-Segment FS Centiles[%] Segment 5	centile	>95	95	56	4	0.071429	-0.037037
Intra-operator	LV 24-Segment FS Centiles[%] Segment 5	centile	<5	5	30	9	0.3	0.366197
Intra-operator	LV 24-Segment FS Centiles[%] Segment 5	centile	>95	95	30	2	0.066667	0
Inter-operator	LV 24-Segment FS Centiles[%] Segment 6	centile	<5	5	56	20	0.357143	0.16042
Inter-operator	LV 24-Segment FS Centiles[%] Segment 6	centile	>95	95	56	4	0.071429	-0.037037
Intra-operator	LV 24-Segment FS Centiles[%] Segment 6	centile	<5	5	30	9	0.3	0.372093
Intra-operator	LV 24-Segment FS Centiles[%] Segment 6	centile	>95	95	30	1	0.033333	0
Inter-operator	LV 24-Segment FS Centiles[%] Segment 7	centile	<5	5	56	23	0.410714	0.052941
Inter-operator	LV 24-Segment FS Centiles[%] Segment 7	centile	>95	95	56	4	0.071429	0.3
Intra-operator	LV 24-Segment FS Centiles[%] Segment 7	centile	<5	5	30	11	0.366667	0.26009
Intra-operator	LV 24-Segment FS Centiles[%] Segment 7	centile	>95	95	30	0	0	
Inter-operator	LV 24-Segment FS Centiles[%] Segment 8	centile	<5	5	56	23	0.410714	0.110497

group	parameter	scale	threshold	thr_value	usable_n	flips	flip_rate	kappa
Inter-operator	LV 24-Segment FS Centiles[%] Segment 8	centile	>95	95	56	5	0.089286	0.247312
Intra-operator	LV 24-Segment FS Centiles[%] Segment 8	centile	<5	5	30	11	0.366667	0.273128
Intra-operator	LV 24-Segment FS Centiles[%] Segment 8	centile	>95	95	30	0	0	
Inter-operator	LV 24-Segment FS Centiles[%] Segment 9	centile	<5	5	56	26	0.464286	0.031915
Inter-operator	LV 24-Segment FS Centiles[%] Segment 9	centile	>95	95	56	6	0.107143	0.196172
Intra-operator	LV 24-Segment FS Centiles[%] Segment 9	centile	<5	5	30	13	0.433333	0.109589
Intra-operator	LV 24-Segment FS Centiles[%] Segment 9	centile	>95	95	30	0	0	
Inter-operator	LV 24-Segment SI Centiles[%] Segment 1	centile	<5	5	56	11	0.196429	0.398438
Inter-operator	LV 24-Segment SI Centiles[%] Segment 1	centile	>95	95	56	1	0.017857	0
Intra-operator	LV 24-Segment SI Centiles[%] Segment 1	centile	<5	5	30	9	0.3	0.262295
Intra-operator	LV 24-Segment SI Centiles[%] Segment 1	centile	>95	95	30	1	0.033333	0
Inter-operator	LV 24-Segment SI Centiles[%] Segment 10	centile	<5	5	56	10	0.178571	0.478585
Inter-operator	LV 24-Segment SI Centiles[%] Segment 10	centile	>95	95	56	6	0.107143	-0.05
Intra-operator	LV 24-Segment SI Centiles[%] Segment 10	centile	<5	5	30	10	0.333333	0.210526
Intra-operator	LV 24-Segment SI Centiles[%] Segment 10	centile	>95	95	30	1	0.033333	0
Inter-operator	LV 24-Segment SI Centiles[%] Segment 11	centile	<5	5	56	10	0.178571	0.478585
Inter-operator	LV 24-Segment SI Centiles[%] Segment 11	centile	>95	95	56	8	0.142857	-0.07177
Intra-operator	LV 24-Segment SI Centiles[%] Segment 11	centile	<5	5	30	10	0.333333	0.210526
Intra-operator	LV 24-Segment SI Centiles[%] Segment 11	centile	>95	95	30	1	0.033333	0
Inter-operator	LV 24-Segment SI Centiles[%] Segment 12	centile	<5	5	56	10	0.178571	0.473684
Inter-operator	LV 24-Segment SI Centiles[%] Segment 12	centile	>95	95	56	6	0.107143	0.196172
Intra-operator	LV 24-Segment SI Centiles[%] Segment 12	centile	<5	5	30	11	0.366667	0.108108
Intra-operator	LV 24-Segment SI Centiles[%] Segment 12	centile	>95	95	30	2	0.066667	0
Inter-operator	LV 24-Segment SI Centiles[%] Segment 13	centile	<5	5	56	10	0.178571	0.473684
Inter-operator	LV 24-Segment SI Centiles[%] Segment 13	centile	>95	95	56	6	0.107143	0.196172
Intra-operator	LV 24-Segment SI Centiles[%] Segment 13	centile	<5	5	30	12	0.4	-0.090909
Intra-operator	LV 24-Segment SI Centiles[%] Segment 13	centile	>95	95	30	2	0.066667	0
Inter-operator	LV 24-Segment SI Centiles[%] Segment 14	centile	<5	5	56	12	0.214286	0.222222
Inter-operator	LV 24-Segment SI Centiles[%] Segment 14	centile	>95	95	56	7	0.125	0.155172
Intra-operator	LV 24-Segment SI Centiles[%] Segment 14	centile	<5	5	30	11	0.366667	-0.064516
Intra-operator	LV 24-Segment SI Centiles[%] Segment 14	centile	>95	95	30	2	0.066667	0
Inter-operator	LV 24-Segment SI Centiles[%] Segment 15	centile	<5	5	56	11	0.196429	0.167568
Inter-operator	LV 24-Segment SI Centiles[%] Segment 15	centile	>95	95	56	7	0.125	0.294964
Intra-operator	LV 24-Segment SI Centiles[%] Segment 15	centile	<5	5	30	11	0.366667	-0.064516
Intra-operator	LV 24-Segment SI Centiles[%] Segment 15	centile	>95	95	30	2	0.066667	-0.034483
Inter-operator	LV 24-Segment SI Centiles[%] Segment 16	centile	<5	5	56	11	0.196429	0.060976
Inter-operator	LV 24-Segment SI Centiles[%] Segment 16	centile	>95	95	56	6	0.107143	0.34375
Intra-operator	LV 24-Segment SI Centiles[%] Segment 16	centile	<5	5	30	8	0.266667	-0.111111
Intra-operator	LV 24-Segment SI Centiles[%] Segment 16	centile	>95	95	30	3	0.1	-0.046512
Inter-operator	LV 24-Segment SI Centiles[%] Segment 17	centile	<5	5	56	8	0.142857	0.135135

group	parameter	scale	threshold	thr_value	usable_n	flips	flip_rate	kappa
Inter-operator	LV 24-Segment SI Centiles[%] Segment 17	centile	>95	95	56	6	0.107143	0.34375
Intra-operator	LV 24-Segment SI Centiles[%] Segment 17	centile	<5	5	30	7	0.233333	-0.105263
Intra-operator	LV 24-Segment SI Centiles[%] Segment 17	centile	>95	95	30	4	0.133333	-0.071429
Inter-operator	LV 24-Segment SI Centiles[%] Segment 18	centile	<5	5	56	8	0.142857	0.135135
Inter-operator	LV 24-Segment SI Centiles[%] Segment 18	centile	>95	95	56	6	0.107143	0.34375
Intra-operator	LV 24-Segment SI Centiles[%] Segment 18	centile	<5	5	30	6	0.2	-0.097561
Intra-operator	LV 24-Segment SI Centiles[%] Segment 18	centile	>95	95	30	4	0.133333	-0.071429
Inter-operator	LV 24-Segment SI Centiles[%] Segment 19	centile	<5	5	56	6	0.107143	0.207547
Inter-operator	LV 24-Segment SI Centiles[%] Segment 19	centile	>95	95	56	8	0.142857	0.255814
Intra-operator	LV 24-Segment SI Centiles[%] Segment 19	centile	<5	5	30	5	0.166667	-0.056338
Intra-operator	LV 24-Segment SI Centiles[%] Segment 19	centile	>95	95	30	5	0.166667	-0.086957
Inter-operator	LV 24-Segment SI Centiles[%] Segment 2	centile	<5	5	56	10	0.178571	0.470699
Inter-operator	LV 24-Segment SI Centiles[%] Segment 2	centile	>95	95	56	2	0.035714	-0.018182
Intra-operator	LV 24-Segment SI Centiles[%] Segment 2	centile	<5	5	30	10	0.333333	0.210526
Intra-operator	LV 24-Segment SI Centiles[%] Segment 2	centile	>95	95	30	1	0.033333	0
Inter-operator	LV 24-Segment SI Centiles[%] Segment 20	centile	<5	5	56	5	0.089286	-0.029412
Inter-operator	LV 24-Segment SI Centiles[%] Segment 20	centile	>95	95	56	9	0.160714	0.311475
Intra-operator	LV 24-Segment SI Centiles[%] Segment 20	centile	<5	5	30	4	0.133333	-0.052632
Intra-operator	LV 24-Segment SI Centiles[%] Segment 20	centile	>95	95	30	5	0.166667	-0.086957
Inter-operator	LV 24-Segment SI Centiles[%] Segment 21	centile	<5	5	56	4	0.071429	-0.027523
Inter-operator	LV 24-Segment SI Centiles[%] Segment 21	centile	>95	95	56	10	0.178571	0.278351
Intra-operator	LV 24-Segment SI Centiles[%] Segment 21	centile	<5	5	30	3	0.1	-0.046512
Intra-operator	LV 24-Segment SI Centiles[%] Segment 21	centile	>95	95	30	5	0.166667	0.193548
Inter-operator	LV 24-Segment SI Centiles[%] Segment 22	centile	<5	5	56	3	0.053571	-0.02439
Inter-operator	LV 24-Segment SI Centiles[%] Segment 22	centile	>95	95	56	11	0.196429	0.24878
Intra-operator	LV 24-Segment SI Centiles[%] Segment 22	centile	<5	5	30	2	0.066667	-0.034483
Intra-operator	LV 24-Segment SI Centiles[%] Segment 22	centile	>95	95	30	6	0.2	0.134615
Inter-operator	LV 24-Segment SI Centiles[%] Segment 23	centile	<5	5	56	3	0.053571	-0.02439
Inter-operator	LV 24-Segment SI Centiles[%] Segment 23	centile	>95	95	56	11	0.196429	0.24878
Intra-operator	LV 24-Segment SI Centiles[%] Segment 23	centile	<5	5	30	2	0.066667	-0.034483
Intra-operator	LV 24-Segment SI Centiles[%] Segment 23	centile	>95	95	30	6	0.2	0.134615
Inter-operator	LV 24-Segment SI Centiles[%] Segment 24	centile	<5	5	56	3	0.053571	-0.02439
Inter-operator	LV 24-Segment SI Centiles[%] Segment 24	centile	>95	95	56	13	0.232143	0.112195
Intra-operator	LV 24-Segment SI Centiles[%] Segment 24	centile	<5	5	30	2	0.066667	-0.034483
Intra-operator	LV 24-Segment SI Centiles[%] Segment 24	centile	>95	95	30	6	0.2	0.134615
Inter-operator	LV 24-Segment SI Centiles[%] Segment 3	centile	<5	5	56	9	0.160714	0.585526
Inter-operator	LV 24-Segment SI Centiles[%] Segment 3	centile	>95	95	56	3	0.053571	-0.02439
Intra-operator	LV 24-Segment SI Centiles[%] Segment 3	centile	<5	5	30	11	0.366667	0.153846
Intra-operator	LV 24-Segment SI Centiles[%] Segment 3	centile	>95	95	30	1	0.033333	0
Inter-operator	LV 24-Segment SI Centiles[%] Segment 4	centile	<5	5	56	9	0.160714	0.585526

group	parameter	scale	threshold	thr_value	usable_n	flips	flip_rate	kappa
Inter-operator	LV 24-Segment SI Centiles[%] Segment 4	centile	>95	95	56	3	0.053571	-0.02439
Intra-operator	LV 24-Segment SI Centiles[%] Segment 4	centile	<5	5	30	11	0.366667	0.195122
Intra-operator	LV 24-Segment SI Centiles[%] Segment 4	centile	>95	95	30	2	0.066667	0
Inter-operator	LV 24-Segment SI Centiles[%] Segment 5	centile	<5	5	56	8	0.142857	0.650546
Inter-operator	LV 24-Segment SI Centiles[%] Segment 5	centile	>95	95	56	3	0.053571	-0.02439
Intra-operator	LV 24-Segment SI Centiles[%] Segment 5	centile	<5	5	30	10	0.333333	0.25
Intra-operator	LV 24-Segment SI Centiles[%] Segment 5	centile	>95	95	30	2	0.066667	-0.034483
Inter-operator	LV 24-Segment SI Centiles[%] Segment 6	centile	<5	5	56	9	0.160714	0.6
Inter-operator	LV 24-Segment SI Centiles[%] Segment 6	centile	>95	95	56	4	0.071429	-0.037037
Intra-operator	LV 24-Segment SI Centiles[%] Segment 6	centile	<5	5	30	10	0.333333	0.25
Intra-operator	LV 24-Segment SI Centiles[%] Segment 6	centile	>95	95	30	2	0.066667	-0.034483
Inter-operator	LV 24-Segment SI Centiles[%] Segment 7	centile	<5	5	56	10	0.178571	0.545455
Inter-operator	LV 24-Segment SI Centiles[%] Segment 7	centile	>95	95	56	4	0.071429	-0.037037
Intra-operator	LV 24-Segment SI Centiles[%] Segment 7	centile	<5	5	30	10	0.333333	0.25
Intra-operator	LV 24-Segment SI Centiles[%] Segment 7	centile	>95	95	30	2	0.066667	-0.034483
Inter-operator	LV 24-Segment SI Centiles[%] Segment 8	centile	<5	5	56	12	0.214286	0.432432
Inter-operator	LV 24-Segment SI Centiles[%] Segment 8	centile	>95	95	56	5	0.089286	-0.044776
Intra-operator	LV 24-Segment SI Centiles[%] Segment 8	centile	<5	5	30	10	0.333333	0.25
Intra-operator	LV 24-Segment SI Centiles[%] Segment 8	centile	>95	95	30	2	0.066667	-0.034483
Inter-operator	LV 24-Segment SI Centiles[%] Segment 9	centile	<5	5	56	10	0.178571	0.502664
Inter-operator	LV 24-Segment SI Centiles[%] Segment 9	centile	>95	95	56	5	0.089286	-0.044776
Intra-operator	LV 24-Segment SI Centiles[%] Segment 9	centile	<5	5	30	10	0.333333	0.25
Intra-operator	LV 24-Segment SI Centiles[%] Segment 9	centile	>95	95	30	1	0.033333	0
Inter-operator	LV_ Basal-Apical FS.Lt BASE-AP FS %[%]	centile	<5	5	56	18	0.321429	0.360406
Inter-operator	LV_ Basal-Apical FS.Lt BASE-AP FS %[%]	centile	>95	95	56	12	0.214286	0.213115
Intra-operator	LV_ Basal-Apical FS.Lt BASE-AP FS %[%]	centile	<5	5	30	5	0.166667	0.634146
Intra-operator	LV_ Basal-Apical FS.Lt BASE-AP FS %[%]	centile	>95	95	30	6	0.2	0.142857
Inter-operator	LV_ Basal-Apical FS.Lt BASE-AP FS ZS[]	zscore	Z<-2	2	55	19	0.345455	0.296296
Inter-operator	LV_ Basal-Apical FS.Lt BASE-AP FS ZS[]	zscore	Z>2	2	55	10	0.181818	0.270557
Inter-operator	LV_ Basal-Apical FS.Lt BASE-AP FS ZS[]	zscore	|Z|>1.645	1.645	55	22	0.4	0.115497
Inter-operator	LV_ Basal-Apical FS.Lt BASE-AP FS ZS[]	zscore	|Z|>2.0	2	55	25	0.454545	0.071573
Intra-operator	LV_ Basal-Apical FS.Lt BASE-AP FS ZS[]	zscore	Z<-2	2	28	5	0.178571	0.642857
Intra-operator	LV_ Basal-Apical FS.Lt BASE-AP FS ZS[]	zscore	Z>2	2	28	4	0.142857	0.263158
Intra-operator	LV_ Basal-Apical FS.Lt BASE-AP FS ZS[]	zscore	|Z|>1.645	1.645	28	7	0.25	0.309859
Intra-operator	LV_ Basal-Apical FS.Lt BASE-AP FS ZS[]	zscore	|Z|>2.0	2	28	7	0.25	0.467391
Inter-operator	LV_ Basal-Lat.Wall FS.Lt Basal-Lat FS % (AC)[%]	centile	<5	5	56	21	0.375	0.238342
Inter-operator	LV_ Basal-Lat.Wall FS.Lt Basal-Lat FS % (AC)[%]	centile	>95	95	56	13	0.232143	-0.130435
Intra-operator	LV_ Basal-Lat.Wall FS.Lt Basal-Lat FS % (AC)[%]	centile	<5	5	30	6	0.2	0.60177
Intra-operator	LV_ Basal-Lat.Wall FS.Lt Basal-Lat FS % (AC)[%]	centile	>95	95	30	6	0.2	0.166667
Inter-operator	LV_ Basal-Lat.Wall FS.Lt Basal-Lat FS % (AUA)[%]	centile	<5	5	54	19	0.351852	0.255443

group	parameter	scale	threshold	thr_value	usable_n	flips	flip_rate	kappa
Inter-operator	LV_ Basal-Lat.Wall FS.Lt Basal-Lat FS % (AUA)[%]	centile	>95	95	54	12	0.222222	-0.125
Intra-operator	LV_ Basal-Lat.Wall FS.Lt Basal-Lat FS % (AUA)[%]	centile	<5	5	30	6	0.2	0.60177
Intra-operator	LV_ Basal-Lat.Wall FS.Lt Basal-Lat FS % (AUA)[%]	centile	>95	95	30	6	0.2	0.166667
Inter-operator	LV_ Basal-Lat.Wall FS.Lt Basal-Lat FS % (BPD)[%]	centile	<5	5	55	19	0.345455	0.285714
Inter-operator	LV_ Basal-Lat.Wall FS.Lt Basal-Lat FS % (BPD)[%]	centile	>95	95	55	14	0.254545	-0.142433
Intra-operator	LV_ Basal-Lat.Wall FS.Lt Basal-Lat FS % (BPD)[%]	centile	<5	5	30	6	0.2	0.60177
Intra-operator	LV_ Basal-Lat.Wall FS.Lt Basal-Lat FS % (BPD)[%]	centile	>95	95	30	6	0.2	0.166667
Inter-operator	LV_ Basal-Lat.Wall FS.Lt Basal-Lat FS % (EFW)[%]	centile	<5	5	55	18	0.327273	0.31912
Inter-operator	LV_ Basal-Lat.Wall FS.Lt Basal-Lat FS % (EFW)[%]	centile	>95	95	55	12	0.218182	-0.122449
Intra-operator	LV_ Basal-Lat.Wall FS.Lt Basal-Lat FS % (EFW)[%]	centile	<5	5	30	7	0.233333	0.537445
Intra-operator	LV_ Basal-Lat.Wall FS.Lt Basal-Lat FS % (EFW)[%]	centile	>95	95	30	6	0.2	0.166667
Inter-operator	LV_ Basal-Lat.Wall FS.Lt Basal-Lat FS % (FL)[%]	centile	<5	5	55	20	0.363636	0.252717
Inter-operator	LV_ Basal-Lat.Wall FS.Lt Basal-Lat FS % (FL)[%]	centile	>95	95	55	13	0.236364	-0.125984
Intra-operator	LV_ Basal-Lat.Wall FS.Lt Basal-Lat FS % (FL)[%]	centile	<5	5	29	6	0.206897	0.587678
Intra-operator	LV_ Basal-Lat.Wall FS.Lt Basal-Lat FS % (FL)[%]	centile	>95	95	29	5	0.172414	0.20765
Inter-operator	LV_ Basal-Lat.Wall FS.Lt Basal-Lat FS % (GA)[%]	centile	<5	5	51	17	0.333333	0.294548
Inter-operator	LV_ Basal-Lat.Wall FS.Lt Basal-Lat FS % (GA)[%]	centile	>95	95	51	7	0.137255	-0.072072
Intra-operator	LV_ Basal-Lat.Wall FS.Lt Basal-Lat FS % (GA)[%]	centile	<5	5	28	8	0.285714	0.428571
Intra-operator	LV_ Basal-Lat.Wall FS.Lt Basal-Lat FS % (GA)[%]	centile	>95	95	28	6	0.214286	0.16
Inter-operator	LV_ Basal-Lat.Wall FS.Lt Basal-Lat FS % (HC)[%]	centile	<5	5	55	20	0.363636	0.252717
Inter-operator	LV_ Basal-Lat.Wall FS.Lt Basal-Lat FS % (HC)[%]	centile	>95	95	55	14	0.254545	-0.142433
Intra-operator	LV_ Basal-Lat.Wall FS.Lt Basal-Lat FS % (HC)[%]	centile	<5	5	30	6	0.2	0.60177
Intra-operator	LV_ Basal-Lat.Wall FS.Lt Basal-Lat FS % (HC)[%]	centile	>95	95	30	6	0.2	0.166667
Inter-operator	LV_ Basal-Sep.Wall FS.Lt Basal-Sep FS % (AC)[%]	centile	<5	5	56	14	0.25	0.366721
Inter-operator	LV_ Basal-Sep.Wall FS.Lt Basal-Sep FS % (AC)[%]	centile	>95	95	56	12	0.214286	0.363636
Intra-operator	LV_ Basal-Sep.Wall FS.Lt Basal-Sep FS % (AC)[%]	centile	<5	5	30	10	0.333333	0.210526
Intra-operator	LV_ Basal-Sep.Wall FS.Lt Basal-Sep FS % (AC)[%]	centile	>95	95	30	4	0.133333	0.52381
Inter-operator	LV_ Basal-Sep.Wall FS.Lt Basal-Sep FS % (AUA)[%]	centile	<5	5	54	13	0.240741	0.414023
Inter-operator	LV_ Basal-Sep.Wall FS.Lt Basal-Sep FS % (AUA)[%]	centile	>95	95	54	12	0.222222	0.357143
Intra-operator	LV_ Basal-Sep.Wall FS.Lt Basal-Sep FS % (AUA)[%]	centile	<5	5	30	10	0.333333	0.210526
Intra-operator	LV_ Basal-Sep.Wall FS.Lt Basal-Sep FS % (AUA)[%]	centile	>95	95	30	4	0.133333	0.52381
Inter-operator	LV_ Basal-Sep.Wall FS.Lt Basal-Sep FS % (BPD)[%]	centile	<5	5	55	14	0.254545	0.384
Inter-operator	LV_ Basal-Sep.Wall FS.Lt Basal-Sep FS % (BPD)[%]	centile	>95	95	55	12	0.218182	0.318182
Intra-operator	LV_ Basal-Sep.Wall FS.Lt Basal-Sep FS % (BPD)[%]	centile	<5	5	30	9	0.3	0.307692
Intra-operator	LV_ Basal-Sep.Wall FS.Lt Basal-Sep FS % (BPD)[%]	centile	>95	95	30	4	0.133333	0.52381
Inter-operator	LV_ Basal-Sep.Wall FS.Lt Basal-Sep FS % (EFW)[%]	centile	<5	5	55	14	0.254545	0.384
Inter-operator	LV_ Basal-Sep.Wall FS.Lt Basal-Sep FS % (EFW)[%]	centile	>95	95	55	11	0.2	0.352941
Intra-operator	LV_ Basal-Sep.Wall FS.Lt Basal-Sep FS % (EFW)[%]	centile	<5	5	30	9	0.3	0.341463
Intra-operator	LV_ Basal-Sep.Wall FS.Lt Basal-Sep FS % (EFW)[%]	centile	>95	95	30	4	0.133333	0.52381
Inter-operator	LV_ Basal-Sep.Wall FS.Lt Basal-Sep FS % (FL)[%]	centile	<5	5	55	15	0.272727	0.352941

group	parameter	scale	threshold	thr_value	usable_n	flips	flip_rate	kappa
Inter-operator	LV_ Basal-Sep.Wall FS.Lt Basal-Sep FS % (FL)[%]	centile	>95	95	55	12	0.218182	0.318182
Intra-operator	LV_ Basal-Sep.Wall FS.Lt Basal-Sep FS % (FL)[%]	centile	<5	5	29	10	0.344828	0.198895
Intra-operator	LV_ Basal-Sep.Wall FS.Lt Basal-Sep FS % (FL)[%]	centile	>95	95	29	3	0.103448	0.606335
Inter-operator	LV_ Basal-Sep.Wall FS.Lt Basal-Sep FS % (GA)[%]	centile	<5	5	51	14	0.27451	0.340111
Inter-operator	LV_ Basal-Sep.Wall FS.Lt Basal-Sep FS % (GA)[%]	centile	>95	95	51	13	0.254902	0.220917
Intra-operator	LV_ Basal-Sep.Wall FS.Lt Basal-Sep FS % (GA)[%]	centile	<5	5	28	10	0.357143	0.054054
Intra-operator	LV_ Basal-Sep.Wall FS.Lt Basal-Sep FS % (GA)[%]	centile	>95	95	28	4	0.142857	0.517241
Inter-operator	LV_ Basal-Sep.Wall FS.Lt Basal-Sep FS % (HC)[%]	centile	<5	5	55	14	0.254545	0.384
Inter-operator	LV_ Basal-Sep.Wall FS.Lt Basal-Sep FS % (HC)[%]	centile	>95	95	55	12	0.218182	0.318182
Intra-operator	LV_ Basal-Sep.Wall FS.Lt Basal-Sep FS % (HC)[%]	centile	<5	5	30	9	0.3	0.307692
Intra-operator	LV_ Basal-Sep.Wall FS.Lt Basal-Sep FS % (HC)[%]	centile	>95	95	30	4	0.133333	0.52381
Inter-operator	LV_ CO.Lt CO % (AC)[%]	centile	<5	5	28	7	0.25	0.5
Inter-operator	LV_ CO.Lt CO % (AC)[%]	centile	>95	95	28	1	0.035714	0.65
Intra-operator	LV_ CO.Lt CO % (AC)[%]	centile	<5	5	19	2	0.105263	0.728571
Intra-operator	LV_ CO.Lt CO % (AC)[%]	centile	>95	95	19	0	0	
Inter-operator	LV_ CO.Lt CO % (AUA)[%]	centile	<5	5	54	11	0.203704	0.395112
Inter-operator	LV_ CO.Lt CO % (AUA)[%]	centile	>95	95	54	1	0.018519	0.658228
Intra-operator	LV_ CO.Lt CO % (AUA)[%]	centile	<5	5	30	4	0.133333	0.52
Intra-operator	LV_ CO.Lt CO % (AUA)[%]	centile	>95	95	30	0	0	
Inter-operator	LV_ CO.Lt CO % (BPD)[%]	centile	<5	5	51	16	0.313725	0.245841
Inter-operator	LV_ CO.Lt CO % (BPD)[%]	centile	>95	95	51	3	0.058824	-0.026846
Intra-operator	LV_ CO.Lt CO % (BPD)[%]	centile	<5	5	30	6	0.2	0.444444
Intra-operator	LV_ CO.Lt CO % (BPD)[%]	centile	>95	95	30	0	0	
Inter-operator	LV_ CO.Lt CO % (EFW)[%]	centile	<5	5	19	5	0.263158	0.469274
Inter-operator	LV_ CO.Lt CO % (EFW)[%]	centile	>95	95	19	0	0	1
Intra-operator	LV_ CO.Lt CO % (EFW)[%]	centile	<5	5	16	4	0.25	0.428571
Intra-operator	LV_ CO.Lt CO % (EFW)[%]	centile	>95	95	16	0	0	
Inter-operator	LV_ CO.Lt CO % (FL)[%]	centile	<5	5	33	13	0.393939	0.215722
Inter-operator	LV_ CO.Lt CO % (FL)[%]	centile	>95	95	33	1	0.030303	0
Intra-operator	LV_ CO.Lt CO % (FL)[%]	centile	<5	5	17	6	0.352941	0.238806
Intra-operator	LV_ CO.Lt CO % (FL)[%]	centile	>95	95	17	0	0	
Inter-operator	LV_ CO.Lt CO % (GA)[%]	centile	<5	5	51	15	0.294118	0.27762
Inter-operator	LV_ CO.Lt CO % (GA)[%]	centile	>95	95	51	2	0.039216	0.479592
Intra-operator	LV_ CO.Lt CO % (GA)[%]	centile	<5	5	28	5	0.178571	0.339623
Intra-operator	LV_ CO.Lt CO % (GA)[%]	centile	>95	95	28	0	0	
Inter-operator	LV_ CO.Lt CO % (HC)[%]	centile	<5	5	14	5	0.357143	0.285714
Inter-operator	LV_ CO.Lt CO % (HC)[%]	centile	>95	95	14	0	0	1
Intra-operator	LV_ CO.Lt CO % (HC)[%]	centile	<5	5	13	3	0.230769	0.493506
Intra-operator	LV_ CO.Lt CO % (HC)[%]	centile	>95	95	13	0	0	
Inter-operator	LV_ CO/KG.Lt CO/KG % (AC)[%]	centile	<5	5	27	13	0.481481	0.04878

group	parameter	scale	threshold	thr_value	usable_n	flips	flip_rate	kappa
Inter-operator	LV_ CO/KG.Lt CO/KG % (AC)[%]	centile	>95	95	27	0	0	1
Intra-operator	LV_ CO/KG.Lt CO/KG % (AC)[%]	centile	<5	5	19	3	0.157895	0.564885
Intra-operator	LV_ CO/KG.Lt CO/KG % (AC)[%]	centile	>95	95	19	0	0	
Inter-operator	LV_ CO/KG.Lt CO/KG % (AUA)[%]	centile	<5	5	53	25	0.471698	0.052895
Inter-operator	LV_ CO/KG.Lt CO/KG % (AUA)[%]	centile	>95	95	53	0	0	1
Intra-operator	LV_ CO/KG.Lt CO/KG % (AUA)[%]	centile	<5	5	30	8	0.266667	0.368421
Intra-operator	LV_ CO/KG.Lt CO/KG % (AUA)[%]	centile	>95	95	30	0	0	
Inter-operator	LV_ CO/KG.Lt CO/KG % (BPD)[%]	centile	<5	5	51	24	0.470588	0.0347
Inter-operator	LV_ CO/KG.Lt CO/KG % (BPD)[%]	centile	>95	95	51	1	0.019608	0.657718
Intra-operator	LV_ CO/KG.Lt CO/KG % (BPD)[%]	centile	<5	5	30	8	0.266667	0.322034
Intra-operator	LV_ CO/KG.Lt CO/KG % (BPD)[%]	centile	>95	95	30	0	0	
Inter-operator	LV_ CO/KG.Lt CO/KG % (EFW)[%]	centile	<5	5	19	7	0.368421	0.265193
Inter-operator	LV_ CO/KG.Lt CO/KG % (EFW)[%]	centile	>95	95	19	0	0	1
Intra-operator	LV_ CO/KG.Lt CO/KG % (EFW)[%]	centile	<5	5	16	3	0.1875	0.538462
Intra-operator	LV_ CO/KG.Lt CO/KG % (EFW)[%]	centile	>95	95	16	0	0	
Inter-operator	LV_ CO/KG.Lt CO/KG % (FL)[%]	centile	<5	5	32	15	0.46875	0.069767
Inter-operator	LV_ CO/KG.Lt CO/KG % (FL)[%]	centile	>95	95	32	0	0	1
Intra-operator	LV_ CO/KG.Lt CO/KG % (FL)[%]	centile	<5	5	17	5	0.294118	0.330709
Intra-operator	LV_ CO/KG.Lt CO/KG % (FL)[%]	centile	>95	95	17	0	0	
Inter-operator	LV_ CO/KG.Lt CO/KG % (GA)[%]	centile	<5	5	50	20	0.4	0.193548
Inter-operator	LV_ CO/KG.Lt CO/KG % (GA)[%]	centile	>95	95	50	1	0.02	0.657534
Intra-operator	LV_ CO/KG.Lt CO/KG % (GA)[%]	centile	<5	5	28	8	0.285714	0.36
Intra-operator	LV_ CO/KG.Lt CO/KG % (GA)[%]	centile	>95	95	28	0	0	
Inter-operator	LV_ CO/KG.Lt CO/KG % (HC)[%]	centile	<5	5	14	5	0.357143	0.285714
Inter-operator	LV_ CO/KG.Lt CO/KG % (HC)[%]	centile	>95	95	14	0	0	1
Intra-operator	LV_ CO/KG.Lt CO/KG % (HC)[%]	centile	<5	5	13	4	0.307692	0.35
Intra-operator	LV_ CO/KG.Lt CO/KG % (HC)[%]	centile	>95	95	13	0	0	
Inter-operator	LV_ ED Area.Lt ED Area % (AC)[%]	centile	<5	5	56	6	0.107143	0.34375
Inter-operator	LV_ ED Area.Lt ED Area % (AC)[%]	centile	>95	95	56	7	0.125	0.514851
Intra-operator	LV_ ED Area.Lt ED Area % (AC)[%]	centile	<5	5	30	7	0.233333	0.086957
Intra-operator	LV_ ED Area.Lt ED Area % (AC)[%]	centile	>95	95	30	3	0.1	0.516129
Inter-operator	LV_ ED Area.Lt ED Area % (AUA)[%]	centile	<5	5	54	7	0.12963	0.613497
Inter-operator	LV_ ED Area.Lt ED Area % (AUA)[%]	centile	>95	95	54	3	0.055556	0.547486
Intra-operator	LV_ ED Area.Lt ED Area % (AUA)[%]	centile	<5	5	30	8	0.266667	0.368421
Intra-operator	LV_ ED Area.Lt ED Area % (AUA)[%]	centile	>95	95	30	1	0.033333	0.651163
Inter-operator	LV_ ED Area.Lt ED Area % (BPD)[%]	centile	<5	5	55	6	0.109091	0.713043
Inter-operator	LV_ ED Area.Lt ED Area % (BPD)[%]	centile	>95	95	55	3	0.054545	0.542936
Intra-operator	LV_ ED Area.Lt ED Area % (BPD)[%]	centile	<5	5	30	8	0.266667	0.368421
Intra-operator	LV_ ED Area.Lt ED Area % (BPD)[%]	centile	>95	95	30	1	0.033333	0.651163
Inter-operator	LV_ ED Area.Lt ED Area % (EFW)[%]	centile	<5	5	55	6	0.109091	0.56117

group	parameter	scale	threshold	thr_value	usable_n	flips	flip_rate	kappa
Inter-operator	LV_ ED Area.Lt ED Area % (EFW)[%]	centile	>95	95	55	3	0.054545	0.372624
Intra-operator	LV_ ED Area.Lt ED Area % (EFW)[%]	centile	<5	5	30	9	0.3	0
Intra-operator	LV_ ED Area.Lt ED Area % (EFW)[%]	centile	>95	95	30	0	0	1
Inter-operator	LV_ ED Area.Lt ED Area % (FL)[%]	centile	<5	5	55	8	0.145455	0.468599
Inter-operator	LV_ ED Area.Lt ED Area % (FL)[%]	centile	>95	95	55	5	0.090909	0.495413
Intra-operator	LV_ ED Area.Lt ED Area % (FL)[%]	centile	<5	5	29	6	0.206897	0.280992
Intra-operator	LV_ ED Area.Lt ED Area % (FL)[%]	centile	>95	95	29	1	0.034483	0
Inter-operator	LV_ ED Area.Lt ED Area % (GA)[%]	centile	<5	5	51	9	0.176471	0.49505
Inter-operator	LV_ ED Area.Lt ED Area % (GA)[%]	centile	>95	95	51	1	0.019608	0.790123
Intra-operator	LV_ ED Area.Lt ED Area % (GA)[%]	centile	<5	5	28	9	0.321429	0.240964
Intra-operator	LV_ ED Area.Lt ED Area % (GA)[%]	centile	>95	95	28	0	0	1
Inter-operator	LV_ ED Area.Lt ED Area % (HC)[%]	centile	<5	5	55	7	0.127273	0.673452
Inter-operator	LV_ ED Area.Lt ED Area % (HC)[%]	centile	>95	95	55	4	0.072727	0.463415
Intra-operator	LV_ ED Area.Lt ED Area % (HC)[%]	centile	<5	5	30	8	0.266667	0.402985
Intra-operator	LV_ ED Area.Lt ED Area % (HC)[%]	centile	>95	95	30	1	0.033333	0
Inter-operator	LV_ ED Length.Lt ED Length % (AC)[%]	centile	<5	5	56	16	0.285714	0.423423
Inter-operator	LV_ ED Length.Lt ED Length % (AC)[%]	centile	>95	95	56	0	0	
Intra-operator	LV_ ED Length.Lt ED Length % (AC)[%]	centile	<5	5	30	11	0.366667	0.285714
Intra-operator	LV_ ED Length.Lt ED Length % (AC)[%]	centile	>95	95	30	0	0	
Inter-operator	LV_ ED Length.Lt ED Length % (AUA)[%]	centile	<5	5	54	14	0.259259	0.476454
Inter-operator	LV_ ED Length.Lt ED Length % (AUA)[%]	centile	>95	95	54	0	0	
Intra-operator	LV_ ED Length.Lt ED Length % (AUA)[%]	centile	<5	5	30	12	0.4	0.210526
Intra-operator	LV_ ED Length.Lt ED Length % (AUA)[%]	centile	>95	95	30	0	0	
Inter-operator	LV_ ED Length.Lt ED Length % (BPD)[%]	centile	<5	5	55	15	0.272727	0.456163
Inter-operator	LV_ ED Length.Lt ED Length % (BPD)[%]	centile	>95	95	55	0	0	
Intra-operator	LV_ ED Length.Lt ED Length % (BPD)[%]	centile	<5	5	30	10	0.333333	0.333333
Intra-operator	LV_ ED Length.Lt ED Length % (BPD)[%]	centile	>95	95	30	0	0	
Inter-operator	LV_ ED Length.Lt ED Length % (EFW)[%]	centile	<5	5	55	15	0.272727	0.440678
Inter-operator	LV_ ED Length.Lt ED Length % (EFW)[%]	centile	>95	95	55	0	0	
Intra-operator	LV_ ED Length.Lt ED Length % (EFW)[%]	centile	<5	5	30	12	0.4	0.227468
Intra-operator	LV_ ED Length.Lt ED Length % (EFW)[%]	centile	>95	95	30	0	0	
Inter-operator	LV_ ED Length.Lt ED Length % (FL)[%]	centile	<5	5	55	14	0.254545	0.47973
Inter-operator	LV_ ED Length.Lt ED Length % (FL)[%]	centile	>95	95	55	0	0	
Intra-operator	LV_ ED Length.Lt ED Length % (FL)[%]	centile	<5	5	29	10	0.344828	0.32243
Intra-operator	LV_ ED Length.Lt ED Length % (FL)[%]	centile	>95	95	29	0	0	
Inter-operator	LV_ ED Length.Lt ED Length % (GA)[%]	centile	<5	5	51	11	0.215686	0.559309
Inter-operator	LV_ ED Length.Lt ED Length % (GA)[%]	centile	>95	95	51	0	0	
Intra-operator	LV_ ED Length.Lt ED Length % (GA)[%]	centile	<5	5	28	9	0.321429	0.363636
Intra-operator	LV_ ED Length.Lt ED Length % (GA)[%]	centile	>95	95	28	0	0	
Inter-operator	LV_ ED Length.Lt ED Length % (HC)[%]	centile	<5	5	55	15	0.272727	0.456163

group	parameter	scale	threshold	thr_value	usable_n	flips	flip_rate	kappa
Inter-operator	LV_ ED Length.Lt ED Length % (HC)[%]	centile	>95	95	55	0	0	
Intra-operator	LV_ ED Length.Lt ED Length % (HC)[%]	centile	<5	5	30	9	0.3	0.38914
Intra-operator	LV_ ED Length.Lt ED Length % (HC)[%]	centile	>95	95	30	0	0	
Inter-operator	LV_ EDV.Lt EDV % (AC)[%]	centile	<5	5	28	5	0.178571	0.615385
Inter-operator	LV_ EDV.Lt EDV % (AC)[%]	centile	>95	95	28	2	0.071429	0.626667
Intra-operator	LV_ EDV.Lt EDV % (AC)[%]	centile	<5	5	19	4	0.210526	0.547619
Intra-operator	LV_ EDV.Lt EDV % (AC)[%]	centile	>95	95	19	2	0.105263	0.441176
Inter-operator	LV_ EDV.Lt EDV % (AUA)[%]	centile	<5	5	54	14	0.259259	0.489879
Inter-operator	LV_ EDV.Lt EDV % (AUA)[%]	centile	>95	95	54	8	0.148148	0.414634
Intra-operator	LV_ EDV.Lt EDV % (AUA)[%]	centile	<5	5	30	8	0.266667	0.466667
Intra-operator	LV_ EDV.Lt EDV % (AUA)[%]	centile	>95	95	30	3	0.1	0.347826
Inter-operator	LV_ EDV.Lt EDV % (BPD)[%]	centile	<5	5	51	11	0.215686	0.512598
Inter-operator	LV_ EDV.Lt EDV % (BPD)[%]	centile	>95	95	51	8	0.156863	0.406977
Intra-operator	LV_ EDV.Lt EDV % (BPD)[%]	centile	<5	5	30	7	0.233333	0.524887
Intra-operator	LV_ EDV.Lt EDV % (BPD)[%]	centile	>95	95	30	1	0.033333	0.651163
Inter-operator	LV_ EDV.Lt EDV % (EFW)[%]	centile	<5	5	19	4	0.210526	0.377049
Inter-operator	LV_ EDV.Lt EDV % (EFW)[%]	centile	>95	95	19	1	0.052632	0.641509
Intra-operator	LV_ EDV.Lt EDV % (EFW)[%]	centile	<5	5	16	4	0.25	0.179487
Intra-operator	LV_ EDV.Lt EDV % (EFW)[%]	centile	>95	95	16	1	0.0625	0.764706
Inter-operator	LV_ EDV.Lt EDV % (FL)[%]	centile	<5	5	33	8	0.242424	0.352941
Inter-operator	LV_ EDV.Lt EDV % (FL)[%]	centile	>95	95	33	1	0.030303	0.84058
Intra-operator	LV_ EDV.Lt EDV % (FL)[%]	centile	<5	5	17	4	0.235294	0.392857
Intra-operator	LV_ EDV.Lt EDV % (FL)[%]	centile	>95	95	17	1	0.058824	0.638298
Inter-operator	LV_ EDV.Lt EDV % (GA)[%]	centile	<5	5	51	9	0.176471	0.598425
Inter-operator	LV_ EDV.Lt EDV % (GA)[%]	centile	>95	95	51	6	0.117647	0.337662
Intra-operator	LV_ EDV.Lt EDV % (GA)[%]	centile	<5	5	28	7	0.25	0.484211
Intra-operator	LV_ EDV.Lt EDV % (GA)[%]	centile	>95	95	28	0	0	1
Inter-operator	LV_ EDV.Lt EDV % (HC)[%]	centile	<5	5	14	0	0	1
Inter-operator	LV_ EDV.Lt EDV % (HC)[%]	centile	>95	95	14	4	0.285714	0.176471
Intra-operator	LV_ EDV.Lt EDV % (HC)[%]	centile	<5	5	13	3	0.230769	-0.114286
Intra-operator	LV_ EDV.Lt EDV % (HC)[%]	centile	>95	95	13	1	0.076923	0.628571
Inter-operator	LV_ EF.Lt EF % (AC)[%]	centile	<5	5	28	10	0.357143	0.181287
Inter-operator	LV_ EF.Lt EF % (AC)[%]	centile	>95	95	28	1	0.035714	0
Intra-operator	LV_ EF.Lt EF % (AC)[%]	centile	<5	5	19	2	0.105263	0.688525
Intra-operator	LV_ EF.Lt EF % (AC)[%]	centile	>95	95	19	0	0	
Inter-operator	LV_ EF.Lt EF % (AUA)[%]	centile	<5	5	54	17	0.314815	0.231156
Inter-operator	LV_ EF.Lt EF % (AUA)[%]	centile	>95	95	54	1	0.018519	0
Intra-operator	LV_ EF.Lt EF % (AUA)[%]	centile	<5	5	30	6	0.2	0.444444
Intra-operator	LV_ EF.Lt EF % (AUA)[%]	centile	>95	95	30	0	0	
Inter-operator	LV_ EF.Lt EF % (BPD)[%]	centile	<5	5	51	14	0.27451	0.277328

group	parameter	scale	threshold	thr_value	usable_n	flips	flip_rate	kappa
Inter-operator	LV_ EF.Lt EF % (BPD)[%]	centile	>95	95	51	2	0.039216	-0.02
Intra-operator	LV_ EF.Lt EF % (BPD)[%]	centile	<5	5	30	6	0.2	0.444444
Intra-operator	LV_ EF.Lt EF % (BPD)[%]	centile	>95	95	30	0	0	
Inter-operator	LV_ EF.Lt EF % (EFW)[%]	centile	<5	5	19	7	0.368421	0.184049
Inter-operator	LV_ EF.Lt EF % (EFW)[%]	centile	>95	95	19	1	0.052632	0
Intra-operator	LV_ EF.Lt EF % (EFW)[%]	centile	<5	5	16	1	0.0625	0.818182
Intra-operator	LV_ EF.Lt EF % (EFW)[%]	centile	>95	95	16	0	0	
Inter-operator	LV_ EF.Lt EF % (FL)[%]	centile	<5	5	33	14	0.424242	0.086957
Inter-operator	LV_ EF.Lt EF % (FL)[%]	centile	>95	95	33	1	0.030303	0
Intra-operator	LV_ EF.Lt EF % (FL)[%]	centile	<5	5	17	0	0	1
Intra-operator	LV_ EF.Lt EF % (FL)[%]	centile	>95	95	17	0	0	
Inter-operator	LV_ EF.Lt EF % (GA)[%]	centile	<5	5	51	17	0.333333	0.24147
Inter-operator	LV_ EF.Lt EF % (GA)[%]	centile	>95	95	51	2	0.039216	-0.02
Intra-operator	LV_ EF.Lt EF % (GA)[%]	centile	<5	5	28	5	0.178571	0.551282
Intra-operator	LV_ EF.Lt EF % (GA)[%]	centile	>95	95	28	0	0	
Inter-operator	LV_ EF.Lt EF % (HC)[%]	centile	<5	5	14	4	0.285714	0.317073
Inter-operator	LV_ EF.Lt EF % (HC)[%]	centile	>95	95	14	0	0	
Intra-operator	LV_ EF.Lt EF % (HC)[%]	centile	<5	5	13	1	0.076923	0.80597
Intra-operator	LV_ EF.Lt EF % (HC)[%]	centile	>95	95	13	0	0	
Inter-operator	LV_ Frac. Area change.Lt FRAC % (AC)[%]	centile	<5	5	56	23	0.410714	0.103064
Inter-operator	LV_ Frac. Area change.Lt FRAC % (AC)[%]	centile	>95	95	56	2	0.035714	-0.018182
Intra-operator	LV_ Frac. Area change.Lt FRAC % (AC)[%]	centile	<5	5	30	5	0.166667	0.561404
Intra-operator	LV_ Frac. Area change.Lt FRAC % (AC)[%]	centile	>95	95	30	0	0	
Inter-operator	LV_ Frac. Area change.Lt FRAC % (AUA)[%]	centile	<5	5	54	17	0.314815	0.26087
Inter-operator	LV_ Frac. Area change.Lt FRAC % (AUA)[%]	centile	>95	95	54	2	0.037037	-0.018868
Intra-operator	LV_ Frac. Area change.Lt FRAC % (AUA)[%]	centile	<5	5	30	5	0.166667	0.509804
Intra-operator	LV_ Frac. Area change.Lt FRAC % (AUA)[%]	centile	>95	95	30	0	0	
Inter-operator	LV_ Frac. Area change.Lt FRAC % (BPD)[%]	centile	<5	5	55	19	0.345455	0.211321
Inter-operator	LV_ Frac. Area change.Lt FRAC % (BPD)[%]	centile	>95	95	55	3	0.054545	-0.024845
Intra-operator	LV_ Frac. Area change.Lt FRAC % (BPD)[%]	centile	<5	5	30	5	0.166667	0.509804
Intra-operator	LV_ Frac. Area change.Lt FRAC % (BPD)[%]	centile	>95	95	30	0	0	
Inter-operator	LV_ Frac. Area change.Lt FRAC % (EFW)[%]	centile	<5	5	55	15	0.272727	0.328723
Inter-operator	LV_ Frac. Area change.Lt FRAC % (EFW)[%]	centile	>95	95	55	3	0.054545	-0.024845
Intra-operator	LV_ Frac. Area change.Lt FRAC % (EFW)[%]	centile	<5	5	30	6	0.2	0.37931
Intra-operator	LV_ Frac. Area change.Lt FRAC % (EFW)[%]	centile	>95	95	30	0	0	
Inter-operator	LV_ Frac. Area change.Lt FRAC % (FL)[%]	centile	<5	5	55	17	0.309091	0.264359
Inter-operator	LV_ Frac. Area change.Lt FRAC % (FL)[%]	centile	>95	95	55	2	0.036364	-0.018519
Intra-operator	LV_ Frac. Area change.Lt FRAC % (FL)[%]	centile	<5	5	29	4	0.137931	0.57971
Intra-operator	LV_ Frac. Area change.Lt FRAC % (FL)[%]	centile	>95	95	29	0	0	
Inter-operator	LV_ Frac. Area change.Lt FRAC % (GA)[%]	centile	<5	5	51	20	0.392157	0.141414

group	parameter	scale	threshold	thr_value	usable_n	flips	flip_rate	kappa
Inter-operator	LV_ Frac. Area change.Lt FRAC % (GA)[%]	centile	>95	95	51	3	0.058824	-0.026846
Intra-operator	LV_ Frac. Area change.Lt FRAC % (GA)[%]	centile	<5	5	28	5	0.178571	0.551282
Intra-operator	LV_ Frac. Area change.Lt FRAC % (GA)[%]	centile	>95	95	28	0	0	
Inter-operator	LV_ Frac. Area change.Lt FRAC % (HC)[%]	centile	<5	5	55	19	0.345455	0.211321
Inter-operator	LV_ Frac. Area change.Lt FRAC % (HC)[%]	centile	>95	95	55	2	0.036364	-0.018519
Intra-operator	LV_ Frac. Area change.Lt FRAC % (HC)[%]	centile	<5	5	30	4	0.133333	0.62963
Intra-operator	LV_ Frac. Area change.Lt FRAC % (HC)[%]	centile	>95	95	30	0	0	
Inter-operator	LV_ FreeWall Strain.Lt FWS %[%]	centile	<5	5	56	6	0.107143	0.196172
Inter-operator	LV_ FreeWall Strain.Lt FWS %[%]	centile	>95	95	56	17	0.303571	0.376963
Intra-operator	LV_ FreeWall Strain.Lt FWS %[%]	centile	<5	5	30	1	0.033333	0.651163
Intra-operator	LV_ FreeWall Strain.Lt FWS %[%]	centile	>95	95	30	9	0.3	0.341463
Inter-operator	LV_ FreeWall Strain.Lt FWS ZS[]	zscore	Z<-2	2	55	6	0.109091	-0.050955
Inter-operator	LV_ FreeWall Strain.Lt FWS ZS[]	zscore	Z>2	2	55	18	0.327273	0.345238
Inter-operator	LV_ FreeWall Strain.Lt FWS ZS[]	zscore	|Z|>1.645	1.645	55	17	0.309091	0.326854
Inter-operator	LV_ FreeWall Strain.Lt FWS ZS[]	zscore	|Z|>2.0	2	55	22	0.4	0.187919
Intra-operator	LV_ FreeWall Strain.Lt FWS ZS[]	zscore	Z<-2	2	27	1	0.037037	0
Intra-operator	LV_ FreeWall Strain.Lt FWS ZS[]	zscore	Z>2	2	27	8	0.296296	0.403315
Intra-operator	LV_ FreeWall Strain.Lt FWS ZS[]	zscore	|Z|>1.645	1.645	27	7	0.259259	0.4
Intra-operator	LV_ FreeWall Strain.Lt FWS ZS[]	zscore	|Z|>2.0	2	27	9	0.333333	0.319328
Inter-operator	LV_ Global Strain.Lt GLS %[%]	centile	<5	5	56	11	0.196429	0.153846
Inter-operator	LV_ Global Strain.Lt GLS %[%]	centile	>95	95	56	20	0.357143	0.282971
Intra-operator	LV_ Global Strain.Lt GLS %[%]	centile	<5	5	30	4	0.133333	0.268293
Intra-operator	LV_ Global Strain.Lt GLS %[%]	centile	>95	95	30	4	0.133333	0.684211
Inter-operator	LV_ Global Strain.Lt GLS ZS[]	zscore	Z<-2	2	51	10	0.196078	0.172078
Inter-operator	LV_ Global Strain.Lt GLS ZS[]	zscore	Z>2	2	51	20	0.392157	0.200627
Inter-operator	LV_ Global Strain.Lt GLS ZS[]	zscore	|Z|>1.645	1.645	51	27	0.529412	-0.144638
Inter-operator	LV_ Global Strain.Lt GLS ZS[]	zscore	|Z|>2.0	2	51	28	0.54902	-0.119122
Intra-operator	LV_ Global Strain.Lt GLS ZS[]	zscore	Z<-2	2	24	2	0.083333	0.466667
Intra-operator	LV_ Global Strain.Lt GLS ZS[]	zscore	Z>2	2	24	4	0.166667	0.659574
Intra-operator	LV_ Global Strain.Lt GLS ZS[]	zscore	|Z|>1.645	1.645	24	4	0.166667	0.571429
Intra-operator	LV_ Global Strain.Lt GLS ZS[]	zscore	|Z|>2.0	2	24	6	0.25	0.454545
Inter-operator	LV_ MAPSE lateral.MAPSE lat. % (AC)[%]	centile	<5	5	56	21	0.375	0.24031
Inter-operator	LV_ MAPSE lateral.MAPSE lat. % (AC)[%]	centile	>95	95	56	6	0.107143	-0.05
Intra-operator	LV_ MAPSE lateral.MAPSE lat. % (AC)[%]	centile	<5	5	30	9	0.3	0.347826
Intra-operator	LV_ MAPSE lateral.MAPSE lat. % (AC)[%]	centile	>95	95	30	4	0.133333	-0.052632
Inter-operator	LV_ MAPSE lateral.MAPSE lat. % (AUA)[%]	centile	<5	5	54	19	0.351852	0.286509
Inter-operator	LV_ MAPSE lateral.MAPSE lat. % (AUA)[%]	centile	>95	95	54	6	0.111111	-0.051948
Intra-operator	LV_ MAPSE lateral.MAPSE lat. % (AUA)[%]	centile	<5	5	30	10	0.333333	0.253731
Intra-operator	LV_ MAPSE lateral.MAPSE lat. % (AUA)[%]	centile	>95	95	30	4	0.133333	-0.052632
Inter-operator	LV_ MAPSE lateral.MAPSE lat. % (BPD)[%]	centile	<5	5	55	18	0.327273	0.328358

group	parameter	scale	threshold	thr_value	usable_n	flips	flip_rate	kappa
Inter-operator	LV_ MAPSE lateral.MAPSE lat. % (BPD)[%]	centile	>95	95	55	5	0.090909	-0.029963
Intra-operator	LV_ MAPSE lateral.MAPSE lat. % (BPD)[%]	centile	<5	5	30	10	0.333333	0.253731
Intra-operator	LV_ MAPSE lateral.MAPSE lat. % (BPD)[%]	centile	>95	95	30	4	0.133333	-0.052632
Inter-operator	LV_ MAPSE lateral.MAPSE lat. % (EFW)[%]	centile	<5	5	55	18	0.327273	0.346103
Inter-operator	LV_ MAPSE lateral.MAPSE lat. % (EFW)[%]	centile	>95	95	55	5	0.090909	-0.029963
Intra-operator	LV_ MAPSE lateral.MAPSE lat. % (EFW)[%]	centile	<5	5	30	10	0.333333	0.308756
Intra-operator	LV_ MAPSE lateral.MAPSE lat. % (EFW)[%]	centile	>95	95	30	4	0.133333	-0.052632
Inter-operator	LV_ MAPSE lateral.MAPSE lat. % (FL)[%]	centile	<5	5	56	21	0.375	0.24031
Inter-operator	LV_ MAPSE lateral.MAPSE lat. % (FL)[%]	centile	>95	95	56	5	0.089286	-0.029412
Intra-operator	LV_ MAPSE lateral.MAPSE lat. % (FL)[%]	centile	<5	5	29	8	0.275862	0.39267
Intra-operator	LV_ MAPSE lateral.MAPSE lat. % (FL)[%]	centile	>95	95	29	4	0.137931	-0.054545
Inter-operator	LV_ MAPSE lateral.MAPSE lat. % (GA)[%]	centile	<5	5	51	14	0.27451	0.427885
Inter-operator	LV_ MAPSE lateral.MAPSE lat. % (GA)[%]	centile	>95	95	51	5	0.098039	-0.032389
Intra-operator	LV_ MAPSE lateral.MAPSE lat. % (GA)[%]	centile	<5	5	28	10	0.357143	0.226519
Intra-operator	LV_ MAPSE lateral.MAPSE lat. % (GA)[%]	centile	>95	95	28	3	0.107143	0
Inter-operator	LV_ MAPSE lateral.MAPSE lat. % (HC)[%]	centile	<5	5	55	17	0.309091	0.360902
Inter-operator	LV_ MAPSE lateral.MAPSE lat. % (HC)[%]	centile	>95	95	55	6	0.109091	-0.050955
Intra-operator	LV_ MAPSE lateral.MAPSE lat. % (HC)[%]	centile	<5	5	30	9	0.3	0.307692
Intra-operator	LV_ MAPSE lateral.MAPSE lat. % (HC)[%]	centile	>95	95	30	4	0.133333	-0.052632
Inter-operator	LV_ MAPSE septal.MAPSE sept. % (AC)[%]	centile	<5	5	56	16	0.285714	0.304348
Inter-operator	LV_ MAPSE septal.MAPSE sept. % (AC)[%]	centile	>95	95	56	10	0.178571	0.270833
Intra-operator	LV_ MAPSE septal.MAPSE sept. % (AC)[%]	centile	<5	5	30	9	0.3	0.383562
Intra-operator	LV_ MAPSE septal.MAPSE sept. % (AC)[%]	centile	>95	95	30	4	0.133333	0.268293
Inter-operator	LV_ MAPSE septal.MAPSE sept. % (AUA)[%]	centile	<5	5	54	2	0.037037	-0.018868
Inter-operator	LV_ MAPSE septal.MAPSE sept. % (AUA)[%]	centile	>95	95	54	0	0	
Intra-operator	LV_ MAPSE septal.MAPSE sept. % (AUA)[%]	centile	<5	5	30	0	0	1
Intra-operator	LV_ MAPSE septal.MAPSE sept. % (AUA)[%]	centile	>95	95	30	1	0.033333	0
Inter-operator	LV_ MAPSE septal.MAPSE sept. % (BPD)[%]	centile	<5	5	55	14	0.254545	0.422789
Inter-operator	LV_ MAPSE septal.MAPSE sept. % (BPD)[%]	centile	>95	95	55	11	0.2	0.235145
Intra-operator	LV_ MAPSE septal.MAPSE sept. % (BPD)[%]	centile	<5	5	30	11	0.366667	0.253394
Intra-operator	LV_ MAPSE septal.MAPSE sept. % (BPD)[%]	centile	>95	95	30	5	0.166667	0.193548
Inter-operator	LV_ MAPSE septal.MAPSE sept. % (EFW)[%]	centile	<5	5	55	12	0.218182	0.453642
Inter-operator	LV_ MAPSE septal.MAPSE sept. % (EFW)[%]	centile	>95	95	55	10	0.181818	0.268617
Intra-operator	LV_ MAPSE septal.MAPSE sept. % (EFW)[%]	centile	<5	5	30	11	0.366667	0.253394
Intra-operator	LV_ MAPSE septal.MAPSE sept. % (EFW)[%]	centile	>95	95	30	4	0.133333	0.268293
Inter-operator	LV_ MAPSE septal.MAPSE sept. % (FL)[%]	centile	<5	5	56	15	0.267857	0.396552
Inter-operator	LV_ MAPSE septal.MAPSE sept. % (FL)[%]	centile	>95	95	56	11	0.196429	0.237624
Intra-operator	LV_ MAPSE septal.MAPSE sept. % (FL)[%]	centile	<5	5	29	12	0.413793	0.179245
Intra-operator	LV_ MAPSE septal.MAPSE sept. % (FL)[%]	centile	>95	95	29	3	0.103448	0.345865
Inter-operator	LV_ MAPSE septal.MAPSE sept. % (GA)[%]	centile	<5	5	51	16	0.313725	0.298969

group	parameter	scale	threshold	thr_value	usable_n	flips	flip_rate	kappa
Inter-operator	LV_ MAPSE septal.MAPSE sept. % (GA)[%]	centile	>95	95	51	12	0.235294	0.198953
Intra-operator	LV_ MAPSE septal.MAPSE sept. % (GA)[%]	centile	<5	5	28	12	0.428571	0.120419
Intra-operator	LV_ MAPSE septal.MAPSE sept. % (GA)[%]	centile	>95	95	28	5	0.178571	0.186047
Inter-operator	LV_ MAPSE septal.MAPSE sept. % (HC)[%]	centile	<5	5	55	15	0.272727	0.373576
Inter-operator	LV_ MAPSE septal.MAPSE sept. % (HC)[%]	centile	>95	95	55	11	0.2	0.300578
Intra-operator	LV_ MAPSE septal.MAPSE sept. % (HC)[%]	centile	<5	5	30	11	0.366667	0.266667
Intra-operator	LV_ MAPSE septal.MAPSE sept. % (HC)[%]	centile	>95	95	30	5	0.166667	0.193548
Inter-operator	LV_ SV.Lt SV % (AC)[%]	centile	<5	5	28	10	0.357143	0.28934
Inter-operator	LV_ SV.Lt SV % (AC)[%]	centile	>95	95	28	0	0	1
Intra-operator	LV_ SV.Lt SV % (AC)[%]	centile	<5	5	19	2	0.105263	0.75641
Intra-operator	LV_ SV.Lt SV % (AC)[%]	centile	>95	95	19	0	0	
Inter-operator	LV_ SV.Lt SV % (AUA)[%]	centile	<5	5	54	14	0.259259	0.326203
Inter-operator	LV_ SV.Lt SV % (AUA)[%]	centile	>95	95	54	3	0.055556	0.372093
Intra-operator	LV_ SV.Lt SV % (AUA)[%]	centile	<5	5	30	5	0.166667	0.509804
Intra-operator	LV_ SV.Lt SV % (AUA)[%]	centile	>95	95	30	0	0	
Inter-operator	LV_ SV.Lt SV % (BPD)[%]	centile	<5	5	51	8	0.156863	0.606936
Inter-operator	LV_ SV.Lt SV % (BPD)[%]	centile	>95	95	51	2	0.039216	0.811808
Intra-operator	LV_ SV.Lt SV % (BPD)[%]	centile	<5	5	30	1	0.033333	0.901961
Intra-operator	LV_ SV.Lt SV % (BPD)[%]	centile	>95	95	30	1	0.033333	0.782609
Inter-operator	LV_ SV.Lt SV % (EFW)[%]	centile	<5	5	19	7	0.368421	0.231214
Inter-operator	LV_ SV.Lt SV % (EFW)[%]	centile	>95	95	19	0	0	1
Intra-operator	LV_ SV.Lt SV % (EFW)[%]	centile	<5	5	16	2	0.125	0.75
Intra-operator	LV_ SV.Lt SV % (EFW)[%]	centile	>95	95	16	0	0	
Inter-operator	LV_ SV.Lt SV % (FL)[%]	centile	<5	5	33	10	0.30303	0.388889
Inter-operator	LV_ SV.Lt SV % (FL)[%]	centile	>95	95	33	2	0.060606	0
Intra-operator	LV_ SV.Lt SV % (FL)[%]	centile	<5	5	17	4	0.235294	0.521127
Intra-operator	LV_ SV.Lt SV % (FL)[%]	centile	>95	95	17	0	0	
Inter-operator	LV_ SV.Lt SV % (GA)[%]	centile	<5	5	51	18	0.352941	0.26087
Inter-operator	LV_ SV.Lt SV % (GA)[%]	centile	>95	95	51	3	0.058824	0.37037
Intra-operator	LV_ SV.Lt SV % (GA)[%]	centile	<5	5	28	3	0.107143	0.746988
Intra-operator	LV_ SV.Lt SV % (GA)[%]	centile	>95	95	28	0	0	
Inter-operator	LV_ SV.Lt SV % (HC)[%]	centile	<5	5	14	3	0.214286	0.571429
Inter-operator	LV_ SV.Lt SV % (HC)[%]	centile	>95	95	14	0	0	1
Intra-operator	LV_ SV.Lt SV % (HC)[%]	centile	<5	5	13	3	0.230769	0.493506
Intra-operator	LV_ SV.Lt SV % (HC)[%]	centile	>95	95	13	0	0	
Inter-operator	LV_ SV/KG.Lt SV/KG % (AC)[%]	centile	<5	5	27	10	0.37037	0.274194
Inter-operator	LV_ SV/KG.Lt SV/KG % (AC)[%]	centile	>95	95	27	0	0	1
Intra-operator	LV_ SV/KG.Lt SV/KG % (AC)[%]	centile	<5	5	19	2	0.105263	0.728571
Intra-operator	LV_ SV/KG.Lt SV/KG % (AC)[%]	centile	>95	95	19	0	0	
Inter-operator	LV_ SV/KG.Lt SV/KG % (AUA)[%]	centile	<5	5	53	19	0.358491	0.291344

group	parameter	scale	threshold	thr_value	usable_n	flips	flip_rate	kappa
Inter-operator	LV_ SV/KG.Lt SV/KG % (AUA)[%]	centile	>95	95	53	3	0.056604	0.371542
Intra-operator	LV_ SV/KG.Lt SV/KG % (AUA)[%]	centile	<5	5	30	9	0.3	0.341463
Intra-operator	LV_ SV/KG.Lt SV/KG % (AUA)[%]	centile	>95	95	30	0	0	
Inter-operator	LV_ SV/KG.Lt SV/KG % (BPD)[%]	centile	<5	5	51	19	0.372549	0.257471
Inter-operator	LV_ SV/KG.Lt SV/KG % (BPD)[%]	centile	>95	95	51	5	0.098039	0.234234
Intra-operator	LV_ SV/KG.Lt SV/KG % (BPD)[%]	centile	<5	5	30	10	0.333333	0.253731
Intra-operator	LV_ SV/KG.Lt SV/KG % (BPD)[%]	centile	>95	95	30	0	0	
Inter-operator	LV_ SV/KG.Lt SV/KG % (EFW)[%]	centile	<5	5	19	5	0.263158	0.475138
Inter-operator	LV_ SV/KG.Lt SV/KG % (EFW)[%]	centile	>95	95	19	0	0	1
Intra-operator	LV_ SV/KG.Lt SV/KG % (EFW)[%]	centile	<5	5	16	2	0.125	0.709091
Intra-operator	LV_ SV/KG.Lt SV/KG % (EFW)[%]	centile	>95	95	16	0	0	
Inter-operator	LV_ SV/KG.Lt SV/KG % (FL)[%]	centile	<5	5	32	12	0.375	0.23506
Inter-operator	LV_ SV/KG.Lt SV/KG % (FL)[%]	centile	>95	95	32	1	0.03125	0.652174
Intra-operator	LV_ SV/KG.Lt SV/KG % (FL)[%]	centile	<5	5	17	3	0.176471	0.627737
Intra-operator	LV_ SV/KG.Lt SV/KG % (FL)[%]	centile	>95	95	17	0	0	
Inter-operator	LV_ SV/KG.Lt SV/KG % (GA)[%]	centile	<5	5	50	17	0.34	0.304419
Inter-operator	LV_ SV/KG.Lt SV/KG % (GA)[%]	centile	>95	95	50	5	0.1	0.233129
Intra-operator	LV_ SV/KG.Lt SV/KG % (GA)[%]	centile	<5	5	28	8	0.285714	0.381215
Intra-operator	LV_ SV/KG.Lt SV/KG % (GA)[%]	centile	>95	95	28	0	0	
Inter-operator	LV_ SV/KG.Lt SV/KG % (HC)[%]	centile	<5	5	14	5	0.357143	0.285714
Inter-operator	LV_ SV/KG.Lt SV/KG % (HC)[%]	centile	>95	95	14	0	0	1
Intra-operator	LV_ SV/KG.Lt SV/KG % (HC)[%]	centile	<5	5	13	3	0.230769	0.541176
Intra-operator	LV_ SV/KG.Lt SV/KG % (HC)[%]	centile	>95	95	13	0	0	
Inter-operator	LV_ Sept.Wall Strain.Lt SWS %[%]	centile	<5	5	56	9	0.160714	0.231707
Inter-operator	LV_ Sept.Wall Strain.Lt SWS %[%]	centile	>95	95	56	20	0.357143	0.270833
Intra-operator	LV_ Sept.Wall Strain.Lt SWS %[%]	centile	<5	5	30	5	0.166667	-0.086957
Intra-operator	LV_ Sept.Wall Strain.Lt SWS %[%]	centile	>95	95	30	5	0.166667	0.657534
Inter-operator	LV_ Sept.Wall Strain.Lt SWS ZS[]	zscore	Z<-2	2	56	9	0.160714	0.1
Inter-operator	LV_ Sept.Wall Strain.Lt SWS ZS[]	zscore	Z>2	2	56	18	0.321429	0.326203
Inter-operator	LV_ Sept.Wall Strain.Lt SWS ZS[]	zscore	|Z|>1.645	1.645	56	27	0.482143	0.035714
Inter-operator	LV_ Sept.Wall Strain.Lt SWS ZS[]	zscore	|Z|>2.0	2	56	27	0.482143	0.038168
Intra-operator	LV_ Sept.Wall Strain.Lt SWS ZS[]	zscore	Z<-2	2	29	3	0.103448	-0.048193
Intra-operator	LV_ Sept.Wall Strain.Lt SWS ZS[]	zscore	Z>2	2	29	8	0.275862	0.447619
Intra-operator	LV_ Sept.Wall Strain.Lt SWS ZS[]	zscore	|Z|>1.645	1.645	29	8	0.275862	0.39267
Intra-operator	LV_ Sept.Wall Strain.Lt SWS ZS[]	zscore	|Z|>2.0	2	29	9	0.310345	0.368039
Inter-operator	RV 24-Segment FS Centiles[%] Segment 1	centile	<5	5	56	18	0.321429	0.328895
Inter-operator	RV 24-Segment FS Centiles[%] Segment 1	centile	>95	95	56	4	0.071429	0.3
Intra-operator	RV 24-Segment FS Centiles[%] Segment 1	centile	<5	5	30	12	0.4	0.117647
Intra-operator	RV 24-Segment FS Centiles[%] Segment 1	centile	>95	95	30	2	0.066667	0
Inter-operator	RV 24-Segment FS Centiles[%] Segment 10	centile	<5	5	56	20	0.357143	0.278351

group	parameter	scale	threshold	thr_value	usable_n	flips	flip_rate	kappa
Inter-operator	RV 24-Segment FS Centiles[%] Segment 10	centile	>95	95	56	1	0.017857	0.658537
Intra-operator	RV 24-Segment FS Centiles[%] Segment 10	centile	<5	5	30	12	0.4	0.224138
Intra-operator	RV 24-Segment FS Centiles[%] Segment 10	centile	>95	95	30	0	0	
Inter-operator	RV 24-Segment FS Centiles[%] Segment 11	centile	<5	5	56	20	0.357143	0.266055
Inter-operator	RV 24-Segment FS Centiles[%] Segment 11	centile	>95	95	56	1	0.017857	0.658537
Intra-operator	RV 24-Segment FS Centiles[%] Segment 11	centile	<5	5	30	11	0.366667	0.179104
Intra-operator	RV 24-Segment FS Centiles[%] Segment 11	centile	>95	95	30	0	0	
Inter-operator	RV 24-Segment FS Centiles[%] Segment 12	centile	<5	5	56	22	0.392857	0.19896
Inter-operator	RV 24-Segment FS Centiles[%] Segment 12	centile	>95	95	56	1	0.017857	0.658537
Intra-operator	RV 24-Segment FS Centiles[%] Segment 12	centile	<5	5	30	10	0.333333	0.222798
Intra-operator	RV 24-Segment FS Centiles[%] Segment 12	centile	>95	95	30	0	0	
Inter-operator	RV 24-Segment FS Centiles[%] Segment 13	centile	<5	5	56	21	0.375	0.244216
Inter-operator	RV 24-Segment FS Centiles[%] Segment 13	centile	>95	95	56	1	0.017857	0.658537
Intra-operator	RV 24-Segment FS Centiles[%] Segment 13	centile	<5	5	30	12	0.4	0.104478
Intra-operator	RV 24-Segment FS Centiles[%] Segment 13	centile	>95	95	30	0	0	
Inter-operator	RV 24-Segment FS Centiles[%] Segment 14	centile	<5	5	56	16	0.285714	0.427842
Inter-operator	RV 24-Segment FS Centiles[%] Segment 14	centile	>95	95	56	2	0.035714	0
Intra-operator	RV 24-Segment FS Centiles[%] Segment 14	centile	<5	5	30	13	0.433333	0.084507
Intra-operator	RV 24-Segment FS Centiles[%] Segment 14	centile	>95	95	30	0	0	
Inter-operator	RV 24-Segment FS Centiles[%] Segment 15	centile	<5	5	56	14	0.25	0.5
Inter-operator	RV 24-Segment FS Centiles[%] Segment 15	centile	>95	95	56	2	0.035714	0
Intra-operator	RV 24-Segment FS Centiles[%] Segment 15	centile	<5	5	30	13	0.433333	0.109589
Intra-operator	RV 24-Segment FS Centiles[%] Segment 15	centile	>95	95	30	0	0	
Inter-operator	RV 24-Segment FS Centiles[%] Segment 16	centile	<5	5	56	12	0.214286	0.573604
Inter-operator	RV 24-Segment FS Centiles[%] Segment 16	centile	>95	95	56	3	0.053571	0
Intra-operator	RV 24-Segment FS Centiles[%] Segment 16	centile	<5	5	30	11	0.366667	0.26009
Intra-operator	RV 24-Segment FS Centiles[%] Segment 16	centile	>95	95	30	0	0	
Inter-operator	RV 24-Segment FS Centiles[%] Segment 17	centile	<5	5	56	11	0.196429	0.610127
Inter-operator	RV 24-Segment FS Centiles[%] Segment 17	centile	>95	95	56	3	0.053571	0
Intra-operator	RV 24-Segment FS Centiles[%] Segment 17	centile	<5	5	30	13	0.433333	0.140969
Intra-operator	RV 24-Segment FS Centiles[%] Segment 17	centile	>95	95	30	0	0	
Inter-operator	RV 24-Segment FS Centiles[%] Segment 18	centile	<5	5	56	12	0.214286	0.56701
Inter-operator	RV 24-Segment FS Centiles[%] Segment 18	centile	>95	95	56	2	0.035714	0
Intra-operator	RV 24-Segment FS Centiles[%] Segment 18	centile	<5	5	30	14	0.466667	0.070796
Intra-operator	RV 24-Segment FS Centiles[%] Segment 18	centile	>95	95	30	0	0	
Inter-operator	RV 24-Segment FS Centiles[%] Segment 19	centile	<5	5	56	14	0.25	0.503169
Inter-operator	RV 24-Segment FS Centiles[%] Segment 19	centile	>95	95	56	3	0.053571	-0.02439
Intra-operator	RV 24-Segment FS Centiles[%] Segment 19	centile	<5	5	30	8	0.266667	0.459459
Intra-operator	RV 24-Segment FS Centiles[%] Segment 19	centile	>95	95	30	0	0	
Inter-operator	RV 24-Segment FS Centiles[%] Segment 2	centile	<5	5	56	20	0.357143	0.247312

group	parameter	scale	threshold	thr_value	usable_n	flips	flip_rate	kappa
Inter-operator	RV 24-Segment FS Centiles[%] Segment 2	centile	>95	95	56	3	0.053571	0.382353
Intra-operator	RV 24-Segment FS Centiles[%] Segment 2	centile	<5	5	30	13	0.433333	0.133333
Intra-operator	RV 24-Segment FS Centiles[%] Segment 2	centile	>95	95	30	2	0.066667	0
Inter-operator	RV 24-Segment FS Centiles[%] Segment 20	centile	<5	5	56	13	0.232143	0.539241
Inter-operator	RV 24-Segment FS Centiles[%] Segment 20	centile	>95	95	56	2	0.035714	0.486239
Intra-operator	RV 24-Segment FS Centiles[%] Segment 20	centile	<5	5	30	9	0.3	0.4
Intra-operator	RV 24-Segment FS Centiles[%] Segment 20	centile	>95	95	30	0	0	
Inter-operator	RV 24-Segment FS Centiles[%] Segment 21	centile	<5	5	56	13	0.232143	0.538071
Inter-operator	RV 24-Segment FS Centiles[%] Segment 21	centile	>95	95	56	1	0.017857	0.658537
Intra-operator	RV 24-Segment FS Centiles[%] Segment 21	centile	<5	5	30	9	0.3	0.4
Intra-operator	RV 24-Segment FS Centiles[%] Segment 21	centile	>95	95	30	0	0	
Inter-operator	RV 24-Segment FS Centiles[%] Segment 22	centile	<5	5	56	15	0.267857	0.468354
Inter-operator	RV 24-Segment FS Centiles[%] Segment 22	centile	>95	95	56	2	0.035714	-0.018182
Intra-operator	RV 24-Segment FS Centiles[%] Segment 22	centile	<5	5	30	7	0.233333	0.533333
Intra-operator	RV 24-Segment FS Centiles[%] Segment 22	centile	>95	95	30	0	0	
Inter-operator	RV 24-Segment FS Centiles[%] Segment 23	centile	<5	5	56	15	0.267857	0.465649
Inter-operator	RV 24-Segment FS Centiles[%] Segment 23	centile	>95	95	56	2	0.035714	-0.018182
Intra-operator	RV 24-Segment FS Centiles[%] Segment 23	centile	<5	5	30	6	0.2	0.594595
Intra-operator	RV 24-Segment FS Centiles[%] Segment 23	centile	>95	95	30	0	0	
Inter-operator	RV 24-Segment FS Centiles[%] Segment 24	centile	<5	5	56	15	0.267857	0.468354
Inter-operator	RV 24-Segment FS Centiles[%] Segment 24	centile	>95	95	56	2	0.035714	-0.018182
Intra-operator	RV 24-Segment FS Centiles[%] Segment 24	centile	<5	5	30	6	0.2	0.594595
Intra-operator	RV 24-Segment FS Centiles[%] Segment 24	centile	>95	95	30	0	0	
Inter-operator	RV 24-Segment FS Centiles[%] Segment 3	centile	<5	5	56	17	0.303571	0.347945
Inter-operator	RV 24-Segment FS Centiles[%] Segment 3	centile	>95	95	56	4	0.071429	0.3
Intra-operator	RV 24-Segment FS Centiles[%] Segment 3	centile	<5	5	30	13	0.433333	0.148472
Intra-operator	RV 24-Segment FS Centiles[%] Segment 3	centile	>95	95	30	2	0.066667	0
Inter-operator	RV 24-Segment FS Centiles[%] Segment 4	centile	<5	5	56	17	0.303571	0.358491
Inter-operator	RV 24-Segment FS Centiles[%] Segment 4	centile	>95	95	56	5	0.089286	0.23913
Intra-operator	RV 24-Segment FS Centiles[%] Segment 4	centile	<5	5	30	18	0.6	-0.153846
Intra-operator	RV 24-Segment FS Centiles[%] Segment 4	centile	>95	95	30	1	0.033333	0
Inter-operator	RV 24-Segment FS Centiles[%] Segment 5	centile	<5	5	56	17	0.303571	0.376963
Inter-operator	RV 24-Segment FS Centiles[%] Segment 5	centile	>95	95	56	5	0.089286	0.23913
Intra-operator	RV 24-Segment FS Centiles[%] Segment 5	centile	<5	5	30	17	0.566667	-0.075949
Intra-operator	RV 24-Segment FS Centiles[%] Segment 5	centile	>95	95	30	1	0.033333	0
Inter-operator	RV 24-Segment FS Centiles[%] Segment 6	centile	<5	5	56	20	0.357143	0.278351
Inter-operator	RV 24-Segment FS Centiles[%] Segment 6	centile	>95	95	56	4	0.071429	0.3
Intra-operator	RV 24-Segment FS Centiles[%] Segment 6	centile	<5	5	30	15	0.5	0.074074
Intra-operator	RV 24-Segment FS Centiles[%] Segment 6	centile	>95	95	30	1	0.033333	0
Inter-operator	RV 24-Segment FS Centiles[%] Segment 7	centile	<5	5	56	22	0.392857	0.217281

group	parameter	scale	threshold	thr_value	usable_n	flips	flip_rate	kappa
Inter-operator	RV 24-Segment FS Centiles[%] Segment 7	centile	>95	95	56	5	0.089286	0.23913
Intra-operator	RV 24-Segment FS Centiles[%] Segment 7	centile	<5	5	30	13	0.433333	0.170213
Intra-operator	RV 24-Segment FS Centiles[%] Segment 7	centile	>95	95	30	1	0.033333	0
Inter-operator	RV 24-Segment FS Centiles[%] Segment 8	centile	<5	5	56	23	0.410714	0.176471
Inter-operator	RV 24-Segment FS Centiles[%] Segment 8	centile	>95	95	56	2	0.035714	0.481481
Intra-operator	RV 24-Segment FS Centiles[%] Segment 8	centile	<5	5	30	14	0.466667	0.113924
Intra-operator	RV 24-Segment FS Centiles[%] Segment 8	centile	>95	95	30	1	0.033333	0
Inter-operator	RV 24-Segment FS Centiles[%] Segment 9	centile	<5	5	56	23	0.410714	0.172237
Inter-operator	RV 24-Segment FS Centiles[%] Segment 9	centile	>95	95	56	1	0.017857	0.658537
Intra-operator	RV 24-Segment FS Centiles[%] Segment 9	centile	<5	5	30	13	0.433333	0.16309
Intra-operator	RV 24-Segment FS Centiles[%] Segment 9	centile	>95	95	30	0	0	
Inter-operator	RV 24-Segment SI Centiles[%] Segment 1	centile	<5	5	56	17	0.303571	0.091603
Inter-operator	RV 24-Segment SI Centiles[%] Segment 1	centile	>95	95	56	4	0.071429	-0.027523
Intra-operator	RV 24-Segment SI Centiles[%] Segment 1	centile	<5	5	30	8	0.266667	0.294118
Intra-operator	RV 24-Segment SI Centiles[%] Segment 1	centile	>95	95	30	2	0.066667	-0.034483
Inter-operator	RV 24-Segment SI Centiles[%] Segment 10	centile	<5	5	56	13	0.232143	0.1875
Inter-operator	RV 24-Segment SI Centiles[%] Segment 10	centile	>95	95	56	4	0.071429	0.3
Intra-operator	RV 24-Segment SI Centiles[%] Segment 10	centile	<5	5	30	9	0.3	-0.062992
Intra-operator	RV 24-Segment SI Centiles[%] Segment 10	centile	>95	95	30	4	0.133333	-0.071429
Inter-operator	RV 24-Segment SI Centiles[%] Segment 11	centile	<5	5	56	12	0.214286	0.222222
Inter-operator	RV 24-Segment SI Centiles[%] Segment 11	centile	>95	95	56	3	0.053571	0.548387
Intra-operator	RV 24-Segment SI Centiles[%] Segment 11	centile	<5	5	30	8	0.266667	0
Intra-operator	RV 24-Segment SI Centiles[%] Segment 11	centile	>95	95	30	4	0.133333	-0.071429
Inter-operator	RV 24-Segment SI Centiles[%] Segment 12	centile	<5	5	56	10	0.178571	0.278351
Inter-operator	RV 24-Segment SI Centiles[%] Segment 12	centile	>95	95	56	5	0.089286	0.401709
Intra-operator	RV 24-Segment SI Centiles[%] Segment 12	centile	<5	5	30	6	0.2	0
Intra-operator	RV 24-Segment SI Centiles[%] Segment 12	centile	>95	95	30	4	0.133333	0.259259
Inter-operator	RV 24-Segment SI Centiles[%] Segment 13	centile	<5	5	56	12	0.214286	0.127273
Inter-operator	RV 24-Segment SI Centiles[%] Segment 13	centile	>95	95	56	4	0.071429	0.464115
Intra-operator	RV 24-Segment SI Centiles[%] Segment 13	centile	<5	5	30	6	0.2	0
Intra-operator	RV 24-Segment SI Centiles[%] Segment 13	centile	>95	95	30	3	0.1	0.347826
Inter-operator	RV 24-Segment SI Centiles[%] Segment 14	centile	<5	5	56	10	0.178571	0.183673
Inter-operator	RV 24-Segment SI Centiles[%] Segment 14	centile	>95	95	56	5	0.089286	0.401709
Intra-operator	RV 24-Segment SI Centiles[%] Segment 14	centile	<5	5	30	6	0.2	0
Intra-operator	RV 24-Segment SI Centiles[%] Segment 14	centile	>95	95	30	3	0.1	0.347826
Inter-operator	RV 24-Segment SI Centiles[%] Segment 15	centile	<5	5	56	9	0.160714	0.217391
Inter-operator	RV 24-Segment SI Centiles[%] Segment 15	centile	>95	95	56	7	0.125	0.294964
Intra-operator	RV 24-Segment SI Centiles[%] Segment 15	centile	<5	5	30	5	0.166667	0
Intra-operator	RV 24-Segment SI Centiles[%] Segment 15	centile	>95	95	30	4	0.133333	-0.052632
Inter-operator	RV 24-Segment SI Centiles[%] Segment 16	centile	<5	5	56	8	0.142857	0.121569

group	parameter	scale	threshold	thr_value	usable_n	flips	flip_rate	kappa
Inter-operator	RV 24-Segment SI Centiles[%] Segment 16	centile	>95	95	56	7	0.125	0.294964
Intra-operator	RV 24-Segment SI Centiles[%] Segment 16	centile	<5	5	30	4	0.133333	0
Intra-operator	RV 24-Segment SI Centiles[%] Segment 16	centile	>95	95	30	3	0.1	-0.046512
Inter-operator	RV 24-Segment SI Centiles[%] Segment 17	centile	<5	5	56	6	0.107143	0.192308
Inter-operator	RV 24-Segment SI Centiles[%] Segment 17	centile	>95	95	56	7	0.125	0.294964
Intra-operator	RV 24-Segment SI Centiles[%] Segment 17	centile	<5	5	30	4	0.133333	0
Intra-operator	RV 24-Segment SI Centiles[%] Segment 17	centile	>95	95	30	3	0.1	-0.046512
Inter-operator	RV 24-Segment SI Centiles[%] Segment 18	centile	<5	5	56	5	0.089286	0.23913
Inter-operator	RV 24-Segment SI Centiles[%] Segment 18	centile	>95	95	56	8	0.142857	0.121569
Intra-operator	RV 24-Segment SI Centiles[%] Segment 18	centile	<5	5	30	5	0.166667	-0.056338
Intra-operator	RV 24-Segment SI Centiles[%] Segment 18	centile	>95	95	30	4	0.133333	-0.071429
Inter-operator	RV 24-Segment SI Centiles[%] Segment 19	centile	<5	5	56	5	0.089286	-0.044776
Inter-operator	RV 24-Segment SI Centiles[%] Segment 19	centile	>95	95	56	9	0.160714	0.093525
Intra-operator	RV 24-Segment SI Centiles[%] Segment 19	centile	<5	5	30	4	0.133333	-0.052632
Intra-operator	RV 24-Segment SI Centiles[%] Segment 19	centile	>95	95	30	4	0.133333	-0.052632
Inter-operator	RV 24-Segment SI Centiles[%] Segment 2	centile	<5	5	56	14	0.25	0.214429
Inter-operator	RV 24-Segment SI Centiles[%] Segment 2	centile	>95	95	56	4	0.071429	-0.027523
Intra-operator	RV 24-Segment SI Centiles[%] Segment 2	centile	<5	5	30	8	0.266667	0.294118
Intra-operator	RV 24-Segment SI Centiles[%] Segment 2	centile	>95	95	30	2	0.066667	-0.034483
Inter-operator	RV 24-Segment SI Centiles[%] Segment 20	centile	<5	5	56	4	0.071429	-0.037037
Inter-operator	RV 24-Segment SI Centiles[%] Segment 20	centile	>95	95	56	10	0.178571	0.066667
Intra-operator	RV 24-Segment SI Centiles[%] Segment 20	centile	<5	5	30	4	0.133333	-0.052632
Intra-operator	RV 24-Segment SI Centiles[%] Segment 20	centile	>95	95	30	6	0.2	-0.097561
Inter-operator	RV 24-Segment SI Centiles[%] Segment 21	centile	<5	5	56	4	0.071429	-0.037037
Inter-operator	RV 24-Segment SI Centiles[%] Segment 21	centile	>95	95	56	11	0.196429	0.043478
Intra-operator	RV 24-Segment SI Centiles[%] Segment 21	centile	<5	5	30	4	0.133333	-0.052632
Intra-operator	RV 24-Segment SI Centiles[%] Segment 21	centile	>95	95	30	7	0.233333	-0.129032
Inter-operator	RV 24-Segment SI Centiles[%] Segment 22	centile	<5	5	56	2	0.035714	0
Inter-operator	RV 24-Segment SI Centiles[%] Segment 22	centile	>95	95	56	8	0.142857	0.121569
Intra-operator	RV 24-Segment SI Centiles[%] Segment 22	centile	<5	5	30	3	0.1	-0.046512
Intra-operator	RV 24-Segment SI Centiles[%] Segment 22	centile	>95	95	30	7	0.233333	-0.129032
Inter-operator	RV 24-Segment SI Centiles[%] Segment 23	centile	<5	5	56	1	0.017857	0
Inter-operator	RV 24-Segment SI Centiles[%] Segment 23	centile	>95	95	56	8	0.142857	0.125
Intra-operator	RV 24-Segment SI Centiles[%] Segment 23	centile	<5	5	30	3	0.1	-0.046512
Intra-operator	RV 24-Segment SI Centiles[%] Segment 23	centile	>95	95	30	7	0.233333	-0.129032
Inter-operator	RV 24-Segment SI Centiles[%] Segment 24	centile	<5	5	56	1	0.017857	0
Inter-operator	RV 24-Segment SI Centiles[%] Segment 24	centile	>95	95	56	8	0.142857	0.125
Intra-operator	RV 24-Segment SI Centiles[%] Segment 24	centile	<5	5	30	3	0.1	-0.046512
Intra-operator	RV 24-Segment SI Centiles[%] Segment 24	centile	>95	95	30	7	0.233333	-0.129032
Inter-operator	RV 24-Segment SI Centiles[%] Segment 3	centile	<5	5	56	16	0.285714	0.153119

group	parameter	scale	threshold	thr_value	usable_n	flips	flip_rate	kappa
Inter-operator	RV 24-Segment SI Centiles[%] Segment 3	centile	>95	95	56	4	0.071429	-0.027523
Intra-operator	RV 24-Segment SI Centiles[%] Segment 3	centile	<5	5	30	8	0.266667	0.294118
Intra-operator	RV 24-Segment SI Centiles[%] Segment 3	centile	>95	95	30	3	0.1	-0.046512
Inter-operator	RV 24-Segment SI Centiles[%] Segment 4	centile	<5	5	56	16	0.285714	0.153119
Inter-operator	RV 24-Segment SI Centiles[%] Segment 4	centile	>95	95	56	4	0.071429	-0.027523
Intra-operator	RV 24-Segment SI Centiles[%] Segment 4	centile	<5	5	30	10	0.333333	0.166667
Intra-operator	RV 24-Segment SI Centiles[%] Segment 4	centile	>95	95	30	3	0.1	-0.046512
Inter-operator	RV 24-Segment SI Centiles[%] Segment 5	centile	<5	5	56	16	0.285714	0.153119
Inter-operator	RV 24-Segment SI Centiles[%] Segment 5	centile	>95	95	56	5	0.089286	-0.044776
Intra-operator	RV 24-Segment SI Centiles[%] Segment 5	centile	<5	5	30	10	0.333333	0.166667
Intra-operator	RV 24-Segment SI Centiles[%] Segment 5	centile	>95	95	30	4	0.133333	-0.071429
Inter-operator	RV 24-Segment SI Centiles[%] Segment 6	centile	<5	5	56	16	0.285714	0.153119
Inter-operator	RV 24-Segment SI Centiles[%] Segment 6	centile	>95	95	56	5	0.089286	-0.044776
Intra-operator	RV 24-Segment SI Centiles[%] Segment 6	centile	<5	5	30	10	0.333333	0.166667
Intra-operator	RV 24-Segment SI Centiles[%] Segment 6	centile	>95	95	30	3	0.1	-0.046512
Inter-operator	RV 24-Segment SI Centiles[%] Segment 7	centile	<5	5	56	15	0.267857	0.182879
Inter-operator	RV 24-Segment SI Centiles[%] Segment 7	centile	>95	95	56	5	0.089286	0.23913
Intra-operator	RV 24-Segment SI Centiles[%] Segment 7	centile	<5	5	30	7	0.233333	0.322581
Intra-operator	RV 24-Segment SI Centiles[%] Segment 7	centile	>95	95	30	3	0.1	-0.046512
Inter-operator	RV 24-Segment SI Centiles[%] Segment 8	centile	<5	5	56	14	0.25	0.155172
Inter-operator	RV 24-Segment SI Centiles[%] Segment 8	centile	>95	95	56	4	0.071429	0.464115
Intra-operator	RV 24-Segment SI Centiles[%] Segment 8	centile	<5	5	30	7	0.233333	0.255319
Intra-operator	RV 24-Segment SI Centiles[%] Segment 8	centile	>95	95	30	3	0.1	-0.046512
Inter-operator	RV 24-Segment SI Centiles[%] Segment 9	centile	<5	5	56	14	0.25	0.155172
Inter-operator	RV 24-Segment SI Centiles[%] Segment 9	centile	>95	95	56	3	0.053571	0.543478
Intra-operator	RV 24-Segment SI Centiles[%] Segment 9	centile	<5	5	30	9	0.3	-0.062992
Intra-operator	RV 24-Segment SI Centiles[%] Segment 9	centile	>95	95	30	3	0.1	-0.046512
Inter-operator	RV_ Basal-Apical FS.Rt BASE-AP FS %[%]	centile	<5	5	56	25	0.446429	0.118388
Inter-operator	RV_ Basal-Apical FS.Rt BASE-AP FS %[%]	centile	>95	95	56	8	0.142857	-0.056604
Intra-operator	RV_ Basal-Apical FS.Rt BASE-AP FS %[%]	centile	<5	5	30	9	0.3	0.27027
Intra-operator	RV_ Basal-Apical FS.Rt BASE-AP FS %[%]	centile	>95	95	30	4	0.133333	-0.071429
Inter-operator	RV_ Basal-Apical FS.Rt BASE-AP FS ZS[]	zscore	Z<-2	2	53	22	0.415094	0.12855
Inter-operator	RV_ Basal-Apical FS.Rt BASE-AP FS ZS[]	zscore	Z>2	2	53	7	0.132075	-0.033426
Inter-operator	RV_ Basal-Apical FS.Rt BASE-AP FS ZS[]	zscore	|Z|>1.645	1.645	53	30	0.566038	-0.134094
Inter-operator	RV_ Basal-Apical FS.Rt BASE-AP FS ZS[]	zscore	|Z|>2.0	2	53	27	0.509434	-0.031723
Intra-operator	RV_ Basal-Apical FS.Rt BASE-AP FS ZS[]	zscore	Z<-2	2	25	11	0.44	0.104235
Intra-operator	RV_ Basal-Apical FS.Rt BASE-AP FS ZS[]	zscore	Z>2	2	25	4	0.16	-0.086957
Intra-operator	RV_ Basal-Apical FS.Rt BASE-AP FS ZS[]	zscore	|Z|>1.645	1.645	25	6	0.24	0.364407
Intra-operator	RV_ Basal-Apical FS.Rt BASE-AP FS ZS[]	zscore	|Z|>2.0	2	25	9	0.36	0.181818
Inter-operator	RV_ Basal-Lat.Wall FS.Rt Basal-Lat FS % (AC)[%]	centile	<5	5	56	23	0.410714	0.20297

group	parameter	scale	threshold	thr_value	usable_n	flips	flip_rate	kappa
Inter-operator	RV_ Basal-Lat.Wall FS.Rt Basal-Lat FS % (AC)[%]	centile	>95	95	56	5	0.089286	-0.029412
Intra-operator	RV_ Basal-Lat.Wall FS.Rt Basal-Lat FS % (AC)[%]	centile	<5	5	30	9	0.3	0.366197
Intra-operator	RV_ Basal-Lat.Wall FS.Rt Basal-Lat FS % (AC)[%]	centile	>95	95	30	1	0.033333	0
Inter-operator	RV_ Basal-Lat.Wall FS.Rt Basal-Lat FS % (AUA)[%]	centile	<5	5	54	18	0.333333	0.354582
Inter-operator	RV_ Basal-Lat.Wall FS.Rt Basal-Lat FS % (AUA)[%]	centile	>95	95	54	5	0.092593	-0.030534
Intra-operator	RV_ Basal-Lat.Wall FS.Rt Basal-Lat FS % (AUA)[%]	centile	<5	5	30	9	0.3	0.366197
Intra-operator	RV_ Basal-Lat.Wall FS.Rt Basal-Lat FS % (AUA)[%]	centile	>95	95	30	1	0.033333	0
Inter-operator	RV_ Basal-Lat.Wall FS.Rt Basal-Lat FS % (BPD)[%]	centile	<5	5	55	20	0.363636	0.285714
Inter-operator	RV_ Basal-Lat.Wall FS.Rt Basal-Lat FS % (BPD)[%]	centile	>95	95	55	5	0.090909	-0.029963
Intra-operator	RV_ Basal-Lat.Wall FS.Rt Basal-Lat FS % (BPD)[%]	centile	<5	5	30	8	0.266667	0.425837
Intra-operator	RV_ Basal-Lat.Wall FS.Rt Basal-Lat FS % (BPD)[%]	centile	>95	95	30	1	0.033333	0
Inter-operator	RV_ Basal-Lat.Wall FS.Rt Basal-Lat FS % (EFW)[%]	centile	<5	5	55	18	0.327273	0.366197
Inter-operator	RV_ Basal-Lat.Wall FS.Rt Basal-Lat FS % (EFW)[%]	centile	>95	95	55	5	0.090909	-0.029963
Intra-operator	RV_ Basal-Lat.Wall FS.Rt Basal-Lat FS % (EFW)[%]	centile	<5	5	30	7	0.233333	0.461538
Intra-operator	RV_ Basal-Lat.Wall FS.Rt Basal-Lat FS % (EFW)[%]	centile	>95	95	30	1	0.033333	0
Inter-operator	RV_ Basal-Lat.Wall FS.Rt Basal-Lat FS % (FL)[%]	centile	<5	5	55	19	0.345455	0.319218
Inter-operator	RV_ Basal-Lat.Wall FS.Rt Basal-Lat FS % (FL)[%]	centile	>95	95	55	5	0.090909	-0.029963
Intra-operator	RV_ Basal-Lat.Wall FS.Rt Basal-Lat FS % (FL)[%]	centile	<5	5	29	8	0.275862	0.414141
Intra-operator	RV_ Basal-Lat.Wall FS.Rt Basal-Lat FS % (FL)[%]	centile	>95	95	29	1	0.034483	0
Inter-operator	RV_ Basal-Lat.Wall FS.Rt Basal-Lat FS % (GA)[%]	centile	<5	5	51	20	0.392157	0.231928
Inter-operator	RV_ Basal-Lat.Wall FS.Rt Basal-Lat FS % (GA)[%]	centile	>95	95	51	5	0.098039	-0.032389
Intra-operator	RV_ Basal-Lat.Wall FS.Rt Basal-Lat FS % (GA)[%]	centile	<5	5	28	8	0.285714	0.40107
Intra-operator	RV_ Basal-Lat.Wall FS.Rt Basal-Lat FS % (GA)[%]	centile	>95	95	28	1	0.035714	0
Inter-operator	RV_ Basal-Lat.Wall FS.Rt Basal-Lat FS % (HC)[%]	centile	<5	5	55	20	0.363636	0.285714
Inter-operator	RV_ Basal-Lat.Wall FS.Rt Basal-Lat FS % (HC)[%]	centile	>95	95	55	5	0.090909	-0.029963
Intra-operator	RV_ Basal-Lat.Wall FS.Rt Basal-Lat FS % (HC)[%]	centile	<5	5	30	9	0.3	0.366197
Intra-operator	RV_ Basal-Lat.Wall FS.Rt Basal-Lat FS % (HC)[%]	centile	>95	95	30	1	0.033333	0
Inter-operator	RV_ Basal-Sep.Wall FS.Rt Basal-Sep FS % (AC)[%]	centile	<5	5	56	21	0.375	0.194521
Inter-operator	RV_ Basal-Sep.Wall FS.Rt Basal-Sep FS % (AC)[%]	centile	>95	95	56	8	0.142857	-0.032258
Intra-operator	RV_ Basal-Sep.Wall FS.Rt Basal-Sep FS % (AC)[%]	centile	<5	5	30	21	0.7	-0.478873
Intra-operator	RV_ Basal-Sep.Wall FS.Rt Basal-Sep FS % (AC)[%]	centile	>95	95	30	4	0.133333	-0.071429
Inter-operator	RV_ Basal-Sep.Wall FS.Rt Basal-Sep FS % (AUA)[%]	centile	<5	5	54	21	0.388889	0.177068
Inter-operator	RV_ Basal-Sep.Wall FS.Rt Basal-Sep FS % (AUA)[%]	centile	>95	95	54	8	0.148148	-0.033493
Intra-operator	RV_ Basal-Sep.Wall FS.Rt Basal-Sep FS % (AUA)[%]	centile	<5	5	30	18	0.6	-0.343284
Intra-operator	RV_ Basal-Sep.Wall FS.Rt Basal-Sep FS % (AUA)[%]	centile	>95	95	30	4	0.133333	-0.071429
Inter-operator	RV_ Basal-Sep.Wall FS.Rt Basal-Sep FS % (BPD)[%]	centile	<5	5	55	22	0.4	0.157382
Inter-operator	RV_ Basal-Sep.Wall FS.Rt Basal-Sep FS % (BPD)[%]	centile	>95	95	55	8	0.145455	-0.032864
Intra-operator	RV_ Basal-Sep.Wall FS.Rt Basal-Sep FS % (BPD)[%]	centile	<5	5	30	16	0.533333	-0.126761
Intra-operator	RV_ Basal-Sep.Wall FS.Rt Basal-Sep FS % (BPD)[%]	centile	>95	95	30	4	0.133333	-0.071429
Inter-operator	RV_ Basal-Sep.Wall FS.Rt Basal-Sep FS % (EFW)[%]	centile	<5	5	55	23	0.418182	0.129387

group	parameter	scale	threshold	thr_value	usable_n	flips	flip_rate	kappa
Inter-operator	RV_ Basal-Sep.Wall FS.Rt Basal-Sep FS % (EFW)[%]	centile	>95	95	55	8	0.145455	-0.032864
Intra-operator	RV_ Basal-Sep.Wall FS.Rt Basal-Sep FS % (EFW)[%]	centile	<5	5	30	17	0.566667	-0.243902
Intra-operator	RV_ Basal-Sep.Wall FS.Rt Basal-Sep FS % (EFW)[%]	centile	>95	95	30	4	0.133333	-0.071429
Inter-operator	RV_ Basal-Sep.Wall FS.Rt Basal-Sep FS % (FL)[%]	centile	<5	5	55	22	0.4	0.157382
Inter-operator	RV_ Basal-Sep.Wall FS.Rt Basal-Sep FS % (FL)[%]	centile	>95	95	55	7	0.127273	-0.032172
Intra-operator	RV_ Basal-Sep.Wall FS.Rt Basal-Sep FS % (FL)[%]	centile	<5	5	29	18	0.62069	-0.366492
Intra-operator	RV_ Basal-Sep.Wall FS.Rt Basal-Sep FS % (FL)[%]	centile	>95	95	29	4	0.137931	-0.074074
Inter-operator	RV_ Basal-Sep.Wall FS.Rt Basal-Sep FS % (GA)[%]	centile	<5	5	51	20	0.392157	0.142857
Inter-operator	RV_ Basal-Sep.Wall FS.Rt Basal-Sep FS % (GA)[%]	centile	>95	95	51	8	0.156863	-0.035533
Intra-operator	RV_ Basal-Sep.Wall FS.Rt Basal-Sep FS % (GA)[%]	centile	<5	5	28	17	0.607143	-0.293478
Intra-operator	RV_ Basal-Sep.Wall FS.Rt Basal-Sep FS % (GA)[%]	centile	>95	95	28	4	0.142857	-0.076923
Inter-operator	RV_ Basal-Sep.Wall FS.Rt Basal-Sep FS % (HC)[%]	centile	<5	5	55	22	0.4	0.157382
Inter-operator	RV_ Basal-Sep.Wall FS.Rt Basal-Sep FS % (HC)[%]	centile	>95	95	55	8	0.145455	-0.032864
Intra-operator	RV_ Basal-Sep.Wall FS.Rt Basal-Sep FS % (HC)[%]	centile	<5	5	30	16	0.533333	-0.176471
Intra-operator	RV_ Basal-Sep.Wall FS.Rt Basal-Sep FS % (HC)[%]	centile	>95	95	30	4	0.133333	-0.071429
Inter-operator	RV_ ED Area.Rt ED Area % (AC)[%]	centile	<5	5	56	8	0.142857	0.255814
Inter-operator	RV_ ED Area.Rt ED Area % (AC)[%]	centile	>95	95	56	8	0.142857	0.700935
Intra-operator	RV_ ED Area.Rt ED Area % (AC)[%]	centile	<5	5	30	6	0.2	0.52381
Intra-operator	RV_ ED Area.Rt ED Area % (AC)[%]	centile	>95	95	30	5	0.166667	0.444444
Inter-operator	RV_ ED Area.Rt ED Area % (AUA)[%]	centile	<5	5	54	10	0.185185	0.274194
Inter-operator	RV_ ED Area.Rt ED Area % (AUA)[%]	centile	>95	95	54	10	0.185185	0.497207
Intra-operator	RV_ ED Area.Rt ED Area % (AUA)[%]	centile	<5	5	30	8	0.266667	0.425837
Intra-operator	RV_ ED Area.Rt ED Area % (AUA)[%]	centile	>95	95	30	3	0.1	0.366197
Inter-operator	RV_ ED Area.Rt ED Area % (BPD)[%]	centile	<5	5	55	12	0.218182	0.319588
Inter-operator	RV_ ED Area.Rt ED Area % (BPD)[%]	centile	>95	95	55	7	0.127273	0.554913
Intra-operator	RV_ ED Area.Rt ED Area % (BPD)[%]	centile	<5	5	30	9	0.3	0.372093
Intra-operator	RV_ ED Area.Rt ED Area % (BPD)[%]	centile	>95	95	30	1	0.033333	0.651163
Inter-operator	RV_ ED Area.Rt ED Area % (EFW)[%]	centile	<5	5	55	5	0.090909	0.395604
Inter-operator	RV_ ED Area.Rt ED Area % (EFW)[%]	centile	>95	95	55	8	0.145455	0.46988
Intra-operator	RV_ ED Area.Rt ED Area % (EFW)[%]	centile	<5	5	30	5	0.166667	0.444444
Intra-operator	RV_ ED Area.Rt ED Area % (EFW)[%]	centile	>95	95	30	0	0	1
Inter-operator	RV_ ED Area.Rt ED Area % (FL)[%]	centile	<5	5	55	10	0.181818	0.337349
Inter-operator	RV_ ED Area.Rt ED Area % (FL)[%]	centile	>95	95	55	7	0.127273	0.589989
Intra-operator	RV_ ED Area.Rt ED Area % (FL)[%]	centile	<5	5	29	6	0.206897	0.544503
Intra-operator	RV_ ED Area.Rt ED Area % (FL)[%]	centile	>95	95	29	2	0.068966	0.462963
Inter-operator	RV_ ED Area.Rt ED Area % (GA)[%]	centile	<5	5	51	10	0.196078	0.379562
Inter-operator	RV_ ED Area.Rt ED Area % (GA)[%]	centile	>95	95	51	5	0.098039	0.701754
Intra-operator	RV_ ED Area.Rt ED Area % (GA)[%]	centile	<5	5	28	7	0.25	0.473118
Intra-operator	RV_ ED Area.Rt ED Area % (GA)[%]	centile	>95	95	28	1	0.035714	0.65
Inter-operator	RV_ ED Area.Rt ED Area % (HC)[%]	centile	<5	5	55	11	0.2	0.460303

group	parameter	scale	threshold	thr_value	usable_n	flips	flip_rate	kappa
Inter-operator	RV_ ED Area.Rt ED Area % (HC)[%]	centile	>95	95	55	10	0.181818	0.542429
Intra-operator	RV_ ED Area.Rt ED Area % (HC)[%]	centile	<5	5	30	11	0.366667	0.303797
Intra-operator	RV_ ED Area.Rt ED Area % (HC)[%]	centile	>95	95	30	1	0.033333	0.651163
Inter-operator	RV_ ED Length.Rt ED Length % (AC)[%]	centile	<5	5	56	13	0.232143	0.443425
Inter-operator	RV_ ED Length.Rt ED Length % (AC)[%]	centile	>95	95	56	2	0.035714	-0.018182
Intra-operator	RV_ ED Length.Rt ED Length % (AC)[%]	centile	<5	5	30	10	0.333333	0.342105
Intra-operator	RV_ ED Length.Rt ED Length % (AC)[%]	centile	>95	95	30	0	0	
Inter-operator	RV_ ED Length.Rt ED Length % (AUA)[%]	centile	<5	5	54	13	0.240741	0.452418
Inter-operator	RV_ ED Length.Rt ED Length % (AUA)[%]	centile	>95	95	54	1	0.018519	0
Intra-operator	RV_ ED Length.Rt ED Length % (AUA)[%]	centile	<5	5	30	8	0.266667	0.466667
Intra-operator	RV_ ED Length.Rt ED Length % (AUA)[%]	centile	>95	95	30	0	0	
Inter-operator	RV_ ED Length.Rt ED Length % (BPD)[%]	centile	<5	5	55	12	0.218182	0.518248
Inter-operator	RV_ ED Length.Rt ED Length % (BPD)[%]	centile	>95	95	55	1	0.018182	0
Intra-operator	RV_ ED Length.Rt ED Length % (BPD)[%]	centile	<5	5	30	8	0.266667	0.447005
Intra-operator	RV_ ED Length.Rt ED Length % (BPD)[%]	centile	>95	95	30	1	0.033333	0
Inter-operator	RV_ ED Length.Rt ED Length % (EFW)[%]	centile	<5	5	55	13	0.236364	0.457099
Inter-operator	RV_ ED Length.Rt ED Length % (EFW)[%]	centile	>95	95	55	1	0.018182	0
Intra-operator	RV_ ED Length.Rt ED Length % (EFW)[%]	centile	<5	5	30	9	0.3	0.405286
Intra-operator	RV_ ED Length.Rt ED Length % (EFW)[%]	centile	>95	95	30	0	0	
Inter-operator	RV_ ED Length.Rt ED Length % (FL)[%]	centile	<5	5	55	12	0.218182	0.489954
Inter-operator	RV_ ED Length.Rt ED Length % (FL)[%]	centile	>95	95	55	1	0.018182	0
Intra-operator	RV_ ED Length.Rt ED Length % (FL)[%]	centile	<5	5	29	9	0.310345	0.382979
Intra-operator	RV_ ED Length.Rt ED Length % (FL)[%]	centile	>95	95	29	0	0	
Inter-operator	RV_ ED Length.Rt ED Length % (GA)[%]	centile	<5	5	51	11	0.215686	0.490463
Inter-operator	RV_ ED Length.Rt ED Length % (GA)[%]	centile	>95	95	51	1	0.019608	0
Intra-operator	RV_ ED Length.Rt ED Length % (GA)[%]	centile	<5	5	28	9	0.321429	0.376238
Intra-operator	RV_ ED Length.Rt ED Length % (GA)[%]	centile	>95	95	28	0	0	
Inter-operator	RV_ ED Length.Rt ED Length % (HC)[%]	centile	<5	5	55	15	0.272727	0.406048
Inter-operator	RV_ ED Length.Rt ED Length % (HC)[%]	centile	>95	95	55	1	0.018182	0
Intra-operator	RV_ ED Length.Rt ED Length % (HC)[%]	centile	<5	5	30	7	0.233333	0.524887
Intra-operator	RV_ ED Length.Rt ED Length % (HC)[%]	centile	>95	95	30	0	0	
Inter-operator	RV_ Frac. Area change.Rt FRAC % (AC)[%]	centile	<5	5	56	13	0.232143	0.338182
Inter-operator	RV_ Frac. Area change.Rt FRAC % (AC)[%]	centile	>95	95	56	2	0.035714	0
Intra-operator	RV_ Frac. Area change.Rt FRAC % (AC)[%]	centile	<5	5	30	7	0.233333	-0.129032
Intra-operator	RV_ Frac. Area change.Rt FRAC % (AC)[%]	centile	>95	95	30	1	0.033333	0
Inter-operator	RV_ Frac. Area change.Rt FRAC % (AUA)[%]	centile	<5	5	54	12	0.222222	0.368421
Inter-operator	RV_ Frac. Area change.Rt FRAC % (AUA)[%]	centile	>95	95	54	2	0.037037	0
Intra-operator	RV_ Frac. Area change.Rt FRAC % (AUA)[%]	centile	<5	5	30	7	0.233333	-0.129032
Intra-operator	RV_ Frac. Area change.Rt FRAC % (AUA)[%]	centile	>95	95	30	1	0.033333	0
Inter-operator	RV_ Frac. Area change.Rt FRAC % (BPD)[%]	centile	<5	5	55	13	0.236364	0.368932

group	parameter	scale	threshold	thr_value	usable_n	flips	flip_rate	kappa
Inter-operator	RV_ Frac. Area change.Rt FRAC % (BPD)[%]	centile	>95	95	55	2	0.036364	0
Intra-operator	RV_ Frac. Area change.Rt FRAC % (BPD)[%]	centile	<5	5	30	7	0.233333	-0.129032
Intra-operator	RV_ Frac. Area change.Rt FRAC % (BPD)[%]	centile	>95	95	30	1	0.033333	0
Inter-operator	RV_ Frac. Area change.Rt FRAC % (EFW)[%]	centile	<5	5	55	13	0.236364	0.334884
Inter-operator	RV_ Frac. Area change.Rt FRAC % (EFW)[%]	centile	>95	95	55	2	0.036364	0
Intra-operator	RV_ Frac. Area change.Rt FRAC % (EFW)[%]	centile	<5	5	30	7	0.233333	-0.129032
Intra-operator	RV_ Frac. Area change.Rt FRAC % (EFW)[%]	centile	>95	95	30	1	0.033333	0
Inter-operator	RV_ Frac. Area change.Rt FRAC % (FL)[%]	centile	<5	5	55	13	0.236364	0.334884
Inter-operator	RV_ Frac. Area change.Rt FRAC % (FL)[%]	centile	>95	95	55	2	0.036364	0
Intra-operator	RV_ Frac. Area change.Rt FRAC % (FL)[%]	centile	<5	5	29	7	0.241379	-0.134078
Intra-operator	RV_ Frac. Area change.Rt FRAC % (FL)[%]	centile	>95	95	29	1	0.034483	0
Inter-operator	RV_ Frac. Area change.Rt FRAC % (GA)[%]	centile	<5	5	51	12	0.235294	0.381818
Inter-operator	RV_ Frac. Area change.Rt FRAC % (GA)[%]	centile	>95	95	51	2	0.039216	0
Intra-operator	RV_ Frac. Area change.Rt FRAC % (GA)[%]	centile	<5	5	28	7	0.25	-0.139535
Intra-operator	RV_ Frac. Area change.Rt FRAC % (GA)[%]	centile	>95	95	28	1	0.035714	0
Inter-operator	RV_ Frac. Area change.Rt FRAC % (HC)[%]	centile	<5	5	55	13	0.236364	0.334884
Inter-operator	RV_ Frac. Area change.Rt FRAC % (HC)[%]	centile	>95	95	55	2	0.036364	0
Intra-operator	RV_ Frac. Area change.Rt FRAC % (HC)[%]	centile	<5	5	30	7	0.233333	-0.129032
Intra-operator	RV_ Frac. Area change.Rt FRAC % (HC)[%]	centile	>95	95	30	1	0.033333	0
Inter-operator	RV_ FreeWall Strain.Rt FWS %[%]	centile	<5	5	56	5	0.089286	0.247312
Inter-operator	RV_ FreeWall Strain.Rt FWS %[%]	centile	>95	95	56	16	0.285714	0.419689
Intra-operator	RV_ FreeWall Strain.Rt FWS %[%]	centile	<5	5	30	3	0.1	-0.046512
Intra-operator	RV_ FreeWall Strain.Rt FWS %[%]	centile	>95	95	30	8	0.266667	0.368421
Inter-operator	RV_ FreeWall Strain.Rt FWS ZS[]	zscore	Z<-2	2	53	4	0.075472	-0.039216
Inter-operator	RV_ FreeWall Strain.Rt FWS ZS[]	zscore	Z>2	2	53	20	0.377358	0.242857
Inter-operator	RV_ FreeWall Strain.Rt FWS ZS[]	zscore	|Z|>1.645	1.645	53	19	0.358491	0.244561
Inter-operator	RV_ FreeWall Strain.Rt FWS ZS[]	zscore	|Z|>2.0	2	53	22	0.415094	0.174221
Intra-operator	RV_ FreeWall Strain.Rt FWS ZS[]	zscore	Z<-2	2	25	3	0.12	-0.056338
Intra-operator	RV_ FreeWall Strain.Rt FWS ZS[]	zscore	Z>2	2	25	6	0.24	0.482759
Intra-operator	RV_ FreeWall Strain.Rt FWS ZS[]	zscore	|Z|>1.645	1.645	25	6	0.24	0.409449
Intra-operator	RV_ FreeWall Strain.Rt FWS ZS[]	zscore	|Z|>2.0	2	25	5	0.2	0.524715
Inter-operator	RV_ Global Strain.Rt GLS %[%]	centile	<5	5	56	7	0.125	-0.053763
Inter-operator	RV_ Global Strain.Rt GLS %[%]	centile	>95	95	56	19	0.339286	0.3
Intra-operator	RV_ Global Strain.Rt GLS %[%]	centile	<5	5	30	3	0.1	-0.046512
Intra-operator	RV_ Global Strain.Rt GLS %[%]	centile	>95	95	30	6	0.2	0.444444
Inter-operator	RV_ Global Strain.Rt GLS ZS[]	zscore	Z<-2	2	53	7	0.132075	-0.05698
Inter-operator	RV_ Global Strain.Rt GLS ZS[]	zscore	Z>2	2	53	19	0.358491	0.285309
Inter-operator	RV_ Global Strain.Rt GLS ZS[]	zscore	|Z|>1.645	1.645	53	22	0.415094	0.098918
Inter-operator	RV_ Global Strain.Rt GLS ZS[]	zscore	|Z|>2.0	2	53	22	0.415094	0.13373
Intra-operator	RV_ Global Strain.Rt GLS ZS[]	zscore	Z<-2	2	25	2	0.08	-0.041667

group	parameter	scale	threshold	thr_value	usable_n	flips	flip_rate	kappa
Intra-operator	RV_ Global Strain.Rt GLS ZS[]	zscore	Z>2	2	25	5	0.2	0.545455
Intra-operator	RV_ Global Strain.Rt GLS ZS[]	zscore	|Z|>1.645	1.645	25	6	0.24	0.278846
Intra-operator	RV_ Global Strain.Rt GLS ZS[]	zscore	|Z|>2.0	2	25	5	0.2	0.505929
Inter-operator	RV_ Sept.Wall Strain.Rt SWS %[%]	centile	<5	5	56	5	0.089286	0.23913
Inter-operator	RV_ Sept.Wall Strain.Rt SWS %[%]	centile	>95	95	56	12	0.214286	0.453659
Intra-operator	RV_ Sept.Wall Strain.Rt SWS %[%]	centile	<5	5	30	2	0.066667	0
Intra-operator	RV_ Sept.Wall Strain.Rt SWS %[%]	centile	>95	95	30	11	0.366667	0.26009
Inter-operator	RV_ Sept.Wall Strain.Rt SWS ZS[]	zscore	Z<-2	2	56	5	0.089286	-0.044776
Inter-operator	RV_ Sept.Wall Strain.Rt SWS ZS[]	zscore	Z>2	2	56	13	0.232143	0.368056
Inter-operator	RV_ Sept.Wall Strain.Rt SWS ZS[]	zscore	|Z|>1.645	1.645	56	17	0.303571	0.314121
Inter-operator	RV_ Sept.Wall Strain.Rt SWS ZS[]	zscore	|Z|>2.0	2	56	18	0.321429	0.217391
Intra-operator	RV_ Sept.Wall Strain.Rt SWS ZS[]	zscore	Z<-2	2	29	2	0.068966	0
Intra-operator	RV_ Sept.Wall Strain.Rt SWS ZS[]	zscore	Z>2	2	29	10	0.344828	0.289216
Intra-operator	RV_ Sept.Wall Strain.Rt SWS ZS[]	zscore	|Z|>1.645	1.645	29	10	0.344828	0.32243
Intra-operator	RV_ Sept.Wall Strain.Rt SWS ZS[]	zscore	|Z|>2.0	2	29	10	0.344828	0.30622
Inter-operator	RV_ TAPSE lateral.TAPSE lat. % (AC)[%]	centile	<5	5	56	17	0.303571	0.395939
Inter-operator	RV_ TAPSE lateral.TAPSE lat. % (AC)[%]	centile	>95	95	56	4	0.071429	-0.027523
Intra-operator	RV_ TAPSE lateral.TAPSE lat. % (AC)[%]	centile	<5	5	30	8	0.266667	0.322034
Intra-operator	RV_ TAPSE lateral.TAPSE lat. % (AC)[%]	centile	>95	95	30	1	0.033333	0
Inter-operator	RV_ TAPSE lateral.TAPSE lat. % (AUA)[%]	centile	<5	5	54	14	0.259259	0.481481
Inter-operator	RV_ TAPSE lateral.TAPSE lat. % (AUA)[%]	centile	>95	95	54	4	0.074074	-0.028571
Intra-operator	RV_ TAPSE lateral.TAPSE lat. % (AUA)[%]	centile	<5	5	30	8	0.266667	0.322034
Intra-operator	RV_ TAPSE lateral.TAPSE lat. % (AUA)[%]	centile	>95	95	30	1	0.033333	0
Inter-operator	RV_ TAPSE lateral.TAPSE lat. % (BPD)[%]	centile	<5	5	55	15	0.272727	0.449633
Inter-operator	RV_ TAPSE lateral.TAPSE lat. % (BPD)[%]	centile	>95	95	55	3	0.054545	-0.024845
Intra-operator	RV_ TAPSE lateral.TAPSE lat. % (BPD)[%]	centile	<5	5	30	8	0.266667	0.322034
Intra-operator	RV_ TAPSE lateral.TAPSE lat. % (BPD)[%]	centile	>95	95	30	1	0.033333	0
Inter-operator	RV_ TAPSE lateral.TAPSE lat. % (EFW)[%]	centile	<5	5	55	18	0.327273	0.346966
Inter-operator	RV_ TAPSE lateral.TAPSE lat. % (EFW)[%]	centile	>95	95	55	4	0.072727	-0.028037
Intra-operator	RV_ TAPSE lateral.TAPSE lat. % (EFW)[%]	centile	<5	5	30	8	0.266667	0.322034
Intra-operator	RV_ TAPSE lateral.TAPSE lat. % (EFW)[%]	centile	>95	95	30	1	0.033333	0
Inter-operator	RV_ TAPSE lateral.TAPSE lat. % (FL)[%]	centile	<5	5	56	16	0.285714	0.42268
Inter-operator	RV_ TAPSE lateral.TAPSE lat. % (FL)[%]	centile	>95	95	56	3	0.053571	-0.02439
Intra-operator	RV_ TAPSE lateral.TAPSE lat. % (FL)[%]	centile	<5	5	29	8	0.275862	0.313609
Intra-operator	RV_ TAPSE lateral.TAPSE lat. % (FL)[%]	centile	>95	95	29	1	0.034483	0
Inter-operator	RV_ TAPSE lateral.TAPSE lat. % (GA)[%]	centile	<5	5	51	16	0.313725	0.367442
Inter-operator	RV_ TAPSE lateral.TAPSE lat. % (GA)[%]	centile	>95	95	51	4	0.078431	-0.030303
Intra-operator	RV_ TAPSE lateral.TAPSE lat. % (GA)[%]	centile	<5	5	28	7	0.25	0.371795
Intra-operator	RV_ TAPSE lateral.TAPSE lat. % (GA)[%]	centile	>95	95	28	1	0.035714	0
Inter-operator	RV_ TAPSE lateral.TAPSE lat. % (HC)[%]	centile	<5	5	55	14	0.254545	0.483221

group	parameter	scale	threshold	thr_value	usable_n	flips	flip_rate	kappa
Inter-operator	RV_ TAPSE lateral.TAPSE lat. % (HC)[%]	centile	>95	95	55	3	0.054545	-0.024845
Intra-operator	RV_ TAPSE lateral.TAPSE lat. % (HC)[%]	centile	<5	5	30	8	0.266667	0.322034
Intra-operator	RV_ TAPSE lateral.TAPSE lat. % (HC)[%]	centile	>95	95	30	1	0.033333	0
Inter-operator	RV_ TAPSE septal.TAPSE sept. % (AC)[%]	centile	<5	5	55	24	0.436364	0.124668
Inter-operator	RV_ TAPSE septal.TAPSE sept. % (AC)[%]	centile	>95	95	55	6	0.109091	-0.03125
Intra-operator	RV_ TAPSE septal.TAPSE sept. % (AC)[%]	centile	<5	5	30	7	0.233333	0.086957
Intra-operator	RV_ TAPSE septal.TAPSE sept. % (AC)[%]	centile	>95	95	30	2	0.066667	-0.034483
Inter-operator	RV_ TAPSE septal.TAPSE sept. % (AUA)[%]	centile	<5	5	53	20	0.377358	0.243937
Inter-operator	RV_ TAPSE septal.TAPSE sept. % (AUA)[%]	centile	>95	95	53	6	0.113208	-0.032468
Intra-operator	RV_ TAPSE septal.TAPSE sept. % (AUA)[%]	centile	<5	5	30	6	0.2	0.285714
Intra-operator	RV_ TAPSE septal.TAPSE sept. % (AUA)[%]	centile	>95	95	30	2	0.066667	-0.034483
Inter-operator	RV_ TAPSE septal.TAPSE sept. % (BPD)[%]	centile	<5	5	54	20	0.37037	0.243697
Inter-operator	RV_ TAPSE septal.TAPSE sept. % (BPD)[%]	centile	>95	95	54	6	0.111111	-0.031847
Intra-operator	RV_ TAPSE septal.TAPSE sept. % (BPD)[%]	centile	<5	5	30	7	0.233333	0.086957
Intra-operator	RV_ TAPSE septal.TAPSE sept. % (BPD)[%]	centile	>95	95	30	2	0.066667	-0.034483
Inter-operator	RV_ TAPSE septal.TAPSE sept. % (EFW)[%]	centile	<5	5	54	22	0.407407	0.185185
Inter-operator	RV_ TAPSE septal.TAPSE sept. % (EFW)[%]	centile	>95	95	54	6	0.111111	-0.031847
Intra-operator	RV_ TAPSE septal.TAPSE sept. % (EFW)[%]	centile	<5	5	30	8	0.266667	0.047619
Intra-operator	RV_ TAPSE septal.TAPSE sept. % (EFW)[%]	centile	>95	95	30	2	0.066667	-0.034483
Inter-operator	RV_ TAPSE septal.TAPSE sept. % (FL)[%]	centile	<5	5	55	22	0.4	0.186828
Inter-operator	RV_ TAPSE septal.TAPSE sept. % (FL)[%]	centile	>95	95	55	6	0.109091	-0.03125
Intra-operator	RV_ TAPSE septal.TAPSE sept. % (FL)[%]	centile	<5	5	29	6	0.206897	0.280992
Intra-operator	RV_ TAPSE septal.TAPSE sept. % (FL)[%]	centile	>95	95	29	2	0.068966	-0.035714
Inter-operator	RV_ TAPSE septal.TAPSE sept. % (GA)[%]	centile	<5	5	50	20	0.4	0.189627
Inter-operator	RV_ TAPSE septal.TAPSE sept. % (GA)[%]	centile	>95	95	50	5	0.1	-0.033058
Intra-operator	RV_ TAPSE septal.TAPSE sept. % (GA)[%]	centile	<5	5	28	7	0.25	0.075472
Intra-operator	RV_ TAPSE septal.TAPSE sept. % (GA)[%]	centile	>95	95	28	2	0.071429	-0.037037
Inter-operator	RV_ TAPSE septal.TAPSE sept. % (HC)[%]	centile	<5	5	54	21	0.388889	0.209205
Inter-operator	RV_ TAPSE septal.TAPSE sept. % (HC)[%]	centile	>95	95	54	6	0.111111	-0.031847
Intra-operator	RV_ TAPSE septal.TAPSE sept. % (HC)[%]	centile	<5	5	30	7	0.233333	0.086957
Intra-operator	RV_ TAPSE septal.TAPSE sept. % (HC)[%]	centile	>95	95	30	2	0.066667	-0.034483

Supplementary File S3

WorstCaseSummary: Worst-case flip rate per parameter (by group and scale)

group	parameter	scale	usable_n	max_flip_rate	min_kappa
Intra-operator	RV_ Basal-Sep.Wall FS.Rt Basal-Sep FS % (AC)[%]	centile	30	0.7	-0.478873
Intra-operator	RV_ Basal-Sep.Wall FS.Rt Basal-Sep FS % (FL)[%]	centile	29	0.62069	-0.366492
Intra-operator	RV_ Basal-Sep.Wall FS.Rt Basal-Sep FS % (GA)[%]	centile	28	0.607143	-0.293478
Intra-operator	RV 24-Segment FS Centiles[%] Segment 4	centile	30	0.6	-0.153846
Intra-operator	RV_ Basal-Sep.Wall FS.Rt Basal-Sep FS % (AUA)[%]	centile	30	0.6	-0.343284
Intra-operator	RV 24-Segment FS Centiles[%] Segment 5	centile	30	0.566667	-0.075949
Intra-operator	RV_ Basal-Sep.Wall FS.Rt Basal-Sep FS % (EFW)[%]	centile	30	0.566667	-0.243902
Intra-operator	RV_ Basal-Sep.Wall FS.Rt Basal-Sep FS % (BPD)[%]	centile	30	0.533333	-0.126761
Intra-operator	RV_ Basal-Sep.Wall FS.Rt Basal-Sep FS % (HC)[%]	centile	30	0.533333	-0.176471
Intra-operator	RV 24-Segment FS Centiles[%] Segment 6	centile	30	0.5	0
Inter-operator	LV_ CO/KG.Lt CO/KG % (AC)[%]	centile	27	0.481481	0.04878
Inter-operator	LV_ CO/KG.Lt CO/KG % (AUA)[%]	centile	53	0.471698	0.052895
Inter-operator	LV_ CO/KG.Lt CO/KG % (BPD)[%]	centile	51	0.470588	0.0347
Inter-operator	LV_ CO/KG.Lt CO/KG % (FL)[%]	centile	32	0.46875	0.069767
Intra-operator	LV 24-Segment FS Centiles[%] Segment 1	centile	30	0.466667	-0.088083
Intra-operator	LV 24-Segment FS Centiles[%] Segment 10	centile	30	0.466667	0.032258
Intra-operator	RV 24-Segment FS Centiles[%] Segment 18	centile	30	0.466667	0.070796
Intra-operator	RV 24-Segment FS Centiles[%] Segment 8	centile	30	0.466667	0
Inter-operator	LV 24-Segment FS Centiles[%] Segment 10	centile	56	0.464286	0.056995
Inter-operator	LV 24-Segment FS Centiles[%] Segment 13	centile	56	0.464286	0.09
Inter-operator	LV 24-Segment FS Centiles[%] Segment 14	centile	56	0.464286	0.085427
Inter-operator	LV 24-Segment FS Centiles[%] Segment 16	centile	56	0.464286	0.080808
Inter-operator	LV 24-Segment FS Centiles[%] Segment 17	centile	56	0.464286	0
Inter-operator	LV 24-Segment FS Centiles[%] Segment 18	centile	56	0.464286	0
Inter-operator	LV 24-Segment FS Centiles[%] Segment 9	centile	56	0.464286	0.031915
Inter-operator	LV 24-Segment FS Centiles[%] Segment 11	centile	56	0.446429	0.118388
Inter-operator	RV_ Basal-Apical FS.Rt BASE-AP FS %[%]	centile	56	0.446429	-0.056604
Inter-operator	RV_ TAPSE septal.TAPSE sept. % (AC)[%]	centile	55	0.436364	-0.03125
Intra-operator	LV 24-Segment FS Centiles[%] Segment 2	centile	30	0.433333	0
Intra-operator	LV 24-Segment FS Centiles[%] Segment 3	centile	30	0.433333	0
Intra-operator	LV 24-Segment FS Centiles[%] Segment 9	centile	30	0.433333	0.109589
Intra-operator	RV 24-Segment FS Centiles[%] Segment 14	centile	30	0.433333	0.084507
Intra-operator	RV 24-Segment FS Centiles[%] Segment 15	centile	30	0.433333	0.109589
Intra-operator	RV 24-Segment FS Centiles[%] Segment 17	centile	30	0.433333	0.140969
Intra-operator	RV 24-Segment FS Centiles[%] Segment 2	centile	30	0.433333	0
Intra-operator	RV 24-Segment FS Centiles[%] Segment 3	centile	30	0.433333	0
Intra-operator	RV 24-Segment FS Centiles[%] Segment 7	centile	30	0.433333	0

group	parameter	scale	usable_n	max_flip_rate	min_kappa
Intra-operator	RV 24-Segment FS Centiles[%] Segment 9	centile	30	0.433333	0.16309
Inter-operator	LV 24-Segment FS Centiles[%] Segment 12	centile	56	0.428571	0.158949
Inter-operator	LV 24-Segment FS Centiles[%] Segment 19	centile	56	0.428571	0
Intra-operator	LV_ MAPSE septal.MAPSE sept. % (GA)[%]	centile	28	0.428571	0.120419
Inter-operator	LV_ EF.Lt EF % (FL)[%]	centile	33	0.424242	0
Inter-operator	RV_ Basal-Sep.Wall FS.Rt Basal-Sep FS % (EFW)[%]	centile	55	0.418182	-0.032864
Intra-operator	LV_ MAPSE septal.MAPSE sept. % (FL)[%]	centile	29	0.413793	0.179245
Inter-operator	LV 24-Segment FS Centiles[%] Segment 15	centile	56	0.410714	0.188917
Inter-operator	LV 24-Segment FS Centiles[%] Segment 7	centile	56	0.410714	0.052941
Inter-operator	LV 24-Segment FS Centiles[%] Segment 8	centile	56	0.410714	0.110497
Inter-operator	LV_ Frac. Area change.Lt FRAC % (AC)[%]	centile	56	0.410714	-0.018182
Inter-operator	RV 24-Segment FS Centiles[%] Segment 8	centile	56	0.410714	0.176471
Inter-operator	RV 24-Segment FS Centiles[%] Segment 9	centile	56	0.410714	0.172237
Inter-operator	RV_ Basal-Lat.Wall FS.Rt Basal-Lat FS % (AC)[%]	centile	56	0.410714	-0.029412
Inter-operator	RV_ TAPSE septal.TAPSE sept. % (EFW)[%]	centile	54	0.407407	-0.031847
Inter-operator	LV_ CO/KG.Lt CO/KG % (GA)[%]	centile	50	0.4	0.193548
Inter-operator	RV_ Basal-Sep.Wall FS.Rt Basal-Sep FS % (BPD)[%]	centile	55	0.4	-0.032864
Inter-operator	RV_ Basal-Sep.Wall FS.Rt Basal-Sep FS % (FL)[%]	centile	55	0.4	-0.032172
Inter-operator	RV_ Basal-Sep.Wall FS.Rt Basal-Sep FS % (HC)[%]	centile	55	0.4	-0.032864
Inter-operator	RV_ TAPSE septal.TAPSE sept. % (FL)[%]	centile	55	0.4	-0.03125
Inter-operator	RV_ TAPSE septal.TAPSE sept. % (GA)[%]	centile	50	0.4	-0.033058
Intra-operator	LV 24-Segment FS Centiles[%] Segment 11	centile	30	0.4	0.104478
Intra-operator	LV 24-Segment FS Centiles[%] Segment 12	centile	30	0.4	0.104478
Intra-operator	LV 24-Segment SI Centiles[%] Segment 13	centile	30	0.4	-0.090909
Intra-operator	LV_ ED Length.Lt ED Length % (AUA)[%]	centile	30	0.4	0.210526
Intra-operator	LV_ ED Length.Lt ED Length % (EFW)[%]	centile	30	0.4	0.227468
Intra-operator	RV 24-Segment FS Centiles[%] Segment 1	centile	30	0.4	0
Intra-operator	RV 24-Segment FS Centiles[%] Segment 10	centile	30	0.4	0.224138
Intra-operator	RV 24-Segment FS Centiles[%] Segment 13	centile	30	0.4	0.104478
Inter-operator	LV_ CO.Lt CO % (FL)[%]	centile	33	0.393939	0
Inter-operator	LV 24-Segment FS Centiles[%] Segment 1	centile	56	0.392857	-0.044776
Inter-operator	RV 24-Segment FS Centiles[%] Segment 12	centile	56	0.392857	0.19896
Inter-operator	RV 24-Segment FS Centiles[%] Segment 7	centile	56	0.392857	0.217281
Inter-operator	LV_ Frac. Area change.Lt FRAC % (GA)[%]	centile	51	0.392157	-0.026846
Inter-operator	RV_ Basal-Lat.Wall FS.Rt Basal-Lat FS % (GA)[%]	centile	51	0.392157	-0.032389
Inter-operator	RV_ Basal-Sep.Wall FS.Rt Basal-Sep FS % (GA)[%]	centile	51	0.392157	-0.035533
Inter-operator	RV_ Basal-Sep.Wall FS.Rt Basal-Sep FS % (AUA)[%]	centile	54	0.388889	-0.033493
Inter-operator	RV_ TAPSE septal.TAPSE sept. % (HC)[%]	centile	54	0.388889	-0.031847
Inter-operator	RV_ TAPSE septal.TAPSE sept. % (AUA)[%]	centile	53	0.377358	-0.032468
Inter-operator	LV 24-Segment FS Centiles[%] Segment 20	centile	56	0.375	0

group	parameter	scale	usable_n	max_flip_rate	min_kappa
Inter-operator	LV 24-Segment FS Centiles[%] Segment 21	centile	56	0.375	0.261307
Inter-operator	LV_ Basal-Lat.Wall FS.Lt Basal-Lat FS % (AC)[%]	centile	56	0.375	-0.130435
Inter-operator	LV_ MAPSE lateral.MAPSE lat. % (AC)[%]	centile	56	0.375	-0.05
Inter-operator	LV_ MAPSE lateral.MAPSE lat. % (FL)[%]	centile	56	0.375	-0.029412
Inter-operator	LV_ SV/KG.Lt SV/KG % (FL)[%]	centile	32	0.375	0.23506
Inter-operator	RV 24-Segment FS Centiles[%] Segment 13	centile	56	0.375	0.244216
Inter-operator	RV_ Basal-Sep.Wall FS.Rt Basal-Sep FS % (AC)[%]	centile	56	0.375	-0.032258
Inter-operator	LV_ SV/KG.Lt SV/KG % (BPD)[%]	centile	51	0.372549	0.234234
Inter-operator	LV_ SV/KG.Lt SV/KG % (AC)[%]	centile	27	0.37037	0.274194
Inter-operator	RV_ TAPSE septal.TAPSE sept. % (BPD)[%]	centile	54	0.37037	-0.031847
Inter-operator	LV_ CO/KG.Lt CO/KG % (EFW)[%]	centile	19	0.368421	0.265193
Inter-operator	LV_ EF.Lt EF % (EFW)[%]	centile	19	0.368421	0
Inter-operator	LV_ SV.Lt SV % (EFW)[%]	centile	19	0.368421	0.231214
Intra-operator	LV 24-Segment FS Centiles[%] Segment 7	centile	30	0.366667	0.26009
Intra-operator	LV 24-Segment FS Centiles[%] Segment 8	centile	30	0.366667	0.273128
Intra-operator	LV 24-Segment SI Centiles[%] Segment 12	centile	30	0.366667	0
Intra-operator	LV 24-Segment SI Centiles[%] Segment 14	centile	30	0.366667	-0.064516
Intra-operator	LV 24-Segment SI Centiles[%] Segment 15	centile	30	0.366667	-0.064516
Intra-operator	LV 24-Segment SI Centiles[%] Segment 3	centile	30	0.366667	0
Intra-operator	LV 24-Segment SI Centiles[%] Segment 4	centile	30	0.366667	0
Intra-operator	LV_ ED Length.Lt ED Length % (AC)[%]	centile	30	0.366667	0.285714
Intra-operator	LV_ MAPSE septal.MAPSE sept. % (BPD)[%]	centile	30	0.366667	0.193548
Intra-operator	LV_ MAPSE septal.MAPSE sept. % (EFW)[%]	centile	30	0.366667	0.253394
Intra-operator	LV_ MAPSE septal.MAPSE sept. % (HC)[%]	centile	30	0.366667	0.193548
Intra-operator	RV 24-Segment FS Centiles[%] Segment 11	centile	30	0.366667	0.179104
Intra-operator	RV 24-Segment FS Centiles[%] Segment 16	centile	30	0.366667	0.26009
Intra-operator	RV_ ED Area.Rt ED Area % (HC)[%]	centile	30	0.366667	0.303797
Intra-operator	RV_ Sept.Wall Strain.Rt SWS %[%]	centile	30	0.366667	0
Inter-operator	LV_ Basal-Lat.Wall FS.Lt Basal-Lat FS % (FL)[%]	centile	55	0.363636	-0.125984
Inter-operator	LV_ Basal-Lat.Wall FS.Lt Basal-Lat FS % (HC)[%]	centile	55	0.363636	-0.142433
Inter-operator	RV_ Basal-Lat.Wall FS.Rt Basal-Lat FS % (BPD)[%]	centile	55	0.363636	-0.029963
Inter-operator	RV_ Basal-Lat.Wall FS.Rt Basal-Lat FS % (HC)[%]	centile	55	0.363636	-0.029963
Inter-operator	LV_ SV/KG.Lt SV/KG % (AUA)[%]	centile	53	0.358491	0.291344
Inter-operator	LV 24-Segment FS Centiles[%] Segment 4	centile	56	0.357143	-0.02439
Inter-operator	LV 24-Segment FS Centiles[%] Segment 6	centile	56	0.357143	-0.037037
Inter-operator	LV_ CO.Lt CO % (HC)[%]	centile	14	0.357143	0.285714
Inter-operator	LV_ CO/KG.Lt CO/KG % (HC)[%]	centile	14	0.357143	0.285714
Inter-operator	LV_ EF.Lt EF % (AC)[%]	centile	28	0.357143	0
Inter-operator	LV_ Global Strain.Lt GLS %[%]	centile	56	0.357143	0.153846
Inter-operator	LV_ SV.Lt SV % (AC)[%]	centile	28	0.357143	0.28934

group	parameter	scale	usable_n	max_flip_rate	min_kappa
Inter-operator	LV_ SV/KG.Lt SV/KG % (HC)[%]	centile	14	0.357143	0.285714
Inter-operator	LV_ Sept.Wall Strain.Lt SWS %[%]	centile	56	0.357143	0.231707
Inter-operator	RV 24-Segment FS Centiles[%] Segment 10	centile	56	0.357143	0.278351
Inter-operator	RV 24-Segment FS Centiles[%] Segment 11	centile	56	0.357143	0.266055
Inter-operator	RV 24-Segment FS Centiles[%] Segment 2	centile	56	0.357143	0.247312
Inter-operator	RV 24-Segment FS Centiles[%] Segment 6	centile	56	0.357143	0.278351
Intra-operator	LV_ Basal-Sep.Wall FS.Lt Basal-Sep FS % (GA)[%]	centile	28	0.357143	0.054054
Intra-operator	LV_ MAPSE lateral.MAPSE lat. % (GA)[%]	centile	28	0.357143	0
Inter-operator	LV_ SV.Lt SV % (GA)[%]	centile	51	0.352941	0.26087
Intra-operator	LV_ CO.Lt CO % (FL)[%]	centile	17	0.352941	0.238806
Inter-operator	LV_ Basal-Lat.Wall FS.Lt Basal-Lat FS % (AUA)[%]	centile	54	0.351852	-0.125
Inter-operator	LV_ MAPSE lateral.MAPSE lat. % (AUA)[%]	centile	54	0.351852	-0.051948
Inter-operator	LV_ Basal-Lat.Wall FS.Lt Basal-Lat FS % (BPD)[%]	centile	55	0.345455	-0.142433
Inter-operator	LV_ Frac. Area change.Lt FRAC % (BPD)[%]	centile	55	0.345455	-0.024845
Inter-operator	LV_ Frac. Area change.Lt FRAC % (HC)[%]	centile	55	0.345455	-0.018519
Inter-operator	RV_ Basal-Lat.Wall FS.Rt Basal-Lat FS % (FL)[%]	centile	55	0.345455	-0.029963
Intra-operator	LV_ Basal-Sep.Wall FS.Lt Basal-Sep FS % (FL)[%]	centile	29	0.344828	0.198895
Intra-operator	LV_ ED Length.Lt ED Length % (FL)[%]	centile	29	0.344828	0.32243
Inter-operator	LV_ SV/KG.Lt SV/KG % (GA)[%]	centile	50	0.34	0.233129
Inter-operator	LV 24-Segment FS Centiles[%] Segment 22	centile	56	0.339286	0.331658
Inter-operator	LV 24-Segment FS Centiles[%] Segment 23	centile	56	0.339286	0.331658
Inter-operator	LV 24-Segment FS Centiles[%] Segment 5	centile	56	0.339286	-0.037037
Inter-operator	RV_ Global Strain.Rt GLS %[%]	centile	56	0.339286	-0.053763
Inter-operator	LV_ Basal-Lat.Wall FS.Lt Basal-Lat FS % (GA)[%]	centile	51	0.333333	-0.072072
Inter-operator	LV_ EF.Lt EF % (GA)[%]	centile	51	0.333333	-0.02
Inter-operator	RV_ Basal-Lat.Wall FS.Rt Basal-Lat FS % (AUA)[%]	centile	54	0.333333	-0.030534
Intra-operator	LV 24-Segment FS Centiles[%] Segment 13	centile	30	0.333333	0.264706
Intra-operator	LV 24-Segment FS Centiles[%] Segment 15	centile	30	0.333333	0.253731
Intra-operator	LV 24-Segment SI Centiles[%] Segment 10	centile	30	0.333333	0
Intra-operator	LV 24-Segment SI Centiles[%] Segment 11	centile	30	0.333333	0
Intra-operator	LV 24-Segment SI Centiles[%] Segment 2	centile	30	0.333333	0
Intra-operator	LV 24-Segment SI Centiles[%] Segment 5	centile	30	0.333333	-0.034483
Intra-operator	LV 24-Segment SI Centiles[%] Segment 6	centile	30	0.333333	-0.034483
Intra-operator	LV 24-Segment SI Centiles[%] Segment 7	centile	30	0.333333	-0.034483
Intra-operator	LV 24-Segment SI Centiles[%] Segment 8	centile	30	0.333333	-0.034483
Intra-operator	LV 24-Segment SI Centiles[%] Segment 9	centile	30	0.333333	0
Intra-operator	LV_ Basal-Sep.Wall FS.Lt Basal-Sep FS % (AC)[%]	centile	30	0.333333	0.210526
Intra-operator	LV_ Basal-Sep.Wall FS.Lt Basal-Sep FS % (AUA)[%]	centile	30	0.333333	0.210526
Intra-operator	LV_ ED Length.Lt ED Length % (BPD)[%]	centile	30	0.333333	0.333333
Intra-operator	LV_ MAPSE lateral.MAPSE lat. % (AUA)[%]	centile	30	0.333333	-0.052632

group	parameter	scale	usable_n	max_flip_rate	min_kappa
Intra-operator	LV_ MAPSE lateral.MAPSE lat. % (BPD)[%]	centile	30	0.333333	-0.052632
Intra-operator	LV_ MAPSE lateral.MAPSE lat. % (EFW)[%]	centile	30	0.333333	-0.052632
Intra-operator	LV_ SV/KG.Lt SV/KG % (BPD)[%]	centile	30	0.333333	0.253731
Intra-operator	RV 24-Segment FS Centiles[%] Segment 12	centile	30	0.333333	0.222798
Intra-operator	RV 24-Segment SI Centiles[%] Segment 4	centile	30	0.333333	-0.046512
Intra-operator	RV 24-Segment SI Centiles[%] Segment 5	centile	30	0.333333	-0.071429
Intra-operator	RV 24-Segment SI Centiles[%] Segment 6	centile	30	0.333333	-0.046512
Intra-operator	RV_ ED Length.Rt ED Length % (AC)[%]	centile	30	0.333333	0.342105
Inter-operator	LV_ Basal-Lat.Wall FS.Lt Basal-Lat FS % (EFW)[%]	centile	55	0.327273	-0.122449
Inter-operator	LV_ MAPSE lateral.MAPSE lat. % (BPD)[%]	centile	55	0.327273	-0.029963
Inter-operator	LV_ MAPSE lateral.MAPSE lat. % (EFW)[%]	centile	55	0.327273	-0.029963
Inter-operator	RV_ Basal-Lat.Wall FS.Rt Basal-Lat FS % (EFW)[%]	centile	55	0.327273	-0.029963
Inter-operator	RV_ TAPSE lateral.TAPSE lat. % (EFW)[%]	centile	55	0.327273	-0.028037
Inter-operator	LV 24-Segment FS Centiles[%] Segment 2	centile	56	0.321429	-0.02439
Inter-operator	LV 24-Segment FS Centiles[%] Segment 24	centile	56	0.321429	0.363636
Inter-operator	LV 24-Segment FS Centiles[%] Segment 3	centile	56	0.321429	-0.02439
Inter-operator	LV_ Basal-Apical FS.Lt BASE-AP FS %[%]	centile	56	0.321429	0.213115
Inter-operator	RV 24-Segment FS Centiles[%] Segment 1	centile	56	0.321429	0.3
Intra-operator	LV_ ED Area.Lt ED Area % (GA)[%]	centile	28	0.321429	0.240964
Intra-operator	LV_ ED Length.Lt ED Length % (GA)[%]	centile	28	0.321429	0.363636
Intra-operator	RV_ ED Length.Rt ED Length % (GA)[%]	centile	28	0.321429	0.376238
Inter-operator	LV_ EF.Lt EF % (AUA)[%]	centile	54	0.314815	0
Inter-operator	LV_ Frac. Area change.Lt FRAC % (AUA)[%]	centile	54	0.314815	-0.018868
Inter-operator	LV_ CO.Lt CO % (BPD)[%]	centile	51	0.313725	-0.026846
Inter-operator	LV_ MAPSE septal.MAPSE sept. % (GA)[%]	centile	51	0.313725	0.198953
Inter-operator	RV_ TAPSE lateral.TAPSE lat. % (GA)[%]	centile	51	0.313725	-0.030303
Intra-operator	RV_ ED Length.Rt ED Length % (FL)[%]	centile	29	0.310345	0.382979
Inter-operator	LV_ Frac. Area change.Lt FRAC % (FL)[%]	centile	55	0.309091	-0.018519
Inter-operator	LV_ MAPSE lateral.MAPSE lat. % (HC)[%]	centile	55	0.309091	-0.050955
Intra-operator	LV_ CO/KG.Lt CO/KG % (HC)[%]	centile	13	0.307692	0.35
Inter-operator	LV_ FreeWall Strain.Lt FWS %[%]	centile	56	0.303571	0.196172
Inter-operator	RV 24-Segment FS Centiles[%] Segment 3	centile	56	0.303571	0.3
Inter-operator	RV 24-Segment FS Centiles[%] Segment 4	centile	56	0.303571	0.23913
Inter-operator	RV 24-Segment FS Centiles[%] Segment 5	centile	56	0.303571	0.23913
Inter-operator	RV 24-Segment SI Centiles[%] Segment 1	centile	56	0.303571	-0.027523
Inter-operator	RV_ TAPSE lateral.TAPSE lat. % (AC)[%]	centile	56	0.303571	-0.027523
Inter-operator	LV_ SV.Lt SV % (FL)[%]	centile	33	0.30303	0
Intra-operator	LV 24-Segment FS Centiles[%] Segment 14	centile	30	0.3	0.314721
Intra-operator	LV 24-Segment FS Centiles[%] Segment 16	centile	30	0.3	0.341463
Intra-operator	LV 24-Segment FS Centiles[%] Segment 22	centile	30	0.3	0.4

group	parameter	scale	usable_n	max_flip_rate	min_kappa
Intra-operator	LV 24-Segment FS Centiles[%] Segment 5	centile	30	0.3	0
Intra-operator	LV 24-Segment FS Centiles[%] Segment 6	centile	30	0.3	0
Intra-operator	LV 24-Segment SI Centiles[%] Segment 1	centile	30	0.3	0
Intra-operator	LV_ Basal-Sep.Wall FS.Lt Basal-Sep FS % (BPD)[%]	centile	30	0.3	0.307692
Intra-operator	LV_ Basal-Sep.Wall FS.Lt Basal-Sep FS % (EFW)[%]	centile	30	0.3	0.341463
Intra-operator	LV_ Basal-Sep.Wall FS.Lt Basal-Sep FS % (HC)[%]	centile	30	0.3	0.307692
Intra-operator	LV_ ED Area.Lt ED Area % (EFW)[%]	centile	30	0.3	0
Intra-operator	LV_ ED Length.Lt ED Length % (HC)[%]	centile	30	0.3	0.38914
Intra-operator	LV_ FreeWall Strain.Lt FWS %[%]	centile	30	0.3	0.341463
Intra-operator	LV_ MAPSE lateral.MAPSE lat. % (AC)[%]	centile	30	0.3	-0.052632
Intra-operator	LV_ MAPSE lateral.MAPSE lat. % (HC)[%]	centile	30	0.3	-0.052632
Intra-operator	LV_ MAPSE septal.MAPSE sept. % (AC)[%]	centile	30	0.3	0.268293
Intra-operator	LV_ SV/KG.Lt SV/KG % (AUA)[%]	centile	30	0.3	0.341463
Intra-operator	RV 24-Segment FS Centiles[%] Segment 20	centile	30	0.3	0.4
Intra-operator	RV 24-Segment FS Centiles[%] Segment 21	centile	30	0.3	0.4
Intra-operator	RV 24-Segment SI Centiles[%] Segment 10	centile	30	0.3	-0.071429
Intra-operator	RV 24-Segment SI Centiles[%] Segment 9	centile	30	0.3	-0.062992
Intra-operator	RV_ Basal-Apical FS.Rt BASE-AP FS %[%]	centile	30	0.3	-0.071429
Intra-operator	RV_ Basal-Lat.Wall FS.Rt Basal-Lat FS % (AC)[%]	centile	30	0.3	0
Intra-operator	RV_ Basal-Lat.Wall FS.Rt Basal-Lat FS % (AUA)[%]	centile	30	0.3	0
Intra-operator	RV_ Basal-Lat.Wall FS.Rt Basal-Lat FS % (HC)[%]	centile	30	0.3	0
Intra-operator	RV_ ED Area.Rt ED Area % (BPD)[%]	centile	30	0.3	0.372093
Intra-operator	RV_ ED Length.Rt ED Length % (EFW)[%]	centile	30	0.3	0.405286
Inter-operator	LV_ CO.Lt CO % (GA)[%]	centile	51	0.294118	0.27762
Intra-operator	LV_ CO/KG.Lt CO/KG % (FL)[%]	centile	17	0.294118	0.330709
Inter-operator	LV_ ED Length.Lt ED Length % (AC)[%]	centile	56	0.285714	0.423423
Inter-operator	LV_ EDV.Lt EDV % (HC)[%]	centile	14	0.285714	0.176471
Inter-operator	LV_ EF.Lt EF % (HC)[%]	centile	14	0.285714	0.317073
Inter-operator	LV_ MAPSE septal.MAPSE sept. % (AC)[%]	centile	56	0.285714	0.270833
Inter-operator	RV 24-Segment FS Centiles[%] Segment 14	centile	56	0.285714	0
Inter-operator	RV 24-Segment SI Centiles[%] Segment 3	centile	56	0.285714	-0.027523
Inter-operator	RV 24-Segment SI Centiles[%] Segment 4	centile	56	0.285714	-0.027523
Inter-operator	RV 24-Segment SI Centiles[%] Segment 5	centile	56	0.285714	-0.044776
Inter-operator	RV 24-Segment SI Centiles[%] Segment 6	centile	56	0.285714	-0.044776
Inter-operator	RV_ FreeWall Strain.Rt FWS %[%]	centile	56	0.285714	0.247312
Inter-operator	RV_ TAPSE lateral.TAPSE lat. % (FL)[%]	centile	56	0.285714	-0.02439
Intra-operator	LV_ Basal-Lat.Wall FS.Lt Basal-Lat FS % (GA)[%]	centile	28	0.285714	0.16
Intra-operator	LV_ CO/KG.Lt CO/KG % (GA)[%]	centile	28	0.285714	0.36
Intra-operator	LV_ SV/KG.Lt SV/KG % (GA)[%]	centile	28	0.285714	0.381215
Intra-operator	RV_ Basal-Lat.Wall FS.Rt Basal-Lat FS % (GA)[%]	centile	28	0.285714	0

group	parameter	scale	usable_n	max_flip_rate	min_kappa
Intra-operator	LV_ MAPSE lateral.MAPSE lat. % (FL)[%]	centile	29	0.275862	-0.054545
Intra-operator	RV_ Basal-Lat.Wall FS.Rt Basal-Lat FS % (FL)[%]	centile	29	0.275862	0
Intra-operator	RV_ TAPSE lateral.TAPSE lat. % (FL)[%]	centile	29	0.275862	0
Inter-operator	LV_ Basal-Sep.Wall FS.Lt Basal-Sep FS % (GA)[%]	centile	51	0.27451	0.220917
Inter-operator	LV_ EF.Lt EF % (BPD)[%]	centile	51	0.27451	-0.02
Inter-operator	LV_ MAPSE lateral.MAPSE lat. % (GA)[%]	centile	51	0.27451	-0.032389
Inter-operator	LV_ Basal-Sep.Wall FS.Lt Basal-Sep FS % (FL)[%]	centile	55	0.272727	0.318182
Inter-operator	LV_ ED Length.Lt ED Length % (BPD)[%]	centile	55	0.272727	0.456163
Inter-operator	LV_ ED Length.Lt ED Length % (EFW)[%]	centile	55	0.272727	0.440678
Inter-operator	LV_ ED Length.Lt ED Length % (HC)[%]	centile	55	0.272727	0.456163
Inter-operator	LV_ Frac. Area change.Lt FRAC % (EFW)[%]	centile	55	0.272727	-0.024845
Inter-operator	LV_ MAPSE septal.MAPSE sept. % (HC)[%]	centile	55	0.272727	0.300578
Inter-operator	RV_ ED Length.Rt ED Length % (HC)[%]	centile	55	0.272727	0
Inter-operator	RV_ TAPSE lateral.TAPSE lat. % (BPD)[%]	centile	55	0.272727	-0.024845
Inter-operator	LV_ MAPSE septal.MAPSE sept. % (FL)[%]	centile	56	0.267857	0.237624
Inter-operator	RV 24-Segment FS Centiles[%] Segment 22	centile	56	0.267857	-0.018182
Inter-operator	RV 24-Segment FS Centiles[%] Segment 23	centile	56	0.267857	-0.018182
Inter-operator	RV 24-Segment FS Centiles[%] Segment 24	centile	56	0.267857	-0.018182
Inter-operator	RV 24-Segment SI Centiles[%] Segment 7	centile	56	0.267857	0.182879
Intra-operator	LV 24-Segment FS Centiles[%] Segment 17	centile	30	0.266667	0.425837
Intra-operator	LV 24-Segment FS Centiles[%] Segment 23	centile	30	0.266667	0.466667
Intra-operator	LV 24-Segment FS Centiles[%] Segment 24	centile	30	0.266667	0.466667
Intra-operator	LV 24-Segment FS Centiles[%] Segment 4	centile	30	0.266667	0
Intra-operator	LV 24-Segment SI Centiles[%] Segment 16	centile	30	0.266667	-0.111111
Intra-operator	LV_ CO/KG.Lt CO/KG % (AUA)[%]	centile	30	0.266667	0.368421
Intra-operator	LV_ CO/KG.Lt CO/KG % (BPD)[%]	centile	30	0.266667	0.322034
Intra-operator	LV_ ED Area.Lt ED Area % (AUA)[%]	centile	30	0.266667	0.368421
Intra-operator	LV_ ED Area.Lt ED Area % (BPD)[%]	centile	30	0.266667	0.368421
Intra-operator	LV_ ED Area.Lt ED Area % (HC)[%]	centile	30	0.266667	0
Intra-operator	LV_ EDV.Lt EDV % (AUA)[%]	centile	30	0.266667	0.347826
Intra-operator	RV 24-Segment FS Centiles[%] Segment 19	centile	30	0.266667	0.459459
Intra-operator	RV 24-Segment SI Centiles[%] Segment 1	centile	30	0.266667	-0.034483
Intra-operator	RV 24-Segment SI Centiles[%] Segment 11	centile	30	0.266667	-0.071429
Intra-operator	RV 24-Segment SI Centiles[%] Segment 2	centile	30	0.266667	-0.034483
Intra-operator	RV 24-Segment SI Centiles[%] Segment 3	centile	30	0.266667	-0.046512
Intra-operator	RV_ Basal-Lat.Wall FS.Rt Basal-Lat FS % (BPD)[%]	centile	30	0.266667	0
Intra-operator	RV_ ED Area.Rt ED Area % (AUA)[%]	centile	30	0.266667	0.366197
Intra-operator	RV_ ED Length.Rt ED Length % (AUA)[%]	centile	30	0.266667	0.466667
Intra-operator	RV_ ED Length.Rt ED Length % (BPD)[%]	centile	30	0.266667	0
Intra-operator	RV_ FreeWall Strain.Rt FWS %[%]	centile	30	0.266667	-0.046512

group	parameter	scale	usable_n	max_flip_rate	min_kappa
Intra-operator	RV_ TAPSE lateral.TAPSE lat. % (AC)[%]	centile	30	0.266667	0
Intra-operator	RV_ TAPSE lateral.TAPSE lat. % (AUA)[%]	centile	30	0.266667	0
Intra-operator	RV_ TAPSE lateral.TAPSE lat. % (BPD)[%]	centile	30	0.266667	0
Intra-operator	RV_ TAPSE lateral.TAPSE lat. % (EFW)[%]	centile	30	0.266667	0
Intra-operator	RV_ TAPSE lateral.TAPSE lat. % (HC)[%]	centile	30	0.266667	0
Intra-operator	RV_ TAPSE septal.TAPSE sept. % (EFW)[%]	centile	30	0.266667	-0.034483
Inter-operator	LV_ CO.Lt CO % (EFW)[%]	centile	19	0.263158	0.469274
Inter-operator	LV_ SV/KG.Lt SV/KG % (EFW)[%]	centile	19	0.263158	0.475138
Inter-operator	LV_ ED Length.Lt ED Length % (AUA)[%]	centile	54	0.259259	0.476454
Inter-operator	LV_ EDV.Lt EDV % (AUA)[%]	centile	54	0.259259	0.414634
Inter-operator	LV_ SV.Lt SV % (AUA)[%]	centile	54	0.259259	0.326203
Inter-operator	RV_ TAPSE lateral.TAPSE lat. % (AUA)[%]	centile	54	0.259259	-0.028571
Inter-operator	LV_ Basal-Sep.Wall FS.Lt Basal-Sep FS % (BPD)[%]	centile	55	0.254545	0.318182
Inter-operator	LV_ Basal-Sep.Wall FS.Lt Basal-Sep FS % (EFW)[%]	centile	55	0.254545	0.352941
Inter-operator	LV_ Basal-Sep.Wall FS.Lt Basal-Sep FS % (HC)[%]	centile	55	0.254545	0.318182
Inter-operator	LV_ ED Length.Lt ED Length % (FL)[%]	centile	55	0.254545	0.47973
Inter-operator	LV_ MAPSE septal.MAPSE sept. % (BPD)[%]	centile	55	0.254545	0.235145
Inter-operator	RV_ TAPSE lateral.TAPSE lat. % (HC)[%]	centile	55	0.254545	-0.024845
Inter-operator	LV_ Basal-Sep.Wall FS.Lt Basal-Sep FS % (AC)[%]	centile	56	0.25	0.363636
Inter-operator	LV_ CO.Lt CO % (AC)[%]	centile	28	0.25	0.5
Inter-operator	RV 24-Segment FS Centiles[%] Segment 15	centile	56	0.25	0
Inter-operator	RV 24-Segment FS Centiles[%] Segment 19	centile	56	0.25	-0.02439
Inter-operator	RV 24-Segment SI Centiles[%] Segment 2	centile	56	0.25	-0.027523
Inter-operator	RV 24-Segment SI Centiles[%] Segment 8	centile	56	0.25	0.155172
Inter-operator	RV 24-Segment SI Centiles[%] Segment 9	centile	56	0.25	0.155172
Intra-operator	LV_ CO.Lt CO % (EFW)[%]	centile	16	0.25	0.428571
Intra-operator	LV_ EDV.Lt EDV % (EFW)[%]	centile	16	0.25	0.179487
Intra-operator	LV_ EDV.Lt EDV % (GA)[%]	centile	28	0.25	0.484211
Intra-operator	RV_ ED Area.Rt ED Area % (GA)[%]	centile	28	0.25	0.473118
Intra-operator	RV_ Frac. Area change.Rt FRAC % (GA)[%]	centile	28	0.25	-0.139535
Intra-operator	RV_ TAPSE lateral.TAPSE lat. % (GA)[%]	centile	28	0.25	0
Intra-operator	RV_ TAPSE septal.TAPSE sept. % (GA)[%]	centile	28	0.25	-0.037037
Inter-operator	LV_ EDV.Lt EDV % (FL)[%]	centile	33	0.242424	0.352941
Intra-operator	RV_ Frac. Area change.Rt FRAC % (FL)[%]	centile	29	0.241379	-0.134078
Inter-operator	LV_ Basal-Sep.Wall FS.Lt Basal-Sep FS % (AUA)[%]	centile	54	0.240741	0.357143
Inter-operator	RV_ ED Length.Rt ED Length % (AUA)[%]	centile	54	0.240741	0
Inter-operator	RV_ ED Length.Rt ED Length % (EFW)[%]	centile	55	0.236364	0
Inter-operator	RV_ Frac. Area change.Rt FRAC % (BPD)[%]	centile	55	0.236364	0
Inter-operator	RV_ Frac. Area change.Rt FRAC % (EFW)[%]	centile	55	0.236364	0
Inter-operator	RV_ Frac. Area change.Rt FRAC % (FL)[%]	centile	55	0.236364	0

group	parameter	scale	usable_n	max_flip_rate	min_kappa
Inter-operator	RV_ Frac. Area change.Rt FRAC % (HC)[%]	centile	55	0.236364	0
Inter-operator	RV_ Frac. Area change.Rt FRAC % (GA)[%]	centile	51	0.235294	0
Intra-operator	LV_ EDV.Lt EDV % (FL)[%]	centile	17	0.235294	0.392857
Intra-operator	LV_ SV.Lt SV % (FL)[%]	centile	17	0.235294	0.521127
Intra-operator	LV 24-Segment SI Centiles[%] Segment 17	centile	30	0.233333	-0.105263
Intra-operator	LV_ Basal-Lat.Wall FS.Lt Basal-Lat FS % (EFW)[%]	centile	30	0.233333	0.166667
Intra-operator	LV_ ED Area.Lt ED Area % (AC)[%]	centile	30	0.233333	0.086957
Intra-operator	LV_ EDV.Lt EDV % (BPD)[%]	centile	30	0.233333	0.524887
Intra-operator	RV 24-Segment FS Centiles[%] Segment 22	centile	30	0.233333	0.533333
Intra-operator	RV 24-Segment SI Centiles[%] Segment 21	centile	30	0.233333	-0.129032
Intra-operator	RV 24-Segment SI Centiles[%] Segment 22	centile	30	0.233333	-0.129032
Intra-operator	RV 24-Segment SI Centiles[%] Segment 23	centile	30	0.233333	-0.129032
Intra-operator	RV 24-Segment SI Centiles[%] Segment 24	centile	30	0.233333	-0.129032
Intra-operator	RV 24-Segment SI Centiles[%] Segment 7	centile	30	0.233333	-0.046512
Intra-operator	RV 24-Segment SI Centiles[%] Segment 8	centile	30	0.233333	-0.046512
Intra-operator	RV_ Basal-Lat.Wall FS.Rt Basal-Lat FS % (EFW)[%]	centile	30	0.233333	0
Intra-operator	RV_ ED Length.Rt ED Length % (HC)[%]	centile	30	0.233333	0.524887
Intra-operator	RV_ Frac. Area change.Rt FRAC % (AC)[%]	centile	30	0.233333	-0.129032
Intra-operator	RV_ Frac. Area change.Rt FRAC % (AUA)[%]	centile	30	0.233333	-0.129032
Intra-operator	RV_ Frac. Area change.Rt FRAC % (BPD)[%]	centile	30	0.233333	-0.129032
Intra-operator	RV_ Frac. Area change.Rt FRAC % (EFW)[%]	centile	30	0.233333	-0.129032
Intra-operator	RV_ Frac. Area change.Rt FRAC % (HC)[%]	centile	30	0.233333	-0.129032
Intra-operator	RV_ TAPSE septal.TAPSE sept. % (AC)[%]	centile	30	0.233333	-0.034483
Intra-operator	RV_ TAPSE septal.TAPSE sept. % (BPD)[%]	centile	30	0.233333	-0.034483
Intra-operator	RV_ TAPSE septal.TAPSE sept. % (HC)[%]	centile	30	0.233333	-0.034483
Inter-operator	LV 24-Segment SI Centiles[%] Segment 24	centile	56	0.232143	-0.02439
Inter-operator	RV 24-Segment FS Centiles[%] Segment 20	centile	56	0.232143	0.486239
Inter-operator	RV 24-Segment FS Centiles[%] Segment 21	centile	56	0.232143	0.538071
Inter-operator	RV 24-Segment SI Centiles[%] Segment 10	centile	56	0.232143	0.1875
Inter-operator	RV_ ED Length.Rt ED Length % (AC)[%]	centile	56	0.232143	-0.018182
Inter-operator	RV_ Frac. Area change.Rt FRAC % (AC)[%]	centile	56	0.232143	0
Intra-operator	LV_ CO.Lt CO % (HC)[%]	centile	13	0.230769	0.493506
Intra-operator	LV_ EDV.Lt EDV % (HC)[%]	centile	13	0.230769	-0.114286
Intra-operator	LV_ SV.Lt SV % (HC)[%]	centile	13	0.230769	0.493506
Intra-operator	LV_ SV/KG.Lt SV/KG % (HC)[%]	centile	13	0.230769	0.541176
Inter-operator	RV_ Frac. Area change.Rt FRAC % (AUA)[%]	centile	54	0.222222	0
Inter-operator	LV_ MAPSE septal.MAPSE sept. % (EFW)[%]	centile	55	0.218182	0.268617
Inter-operator	RV_ ED Area.Rt ED Area % (BPD)[%]	centile	55	0.218182	0.319588
Inter-operator	RV_ ED Length.Rt ED Length % (BPD)[%]	centile	55	0.218182	0
Inter-operator	RV_ ED Length.Rt ED Length % (FL)[%]	centile	55	0.218182	0

group	parameter	scale	usable_n	max_flip_rate	min_kappa
Inter-operator	LV_ ED Length.Lt ED Length % (GA)[%]	centile	51	0.215686	0.559309
Inter-operator	LV_ EDV.Lt EDV % (BPD)[%]	centile	51	0.215686	0.406977
Inter-operator	RV_ ED Length.Rt ED Length % (GA)[%]	centile	51	0.215686	0
Inter-operator	LV 24-Segment SI Centiles[%] Segment 14	centile	56	0.214286	0.155172
Inter-operator	LV 24-Segment SI Centiles[%] Segment 8	centile	56	0.214286	-0.044776
Inter-operator	LV_ SV.Lt SV % (HC)[%]	centile	14	0.214286	0.571429
Inter-operator	RV 24-Segment FS Centiles[%] Segment 16	centile	56	0.214286	0
Inter-operator	RV 24-Segment FS Centiles[%] Segment 18	centile	56	0.214286	0
Inter-operator	RV 24-Segment SI Centiles[%] Segment 11	centile	56	0.214286	0.222222
Inter-operator	RV 24-Segment SI Centiles[%] Segment 13	centile	56	0.214286	0.127273
Inter-operator	RV_ Sept.Wall Strain.Rt SWS %[%]	centile	56	0.214286	0.23913
Inter-operator	LV_ EDV.Lt EDV % (EFW)[%]	centile	19	0.210526	0.377049
Intra-operator	LV_ EDV.Lt EDV % (AC)[%]	centile	19	0.210526	0.441176
Intra-operator	LV_ Basal-Lat.Wall FS.Lt Basal-Lat FS % (FL)[%]	centile	29	0.206897	0.20765
Intra-operator	LV_ ED Area.Lt ED Area % (FL)[%]	centile	29	0.206897	0
Intra-operator	RV_ ED Area.Rt ED Area % (FL)[%]	centile	29	0.206897	0.462963
Intra-operator	RV_ TAPSE septal.TAPSE sept. % (FL)[%]	centile	29	0.206897	-0.035714
Inter-operator	LV_ CO.Lt CO % (AUA)[%]	centile	54	0.203704	0.395112
Inter-operator	RV_ ED Area.Rt ED Area % (HC)[%]	centile	55	0.2	0.460303
Intra-operator	LV 24-Segment FS Centiles[%] Segment 19	centile	30	0.2	0.577465
Intra-operator	LV 24-Segment FS Centiles[%] Segment 20	centile	30	0.2	0.577465
Intra-operator	LV 24-Segment FS Centiles[%] Segment 21	centile	30	0.2	0.577465
Intra-operator	LV 24-Segment SI Centiles[%] Segment 18	centile	30	0.2	-0.097561
Intra-operator	LV 24-Segment SI Centiles[%] Segment 22	centile	30	0.2	-0.034483
Intra-operator	LV 24-Segment SI Centiles[%] Segment 23	centile	30	0.2	-0.034483
Intra-operator	LV 24-Segment SI Centiles[%] Segment 24	centile	30	0.2	-0.034483
Intra-operator	LV_ Basal-Apical FS.Lt BASE-AP FS %[%]	centile	30	0.2	0.142857
Intra-operator	LV_ Basal-Lat.Wall FS.Lt Basal-Lat FS % (AC)[%]	centile	30	0.2	0.166667
Intra-operator	LV_ Basal-Lat.Wall FS.Lt Basal-Lat FS % (AUA)[%]	centile	30	0.2	0.166667
Intra-operator	LV_ Basal-Lat.Wall FS.Lt Basal-Lat FS % (BPD)[%]	centile	30	0.2	0.166667
Intra-operator	LV_ Basal-Lat.Wall FS.Lt Basal-Lat FS % (HC)[%]	centile	30	0.2	0.166667
Intra-operator	LV_ CO.Lt CO % (BPD)[%]	centile	30	0.2	0.444444
Intra-operator	LV_ EF.Lt EF % (AUA)[%]	centile	30	0.2	0.444444
Intra-operator	LV_ EF.Lt EF % (BPD)[%]	centile	30	0.2	0.444444
Intra-operator	LV_ Frac. Area change.Lt FRAC % (EFW)[%]	centile	30	0.2	0.37931
Intra-operator	RV 24-Segment FS Centiles[%] Segment 23	centile	30	0.2	0.594595
Intra-operator	RV 24-Segment FS Centiles[%] Segment 24	centile	30	0.2	0.594595
Intra-operator	RV 24-Segment SI Centiles[%] Segment 12	centile	30	0.2	0
Intra-operator	RV 24-Segment SI Centiles[%] Segment 13	centile	30	0.2	0
Intra-operator	RV 24-Segment SI Centiles[%] Segment 14	centile	30	0.2	0

group	parameter	scale	usable_n	max_flip_rate	min_kappa
Intra-operator	RV 24-Segment SI Centiles[%] Segment 20	centile	30	0.2	-0.097561
Intra-operator	RV_ ED Area.Rt ED Area % (AC)[%]	centile	30	0.2	0.444444
Intra-operator	RV_ Global Strain.Rt GLS %[%]	centile	30	0.2	-0.046512
Intra-operator	RV_ TAPSE septal.TAPSE sept. % (AUA)[%]	centile	30	0.2	-0.034483
Inter-operator	LV 24-Segment SI Centiles[%] Segment 1	centile	56	0.196429	0
Inter-operator	LV 24-Segment SI Centiles[%] Segment 15	centile	56	0.196429	0.167568
Inter-operator	LV 24-Segment SI Centiles[%] Segment 16	centile	56	0.196429	0.060976
Inter-operator	LV 24-Segment SI Centiles[%] Segment 22	centile	56	0.196429	-0.02439
Inter-operator	LV 24-Segment SI Centiles[%] Segment 23	centile	56	0.196429	-0.02439
Inter-operator	RV 24-Segment FS Centiles[%] Segment 17	centile	56	0.196429	0
Inter-operator	RV 24-Segment SI Centiles[%] Segment 21	centile	56	0.196429	-0.037037
Inter-operator	RV_ ED Area.Rt ED Area % (GA)[%]	centile	51	0.196078	0.379562
Intra-operator	LV_ CO/KG.Lt CO/KG % (EFW)[%]	centile	16	0.1875	0.538462
Inter-operator	RV_ ED Area.Rt ED Area % (AUA)[%]	centile	54	0.185185	0.274194
Inter-operator	RV_ ED Area.Rt ED Area % (FL)[%]	centile	55	0.181818	0.337349
Inter-operator	LV 24-Segment SI Centiles[%] Segment 10	centile	56	0.178571	-0.05
Inter-operator	LV 24-Segment SI Centiles[%] Segment 11	centile	56	0.178571	-0.07177
Inter-operator	LV 24-Segment SI Centiles[%] Segment 12	centile	56	0.178571	0.196172
Inter-operator	LV 24-Segment SI Centiles[%] Segment 13	centile	56	0.178571	0.196172
Inter-operator	LV 24-Segment SI Centiles[%] Segment 2	centile	56	0.178571	-0.018182
Inter-operator	LV 24-Segment SI Centiles[%] Segment 21	centile	56	0.178571	-0.027523
Inter-operator	LV 24-Segment SI Centiles[%] Segment 7	centile	56	0.178571	-0.037037
Inter-operator	LV 24-Segment SI Centiles[%] Segment 9	centile	56	0.178571	-0.044776
Inter-operator	LV_ EDV.Lt EDV % (AC)[%]	centile	28	0.178571	0.615385
Inter-operator	RV 24-Segment SI Centiles[%] Segment 12	centile	56	0.178571	0.278351
Inter-operator	RV 24-Segment SI Centiles[%] Segment 14	centile	56	0.178571	0.183673
Inter-operator	RV 24-Segment SI Centiles[%] Segment 20	centile	56	0.178571	-0.037037
Intra-operator	LV_ CO.Lt CO % (GA)[%]	centile	28	0.178571	0.339623
Intra-operator	LV_ EF.Lt EF % (GA)[%]	centile	28	0.178571	0.551282
Intra-operator	LV_ Frac. Area change.Lt FRAC % (GA)[%]	centile	28	0.178571	0.551282
Inter-operator	LV_ ED Area.Lt ED Area % (GA)[%]	centile	51	0.176471	0.49505
Inter-operator	LV_ EDV.Lt EDV % (GA)[%]	centile	51	0.176471	0.337662
Intra-operator	LV_ SV/KG.Lt SV/KG % (FL)[%]	centile	17	0.176471	0.627737
Intra-operator	LV 24-Segment FS Centiles[%] Segment 18	centile	30	0.166667	0.637681
Intra-operator	LV 24-Segment SI Centiles[%] Segment 19	centile	30	0.166667	-0.086957
Intra-operator	LV 24-Segment SI Centiles[%] Segment 20	centile	30	0.166667	-0.086957
Intra-operator	LV 24-Segment SI Centiles[%] Segment 21	centile	30	0.166667	-0.046512
Intra-operator	LV_ Frac. Area change.Lt FRAC % (AC)[%]	centile	30	0.166667	0.561404
Intra-operator	LV_ Frac. Area change.Lt FRAC % (AUA)[%]	centile	30	0.166667	0.509804
Intra-operator	LV_ Frac. Area change.Lt FRAC % (BPD)[%]	centile	30	0.166667	0.509804

group	parameter	scale	usable_n	max_flip_rate	min_kappa
Intra-operator	LV_ SV.Lt SV % (AUA)[%]	centile	30	0.166667	0.509804
Intra-operator	LV_ Sept.Wall Strain.Lt SWS %[%]	centile	30	0.166667	-0.086957
Intra-operator	RV 24-Segment SI Centiles[%] Segment 15	centile	30	0.166667	-0.052632
Intra-operator	RV 24-Segment SI Centiles[%] Segment 18	centile	30	0.166667	-0.071429
Intra-operator	RV_ ED Area.Rt ED Area % (EFW)[%]	centile	30	0.166667	0.444444
Inter-operator	LV 24-Segment SI Centiles[%] Segment 20	centile	56	0.160714	-0.029412
Inter-operator	LV 24-Segment SI Centiles[%] Segment 3	centile	56	0.160714	-0.02439
Inter-operator	LV 24-Segment SI Centiles[%] Segment 4	centile	56	0.160714	-0.02439
Inter-operator	LV 24-Segment SI Centiles[%] Segment 6	centile	56	0.160714	-0.037037
Inter-operator	RV 24-Segment SI Centiles[%] Segment 15	centile	56	0.160714	0.217391
Inter-operator	RV 24-Segment SI Centiles[%] Segment 19	centile	56	0.160714	-0.044776
Intra-operator	LV_ CO/KG.Lt CO/KG % (AC)[%]	centile	19	0.157895	0.564885
Inter-operator	LV_ SV.Lt SV % (BPD)[%]	centile	51	0.156863	0.606936
Inter-operator	LV_ ED Area.Lt ED Area % (FL)[%]	centile	55	0.145455	0.468599
Inter-operator	RV_ ED Area.Rt ED Area % (EFW)[%]	centile	55	0.145455	0.395604
Inter-operator	LV 24-Segment SI Centiles[%] Segment 17	centile	56	0.142857	0.135135
Inter-operator	LV 24-Segment SI Centiles[%] Segment 18	centile	56	0.142857	0.135135
Inter-operator	LV 24-Segment SI Centiles[%] Segment 19	centile	56	0.142857	0.207547
Inter-operator	LV 24-Segment SI Centiles[%] Segment 5	centile	56	0.142857	-0.02439
Inter-operator	RV 24-Segment SI Centiles[%] Segment 16	centile	56	0.142857	0.121569
Inter-operator	RV 24-Segment SI Centiles[%] Segment 18	centile	56	0.142857	0.121569
Inter-operator	RV 24-Segment SI Centiles[%] Segment 22	centile	56	0.142857	0
Inter-operator	RV 24-Segment SI Centiles[%] Segment 23	centile	56	0.142857	0
Inter-operator	RV 24-Segment SI Centiles[%] Segment 24	centile	56	0.142857	0
Inter-operator	RV_ ED Area.Rt ED Area % (AC)[%]	centile	56	0.142857	0.255814
Intra-operator	LV_ Frac. Area change.Lt FRAC % (FL)[%]	centile	29	0.137931	0.57971
Intra-operator	LV_ CO.Lt CO % (AUA)[%]	centile	30	0.133333	0.52
Intra-operator	LV_ Frac. Area change.Lt FRAC % (HC)[%]	centile	30	0.133333	0.62963
Intra-operator	LV_ Global Strain.Lt GLS %[%]	centile	30	0.133333	0.268293
Intra-operator	RV 24-Segment SI Centiles[%] Segment 16	centile	30	0.133333	-0.046512
Intra-operator	RV 24-Segment SI Centiles[%] Segment 17	centile	30	0.133333	-0.046512
Intra-operator	RV 24-Segment SI Centiles[%] Segment 19	centile	30	0.133333	-0.052632
Inter-operator	LV_ ED Area.Lt ED Area % (AUA)[%]	centile	54	0.12963	0.547486
Inter-operator	LV_ ED Area.Lt ED Area % (HC)[%]	centile	55	0.127273	0.463415
Inter-operator	LV_ ED Area.Lt ED Area % (AC)[%]	centile	56	0.125	0.34375
Inter-operator	RV 24-Segment SI Centiles[%] Segment 17	centile	56	0.125	0.192308
Intra-operator	LV_ SV.Lt SV % (EFW)[%]	centile	16	0.125	0.75
Intra-operator	LV_ SV/KG.Lt SV/KG % (EFW)[%]	centile	16	0.125	0.709091
Inter-operator	LV_ ED Area.Lt ED Area % (BPD)[%]	centile	55	0.109091	0.542936
Inter-operator	LV_ ED Area.Lt ED Area % (EFW)[%]	centile	55	0.109091	0.372624

group	parameter	scale	usable_n	max_flip_rate	min_kappa
Intra-operator	LV_ SV.Lt SV % (GA)[%]	centile	28	0.107143	0.746988
Intra-operator	LV_ CO.Lt CO % (AC)[%]	centile	19	0.105263	0.728571
Intra-operator	LV_ EF.Lt EF % (AC)[%]	centile	19	0.105263	0.688525
Intra-operator	LV_ SV.Lt SV % (AC)[%]	centile	19	0.105263	0.75641
Intra-operator	LV_ SV/KG.Lt SV/KG % (AC)[%]	centile	19	0.105263	0.728571
Intra-operator	LV_ EF.Lt EF % (HC)[%]	centile	13	0.076923	0.80597
Intra-operator	LV_ EF.Lt EF % (EFW)[%]	centile	16	0.0625	0.818182
Inter-operator	LV_ MAPSE septal.MAPSE sept. % (AUA)[%]	centile	54	0.037037	-0.018868
Intra-operator	4CV Length ED.4CV L ED % (GA)[%]	centile	28	0.035714	0.9
Intra-operator	4CV Trv. Width ED.4CV TW ED % (GA)[%]	centile	28	0.035714	0.78125
Intra-operator	4CV Area (2Dist).4CV A % (FL)[%]	centile	29	0.034483	0.650602
Intra-operator	4CV Circ (2Dist).4CV C % (FL)[%]	centile	29	0.034483	0
Intra-operator	4CV Length ED.4CV L ED % (FL)[%]	centile	29	0.034483	0.868778
Intra-operator	4CV Trv. Width ED.4CV TW ED % (FL)[%]	centile	29	0.034483	0.781955
Intra-operator	4CV Area (2Dist).4CV A % (AUA)[%]	centile	30	0.033333	0.651163
Intra-operator	4CV Area (2Dist).4CV A % (BPD)[%]	centile	30	0.033333	0.83871
Intra-operator	4CV Area (2Dist).4CV A % (HC)[%]	centile	30	0.033333	0.651163
Intra-operator	4CV Circ (2Dist).4CV C % (AUA)[%]	centile	30	0.033333	0.651163
Intra-operator	4CV Circ (2Dist).4CV C % (BPD)[%]	centile	30	0.033333	0.83871
Intra-operator	4CV Length ED.4CV L ED % (AUA)[%]	centile	30	0.033333	0.83871
Intra-operator	4CV Length ED.4CV L ED % (BPD)[%]	centile	30	0.033333	0.782609
Intra-operator	4CV Length ED.4CV L ED % (EFW)[%]	centile	30	0.033333	0.83871
Intra-operator	4CV Length ED.4CV L ED % (HC)[%]	centile	30	0.033333	0.901961
Intra-operator	4CV Trv. Width ED.4CV TW ED % (AUA)[%]	centile	30	0.033333	0.782609
Intra-operator	4CV Trv. Width ED.4CV TW ED % (BPD)[%]	centile	30	0.033333	0
Intra-operator	4CV Trv. Width ED.4CV TW ED % (EFW)[%]	centile	30	0.033333	0.782609
Intra-operator	4CV Trv. Width ED.4CV TW ED % (HC)[%]	centile	30	0.033333	0.651163
Intra-operator	LV_ MAPSE septal.MAPSE sept. % (AUA)[%]	centile	30	0.033333	0
Intra-operator	LV_ SV.Lt SV % (BPD)[%]	centile	30	0.033333	0.782609
Inter-operator	4CV Length ED.4CV L ED % (AUA)[%]	centile	54	0.018519	0.878924
Inter-operator	4CV Area (2Dist).4CV A % (AC)[%]	centile	56	0	1
Inter-operator	4CV Area (2Dist).4CV A % (AUA)[%]	centile	54	0	1
Inter-operator	4CV Area (2Dist).4CV A % (BPD)[%]	centile	55	0	1
Inter-operator	4CV Area (2Dist).4CV A % (EFW)[%]	centile	55	0	1
Inter-operator	4CV Area (2Dist).4CV A % (FL)[%]	centile	56	0	1
Inter-operator	4CV Area (2Dist).4CV A % (HC)[%]	centile	55	0	1
Inter-operator	4CV Circ (2Dist).4CV C % (AC)[%]	centile	56	0	
Inter-operator	4CV Circ (2Dist).4CV C % (AUA)[%]	centile	54	0	1
Inter-operator	4CV Circ (2Dist).4CV C % (BPD)[%]	centile	55	0	1
Inter-operator	4CV Circ (2Dist).4CV C % (EFW)[%]	centile	55	0	1

group	parameter	scale	usable_n	max_flip_rate	min_kappa
Inter-operator	4CV Circ (2Dist).4CV C % (FL)[%]	centile	56	0	1
Inter-operator	4CV Circ (2Dist).4CV C % (GA)[%]	centile	51	0	1
Inter-operator	4CV Circ (2Dist).4CV C % (HC)[%]	centile	55	0	1
Inter-operator	4CV Length ED.4CV L ED % (AC)[%]	centile	56	0	1
Inter-operator	4CV Length ED.4CV L ED % (BPD)[%]	centile	55	0	1
Inter-operator	4CV Length ED.4CV L ED % (EFW)[%]	centile	55	0	1
Inter-operator	4CV Length ED.4CV L ED % (FL)[%]	centile	56	0	1
Inter-operator	4CV Length ED.4CV L ED % (GA)[%]	centile	51	0	1
Inter-operator	4CV Length ED.4CV L ED % (HC)[%]	centile	55	0	1
Inter-operator	4CV Trv. Width ED.4CV TW ED % (AC)[%]	centile	56	0	1
Inter-operator	4CV Trv. Width ED.4CV TW ED % (AUA)[%]	centile	54	0	1
Inter-operator	4CV Trv. Width ED.4CV TW ED % (BPD)[%]	centile	55	0	1
Inter-operator	4CV Trv. Width ED.4CV TW ED % (EFW)[%]	centile	55	0	1
Inter-operator	4CV Trv. Width ED.4CV TW ED % (FL)[%]	centile	56	0	1
Inter-operator	4CV Trv. Width ED.4CV TW ED % (GA)[%]	centile	51	0	1
Inter-operator	4CV Trv. Width ED.4CV TW ED % (HC)[%]	centile	55	0	1
Intra-operator	4CV Area (2Dist).4CV A % (AC)[%]	centile	30	0	1
Intra-operator	4CV Area (2Dist).4CV A % (EFW)[%]	centile	30	0	1
Intra-operator	4CV Circ (2Dist).4CV C % (AC)[%]	centile	30	0	
Intra-operator	4CV Circ (2Dist).4CV C % (EFW)[%]	centile	30	0	1
Intra-operator	4CV Circ (2Dist).4CV C % (GA)[%]	centile	28	0	1
Intra-operator	4CV Circ (2Dist).4CV C % (HC)[%]	centile	30	0	1
Intra-operator	4CV Length ED.4CV L ED % (AC)[%]	centile	30	0	1
Intra-operator	4CV Trv. Width ED.4CV TW ED % (AC)[%]	centile	30	0	1
Intra-operator	LV_ EF.Lt EF % (FL)[%]	centile	17	0	1
Inter-operator	RV_ Basal-Apical FS.Rt BASE-AP FS ZS[]	zscore	53	0.566038	-0.134094
Inter-operator	LV_ Global Strain.Lt GLS ZS[]	zscore	51	0.54902	-0.144638
Inter-operator	LV_ Sept.Wall Strain.Lt SWS ZS[]	zscore	56	0.482143	0.035714
Inter-operator	LV_ Basal-Apical FS.Lt BASE-AP FS ZS[]	zscore	55	0.454545	0.071573
Intra-operator	RV_ Basal-Apical FS.Rt BASE-AP FS ZS[]	zscore	25	0.44	-0.086957
Inter-operator	RV_ FreeWall Strain.Rt FWS ZS[]	zscore	53	0.415094	-0.039216
Inter-operator	RV_ Global Strain.Rt GLS ZS[]	zscore	53	0.415094	-0.05698
Inter-operator	LV_ FreeWall Strain.Lt FWS ZS[]	zscore	55	0.4	-0.050955
Intra-operator	RV_ Sept.Wall Strain.Rt SWS ZS[]	zscore	29	0.344828	0
Intra-operator	LV_ FreeWall Strain.Lt FWS ZS[]	zscore	27	0.333333	0
Inter-operator	RV_ Sept.Wall Strain.Rt SWS ZS[]	zscore	56	0.321429	-0.044776
Intra-operator	LV_ Sept.Wall Strain.Lt SWS ZS[]	zscore	29	0.310345	-0.048193
Intra-operator	LV_ Basal-Apical FS.Lt BASE-AP FS ZS[]	zscore	28	0.25	0.263158
Intra-operator	LV_ Global Strain.Lt GLS ZS[]	zscore	24	0.25	0.454545
Intra-operator	RV_ FreeWall Strain.Rt FWS ZS[]	zscore	25	0.24	-0.056338

group	parameter	scale	usable_n	max_flip_rate	min_kappa
Intra-operator	RV_ Global Strain.Rt GLS ZS[]	zscore	25	0.24	-0.041667

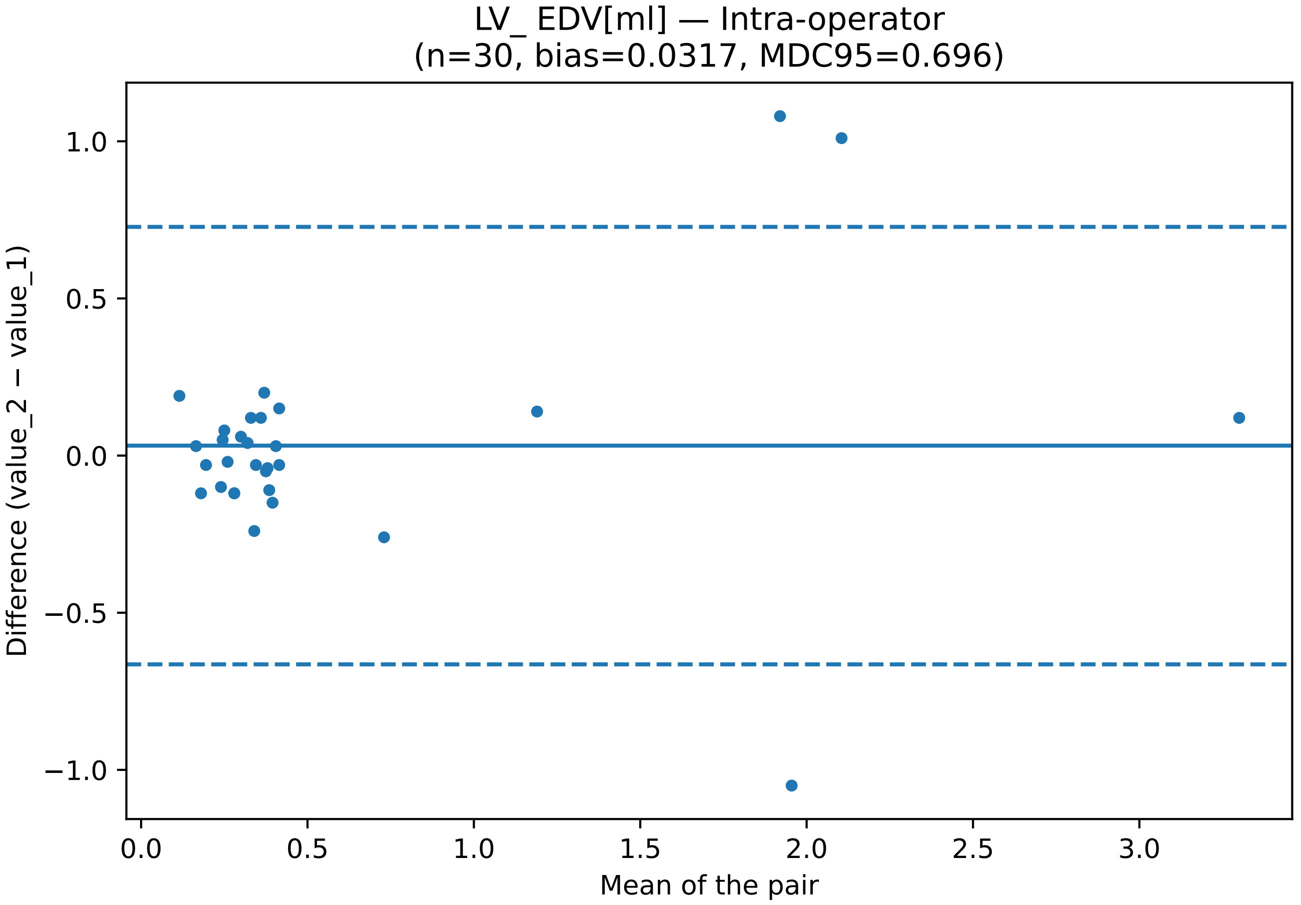
LV_ EDV[ml] — Intra-operator
(n=30, bias=0.0317, MDC95=0.696)
Difference (value_2 − value_1)
Mean of the pair
1.0
0.5
0.0
−0.5
−1.0
0.0
0.5
1.0
1.5
2.0
2.5
3.0

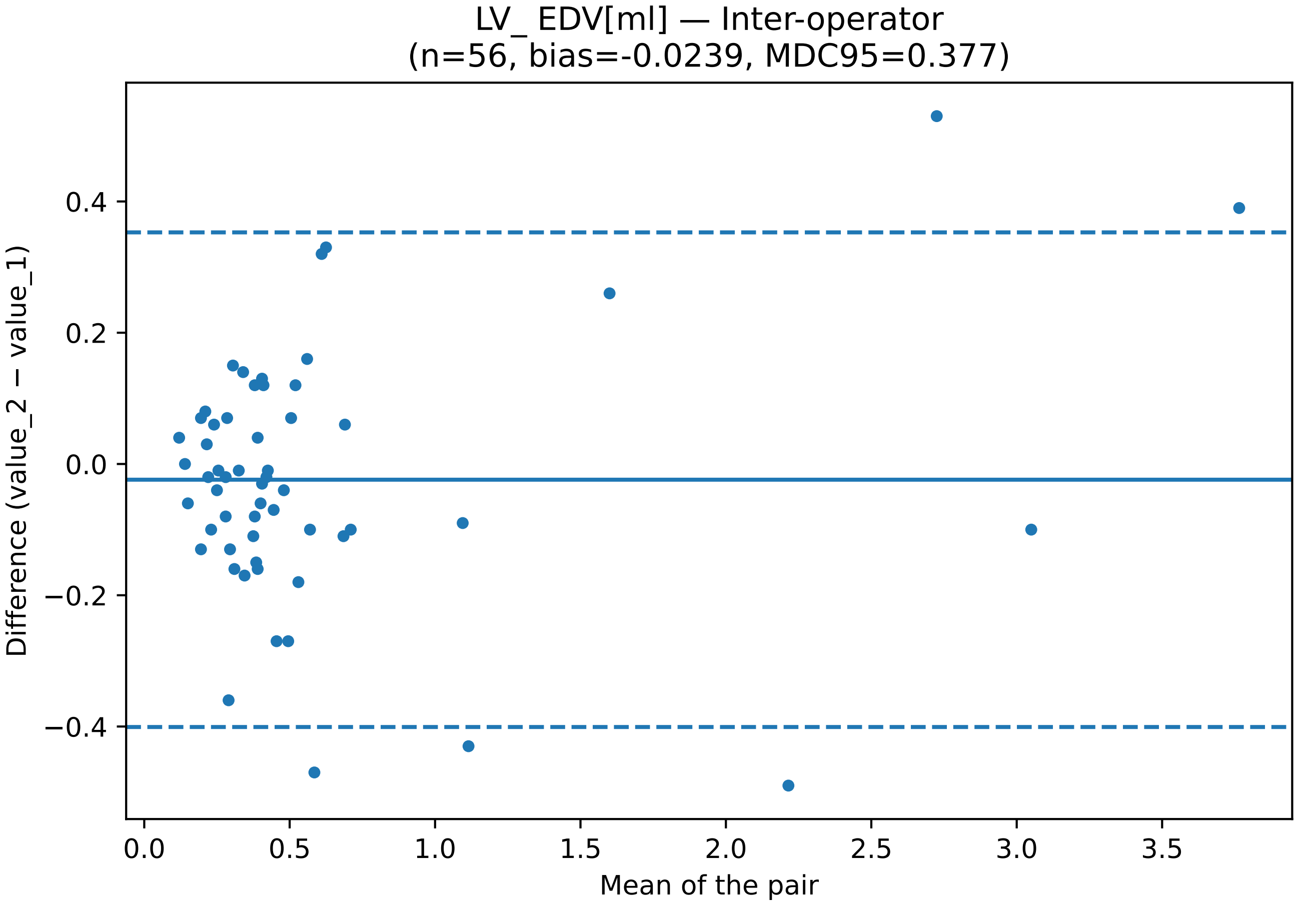

LV_ EDV[ml] — Inter-operator
(n=56, bias=-0.0239, MDC95=0.377)
Difference (value_2 − value_1)
Mean of the pair
0.4
0.2
0.0
−0.2
−0.4
0.0
0.5
1.0
1.5
2.0
2.5
3.0
3.5

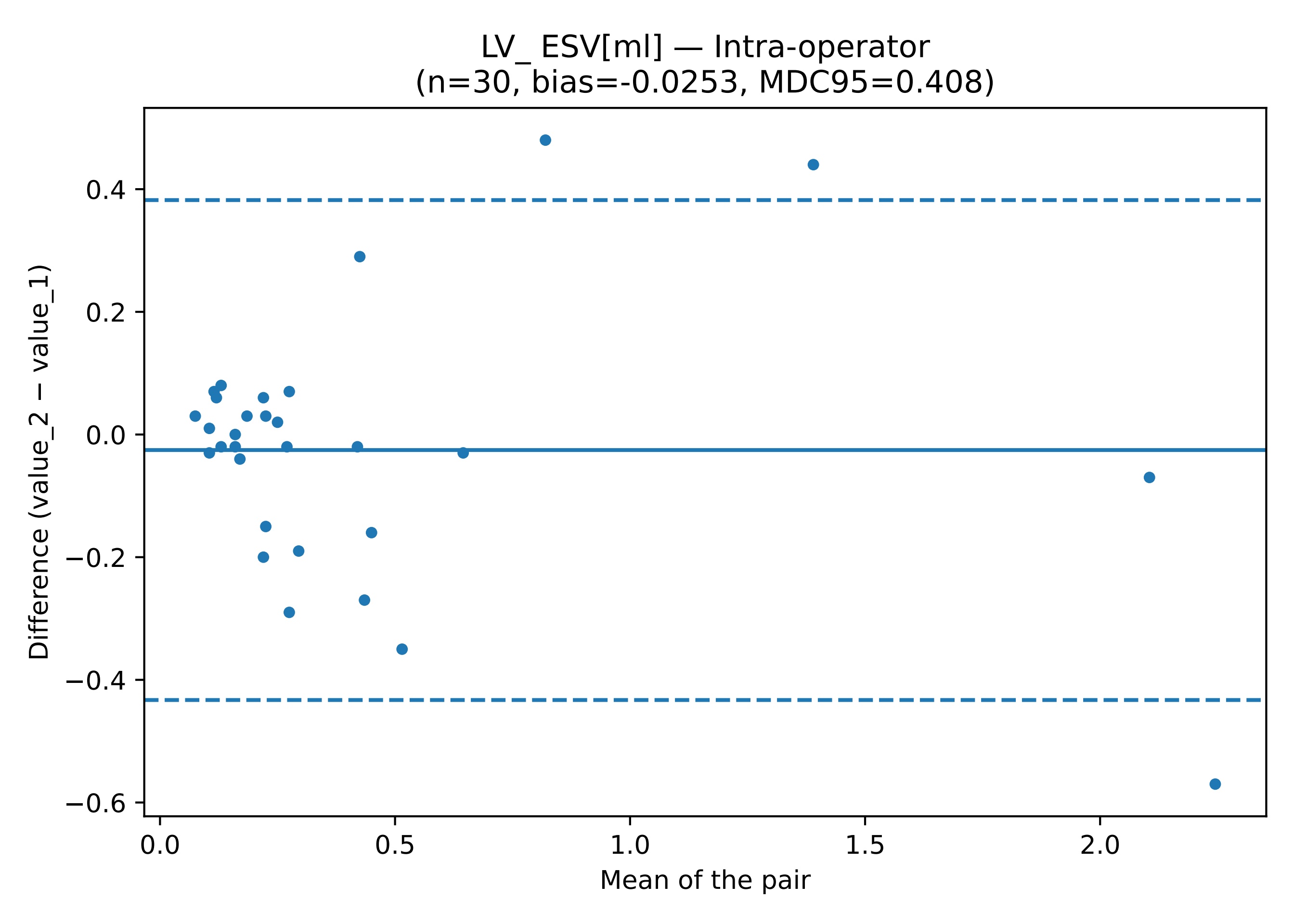

LV_ ESV[ml] — Intra-operator
(n=30, bias=-0.0253, MDC95=0.408)
0.4
0.2
0.0
−0.2
−0.4
−0.6
Difference (value_2 − value_1)
0.0
0.5
1.0
1.5
2.0
Mean of the pair

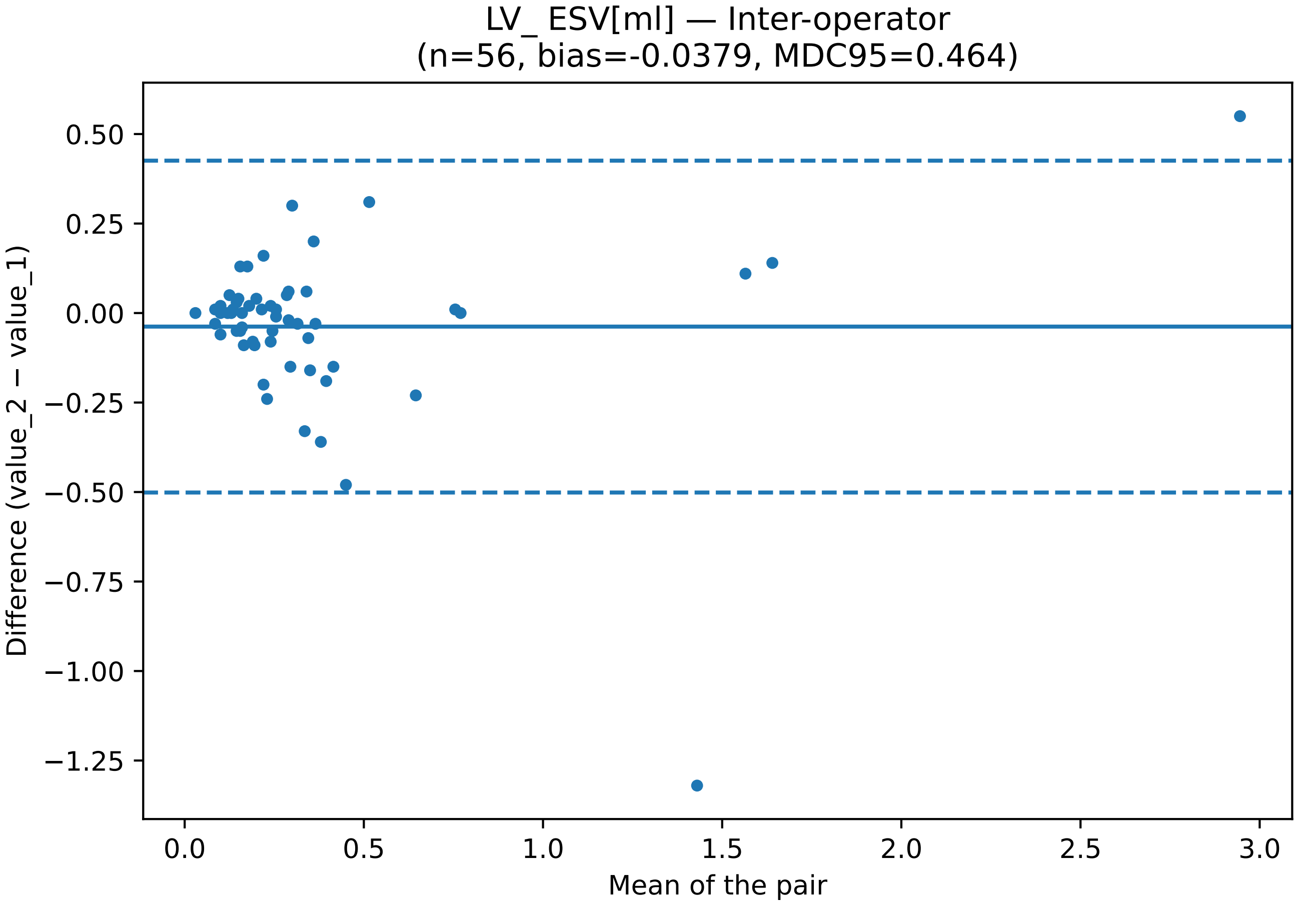

LV_ ESV[ml] — Inter-operator
(n=56, bias=-0.0379, MDC95=0.464)
Difference (value_2 − value_1)
Mean of the pair
0.50
0.25
0.00
−0.25
−0.50
−0.75
−1.00
−1.25
0.0
0.5
1.0
1.5
2.0
2.5
3.0

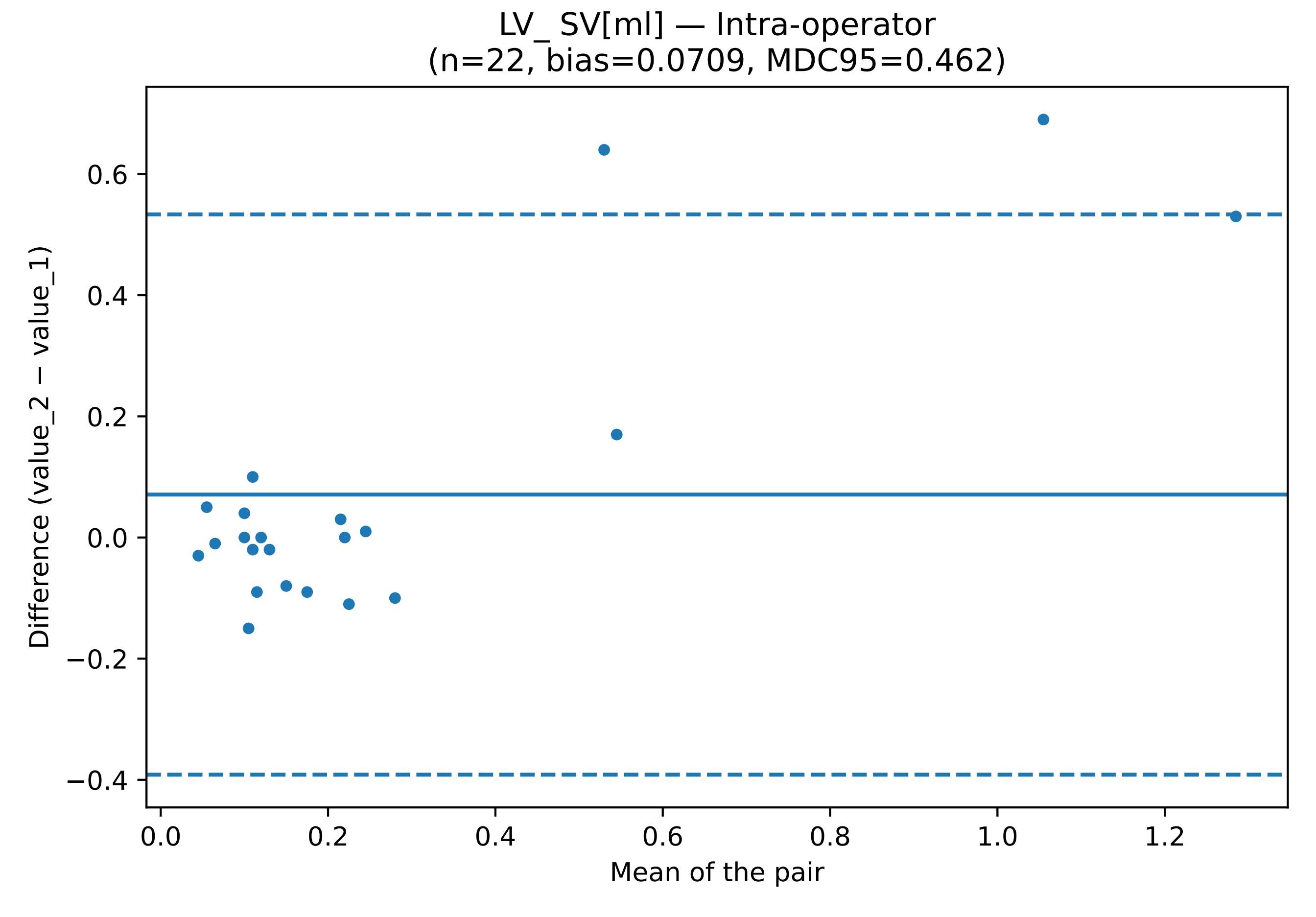

LV_ SV[ml] — Intra-operator
(n=22, bias=0.0709, MDC95=0.462)
Difference (value_2 − value_1)
Mean of the pair
0.6
0.4
0.2
0.0
−0.2
−0.4
0.0
0.2
0.4
0.6
0.8
1.0
1.2

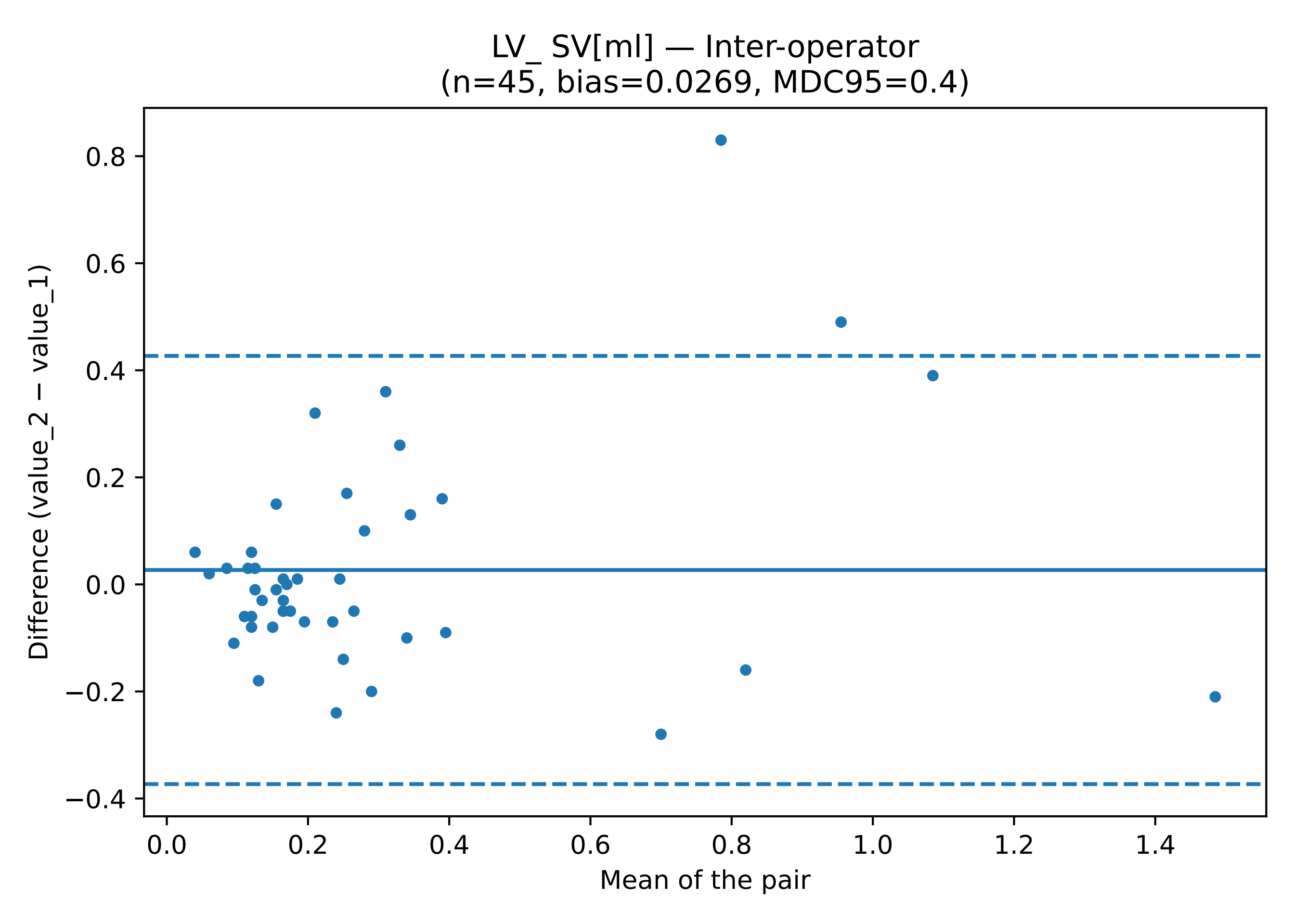
LV_ SV[ml] — Inter-operator
(n=45, bias=0.0269, MDC95=0.4)
Difference (value_2 − value_1)
Mean of the pair
0.8
0.6
0.4
0.2
0.0
−0.2
−0.4
0.0
0.2
0.4
0.6
0.8
1.0
1.2
1.4

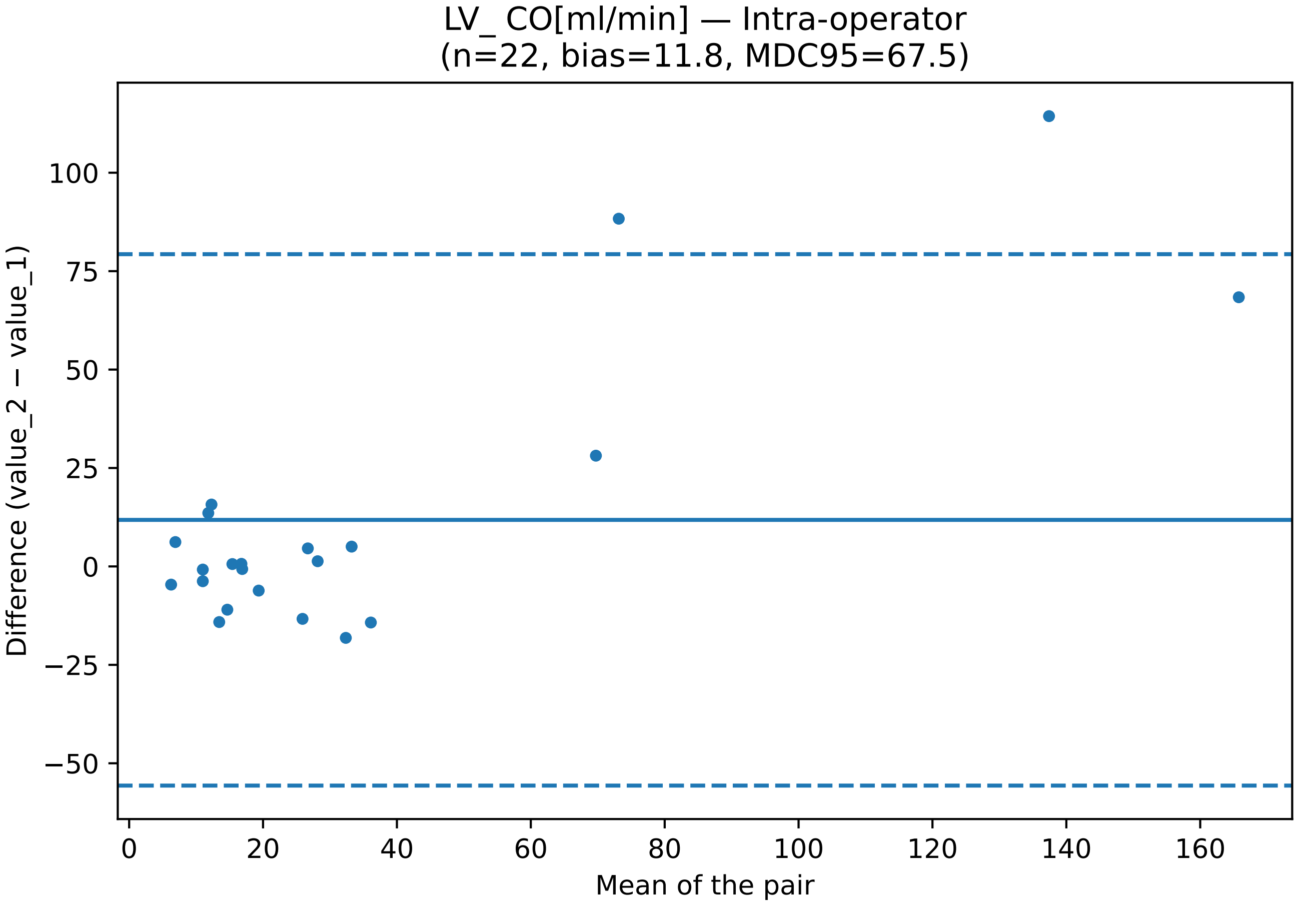
LV_ CO[ml/min] — Intra-operator
(n=22, bias=11.8, MDC95=67.5)
Difference (value_2 − value_1)
Mean of the pair
100
75
50
25
0
−25
−50
0
20
40
60
80
100
120
140
160

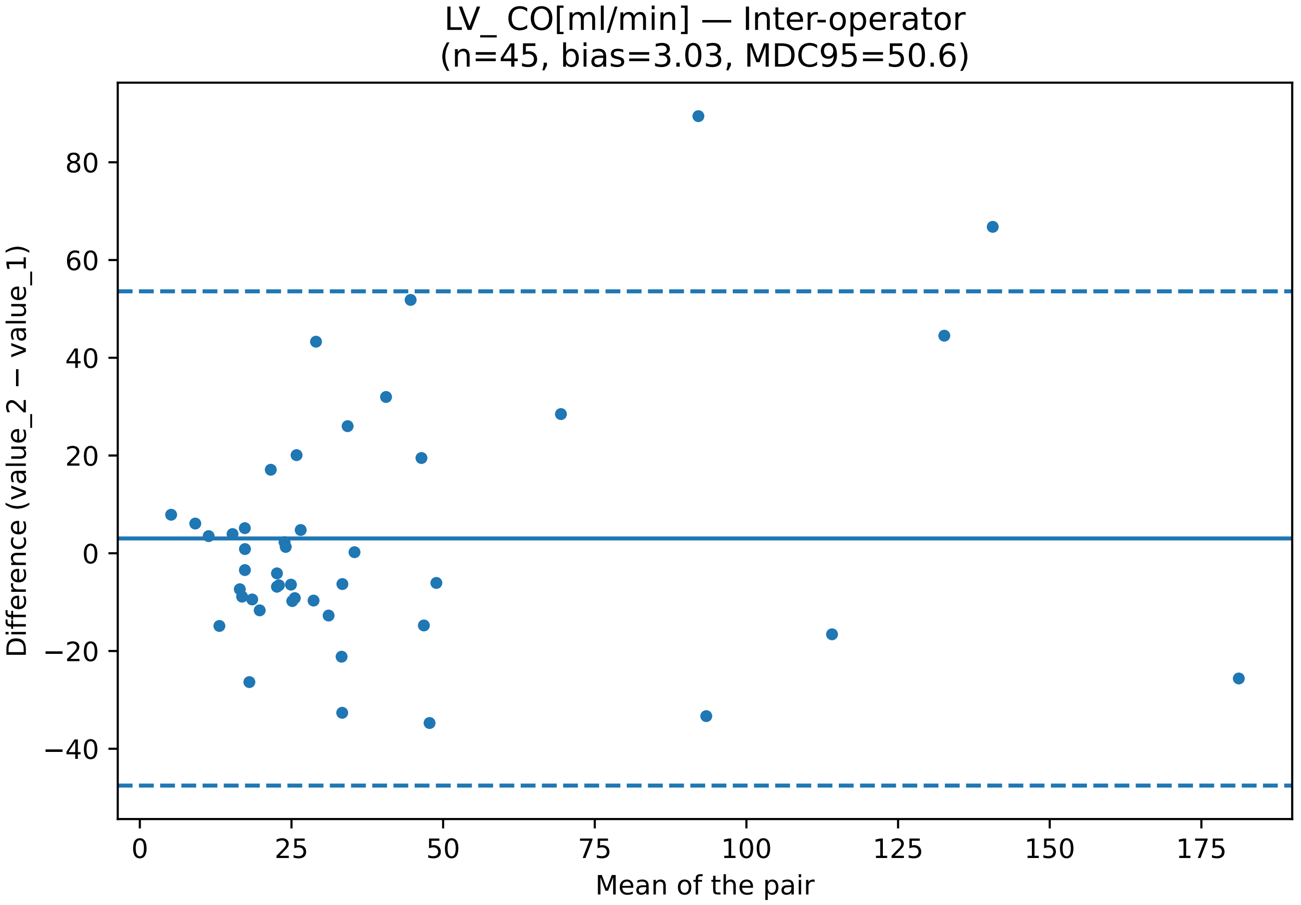

LV_ CO[ml/min] — Inter-operator
(n=45, bias=3.03, MDC95=50.6)
Difference (value_2 − value_1)
Mean of the pair
80
60
40
20
0
−20
−40
0
25
50
75
100
125
150
175

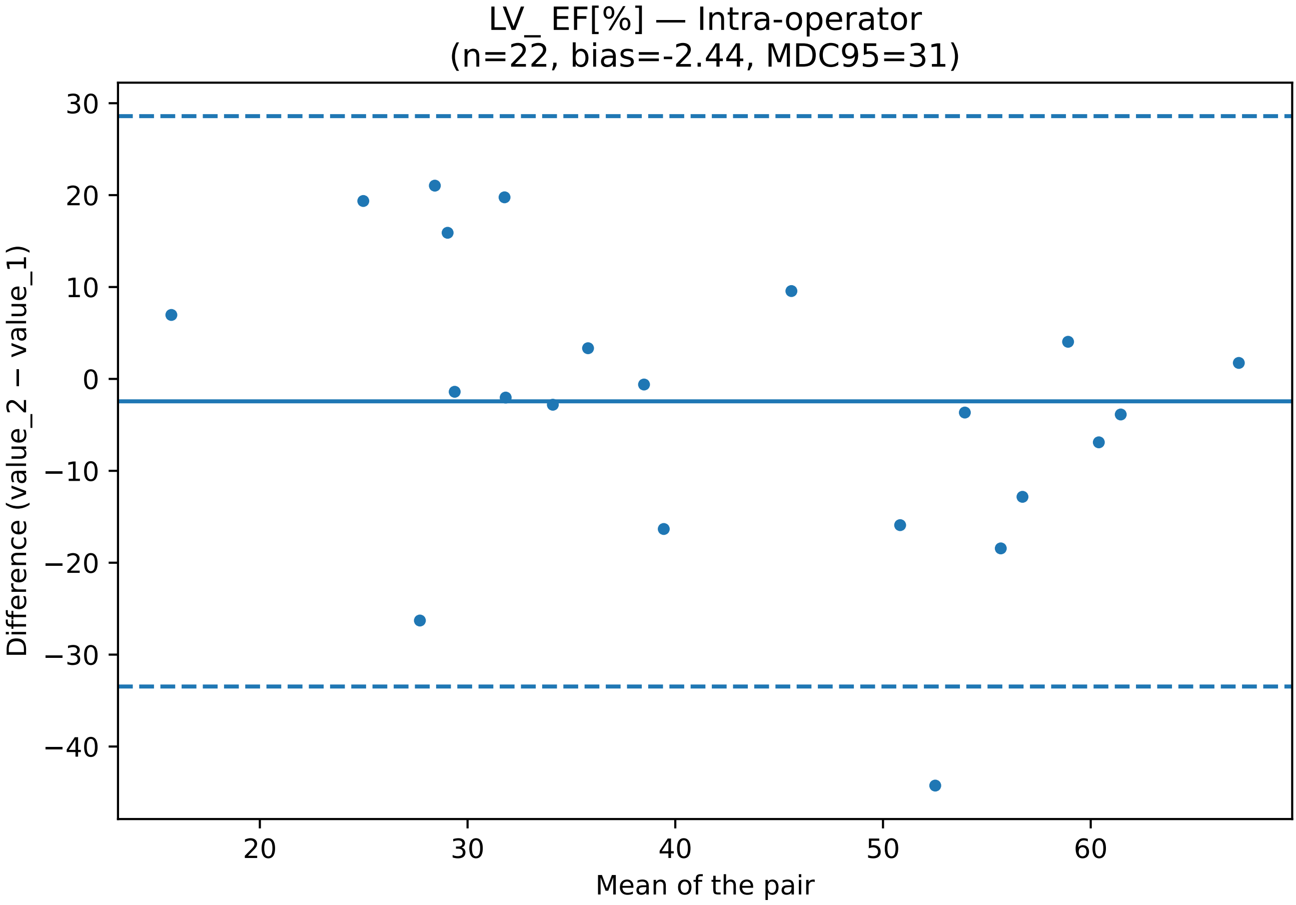

LV_ EF[%] — Intra-operator
(n=22, bias=-2.44, MDC95=31)
Difference (value_2 − value_1)
Mean of the pair
30
20
10
0
−10
−20
−30
−40
20
30
40
50
60

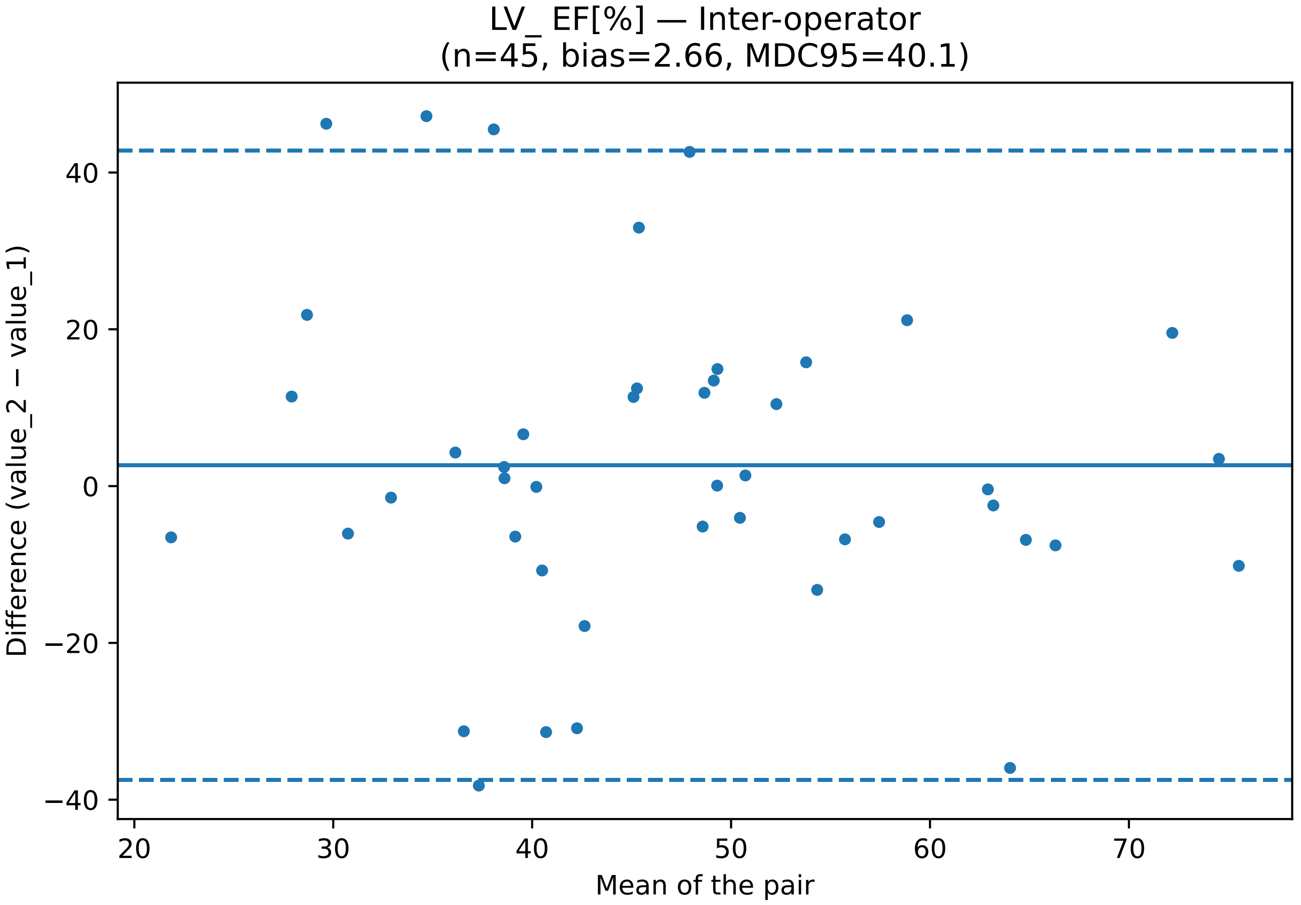

LV_ EF[%] — Inter-operator
(n=45, bias=2.66, MDC95=40.1)
Difference (value_2 − value_1)
40
20
0
−20
−40
20
30
40
50
60
70
Mean of the pair

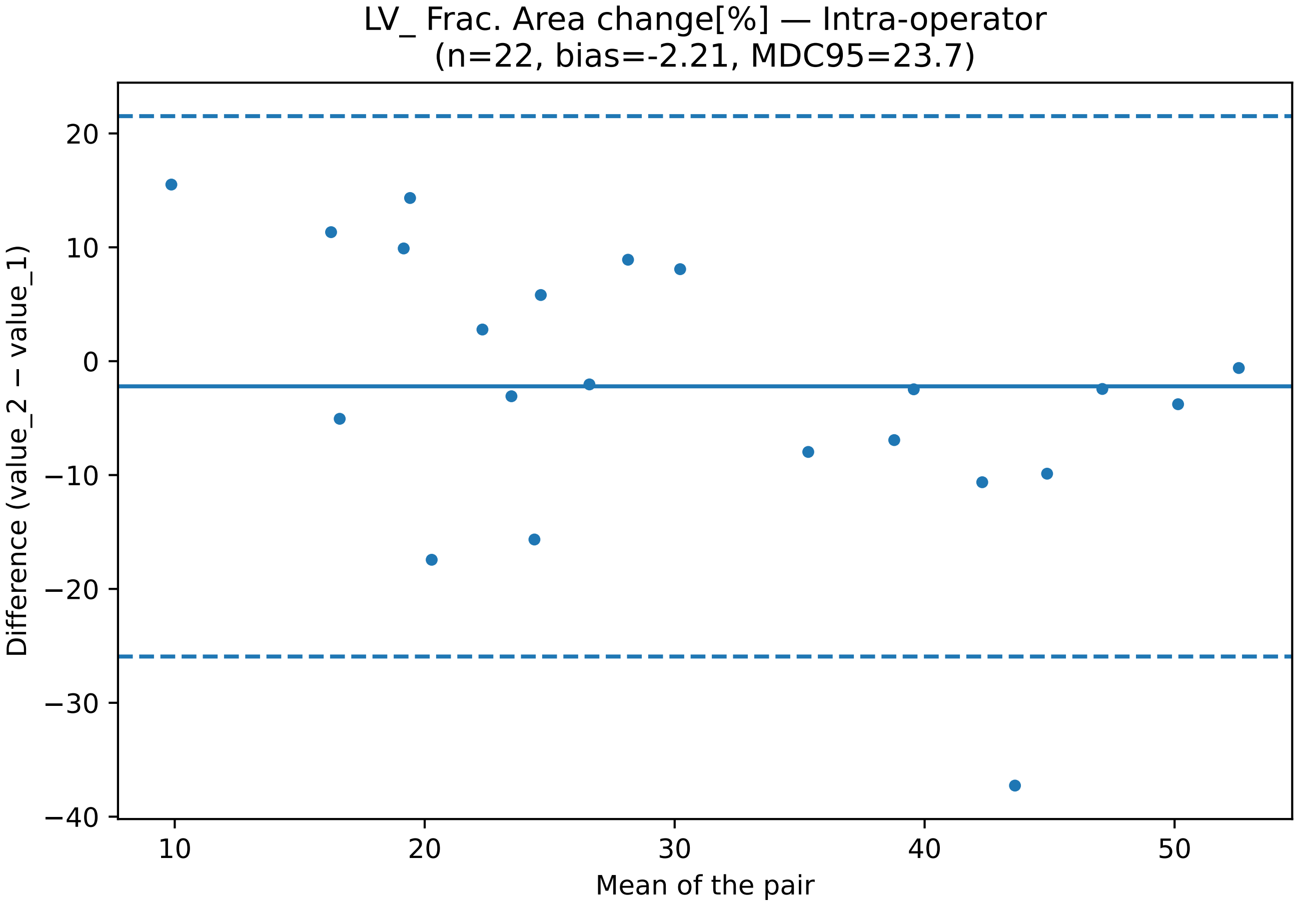

LV_ Frac. Area change[%] — Intra-operator
(n=22, bias=-2.21, MDC95=23.7)
20
10
0
−10
−20
−30
−40
Difference (value_2 − value_1)
10
20
30
40
50
Mean of the pair

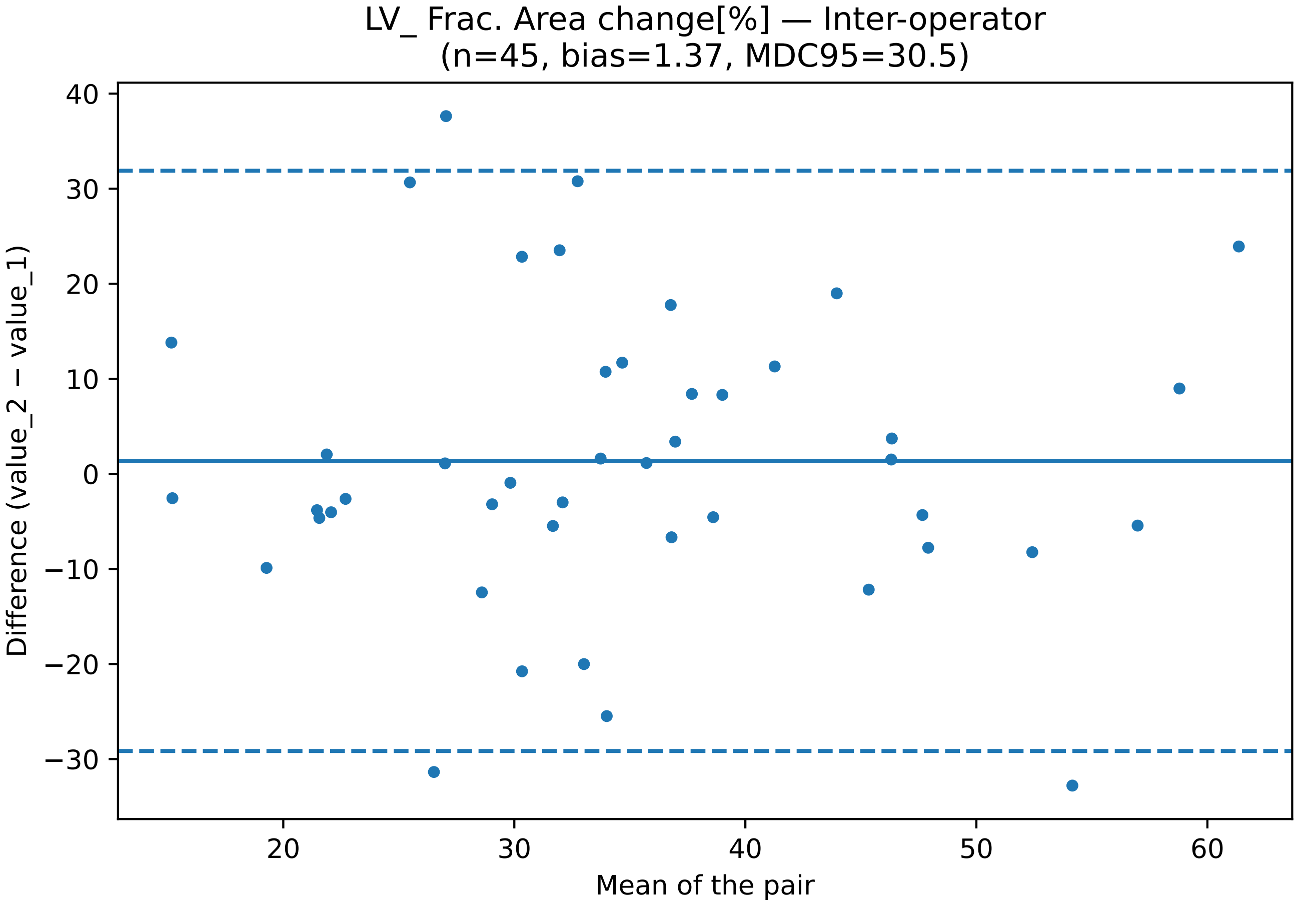
LV_ Frac. Area change[%] — Inter-operator
(n=45, bias=1.37, MDC95=30.5)
Difference (value_2 − value_1)
Mean of the pair
40
30
20
10
0
−10
−20
−30
20
30
40
50
60

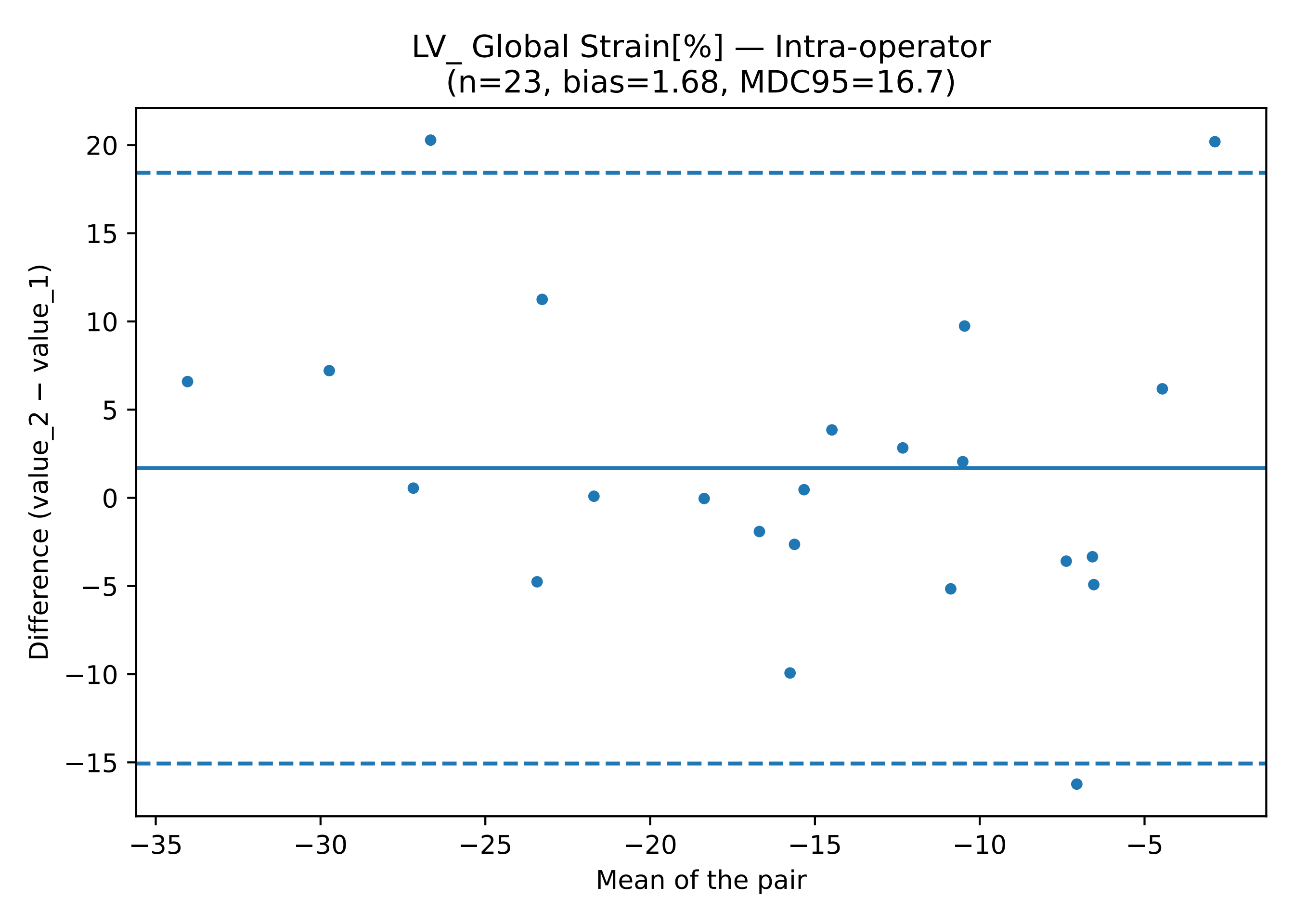

LV_ Global Strain[%] — Intra-operator
(n=23, bias=1.68, MDC95=16.7)
Difference (value_2 − value_1)
Mean of the pair
20
15
10
5
0
−5
−10
−15
−35
−30
−25
−20
−15
−10
−5

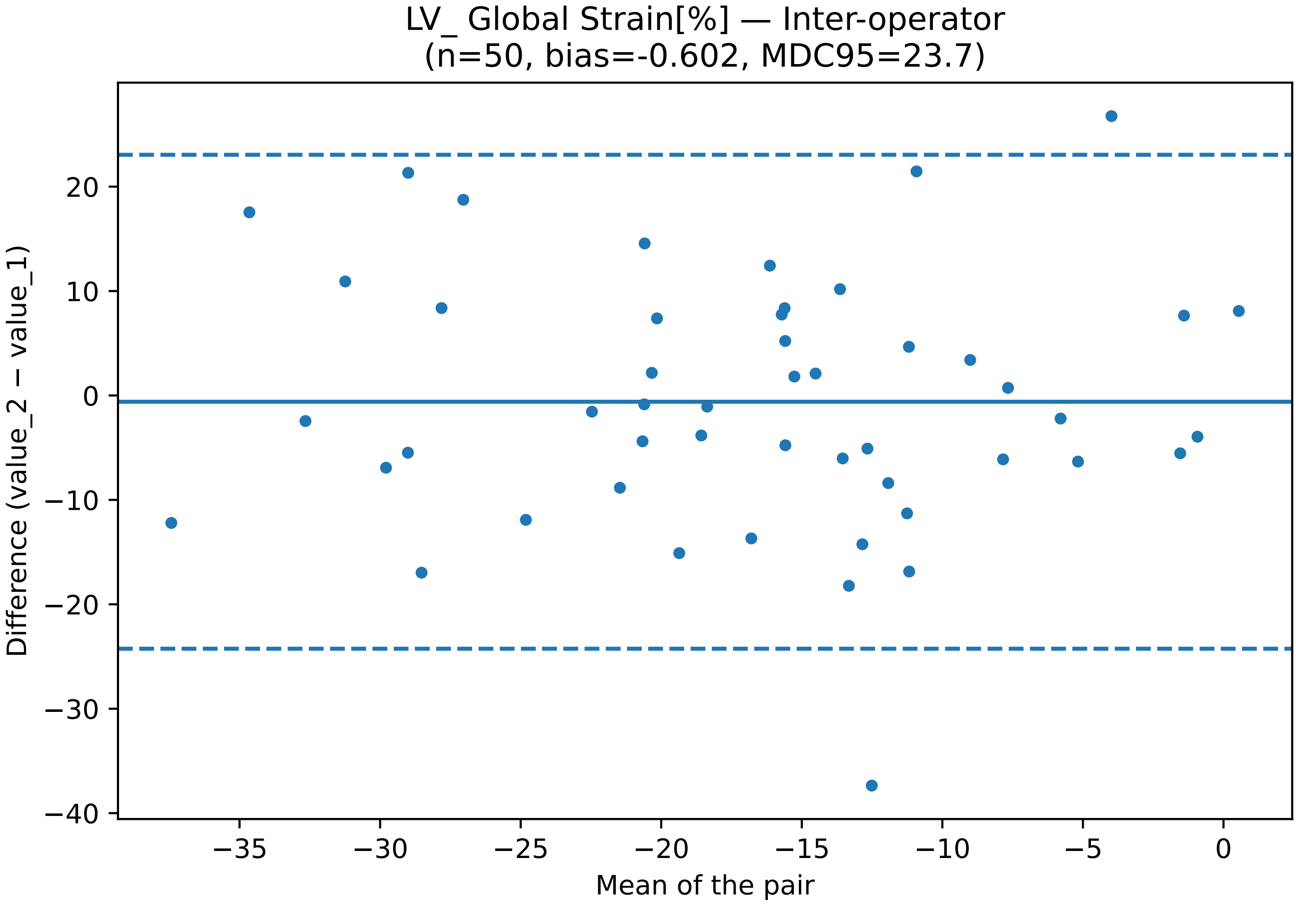

LV_ Global Strain[%] — Inter-operator
(n=50, bias=-0.602, MDC95=23.7)
Difference (value_2 − value_1)
Mean of the pair
20
10
0
−10
−20
−30
−40
−35
−30
−25
−20
−15
−10
−5
0

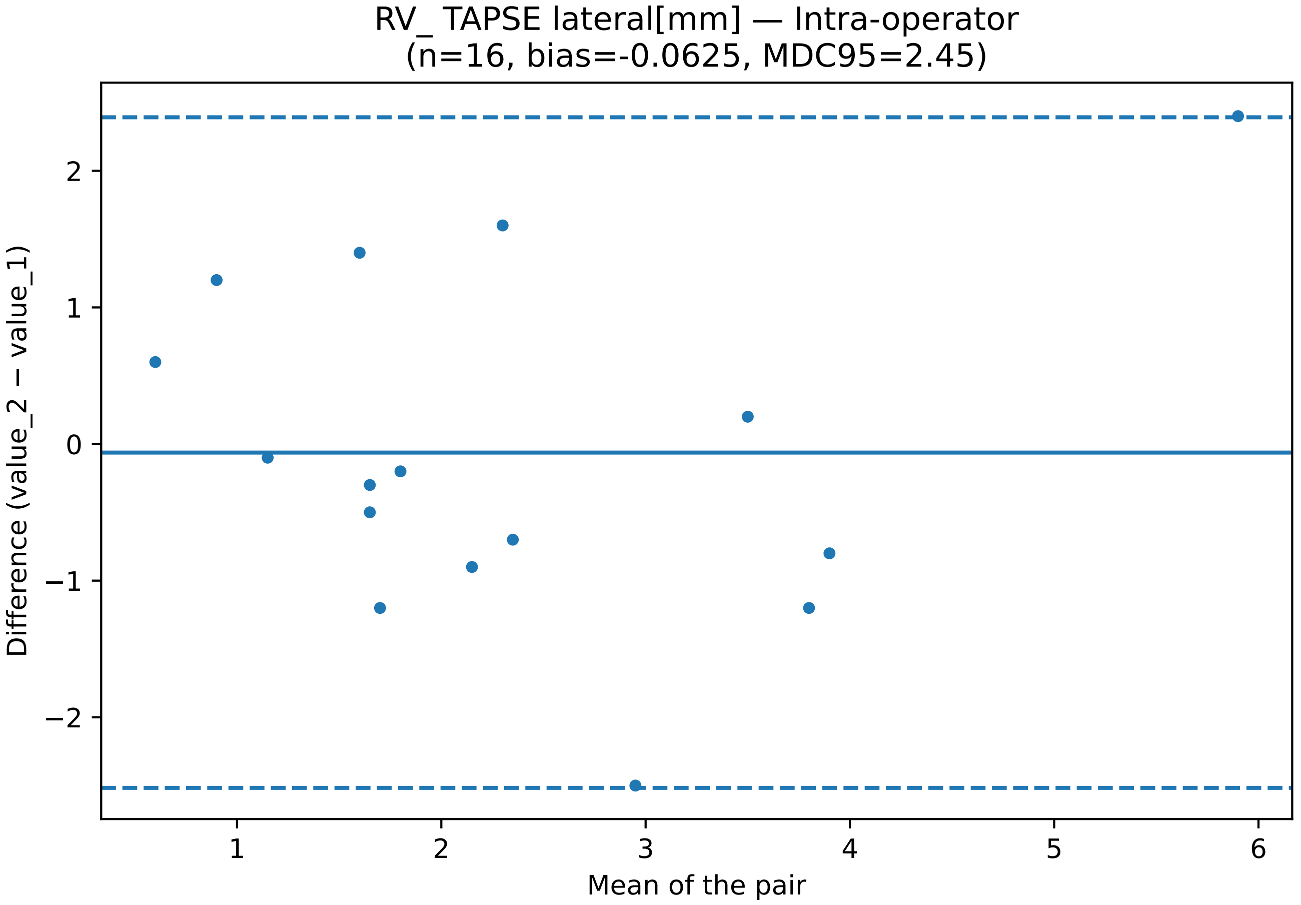

RV_ TAPSE lateral[mm] — Intra-operator
(n=16, bias=-0.0625, MDC95=2.45)
Difference (value_2 − value_1)
Mean of the pair
2
1
0
−1
−2
1
2
3
4
5
6

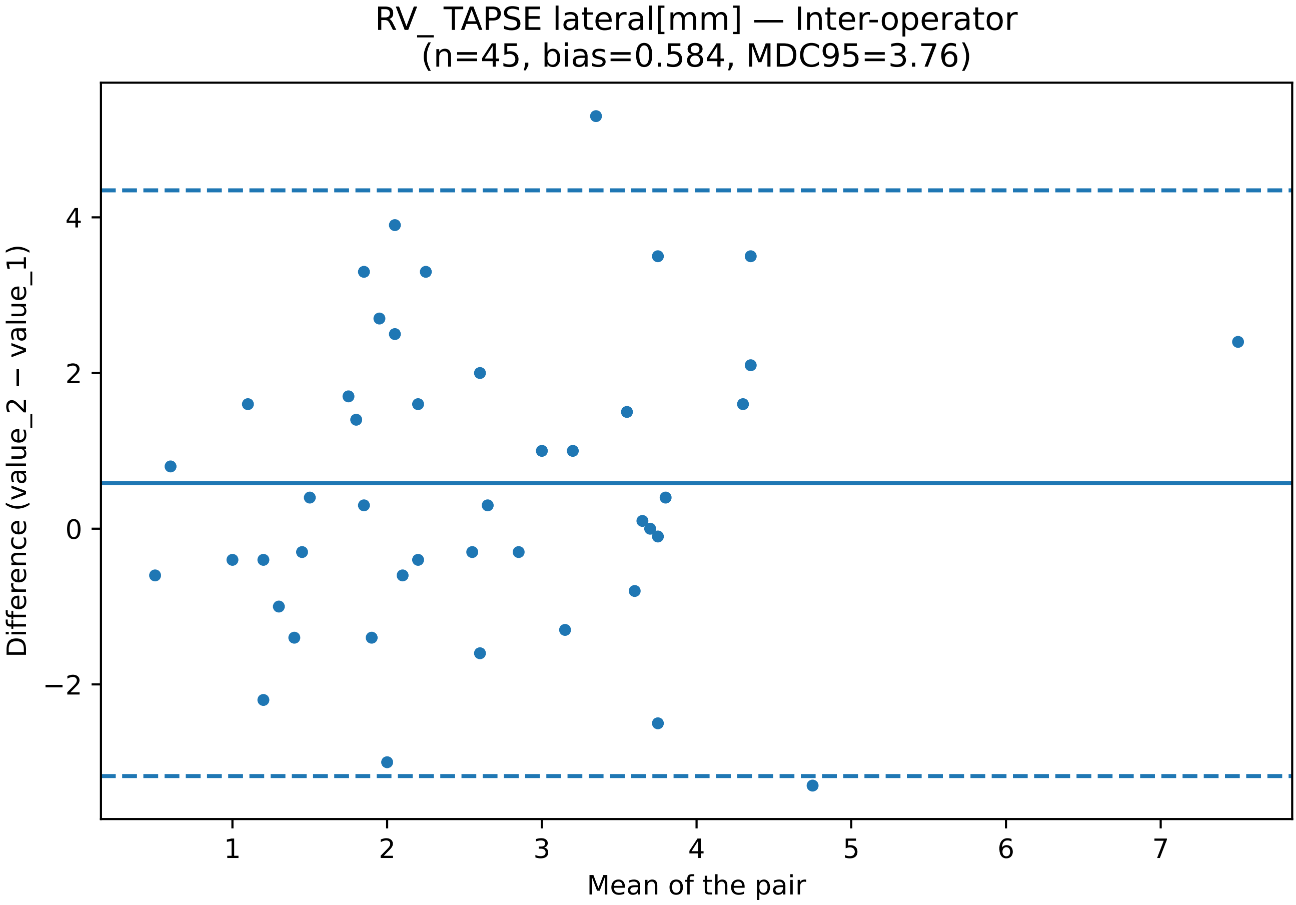

RV_ TAPSE lateral[mm] — Inter-operator
(n=45, bias=0.584, MDC95=3.76)
4
2
0
−2
Difference (value_2 − value_1)
1
2
3
4
5
6
7
Mean of the pair

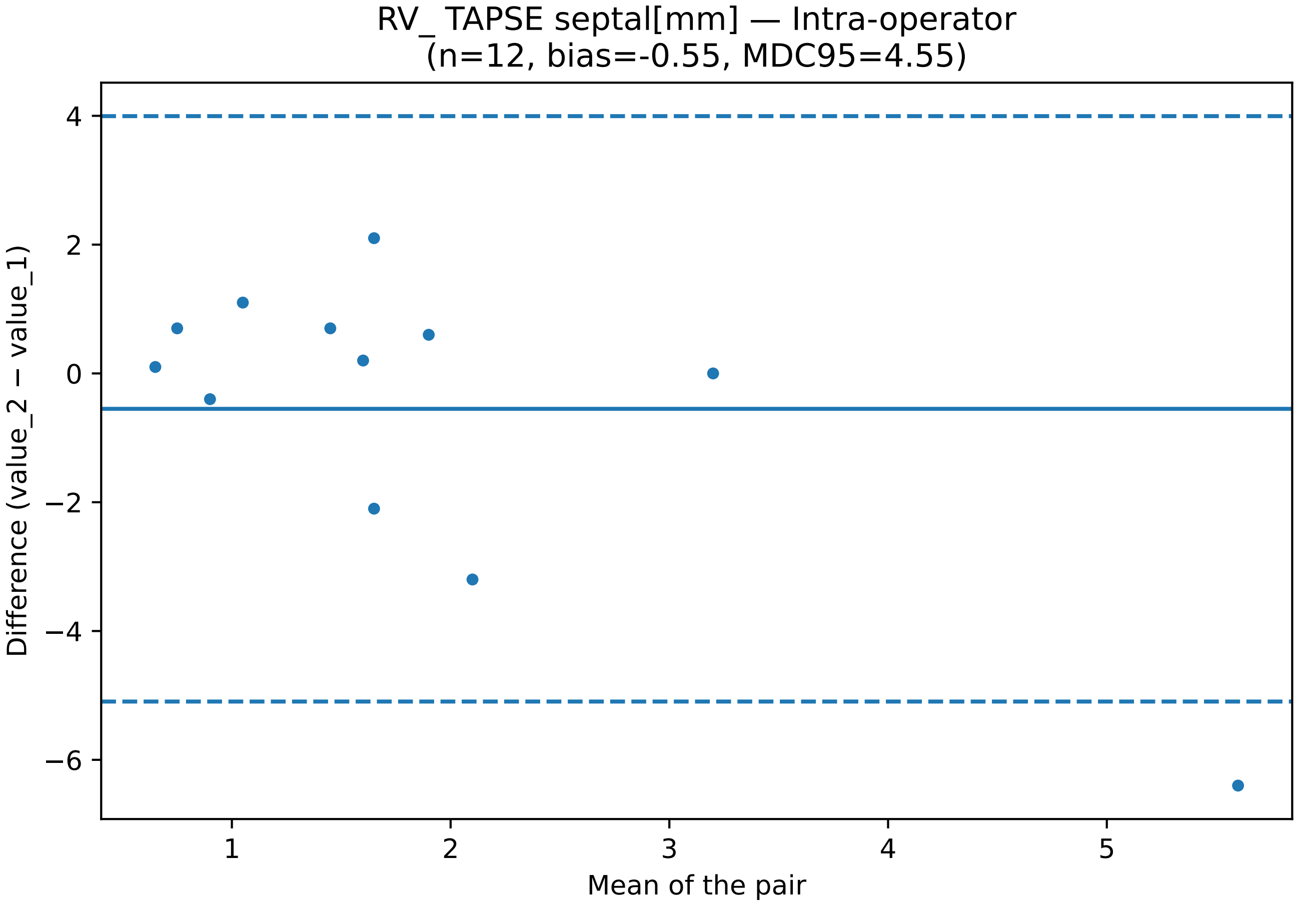
RV_ TAPSE septal[mm] — Intra-operator
(n=12, bias=-0.55, MDC95=4.55)
Difference (value_2 − value_1)
Mean of the pair
4
2
0
−2
−4
−6
1
2
3
4
5

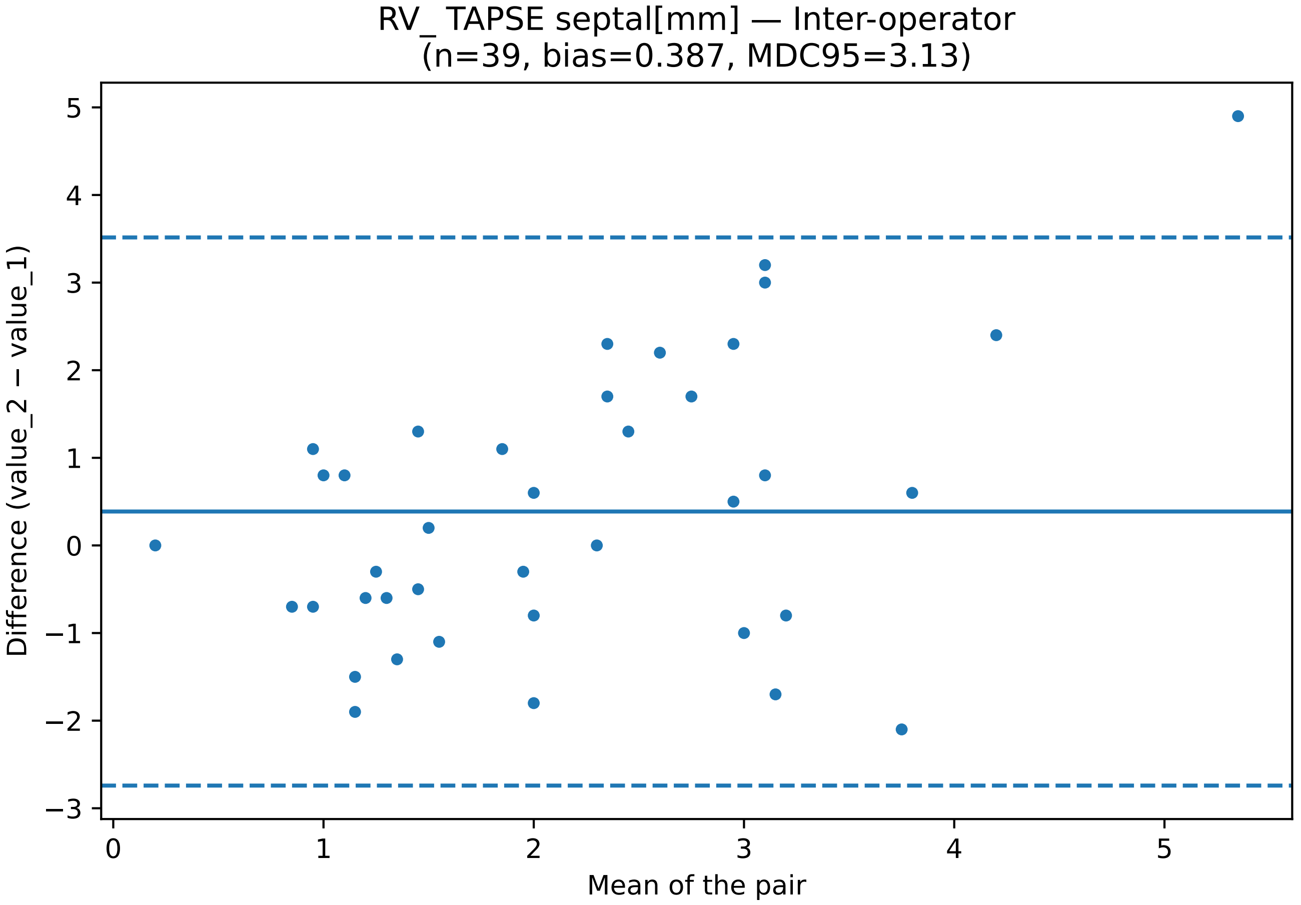
RV_ TAPSE septal[mm] — Inter-operator
(n=39, bias=0.387, MDC95=3.13)
Difference (value_2 − value_1)
Mean of the pair
5
4
3
2
1
0
−1
−2
−3
0
1
2
3
4
5

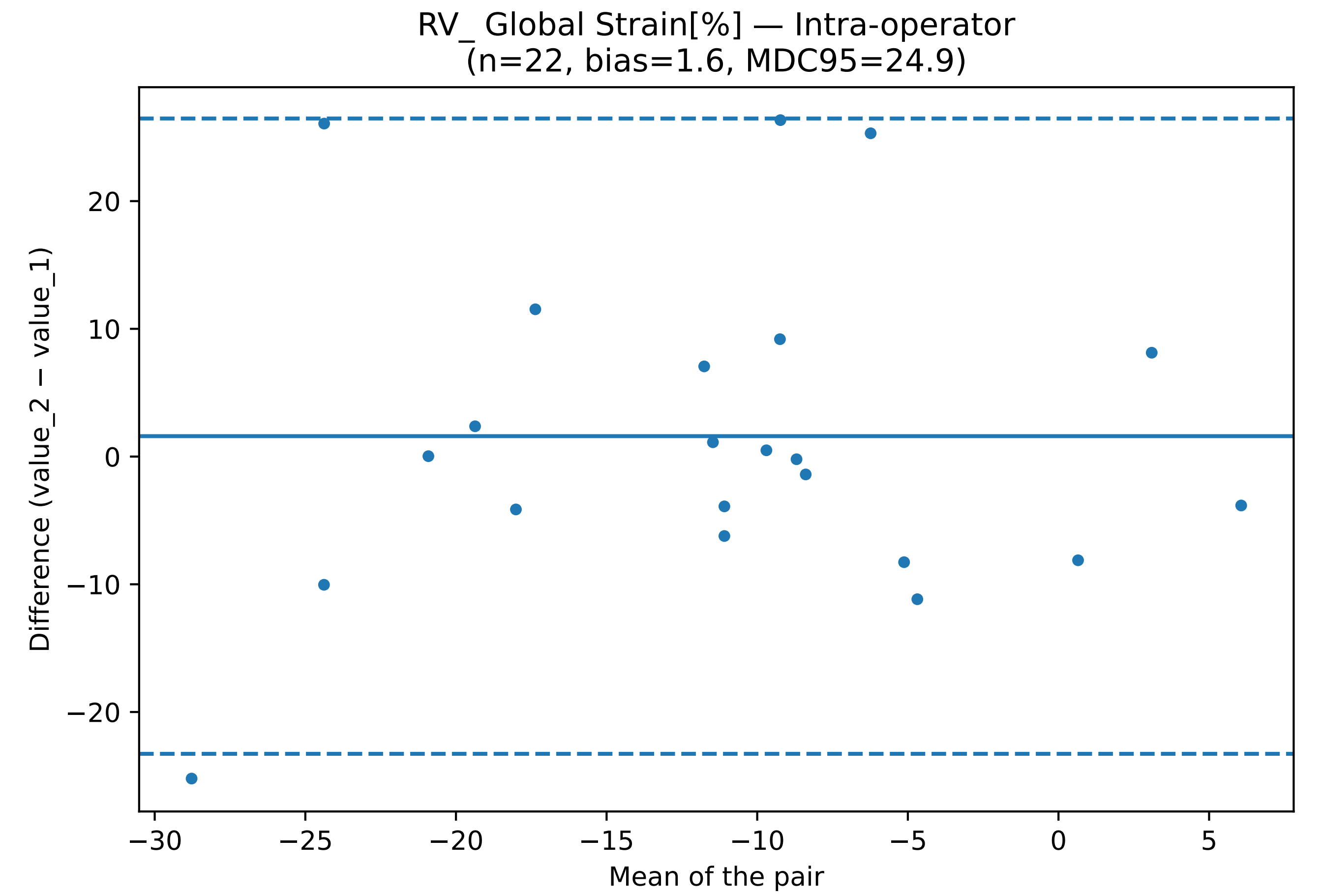
RV_ Global Strain[%] — Intra-operator
(n=22, bias=1.6, MDC95=24.9)
Difference (value_2 − value_1)
Mean of the pair
20
10
0
−10
−20
−30
−25
−20
−15
−10
−5
0
5

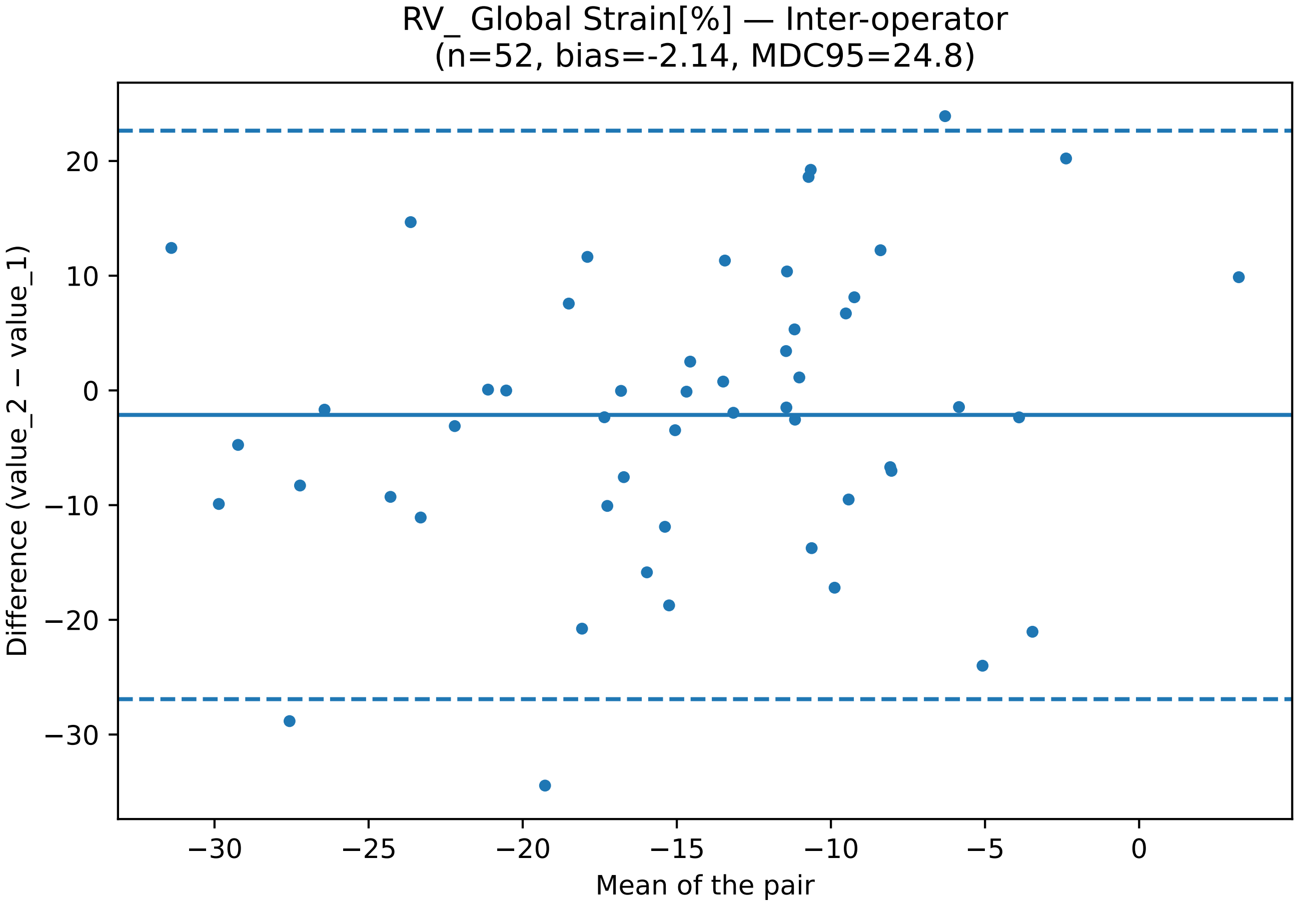
RV_ Global Strain[%] — Inter-operator
(n=52, bias=-2.14, MDC95=24.8)
Difference (value_2 − value_1)
Mean of the pair
20
10
0
−10
−20
−30
−30
−25
−20
−15
−10
−5
0

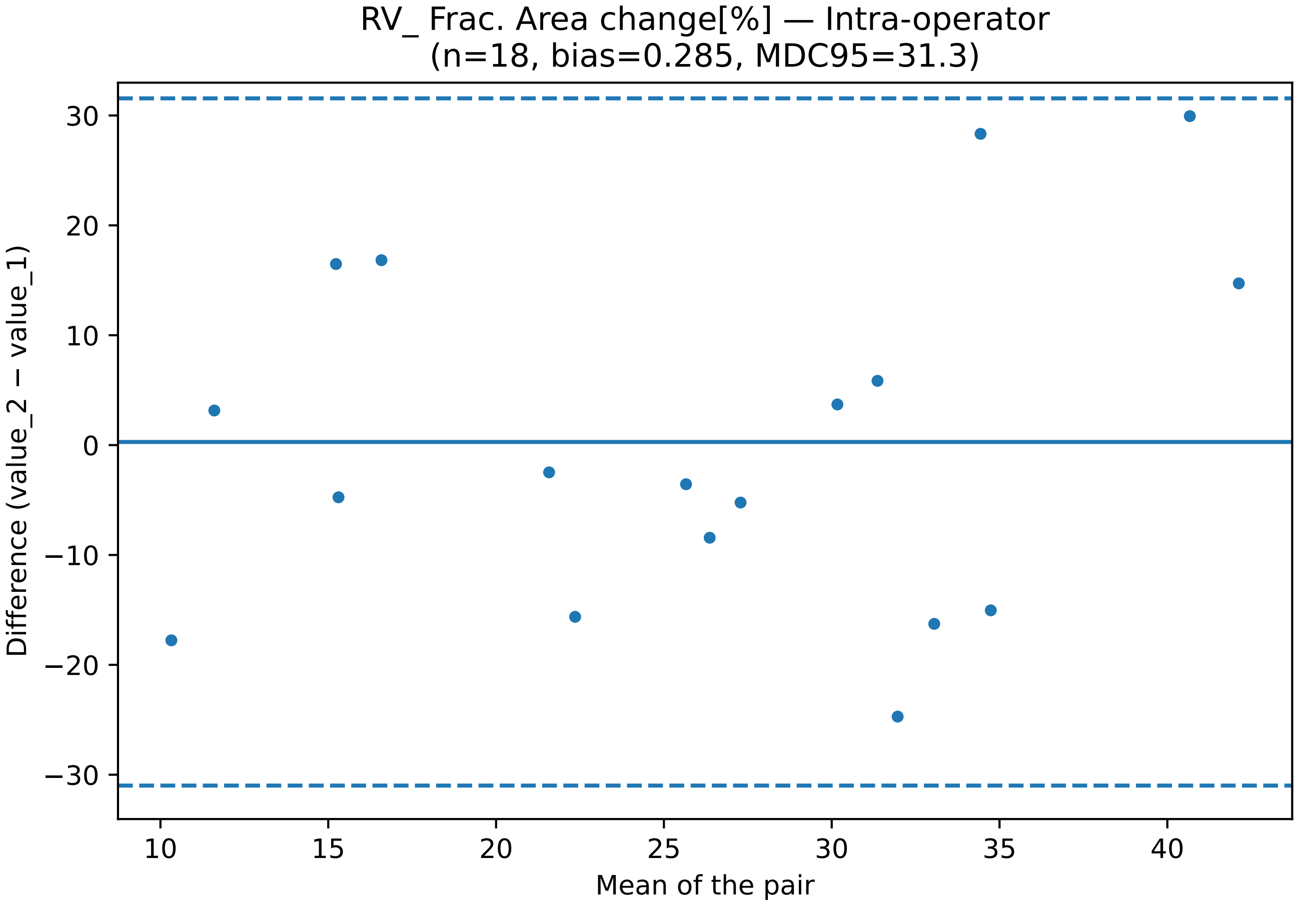

RV_ Frac. Area change[%] — Intra-operator
(n=18, bias=0.285, MDC95=31.3)
Difference (value_2 − value_1)
Mean of the pair
30
20
10
0
−10
−20
−30
10
15
20
25
30
35
40

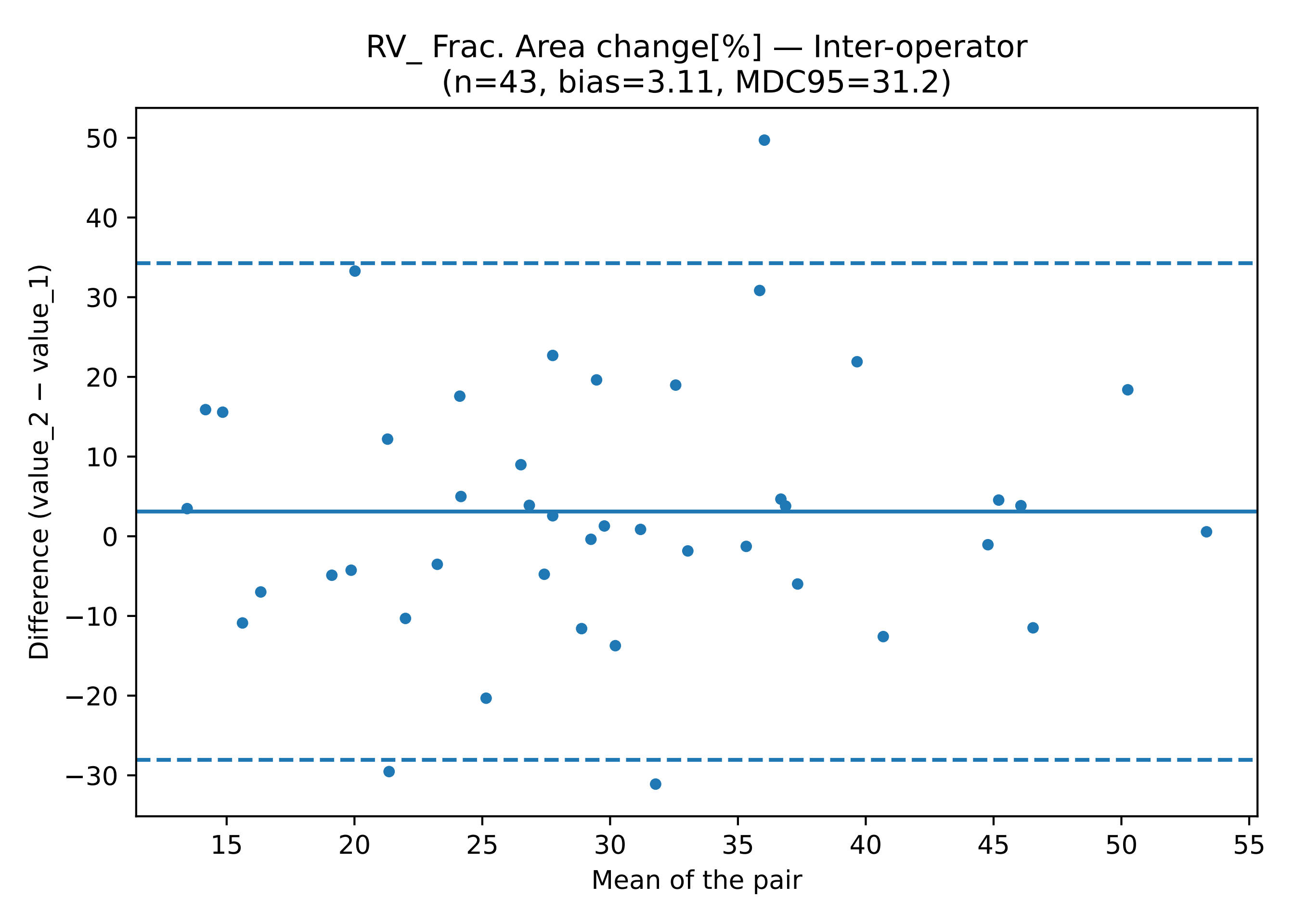

RV_ Frac. Area change[%] — Inter-operator
(n=43, bias=3.11, MDC95=31.2)
Difference (value_2 − value_1)
Mean of the pair
50
40
30
20
10
0
−10
−20
−30
15
20
25
30
35
40
45
50
55

Supplementary File S5

AsIs: S5: Key metrics (as-is: complete pairs only)

metric	group	n_complete	n_usable	bias	sd_diff	loa_low	loa_high	loa_width	mdc95
LV_ EDV[ml]	Intra-operator	30	30	0.031667	0.355082	-0.664294	0.727628	1.391922	0.695961
LV_ EDV[ml]	Inter-operator	56	56	-0.023929	0.192256	-0.400751	0.352894	0.753645	0.376822
LV_ ESV[ml]	Intra-operator	30	30	-0.025333	0.207941	-0.432898	0.382231	0.815129	0.407565
LV_ ESV[ml]	Inter-operator	56	56	-0.037857	0.23648	-0.501357	0.425643	0.927	0.4635
LV_ SV[ml]	Intra-operator	30	30	0.057	0.319646	-0.569506	0.683506	1.253012	0.626506
LV_ SV[ml]	Inter-operator	56	56	0.013929	0.223018	-0.423187	0.451044	0.87423	0.437115
LV_ CO[ml/min]	Intra-operator	30	30	3.901667	65.871076	-125.205643	133.008976	258.214619	129.107309
LV_ CO[ml/min]	Inter-operator	56	56	0.976786	29.234277	-56.322397	58.275969	114.598366	57.299183
LV_ EF[%]	Intra-operator	30	30	45.79	248.346745	-440.96962	532.54962	973.519241	486.75962
LV_ EF[%]	Inter-operator	56	56	1.004107	39.600339	-76.612557	78.620771	155.233328	77.616664
LV_ Frac. Area change[%]	Intra-operator	30	30	16.157667	89.312354	-158.894547	191.20988	350.104427	175.052214
LV_ Frac. Area change[%]	Inter-operator	56	56	-0.365893	27.317158	-53.907522	53.175737	107.083259	53.54163
LV_ Global Strain[%]	Intra-operator	30	30	-4.254667	23.479871	-50.275214	41.765881	92.041094	46.020547
LV_ Global Strain[%]	Inter-operator	56	56	1.192857	13.813485	-25.881573	28.267288	54.148861	27.07443
RV_ TAPSE lateral[mm]	Intra-operator	30	30	-0.06	2.85604	-5.657839	5.537839	11.195678	5.597839
RV_ TAPSE lateral[mm]	Inter-operator	56	56	0.378571	2.049099	-3.637662	4.394805	8.032467	4.016233
RV_ TAPSE septal[mm]	Intra-operator	30	30	0.16	3.538712	-6.775876	7.095876	13.871751	6.935876
RV_ TAPSE septal[mm]	Inter-operator	55	55	0.261818	1.916255	-3.494042	4.017678	7.51172	3.75586

Supplementary File S5

QC_filtered: S5: Key metrics (QC-filtered)

metric	group	n_complete	n_usable	bias	sd_diff	loa_low	loa_high	loa_width	mdc95
LV_ EDV[ml]	Intra-operator	30	30	0.031667	0.355082	-0.664294	0.727628	1.391922	0.695961
LV_ EDV[ml]	Inter-operator	56	56	-0.023929	0.192256	-0.400751	0.352894	0.753645	0.376822
LV_ ESV[ml]	Intra-operator	30	30	-0.025333	0.207941	-0.432898	0.382231	0.815129	0.407565
LV_ ESV[ml]	Inter-operator	56	56	-0.037857	0.23648	-0.501357	0.425643	0.927	0.4635
LV_ SV[ml]	Intra-operator	30	22	0.070909	0.235936	-0.391525	0.533344	0.924869	0.462435
LV_ SV[ml]	Inter-operator	56	45	0.026889	0.20407	-0.373089	0.426866	0.799955	0.399978
LV_ CO[ml/min]	Intra-operator	30	22	11.819545	34.43141	-55.666018	79.305109	134.971127	67.485563
LV_ CO[ml/min]	Inter-operator	56	45	3.031778	25.798591	-47.53346	53.597016	101.130476	50.565238
LV_ EF[%]	Intra-operator	30	22	-2.438182	15.832293	-33.469477	28.593113	62.06259	31.031295
LV_ EF[%]	Inter-operator	56	45	2.663333	20.478679	-37.474878	42.801544	80.276422	40.138211
LV_ Frac. Area change[%]	Intra-operator	30	22	-2.209091	12.105958	-25.936769	21.518587	47.455357	23.727678
LV_ Frac. Area change[%]	Inter-operator	56	45	1.368667	15.572012	-29.152478	31.889811	61.042289	30.521144
LV_ Global Strain[%]	Intra-operator	30	23	1.684783	8.545042	-15.0635	18.433065	33.496565	16.748282
LV_ Global Strain[%]	Inter-operator	56	50	-0.6018	12.066364	-24.251874	23.048274	47.300147	23.650074
RV_ TAPSE lateral[mm]	Intra-operator	30	16	-0.0625	1.252132	-2.516678	2.391678	4.908356	2.454178
RV_ TAPSE lateral[mm]	Inter-operator	56	45	0.584444	1.919512	-3.177799	4.346687	7.524486	3.762243
RV_ TAPSE septal[mm]	Intra-operator	30	12	-0.55	2.318895	-5.095034	3.995034	9.090067	4.545034
RV_ TAPSE septal[mm]	Inter-operator	55	39	0.387179	1.596077	-2.741132	3.515491	6.256623	3.128312

Supplementary File S5

LogScale_Volumes: S5: Log-scale Bland–Altman for positive volumes/flows (ratio LoA)

metric	group	usable_n	bias_log	sd_log	loa_low_log	loa_high_log	ratio_bias	ratio_loa_low	ratio_loa_high	ratio_loa_low_pct	ratio_loa_high_pct
LV_ EDV[ml]	Intra-operator	30	0.045683	0.566363	-1.064389	1.155755	1.046743	0.344939	3.17642	-65.506129	217.641999
LV_ EDV[ml]	Inter-operator	56	-0.082328	0.377883	-0.82298	0.658323	0.92097	0.439121	1.93155	-56.087876	93.155046
LV_ ESV[ml]	Intra-operator	30	-0.055598	0.486495	-1.009129	0.897933	0.945919	0.364536	2.454524	-63.546351	145.45238
LV_ ESV[ml]	Inter-operator	56	-0.10048	0.496567	-1.073751	0.87279	0.904403	0.341724	2.39358	-65.827567	139.358045
LV_ SV[ml]	Intra-operator	22	-0.017141	0.697027	-1.383314	1.349032	0.983005	0.250746	3.853692	-74.925378	285.369212
LV_ SV[ml]	Inter-operator	45	0.036539	0.733993	-1.402088	1.475166	1.037215	0.246083	4.371761	-75.391734	337.176087
LV_ CO[ml/min]	Intra-operator	22	0.106242	0.736591	-1.337476	1.549961	1.112091	0.262507	4.711285	-73.749258	371.128508
LV_ CO[ml/min]	Inter-operator	45	0.031951	0.744475	-1.427219	1.491122	1.032467	0.239975	4.442076	-76.002466	344.207622

Supplementary File S6

Supplementary File S6. Fit-for-purpose ranking.

Score = 0.5*usable_rate + 0.4*(1/(1+relative_MDC95)) + 0.1*(1-max_flip_rate).

Relative_MDC95 = MDC95 / |mean|.

Max_flip_rate is the worst observed flip rate across available thresholds for normalized outputs (centiles/Z-scores); 0 if not applicable.

Inter-operator scores are penalized by 0.3 if a parameter had 100% zero differences in complete pairs (possible copy-forward / non-independent repeat).

Supplementary File S6

All_parameters: All parameters ranked (intra and inter)

parameter	qc_rule	intra_n_usable	intra_usable_rate	intra_rel_mdc95	intra_max_flip_rate	intra_score	inter_n_usable	inter_usable_rate	inter_rel_mdc95	inter_max_flip_rate	inter_score	possible_copy_forward_inter	intra_rank	inter_rank
4CV Area (2Dist).4CV A % (AUA)[%]	centile	30	0.882353	0.303273	0	0.848096	54	0.915254	0.002829	0.033333	0.953166	FALSE	17	1
4CV Circ (2Dist).4CV C % (AUA)[%]	centile	30	0.882353	0.293899	0	0.85032	54	0.915254	0.006128	0.033333	0.951858	FALSE	15	2
4CV Length ED.4CV L ED % (AUA)[%]	centile	30	0.882353	0.292921	0.018519	0.848702	54	0.915254	0.011686	0.033333	0.949674	FALSE	16	3
4CV Trv. Width ED.4CV TW ED % (AUA)[%]	centile	30	0.882353	0.378531	0	0.83134	54	0.915254	0.012515	0.033333	0.94935	FALSE	27	4
LV_ ED Length[cm]	positive	30	0.882353	0.407216	0	0.825426	56	0.949153	0.317558	0	0.878168	FALSE	28	5
LV_ ES Length[cm]	positive	30	0.882353	0.333237	0	0.841198	56	0.949153	0.377065	0	0.865049	FALSE	20	6
RV_ ED Length[cm]	positive	30	0.882353	0.429296	0	0.821034	56	0.949153	0.406536	0	0.858963	FALSE	29	7
RV_ ES Length[cm]	positive	30	0.882353	0.521714	0	0.804038	56	0.949153	0.408071	0	0.858653	FALSE	34	8
LV_ ED Area[cm¸]	none	30	0.882353	0.589294	0	0.792861	56	0.949153	0.427659	0	0.854755	FALSE	38	9
RV_ ED Area[cm¸]	none	30	0.882353	0.781799	0	0.765669	56	0.949153	0.45648	0	0.849211	FALSE	46	10
4CV Trv. Width ED.4CV TW ED ZS(AUA)[]	none	30	0.882353	3.102651	0	0.638674	54	0.915254	0.409973	0	0.84132	FALSE	154	11
4CV Length ED.4CV L ED ZS(AUA)[]	none	30	0.882353	1.114588	0	0.730339	54	0.915254	0.448819	0	0.833714	FALSE	58	12
RV_ ES Area[cm¸]	none	30	0.882353	0.747478	0	0.770078	56	0.949153	0.566861	0	0.829864	FALSE	44	13
LV_ EDV[ml]	positive	30	0.882353	1.125847	0	0.729337	56	0.949153	0.615759	0	0.822138	FALSE	62	14
LV_ MAPSE septal.MAPSE sept. ZS(AUA)[]	none	30	0.882353	0.688301	0	0.778101	54	0.915254	0.529221	0	0.819198	FALSE	43	15
LV_ ES Area[cm¸]	none	30	0.882353	0.524116	0	0.803624	56	0.949153	0.721839	0	0.806886	FALSE	35	16
4CV Area (2Dist).4CV A ZS(AUA)[]	none	30	0.882353	1.122562	0	0.729628	54	0.915254	0.753774	0	0.785707	FALSE	61	17
RV_ ED Area.Rt ED Area % (AC)[%]	centile	30	0.882353	1.366391	0.142857	0.695925	56	0.949153	0.820479	0.2	0.774299	FALSE	75	18
RV_ ED Area.Rt ED Area % (EFW)[%]	centile	30	0.882353	1.671337	0.145455	0.676369	55	0.932203	0.801776	0.166667	0.771438	FALSE	95	19
RV_ Global Strain.Rt GLS %[%]	centile	30	0.882353	0.795571	0.339286	0.730018	56	0.949153	0.865128	0.2	0.769039	FALSE	59	20
RV_ FreeWall Strain.Rt FWS %[%]	centile	30	0.882353	0.760566	0.285714	0.739805	56	0.949153	0.853795	0.266667	0.763683	FALSE	54	21
LV_ FreeWall Strain.Lt FWS %[%]	centile	30	0.882353	0.926004	0.303571	0.718503	56	0.949153	0.899334	0.3	0.755176	FALSE	63	22
LV_ ESV[ml]	positive	30	0.882353	0.929099	0	0.748527	56	0.949153	1.231309	0	0.753843	FALSE	52	23
LV_ Global Strain.Lt GLS %[%]	centile	30	0.882353	0.783748	0.357143	0.729709	56	0.949153	1.098928	0.133333	0.751816	FALSE	60	24
LV_ ED Area.Lt ED Area % (AC)[%]	centile	30	0.882353	1.607381	0.125	0.682087	56	0.949153	1.009411	0.233333	0.750306	FALSE	87	25
LV_ Sept.Wall Strain.Lt SWS %[%]	centile	30	0.882353	0.707239	0.357143	0.739759	56	0.949153	1.148314	0.166667	0.744102	FALSE	55	26
RV_ ED Area.Rt ED Area % (AUA)[%]	centile	30	0.882353	2.11645	0.185185	0.651009	54	0.915254	0.916676	0.266667	0.739655	FALSE	132	27
RV_ ED Area.Rt ED Area % (FL)[%]	centile	29	0.852941	2.232714	0.181818	0.632024	55	0.932203	1.07844	0.206897	0.737864	FALSE	164	28
RV_ Sept.Wall Strain.Rt SWS %[%]	centile	30	0.882353	0.883888	0.214286	0.732075	56	0.949153	1.042182	0.366667	0.733778	FALSE	57	29
RV_ ED Area.Rt ED Area % (BPD)[%]	centile	30	0.882353	2.290414	0.218182	0.640924	55	0.932203	1.09708	0.3	0.726843	FALSE	151	30
LV_ ED Area.Lt ED Area % (FL)[%]	centile	29	0.852941	2.153902	0.145455	0.638752	55	0.932203	1.221435	0.206897	0.725476	FALSE	153	31
LV_ ED Length.Lt ED Length ZS(BPD)[]	none	30	0.882353	1.488056	0	0.701945	55	0.932203	1.523228	0	0.724629	FALSE	72	32
RV_ ED Area.Rt ED Area % (HC)[%]	centile	30	0.882353	2.658978	0.2	0.630497	55	0.932203	1.068943	0.366667	0.72277	FALSE	167	33
LV_ ED Length.Lt ED Length ZS(FL)[]	none	29	0.852941	1.621904	0	0.679031	55	0.932203	1.565827	0	0.721997	FALSE	91	34
4CV Circ (2Dist).4CV C ZS(AUA)[]	none	30	0.882353	1.075385	0	0.733912	54	0.915254	1.434154	0	0.721955	FALSE	56	35
LV_ ED Length.Lt ED Length ZS(HC)[]	none	30	0.882353	1.484868	0	0.702151	55	0.932203	1.603283	0	0.719754	FALSE	71	36
LV_ ED Length.Lt ED Length ZS (EFW)[]	none	30	0.882353	1.585392	0	0.695892	55	0.932203	1.612752	0	0.719197	FALSE	76	37
RV_ TAPSE lateral.TAPSE lat. ZS(FL)[]	none	29	0.852941	1.863671	0	0.666151	56	0.949153	1.773326	0	0.718807	FALSE	107	38
LV_ ED Length.Lt ED Length ZS (AC)[]	none	30	0.882353	1.666307	0	0.691197	56	0.949153	1.773601	0	0.718793	FALSE	79	39
LV_ CO.Lt CO ZS(AUA)[]	none	30	0.882353	1.435048	0	0.705444	54	0.915254	1.520502	0	0.716326	FALSE	70	40
RV_ TAPSE lateral.TAPSE lat. ZS (AC)[]	none	30	0.882353	1.907687	0	0.678743	56	0.949153	1.905353	0	0.712253	FALSE	93	41
LV_ ED Area.Lt ED Area % (EFW)[%]	centile	30	0.882353	2.04699	0.109091	0.661544	55	0.932203	1.285842	0.3	0.711092	FALSE	113	42
RV_ TAPSE lateral.TAPSE lat. ZS(BPD)[]	none	30	0.882353	1.8154	0	0.683252	55	0.932203	1.792584	0	0.709338	FALSE	83	43
RV_ TAPSE lateral.TAPSE lat. ZS(HC)[]	none	30	0.882353	1.813092	0	0.683369	55	0.932203	1.818592	0	0.708017	FALSE	82	44
LV_ ED Length.Lt ED Length ZS(AUA)[]	none	30	0.882353	1.540191	0	0.698645	54	0.915254	1.665702	0	0.707681	FALSE	73	45
LV_ Frac. Area change.Lt FRAC ZS (EFW)[]	none	30	0.882353	4.417508	0	0.615011	55	0.932203	1.858262	0	0.706047	FALSE	192	46
RV_ TAPSE lateral.TAPSE lat. ZS (EFW)[]	none	30	0.882353	1.883955	0	0.679875	55	0.932203	1.863604	0	0.705786	FALSE	90	47
LV_ Frac. Area change.Lt FRAC ZS(HC)[]	none	30	0.882353	4.461099	0	0.614422	55	0.932203	1.876969	0	0.705137	FALSE	196	48
LV_ Frac. Area change.Lt FRAC ZS (AC)[]	none	30	0.882353	4.441982	0	0.614679	56	0.949153	2.07217	0	0.704777	FALSE	194	49
LV_ Frac. Area change.Lt FRAC ZS(BPD)[]	none	30	0.882353	4.430305	0	0.614837	55	0.932203	1.891523	0	0.704437	FALSE	193	50
LV_ ED Area.Lt ED Area % (BPD)[%]	centile	30	0.882353	2.133152	0.109091	0.657934	55	0.932203	1.42545	0.266667	0.704353	FALSE	117	51

parameter	qc_rule	intra_n_usable	intra_usable_rate	intra_rel_mdc95	intra_max_flip_rate	intra_score	inter_n_usable	inter_usable_rate	inter_rel_mdc95	inter_max_flip_rate	inter_score	possible_copy_forward_inter	intra_rank	inter_rank
RV_ Sept.Wall Strain[%]	strain_raw	22	0.647059	1.484057	0	0.584556	50	0.847458	1.235116	0	0.70269	FALSE	251	52
RV_ Frac. Area change.Rt FRAC ZS (AC)[]	none	30	0.882353	2.044992	0	0.67254	56	0.949153	2.13132	0	0.702318	FALSE	101	53
LV_ SV/KG[ml/kg]	none	30	0.882353	4.008684	0	0.621038	55	0.932203	1.944638	0	0.701942	FALSE	180	54
LV_ CO/KG[ml/min/kg]	none	30	0.882353	4.72882	0	0.610999	55	0.932203	1.982242	0	0.700229	FALSE	206	55
LV_ MAPSE lateral.MAPSE lat. ZS (AC)[]	none	30	0.882353	2.590495	0	0.652582	56	0.949153	2.185879	0	0.70013	FALSE	129	56
LV_ MAPSE lateral.MAPSE lat. ZS(FL)[]	none	29	0.852941	2.431002	0	0.643055	56	0.949153	2.189474	0	0.699989	FALSE	150	57
LV_ SV.Lt SV ZS(AUA)[]	none	30	0.882353	1.717973	0	0.688345	54	0.915254	1.813259	0	0.699811	FALSE	81	58
RV_ TAPSE lateral.TAPSE lat. ZS(AUA)[]	none	30	0.882353	1.856156	0	0.681225	54	0.915254	1.836719	0	0.698635	FALSE	89	59
LV_ ED Area.Lt ED Area % (HC)[%]	centile	30	0.882353	2.400454	0.127273	0.646081	55	0.932203	1.524358	0.266667	0.697891	FALSE	145	60
LV_ Frac. Area change.Lt FRAC ZS(FL)[]	none	29	0.852941	4.420842	0	0.60026	55	0.932203	2.0422	0	0.697586	FALSE	227	61
LV_ EF[%]	percent_0_100	22	0.647059	0.734184	0	0.654185	45	0.762712	0.851033	0	0.697451	FALSE	122	62
LV_ ED Area.Lt ED Area % (AUA)[%]	centile	30	0.882353	2.25632	0.12963	0.651052	54	0.915254	1.410032	0.266667	0.696933	FALSE	131	63
LV_ MAPSE lateral.MAPSE lat. ZS(BPD)[]	none	30	0.882353	2.552679	0	0.653768	55	0.932203	2.076769	0	0.696108	FALSE	123	64
LV_ MAPSE lateral.MAPSE lat. ZS(HC)[]	none	30	0.882353	2.55645	0	0.653648	55	0.932203	2.089902	0	0.695556	FALSE	125	65
LV_ Frac. Area change[%]	percent_0_100	22	0.647059	0.77258	0	0.649189	45	0.762712	0.871831	0	0.69505	FALSE	136	66
RV_ Frac. Area change.Rt FRAC ZS (EFW)[]	none	30	0.882353	2.027562	0	0.673296	55	0.932203	2.103561	0	0.694986	FALSE	99	67
RV_ Frac. Area change.Rt FRAC ZS(HC)[]	none	30	0.882353	2.045597	0	0.672514	55	0.932203	2.106572	0	0.694861	FALSE	102	68
RV_ Frac. Area change.Rt FRAC ZS(BPD)[]	none	30	0.882353	2.046866	0	0.672459	55	0.932203	2.114489	0	0.694534	FALSE	103	69
RV_ ED Area.Rt ED Area % (GA)[%]	centile	28	0.823529	2.282633	0.196078	0.61401	51	0.864407	1.1404	0.25	0.694084	FALSE	198	70
RV_ Frac. Area change.Rt FRAC ZS(FL)[]	none	29	0.852941	2.064942	0	0.656979	55	0.932203	2.129092	0	0.693934	FALSE	119	71
LV_ ED Length.Lt ED Length ZS(GA)[]	none	28	0.823529	1.526117	0	0.67011	51	0.864407	1.490014	0	0.692845	FALSE	105	72
LV_ MAPSE lateral.MAPSE lat. ZS (EFW)[]	none	30	0.882353	2.562281	0	0.653464	55	0.932203	2.182077	0	0.691806	FALSE	126	73
RV_ ED Length.Rt ED Length ZS(FL)[]	none	29	0.852941	1.408412	0	0.692555	55	0.932203	2.193137	0	0.69137	FALSE	78	74
LV_ Basal-Sep.Wall FS.Lt Basal-Sep FS % (AC)[%]	centile	30	0.882353	1.726382	0.25	0.662891	56	0.949153	1.667017	0.333333	0.691223	FALSE	112	75
LV_ Basal-Sep.Wall FS.Lt Basal-Sep FS % (HC)[%]	centile	30	0.882353	1.748521	0.254545	0.661255	55	0.932203	1.586019	0.3	0.69078	FALSE	114	76
RV_ ED Length.Rt ED Length ZS(BPD)[]	none	30	0.882353	1.308719	0	0.714433	55	0.932203	2.215586	0	0.690496	FALSE	64	77
LV_ Basal-Sep.Wall FS.Lt Basal-Sep FS % (BPD)[%]	centile	30	0.882353	1.763287	0.254545	0.660477	55	0.932203	1.595685	0.3	0.690204	FALSE	115	78
RV_ Global Strain[%]	strain_raw	22	0.647059	2.187958	0	0.549002	52	0.881356	1.682834	0	0.689774	FALSE	320	79
LV_ Global Strain[%]	strain_raw	23	0.676471	1.066105	0	0.631836	50	0.847458	1.411869	0	0.689575	FALSE	165	80
RV_ ED Length.Rt ED Length % (AC)[%]	centile	30	0.882353	2.629156	0.232143	0.628181	56	0.949153	1.718409	0.333333	0.688388	FALSE	169	81
LV_ Frac. Area change.Lt FRAC ZS(AUA)[]	none	30	0.882353	4.450077	0	0.61457	54	0.915254	2.06267	0	0.688232	FALSE	195	82
RV_ ED Length.Rt ED Length ZS(HC)[]	none	30	0.882353	1.323425	0	0.713336	55	0.932203	2.281826	0	0.687985	FALSE	66	83
RV_ ED Length.Rt ED Length ZS (EFW)[]	none	30	0.882353	1.421576	0	0.706358	55	0.932203	2.300425	0	0.687298	FALSE	69	84
LV_ Basal-Sep.Wall FS.Lt Basal-Sep FS % (EFW)[%]	centile	30	0.882353	1.826696	0.254545	0.65723	55	0.932203	1.646279	0.3	0.687257	FALSE	118	85
RV_ FreeWall Strain[%]	strain_raw	22	0.647059	1.855231	0	0.563623	50	0.847458	1.455728	0	0.686613	FALSE	290	86
RV_ Frac. Area change.Rt FRAC ZS(AUA)[]	none	30	0.882353	2.031495	0	0.673125	54	0.915254	2.110648	0	0.686218	FALSE	100	87
RV_ Basal-Lat.Wall FS.Rt Basal-Lat FS ZS (EFW)[]	none	30	0.882353	2.632389	0	0.651297	55	0.932203	2.369338	0	0.684819	FALSE	130	88
RV_ Basal-Lat.Wall FS.Rt Basal-Lat FS ZS (AC)[]	none	30	0.882353	2.81066	0	0.646145	56	0.949153	2.687673	0	0.683046	FALSE	144	89
LV_ Basal-Sep.Wall FS.Lt Basal-Sep FS % (FL)[%]	centile	29	0.852941	1.708029	0.272727	0.646907	55	0.932203	1.651313	0.344828	0.682488	FALSE	143	90
RV_ Basal-Lat.Wall FS.Rt Basal-Lat FS ZS(FL)[]	none	29	0.852941	2.776549	0	0.632387	55	0.932203	2.45362	0	0.681922	FALSE	161	91
RV_ ED Length.Rt ED Length ZS (AC)[]	none	30	0.882353	1.550826	0	0.697988	56	0.949153	2.737754	0	0.681592	FALSE	74	92
LV_ EF.Lt EF ZS(AUA)[]	none	30	0.882353	7.684258	0	0.587237	54	0.915254	2.227835	0	0.681549	FALSE	243	93
LV_ MAPSE septal.MAPSE sept. % (AC)[%]	centile	30	0.882353	2.568412	0.285714	0.6247	56	0.949153	1.937591	0.3	0.680742	FALSE	173	94
RV_ Basal-Lat.Wall FS.Rt Basal-Lat FS ZS(BPD)[]	none	30	0.882353	2.708054	0	0.64905	55	0.932203	2.51405	0	0.67993	FALSE	137	95
RV_ Basal-Lat.Wall FS.Rt Basal-Lat FS ZS(HC)[]	none	30	0.882353	2.719051	0	0.648731	55	0.932203	2.526799	0	0.679519	FALSE	138	96

parameter	qc_rule	intra_n_usable	intra_usable_rate	intra_rel_mdc95	intra_max_flip_rate	intra_score	inter_n_usable	inter_usable_rate	inter_rel_mdc95	inter_max_flip_rate	inter_score	possible_copy_forward_inter	intra_rank	inter_rank
LV_ CO/KG.Lt CO/KG ZS(AUA)[]	none	30	0.882353	1.851759	0	0.681441	53	0.898305	2.076823	0	0.679157	FALSE	88	97
LV_ MAPSE lateral.MAPSE lat. ZS(AUA)[]	none	30	0.882353	2.554148	0	0.653721	54	0.915254	2.30737	0	0.678569	FALSE	124	98
RV_ ED Area.Rt ED Area ZS (AC)[]	none	30	0.882353	169.503824	0	0.543522	56	0.949153	2.878291	0	0.677714	FALSE	325	99
RV_ ED Length.Rt ED Length ZS(AUA)[]	none	30	0.882353	1.376302	0	0.709505	54	0.915254	2.34998	0	0.677031	FALSE	67	100
LV_ Basal-Sep.Wall FS.Lt Basal-Sep FS % (AUA)[%]	centile	30	0.882353	1.735467	0.240741	0.66333	54	0.915254	1.623514	0.333333	0.676761	FALSE	111	101
LV_ FreeWall Strain[%]	strain_raw	23	0.676471	1.365925	0	0.607302	50	0.847458	1.623798	0	0.67618	FALSE	218	102
LV_ CO.Lt CO ZS(BPD)[]	none	30	0.882353	1.836369	0	0.682202	51	0.864407	1.794201	0	0.675357	FALSE	85	103
RV_ ED Length.Rt ED Length % (HC)[%]	centile	30	0.882353	3.111359	0.272727	0.611195	55	0.932203	2.022062	0.233333	0.675128	FALSE	205	104
RV_ TAPSE lateral.TAPSE lat. ZS(GA)[]	none	28	0.823529	1.733639	0	0.65809	51	0.864407	1.799215	0	0.675101	FALSE	116	105
LV_ CO.Lt CO ZS(GA)[]	none	28	0.823529	1.467338	0	0.673883	51	0.864407	1.802814	0	0.674917	FALSE	98	106
RV_ ED Length.Rt ED Length % (EFW)[%]	centile	30	0.882353	2.792033	0.236364	0.623024	55	0.932203	1.887651	0.3	0.674623	FALSE	177	107
4CV Circ (2Dist).4CV C % (AC)[%]	centile	30	0.882353	0.045903	0	0.923621	56	0.949153	0	0	0.674576	TRUE	2	108
4CV Circ (2Dist).4CV C ZS(FL)[]	none	29	0.852941	1.565811	0	0.682367	56	0.949153	0	0	0.674576	TRUE	84	108
4CV Circ (2Dist).4CV C ZS (AC)[]	none	30	0.882353	0.919276	0	0.749588	56	0.949153	0	0	0.674576	TRUE	51	108
4CV Length ED[mm]	positive	30	0.882353	0.079295	0	0.911789	56	0.949153	0	0	0.674576	TRUE	4	108
4CV Area (2Dist).4CV A ZS(FL)[]	none	29	0.852941	1.787949	0	0.669945	56	0.949153	0	0	0.674576	TRUE	106	108
4CV Circ (2Dist)[mm]	positive	30	0.882353	0.056392	0	0.919824	56	0.949153	0	0	0.674576	TRUE	3	108
4CV Area (2Dist).4CV A % (AC)[%]	centile	30	0.882353	0.189421	0	0.877474	56	0.949153	0	0	0.674576	TRUE	6	108
4CV Length ED.4CV L ED ZS (AC)[]	none	30	0.882353	1.320871	0	0.713526	56	0.949153	0	0	0.674576	TRUE	65	108
4CV Trv. Width ED.4CV TW ED ZS(FL)[]	none	29	0.852941	2.913328	0	0.628685	56	0.949153	0	0	0.674576	TRUE	168	108
4CV Trv. Width ED.4CV TW ED % (AC)[%]	centile	30	0.882353	0.24131	0	0.863417	56	0.949153	0	0	0.674576	TRUE	11	108
4CV Length ED.4CV L ED % (AC)[%]	centile	30	0.882353	0.316738	0	0.844957	56	0.949153	0	0	0.674576	TRUE	19	108
4CV Trv. Width ED.4CV TW ED ZS (AC)[]	none	30	0.882353	3.943663	0	0.622088	56	0.949153	0	0	0.674576	TRUE	179	108
4CV Trv. Width ED[mm]	positive	30	0.882353	0.039218	0	0.926081	56	0.949153	0	0	0.674576	TRUE	1	108
4CV Length ED.4CV L ED ZS(FL)[]	none	29	0.852941	1.209101	0	0.70754	56	0.949153	0	0	0.674576	TRUE	68	108
4CV Area (2Dist)[mm¸]	none	30	0.882353	0.104454	0	0.903346	56	0.949153	0	0	0.674576	TRUE	5	108
4CV Area (2Dist).4CV A ZS (AC)[]	none	30	0.882353	4.267357	0	0.617116	56	0.949153	0	0	0.674576	TRUE	185	108
LV_ Sept.Wall Strain[%]	strain_raw	24	0.705882	1.207653	0	0.634129	48	0.813559	1.384818	0	0.674507	FALSE	155	124
RV_ ED Length.Rt ED Length % (FL)[%]	centile	29	0.852941	2.890179	0.218182	0.607475	55	0.932203	1.871245	0.310345	0.67438	FALSE	216	125
RV_ TAPSE septal.TAPSE sept. ZS(FL)[]	none	29	0.852941	2.727612	0	0.633778	55	0.932203	2.77582	0	0.672039	FALSE	157	126
RV_ ED Length.Rt ED Length % (BPD)[%]	centile	30	0.882353	2.880387	0.218182	0.622441	55	0.932203	2.025381	0.266667	0.67165	FALSE	178	127
LV_ ED Length.Lt ED Length % (BPD)[%]	centile	30	0.882353	3.385399	0.272727	0.605116	55	0.932203	1.888504	0.333333	0.671248	FALSE	222	128
4CV Circ (2Dist).4CV C % (FL)[%]	centile	29	0.852941	0.43605	0	0.805012	56	0.949153	0	0.034483	0.671128	TRUE	33	129
4CV Length ED.4CV L ED % (FL)[%]	centile	29	0.852941	0.377314	0	0.816891	56	0.949153	0	0.034483	0.671128	TRUE	30	129
4CV Area (2Dist).4CV A % (FL)[%]	centile	29	0.852941	0.445525	0	0.803187	56	0.949153	0	0.034483	0.671128	TRUE	36	129
4CV Trv. Width ED.4CV TW ED % (FL)[%]	centile	29	0.852941	0.459742	0	0.800492	56	0.949153	0	0.034483	0.671128	TRUE	37	129
LV_ ED Length.Lt ED Length % (AC)[%]	centile	30	0.882353	3.220694	0.285714	0.607376	56	0.949153	2.003849	0.366667	0.671072	FALSE	217	133
RV_ Basal-Lat.Wall FS.Rt Basal-Lat FS ZS(AUA)[]	none	30	0.882353	2.746206	0	0.647951	54	0.915254	2.539901	0	0.670625	FALSE	141	134
LV_ ED Length.Lt ED Length % (HC)[%]	centile	30	0.882353	3.605992	0.272727	0.600747	55	0.932203	1.989993	0.3	0.669881	FALSE	226	135
LV_ MAPSE septal.MAPSE sept. % (EFW)[%]	centile	30	0.882353	2.545367	0.218182	0.632182	55	0.932203	1.857063	0.366667	0.669439	FALSE	163	136
LV_ MAPSE septal.MAPSE sept. % (HC)[%]	centile	30	0.882353	2.649247	0.272727	0.623515	55	0.932203	1.879586	0.366667	0.668344	FALSE	174	137
LV_ MAPSE septal.MAPSE sept. % (BPD)[%]	centile	30	0.882353	2.6557	0.254545	0.62514	55	0.932203	1.886001	0.366667	0.668035	FALSE	172	138
LV_ MAPSE septal.MAPSE sept. % (FL)[%]	centile	29	0.852941	2.496787	0.267857	0.614076	56	0.949153	2.001818	0.413793	0.66645	FALSE	197	139
RV_ ED Length.Rt ED Length % (AUA)[%]	centile	30	0.882353	2.930984	0.240741	0.618858	54	0.915254	1.954427	0.266667	0.66635	FALSE	184	140
LV_ ED Length.Lt ED Length % (EFW)[%]	centile	30	0.882353	3.011302	0.272727	0.613622	55	0.932203	1.855342	0.4	0.66619	FALSE	199	141
4CV Circ (2Dist).4CV C ZS (EFW)[]	none	30	0.882353	0.889339	0	0.752891	55	0.932203	0	0	0.666102	TRUE	50	142
4CV Area (2Dist).4CV A ZS (EFW)[]	none	30	0.882353	0.684872	0	0.778583	55	0.932203	0	0	0.666102	TRUE	42	142
4CV Trv. Width ED.4CV TW ED ZS(HC)[]	none	30	0.882353	1.587635	0	0.695758	55	0.932203	0	0	0.666102	TRUE	77	142
4CV Area (2Dist).4CV A % (EFW)[%]	centile	30	0.882353	0.20025	0	0.87444	55	0.932203	0	0	0.666102	TRUE	7	142
4CV Circ (2Dist).4CV C ZS(HC)[]	none	30	0.882353	0.510102	0	0.806059	55	0.932203	0	0	0.666102	TRUE	32	142

parameter	qc_rule	intra_n_usable	intra_usable_rate	intra_rel_mdc95	intra_max_flip_rate	intra_score	inter_n_usable	inter_usable_rate	inter_rel_mdc95	inter_max_flip_rate	inter_score	possible_copy_forward_inter	intra_rank	inter_rank
4CV Trv. Width ED.4CV TW ED ZS (EFW)[]	none	30	0.882353	4.078418	0	0.619941	55	0.932203	0	0	0.666102	TRUE	181	142
4CV Trv. Width ED.4CV TW ED ZS(BPD)[]	none	30	0.882353	1.947647	0	0.676878	55	0.932203	0	0	0.666102	TRUE	94	142
4CV Circ (2Dist).4CV C % (EFW)[%]	centile	30	0.882353	0.226163	0	0.867397	55	0.932203	0	0	0.666102	TRUE	10	142
4CV Length ED.4CV L ED ZS(BPD)[]	none	30	0.882353	0.837321	0	0.758885	55	0.932203	0	0	0.666102	TRUE	47	142
4CV Circ (2Dist).4CV C % (HC)[%]	centile	30	0.882353	0.222098	0	0.868482	55	0.932203	0	0	0.666102	TRUE	9	142
4CV Circ (2Dist).4CV C ZS(BPD)[]	none	30	0.882353	0.859313	0	0.75631	55	0.932203	0	0	0.666102	TRUE	48	142
4CV Length ED.4CV L ED ZS (EFW)[]	none	30	0.882353	1.007234	0	0.740456	55	0.932203	0	0	0.666102	TRUE	53	142
4CV Area (2Dist).4CV A ZS(HC)[]	none	30	0.882353	0.595428	0	0.791893	55	0.932203	0	0	0.666102	TRUE	39	142
4CV Length ED.4CV L ED ZS(HC)[]	none	30	0.882353	0.871595	0	0.754898	55	0.932203	0	0	0.666102	TRUE	49	142
4CV Area (2Dist).4CV A ZS(BPD)[]	none	30	0.882353	0.653458	0	0.783094	55	0.932203	0	0	0.666102	TRUE	41	142
RV_ TAPSE septal.TAPSE sept. ZS (AC)[]	none	30	0.882353	2.821843	0	0.645838	55	0.932203	3.002055	0	0.66605	FALSE	146	157
LV_ Basal-Apical FS.Lt BASE-AP FS %[%]	centile	30	0.882353	3.096035	0.321429	0.606689	56	0.949153	2.632942	0.2	0.66468	FALSE	221	158
LV_ ED Length.Lt ED Length % (FL)[%]	centile	29	0.852941	3.20774	0.254545	0.596079	55	0.932203	2.016237	0.344828	0.664234	FALSE	231	159
LV_ ED Area.Lt ED Area % (GA)[%]	centile	28	0.823529	2.005386	0.176471	0.627212	51	0.864407	1.439519	0.321429	0.664027	FALSE	170	160
LV_ EF.Lt EF ZS(BPD)[]	none	30	0.882353	7.726619	0	0.587013	51	0.864407	2.038448	0	0.66385	FALSE	244	161
RV_ TAPSE septal.TAPSE sept. ZS(BPD)[]	none	30	0.882353	2.661065	0	0.650434	54	0.915254	2.776163	0	0.663555	FALSE	134	162
LV_ Frac. Area change.Lt FRAC ZS(GA)[]	none	28	0.823529	4.849648	0	0.580145	51	0.864407	2.055004	0	0.663136	FALSE	264	163
4CV Trv. Width ED.4CV TW ED % (HC)[%]	centile	30	0.882353	0.35845	0	0.83563	55	0.932203	0	0.033333	0.662768	TRUE	24	164
4CV Area (2Dist).4CV A % (HC)[%]	centile	30	0.882353	0.278282	0	0.854097	55	0.932203	0	0.033333	0.662768	TRUE	13	164
4CV Circ (2Dist).4CV C % (BPD)[%]	centile	30	0.882353	0.346481	0	0.838247	55	0.932203	0	0.033333	0.662768	TRUE	23	164
4CV Trv. Width ED.4CV TW ED % (EFW)[%]	centile	30	0.882353	0.36621	0	0.833957	55	0.932203	0	0.033333	0.662768	TRUE	25	164
4CV Trv. Width ED.4CV TW ED % (BPD)[%]	centile	30	0.882353	0.372159	0	0.832688	55	0.932203	0	0.033333	0.662768	TRUE	26	164
4CV Length ED.4CV L ED % (HC)[%]	centile	30	0.882353	0.338164	0	0.840094	55	0.932203	0	0.033333	0.662768	TRUE	21	164
4CV Area (2Dist).4CV A % (BPD)[%]	centile	30	0.882353	0.29097	0	0.851021	55	0.932203	0	0.033333	0.662768	TRUE	14	164
4CV Length ED.4CV L ED % (EFW)[%]	centile	30	0.882353	0.304642	0	0.847774	55	0.932203	0	0.033333	0.662768	TRUE	18	164
4CV Length ED.4CV L ED % (BPD)[%]	centile	30	0.882353	0.25373	0	0.860224	55	0.932203	0	0.033333	0.662768	TRUE	12	164
LV_ EDV.Lt EDV % (AUA)[%]	centile	30	0.882353	2.701629	0.259259	0.623311	54	0.915254	2.037368	0.266667	0.662653	FALSE	175	173
RV_ Frac. Area change.Rt FRAC ZS(GA)[]	none	28	0.823529	2.021143	0	0.644165	51	0.864407	2.076052	0	0.66224	FALSE	149	174
LV_ SV/KG.Lt SV/KG ZS(AUA)[]	none	30	0.882353	1.986075	0	0.675132	53	0.898305	2.545539	0	0.66197	FALSE	96	175
LV_ MAPSE lateral.MAPSE lat. ZS(GA)[]	none	28	0.823529	2.589936	0	0.623187	51	0.864407	2.090158	0	0.661647	FALSE	176	176
RV_ TAPSE septal.TAPSE sept. ZS(HC)[]	none	30	0.882353	2.731007	0	0.648386	54	0.915254	2.847635	0	0.661587	FALSE	139	177
RV_ Frac. Area change[%]	percent_0_100	18	0.529412	1.195642	0	0.546885	43	0.728814	1.037268	0	0.660748	FALSE	322	178
LV_ EDV.Lt EDV % (GA)[%]	centile	28	0.823529	2.370193	0.176471	0.612805	51	0.864407	1.610269	0.25	0.660444	FALSE	201	179
LV_ Basal-Lat.Wall FS.Lt Basal-Lat FS % (AC)[%]	centile	30	0.882353	2.834174	0.375	0.608001	56	0.949153	2.784877	0.2	0.66026	FALSE	214	180
RV_ Basal-Apical FS[%]	percent_0_100	16	0.470588	1.775925	0	0.47939	45	0.762712	1.242376	0	0.659738	FALSE	347	181
RV_ Basal-Sep.Wall FS.Rt Basal-Sep FS ZS (AC)[]	none	30	0.882353	3.432747	0	0.631414	56	0.949153	3.727044	0	0.659196	FALSE	166	182
RV_ TAPSE septal.TAPSE sept. ZS (EFW)[]	none	30	0.882353	2.833417	0	0.645522	54	0.915254	2.944817	0	0.659026	FALSE	147	183
LV_ CO[ml/min]	positive	22	0.647059	1.892895	0	0.561799	45	0.762712	1.251453	0	0.659019	FALSE	292	184
LV_ SV.Lt SV ZS(GA)[]	none	28	0.823529	1.615438	0	0.664703	51	0.864407	2.223079	0	0.656308	FALSE	108	185
LV_ Basal-Lat.Wall FS.Lt Basal-Lat FS ZS (AC)[]	none	30	0.882353	4.784003	0	0.610333	56	0.949153	3.900885	0	0.656194	FALSE	208	186
LV_ Basal-Lat.Wall FS.Lt Basal-Lat FS % (BPD)[%]	centile	30	0.882353	2.773165	0.345455	0.612643	55	0.932203	2.650679	0.2	0.65567	FALSE	202	187
LV_ EDV.Lt EDV % (BPD)[%]	centile	30	0.882353	2.344819	0.215686	0.639196	51	0.864407	1.726357	0.233333	0.655586	FALSE	152	188
LV_ EDV.Lt EDV ZS(AUA)[]	none	30	0.882353	2.094675	0	0.670431	54	0.915254	3.086429	0	0.655512	FALSE	104	189
LV_ Basal-Lat.Wall FS.Lt Basal-Lat FS % (FL)[%]	centile	29	0.852941	2.858977	0.363636	0.593761	55	0.932203	2.650092	0.206897	0.654998	FALSE	235	190
LV_ EF.Lt EF ZS(GA)[]	none	28	0.823529	8.268992	0	0.554919	51	0.864407	2.257478	0	0.654998	FALSE	310	191
LV_ Basal-Lat.Wall FS.Lt Basal-Lat FS % (HC)[%]	centile	30	0.882353	2.807534	0.363636	0.609868	55	0.932203	2.691288	0.2	0.654465	FALSE	209	192
RV_ Basal-Sep.Wall FS.Rt Basal-Sep FS ZS (EFW)[]	none	30	0.882353	3.329678	0	0.633562	55	0.932203	3.526862	0	0.654463	FALSE	158	193
LV_ CO/KG.Lt CO/KG ZS(BPD)[]	none	30	0.882353	1.83861	0	0.682091	51	0.864407	2.288848	0	0.653827	FALSE	86	194
LV_ ED Area.Lt ED Area ZS(BPD)[]	none	30	0.882353	2.259204	0	0.663906	55	0.932203	3.568308	0	0.653661	FALSE	109	195

parameter	qc_rule	intra_n_usable	intra_usable_rate	intra_rel_mdc95	intra_max_flip_rate	intra_score	inter_n_usable	inter_usable_rate	inter_rel_mdc95	inter_max_flip_rate	inter_score	possible_copy_forward_inter	intra_rank	inter_rank
LV_ Basal-Apical FS[%]	percent_0_100	21	0.617647	1.245978	0	0.58692	44	0.745763	1.214602	0	0.653501	FALSE	245	196
RV_ Basal-Lat.Wall FS.Rt Basal-Lat FS % (AC)[%]	centile	30	0.882353	4.201922	0.410714	0.577	56	0.949153	2.674368	0.3	0.653439	FALSE	274	197
RV_ Basal-Lat.Wall FS[%]	percent_0_100	17	0.5	1.182836	0	0.533248	45	0.762712	1.325448	0	0.653366	FALSE	333	198
RV_ Basal-Sep.Wall FS.Rt Basal-Sep FS ZS(BPD)[]	none	30	0.882353	3.316034	0	0.633854	55	0.932203	3.587158	0	0.653302	FALSE	156	199
RV_ Basal-Sep.Wall FS.Rt Basal-Sep FS ZS(HC)[]	none	30	0.882353	3.337056	0	0.633405	55	0.932203	3.607907	0	0.652909	FALSE	159	200
LV_ Basal-Lat.Wall FS.Lt Basal-Lat FS % (EFW)[%]	centile	30	0.882353	2.726941	0.327273	0.615776	55	0.932203	2.642561	0.233333	0.652581	FALSE	189	201
LV_ ED Length.Lt ED Length % (AUA)[%]	centile	30	0.882353	3.35986	0.259259	0.606997	54	0.915254	1.96541	0.4	0.652516	FALSE	219	202
LV_ SV[ml]	positive	22	0.647059	1.701264	0	0.571608	45	0.762712	1.339709	0	0.652317	FALSE	284	203
RV_ Basal-Sep.Wall FS.Rt Basal-Sep FS ZS(FL)[]	none	29	0.852941	3.4426	0	0.616508	55	0.932203	3.653791	0	0.652053	FALSE	186	204
RV_ ED Length.Rt ED Length ZS(GA)[]	none	28	0.823529	1.394068	0	0.678844	51	0.864407	2.391138	0	0.650158	FALSE	92	205
LV_ Basal-Lat.Wall FS.Lt Basal-Lat FS ZS(FL)[]	none	29	0.852941	4.503771	0	0.599148	55	0.932203	3.779138	0	0.649799	FALSE	228	206
RV_ TAPSE septal.TAPSE sept. ZS(AUA)[]	none	30	0.882353	2.755293	0	0.647693	53	0.898305	2.98251	0	0.649592	FALSE	142	207
LV_ FreeWall Strain.Lt FWS ZS[]	zscore	27	0.794118	2.293432	0.4	0.578513	55	0.932203	2.429363	0.333333	0.649408	FALSE	270	208
LV_ Basal-Lat.Wall FS.Lt Basal-Lat FS ZS(HC)[]	none	30	0.882353	4.892295	0	0.609062	55	0.932203	3.892449	0	0.64786	FALSE	212	209
LV_ Basal-Sep.Wall FS[%]	percent_0_100	20	0.588235	1.019598	0	0.592177	42	0.711864	1.095816	0	0.646789	FALSE	236	210
RV_ Basal-Apical FS.Rt BASE-AP FS %[%]	centile	30	0.882353	5.677524	0.446429	0.556436	56	0.949153	2.921626	0.3	0.646575	FALSE	305	211
LV_ Basal-Lat.Wall FS.Lt Basal-Lat FS ZS(BPD)[]	none	30	0.882353	4.947368	0	0.608433	55	0.932203	3.981383	0	0.646401	FALSE	213	212
LV_ Basal-Lat.Wall FS.Lt Basal-Lat FS % (AUA)[%]	centile	30	0.882353	2.781542	0.351852	0.611768	54	0.915254	2.686731	0.2	0.646124	FALSE	204	213
RV_ Basal-Lat.Wall FS.Rt Basal-Lat FS % (BPD)[%]	centile	30	0.882353	4.376487	0.363636	0.579211	55	0.932203	2.761471	0.266667	0.645776	FALSE	268	214
RV_ TAPSE lateral[mm]	tapse_mm	16	0.470588	1.036064	0	0.531752	45	0.762712	1.435362	0	0.645603	FALSE	334	215
RV_ Basal-Lat.Wall FS.Rt Basal-Lat FS ZS(GA)[]	none	28	0.823529	2.708165	0	0.619635	51	0.864407	2.528268	0	0.645573	FALSE	183	216
LV_ Basal-Lat.Wall FS.Lt Basal-Lat FS ZS (EFW)[]	none	30	0.882353	4.989465	0	0.60796	55	0.932203	4.047799	0	0.645344	FALSE	215	217
RV_ Basal-Lat.Wall FS.Rt Basal-Lat FS % (FL)[%]	centile	29	0.852941	4.279376	0.345455	0.567692	55	0.932203	2.757407	0.275862	0.644972	FALSE	286	218
RV_ Basal-Lat.Wall FS.Rt Basal-Lat FS % (HC)[%]	centile	30	0.882353	4.331958	0.363636	0.579832	55	0.932203	2.697019	0.3	0.644297	FALSE	266	219
RV_ Basal-Lat.Wall FS.Rt Basal-Lat FS % (EFW)[%]	centile	30	0.882353	4.415979	0.327273	0.582305	55	0.932203	2.969219	0.233333	0.643544	FALSE	260	220
RV_ Basal-Sep.Wall FS.Rt Basal-Sep FS ZS(AUA)[]	none	30	0.882353	3.363231	0	0.632852	54	0.915254	3.669233	0	0.643294	FALSE	160	221
LV_ CO/KG.Lt CO/KG ZS(GA)[]	none	28	0.823529	1.839446	0	0.652637	50	0.847458	2.348028	0	0.643202	FALSE	128	222
LV_ MAPSE septal[mm]	positive	20	0.588235	1.225844	0	0.573825	42	0.711864	1.139322	0	0.642907	FALSE	282	223
LV_ ED Length.Lt ED Length % (GA)[%]	centile	28	0.823529	3.395206	0.215686	0.581204	51	0.864407	1.807187	0.321429	0.642552	FALSE	262	224
LV_ Basal-Sep.Wall FS.Lt Basal-Sep FS % (GA)[%]	centile	28	0.823529	1.703736	0.27451	0.632257	51	0.864407	1.739157	0.357143	0.642519	FALSE	162	225
LV_ SV.Lt SV % (BPD)[%]	centile	30	0.882353	3.435921	0.156863	0.615663	51	0.864407	2.522838	0.033333	0.642415	FALSE	190	226
RV_ ED Length.Rt ED Length % (GA)[%]	centile	28	0.823529	2.828772	0.215686	0.594668	51	0.864407	1.815151	0.321429	0.642149	FALSE	232	227
LV_ ED Area.Lt ED Area ZS(HC)[]	none	30	0.882353	2.266085	0	0.663647	55	0.932203	4.340239	0	0.641005	FALSE	110	228
LV_ Basal-Lat.Wall FS[%]	percent_0_100	21	0.617647	1.401309	0	0.575399	43	0.728814	1.266413	0	0.640897	FALSE	277	229
RV_ TAPSE lateral.TAPSE lat. % (AC)[%]	centile	30	0.882353	4.909959	0.303571	0.578502	56	0.949153	3.41473	0.266667	0.638515	FALSE	271	230
RV_ Basal-Lat.Wall FS.Rt Basal-Lat FS % (AUA)[%]	centile	30	0.882353	4.311424	0.333333	0.583153	54	0.915254	2.611041	0.3	0.638398	FALSE	258	231
LV_ Frac. Area change.Lt FRAC % (AC)[%]	centile	30	0.882353	4.090097	0.410714	0.578689	56	0.949153	4.016266	0.166667	0.63765	FALSE	269	232
LV_ SV/KG.Lt SV/KG ZS(BPD)[]	none	30	0.882353	1.988258	0	0.675034	51	0.864407	2.829373	0	0.636659	FALSE	97	233
LV_ ED Area.Lt ED Area ZS (EFW)[]	none	30	0.882353	2.679959	0	0.649873	55	0.932203	4.707186	0	0.636189	FALSE	135	234
RV_ TAPSE lateral.TAPSE lat. % (FL)[%]	centile	29	0.852941	5.350686	0.285714	0.560885	56	0.949153	3.566239	0.275862	0.63459	FALSE	294	235
LV_ Basal-Lat.Wall FS.Lt Basal-Lat FS ZS(AUA)[]	none	30	0.882353	4.877527	0	0.609232	54	0.915254	4.329024	0	0.632688	FALSE	211	236
4CV Trv. Width ED.4CV TW ED ZS(GA)[]	none	28	0.823529	1.232145	0	0.690965	51	0.864407	0	0	0.632203	TRUE	80	237
4CV Circ (2Dist).4CV C % (GA)[%]	centile	28	0.823529	0.218699	0	0.839984	51	0.864407	0	0	0.632203	TRUE	22	237
4CV Circ (2Dist).4CV C ZS(GA)[]	none	28	0.823529	0.446866	0	0.788224	51	0.864407	0	0	0.632203	TRUE	40	237

parameter	qc_rule	intra_n_usable	intra_usable_rate	intra_rel_mdc95	intra_max_flip_rate	intra_score	inter_n_usable	inter_usable_rate	inter_rel_mdc95	inter_max_flip_rate	inter_score	possible_copy_forward_inter	intra_rank	inter_rank
4CV Length ED.4CV L ED ZS(GA)[]	none	28	0.823529	0.571981	0	0.766221	51	0.864407	0	0	0.632203	TRUE	45	237
LV_ Frac. Area change.Lt FRAC % (HC)[%]	centile	30	0.882353	4.131074	0.345455	0.584587	55	0.932203	4.078665	0.133333	0.631529	FALSE	250	241
RV_ TAPSE lateral.TAPSE lat. % (EFW)[%]	centile	30	0.882353	5.134868	0.327273	0.57365	55	0.932203	3.381649	0.266667	0.630725	FALSE	283	242
LV_ Frac. Area change.Lt FRAC % (FL)[%]	centile	29	0.852941	2.904852	0.309091	0.597998	55	0.932203	4.109631	0.137931	0.630592	FALSE	229	243
LV_ Sept.Wall Strain.Lt SWS ZS[]	zscore	29	0.852941	2.899912	0.482143	0.580823	56	0.949153	3.61159	0.310345	0.63028	FALSE	263	244
RV_ TAPSE septal.TAPSE sept. % (AC)[%]	centile	30	0.882353	6.556734	0.436364	0.550473	55	0.932203	3.621573	0.233333	0.629319	FALSE	317	245
RV_ TAPSE septal.TAPSE sept. % (FL)[%]	centile	29	0.852941	6.824102	0.4	0.537595	55	0.932203	3.791085	0.206897	0.6289	FALSE	330	246
RV_ TAPSE lateral.TAPSE lat. % (HC)[%]	centile	30	0.882353	5.756856	0.254545	0.574921	55	0.932203	3.483562	0.266667	0.62865	FALSE	278	247
4CV Trv. Width ED.4CV TW ED % (GA)[%]	centile	28	0.823529	0.317619	0	0.815343	51	0.864407	0	0.035714	0.628632	TRUE	31	248
4CV Length ED.4CV L ED % (GA)[%]	centile	28	0.823529	0.108445	0	0.872631	51	0.864407	0	0.035714	0.628632	TRUE	8	248
RV_ Global Strain.Rt GLS ZS[]	zscore	25	0.735294	2.109592	0.415094	0.554772	53	0.898305	2.881821	0.24	0.628197	FALSE	312	250
LV_ Frac. Area change.Lt FRAC % (BPD)[%]	centile	30	0.882353	4.159512	0.345455	0.584158	55	0.932203	4.092784	0.166667	0.627977	FALSE	253	251
LV_ SV/KG.Lt SV/KG ZS(GA)[]	none	28	0.823529	1.933794	0	0.648107	50	0.847458	2.846557	0	0.627718	FALSE	140	252
RV_ TAPSE lateral.TAPSE lat. % (BPD)[%]	centile	30	0.882353	5.564463	0.272727	0.574838	55	0.932203	3.545102	0.266667	0.627442	FALSE	280	253
RV_ FreeWall Strain.Rt FWS ZS[]	zscore	25	0.735294	2.108065	0.415094	0.554835	53	0.898305	2.927239	0.24	0.627005	FALSE	311	254
LV_ ED Area.Lt ED Area ZS(AUA)[]	none	30	0.882353	2.578268	0	0.652962	54	0.915254	4.78784	0	0.626738	FALSE	127	255
LV_ MAPSE septal.MAPSE sept. ZS(FL)[]	none	29	0.852941	4.25018	0	0.602658	56	0.949153	6.96306	0	0.624808	FALSE	225	256
LV_ MAPSE lateral.MAPSE lat. % (FL)[%]	centile	29	0.852941	4.116373	0.375	0.567151	56	0.949153	4.17046	0.275862	0.624353	FALSE	287	257
RV_ TAPSE septal.TAPSE sept. ZS(GA)[]	none	28	0.823529	2.836431	0	0.616028	50	0.847458	3.005963	0	0.62358	FALSE	187	258
RV_ Sept.Wall Strain.Rt SWS ZS[]	zscore	29	0.852941	3.228488	0.321429	0.588924	56	0.949153	3.796995	0.344828	0.623479	FALSE	240	259
RV_ TAPSE lateral.TAPSE lat. % (AUA)[%]	centile	30	0.882353	5.462842	0.259259	0.577143	54	0.915254	3.339915	0.266667	0.623128	FALSE	273	260
LV_ Frac. Area change.Lt FRAC % (EFW)[%]	centile	30	0.882353	4.223665	0.272727	0.590478	55	0.932203	4.202984	0.2	0.622981	FALSE	238	261
LV_ Basal-Lat.Wall FS.Lt Basal-Lat FS ZS(GA)[]	none	28	0.823529	5.188003	0	0.576406	51	0.864407	3.409961	0	0.622907	FALSE	275	262
LV_ MAPSE lateral[mm]	positive	20	0.588235	1.445486	0	0.557684	42	0.711864	1.406287	0	0.622163	FALSE	300	263
LV_ MAPSE lateral.MAPSE lat. % (AC)[%]	centile	30	0.882353	4.028836	0.375	0.583218	56	0.949153	4.175893	0.3	0.621858	FALSE	257	264
LV_ Basal-Apical FS.Lt BASE-AP FS ZS[]	zscore	28	0.823529	2.598818	0.454545	0.577458	55	0.932203	4.09888	0.25	0.61955	FALSE	272	265
LV_ MAPSE septal.MAPSE sept. ZS (AC)[]	none	30	0.882353	4.839546	0	0.609675	56	0.949153	7.912449	0	0.619457	FALSE	210	266
LV_ MAPSE septal.MAPSE sept. % (GA)[%]	centile	28	0.823529	2.524944	0.313725	0.593869	51	0.864407	2.074895	0.428571	0.619432	FALSE	234	267
RV_ TAPSE septal.TAPSE sept. % (HC)[%]	centile	30	0.882353	6.788523	0.388889	0.553645	54	0.915254	3.746662	0.233333	0.618564	FALSE	314	268
RV_ Frac. Area change.Rt FRAC % (AC)[%]	centile	30	0.882353	9.033657	0.232143	0.557828	56	0.949153	4.986756	0.233333	0.618057	FALSE	299	269
RV_ TAPSE septal.TAPSE sept. % (EFW)[%]	centile	30	0.882353	6.568931	0.407407	0.553283	54	0.915254	3.59323	0.266667	0.618045	FALSE	315	270
LV_ Frac. Area change.Lt FRAC % (AUA)[%]	centile	30	0.882353	4.092111	0.314815	0.588248	54	0.915254	4.201525	0.166667	0.617861	FALSE	241	271
LV_ MAPSE septal.MAPSE sept. ZS(BPD)[]	none	30	0.882353	4.578145	0	0.612885	55	0.932203	6.794413	0	0.617421	FALSE	200	272
RV_ Basal-Sep.Wall FS.Rt Basal-Sep FS ZS(GA)[]	none	28	0.823529	3.647299	0	0.597836	51	0.864407	3.755398	0	0.616318	FALSE	230	273
LV_ MAPSE lateral.MAPSE lat. % (EFW)[%]	centile	30	0.882353	3.85491	0.327273	0.59084	55	0.932203	3.798351	0.333333	0.61613	FALSE	237	274
RV_ TAPSE septal.TAPSE sept. % (AUA)[%]	centile	30	0.882353	6.69166	0.377358	0.555445	53	0.898305	3.609237	0.2	0.615935	FALSE	308	275
RV_ TAPSE septal.TAPSE sept. % (BPD)[%]	centile	30	0.882353	6.791665	0.37037	0.555476	54	0.915254	3.929967	0.233333	0.61543	FALSE	307	276
RV_ Basal-Sep.Wall FS.Rt Basal-Sep FS % (HC)[%]	centile	30	0.882353	5.744509	0.4	0.560484	55	0.932203	2.896913	0.533333	0.615414	FALSE	295	277
LV_ MAPSE septal.MAPSE sept. ZS (EFW)[]	none	30	0.882353	4.744144	0	0.610813	55	0.932203	7.164928	0	0.615092	FALSE	207	278
RV_ Basal-Sep.Wall FS.Rt Basal-Sep FS % (BPD)[%]	centile	30	0.882353	5.758977	0.4	0.560357	55	0.932203	2.912983	0.533333	0.614992	FALSE	296	279
LV_ MAPSE septal.MAPSE sept. ZS(HC)[]	none	30	0.882353	4.660396	0	0.611843	55	0.932203	7.437724	0	0.613508	FALSE	203	280
RV_ ED Area.Rt ED Area ZS (EFW)[]	none	30	0.882353	5.44323	0	0.603257	55	0.932203	7.484209	0	0.613248	FALSE	223	281
RV_ Basal-Sep.Wall FS[%]	percent_0_100	14	0.411765	2.604507	0	0.416855	40	0.677966	1.297066	0	0.613118	FALSE	367	282
LV_ EF.Lt EF % (AUA)[%]	centile	30	0.882353	4.409205	0.314815	0.583643	54	0.915254	4.361662	0.2	0.612231	FALSE	256	283

parameter	qc_rule	intra_n_usable	intra_usable_rate	intra_rel_mdc95	intra_max_flip_rate	intra_score	inter_n_usable	inter_usable_rate	inter_rel_mdc95	inter_max_flip_rate	inter_score	possible_copy_forward_inter	intra_rank	inter_rank
LV_ SV.Lt SV % (AUA)[%]	centile	30	0.882353	5.171589	0.259259	0.580064	54	0.915254	4.636622	0.166667	0.611925	FALSE	265	284
LV_ Basal-Lat.Wall FS.Lt Basal-Lat FS % (GA)[%]	centile	28	0.823529	2.604859	0.333333	0.589393	51	0.864407	2.704457	0.285714	0.61161	FALSE	239	285
LV_ SV/KG.Lt SV/KG % (AUA)[%]	centile	30	0.882353	4.20874	0.358491	0.582121	53	0.898305	3.336205	0.3	0.611399	FALSE	261	286
RV_ Basal-Sep.Wall FS.Rt Basal-Sep FS % (EFW)[%]	centile	30	0.882353	5.78741	0.418182	0.558291	55	0.932203	2.933188	0.566667	0.611134	FALSE	297	287
RV_ Frac. Area change.Rt FRAC % (EFW)[%]	centile	30	0.882353	9.154705	0.236364	0.556931	55	0.932203	4.99483	0.233333	0.609493	FALSE	303	288
LV_ MAPSE lateral.MAPSE lat. % (HC)[%]	centile	30	0.882353	4.258224	0.309091	0.586339	55	0.932203	4.472556	0.3	0.609194	FALSE	246	289
RV_ Frac. Area change.Rt FRAC % (BPD)[%]	centile	30	0.882353	9.140413	0.236364	0.556986	55	0.932203	5.022526	0.233333	0.609186	FALSE	302	290
RV_ Basal-Sep.Wall FS.Rt Basal-Sep FS % (AC)[%]	centile	30	0.882353	5.619426	0.375	0.564105	56	0.949153	2.844988	0.7	0.608608	FALSE	289	291
LV_ ED Area.Lt ED Area ZS(FL)[]	none	29	0.852941	3.469952	0	0.615957	55	0.932203	8.468134	0	0.608349	FALSE	188	292
LV_ MAPSE lateral.MAPSE lat. % (BPD)[%]	centile	30	0.882353	4.1405	0.327273	0.586263	55	0.932203	4.302824	0.333333	0.6082	FALSE	247	293
RV_ Frac. Area change.Rt FRAC % (HC)[%]	centile	30	0.882353	9.191185	0.236364	0.55679	55	0.932203	5.136417	0.233333	0.607953	FALSE	304	294
RV_ Frac. Area change.Rt FRAC % (FL)[%]	centile	29	0.852941	9.078901	0.236364	0.542521	55	0.932203	5.066263	0.241379	0.607902	FALSE	326	295
RV_ Basal-Lat.Wall FS.Rt Basal-Lat FS % (GA)[%]	centile	28	0.823529	4.20147	0.392157	0.54945	51	0.864407	2.850623	0.285714	0.607511	FALSE	319	296
RV_ Basal-Sep.Wall FS.Rt Basal-Sep FS % (FL)[%]	centile	29	0.852941	5.669518	0.4	0.546445	55	0.932203	2.882904	0.62069	0.607048	FALSE	323	297
LV_ CO.Lt CO % (AUA)[%]	centile	30	0.882353	6.446347	0.203704	0.574524	54	0.915254	5.414039	0.133333	0.606657	FALSE	281	298
LV_ EDV.Lt EDV ZS(GA)[]	none	28	0.823529	1.765398	0	0.656409	51	0.864407	4.401962	0	0.606251	FALSE	121	299
LV_ CO/KG.Lt CO/KG % (AUA)[%]	centile	30	0.882353	5.502474	0.471698	0.555522	53	0.898305	3.842118	0.266667	0.605094	FALSE	306	300
LV_ ED Area.Lt ED Area ZS(GA)[]	none	28	0.823529	2.474809	0	0.626879	51	0.864407	4.687121	0	0.602538	FALSE	171	301
LV_ ED Area.Lt ED Area ZS (AC)[]	none	30	0.882353	6.505832	0	0.594468	56	0.949153	13.362012	0	0.602428	FALSE	233	302
LV_ MAPSE lateral.MAPSE lat. % (AUA)[%]	centile	30	0.882353	4.14927	0.351852	0.583672	54	0.915254	4.140881	0.333333	0.602102	FALSE	255	303
RV_ Basal-Sep.Wall FS.Rt Basal-Sep FS % (AUA)[%]	centile	30	0.882353	5.725526	0.388889	0.561762	54	0.915254	2.873832	0.6	0.600884	FALSE	293	304
LV_ SV.Lt SV % (GA)[%]	centile	28	0.823529	4.096006	0.352941	0.554963	51	0.864407	4.038835	0.107143	0.600873	FALSE	309	305
RV_ Frac. Area change.Rt FRAC % (AUA)[%]	centile	30	0.882353	9.248791	0.222222	0.557983	54	0.915254	5.057606	0.233333	0.600327	FALSE	298	306
RV_ Basal-Apical FS.Rt BASE-AP FS ZS[]	zscore	25	0.735294	2.739372	0.566038	0.518013	53	0.898305	3.240085	0.44	0.59949	FALSE	340	307
RV_ ED Area.Rt ED Area ZS(AUA)[]	none	30	0.882353	4.0799	0	0.619918	54	0.915254	8.560728	0	0.599465	FALSE	182	308
LV_ EF.Lt EF % (GA)[%]	centile	28	0.823529	4.443283	0.333333	0.551916	51	0.864407	3.843074	0.178571	0.596938	FALSE	316	309
LV_ Frac. Area change.Lt FRAC % (GA)[%]	centile	28	0.823529	4.142355	0.392157	0.550334	51	0.864407	3.860091	0.178571	0.596649	FALSE	318	310
RV_ TAPSE septal[mm]	tapse_mm	12	0.352941	2.424018	0	0.393292	39	0.661017	1.441278	0	0.594357	FALSE	378	311
LV_ Basal-Sep.Wall FS.Lt Basal-Sep FS ZS (EFW)[]	none	30	0.882353	7.674802	0	0.587287	55	0.932203	14.378863	0	0.592111	FALSE	242	312
RV_ TAPSE lateral.TAPSE lat. % (GA)[%]	centile	28	0.823529	5.576275	0.313725	0.541217	51	0.864407	3.737174	0.25	0.591642	FALSE	327	313
LV_ Basal-Sep.Wall FS.Lt Basal-Sep FS ZS (AC)[]	none	30	0.882353	8.563793	0	0.583001	56	0.949153	23.273659	0	0.591055	FALSE	259	314
LV_ SV/KG.Lt SV/KG % (GA)[%]	centile	28	0.823529	4.006307	0.34	0.557664	50	0.847458	3.237223	0.285714	0.589559	FALSE	301	315
LV_ Basal-Sep.Wall FS.Lt Basal-Sep FS ZS(FL)[]	none	29	0.852941	7.266635	0	0.574858	55	0.932203	16.173613	0	0.589393	FALSE	279	316
LV_ SV.Lt SV ZS(BPD)[]	none	30	0.882353	17.572248	0	0.562714	51	0.864407	6.183842	0	0.587884	FALSE	291	317
LV_ Global Strain.Lt GLS ZS[]	zscore	24	0.705882	2.212906	0.54902	0.522537	51	0.864407	3.974378	0.25	0.587615	FALSE	339	318
LV_ SV/KG.Lt SV/KG % (BPD)[%]	centile	30	0.882353	4.307258	0.372549	0.57929	51	0.864407	3.556992	0.333333	0.586647	FALSE	267	319
LV_ EF.Lt EF % (BPD)[%]	centile	30	0.882353	4.560669	0.27451	0.585659	51	0.864407	4.45552	0.2	0.585524	FALSE	248	320
LV_ CO.Lt CO % (BPD)[%]	centile	30	0.882353	5.584235	0.313725	0.570555	51	0.864407	4.467259	0.2	0.585366	FALSE	285	321
LV_ CO.Lt CO % (GA)[%]	centile	28	0.823529	5.828802	0.294118	0.540928	51	0.864407	4.673718	0.178571	0.584847	FALSE	328	322
LV_ CO/KG.Lt CO/KG % (BPD)[%]	centile	30	0.882353	5.670821	0.470588	0.55408	51	0.864407	4.079998	0.266667	0.584277	FALSE	313	323
RV_ TAPSE septal.TAPSE sept. % (GA)[%]	centile	28	0.823529	6.781968	0.4	0.523166	50	0.847458	3.723863	0.25	0.583405	FALSE	338	324
LV_ Basal-Sep.Wall FS.Lt Basal-Sep FS ZS(BPD)[]	none	30	0.882353	8.232318	0	0.584503	55	0.932203	22.134248	0	0.583392	FALSE	252	325
LV_ EDV.Lt EDV ZS(BPD)[]	none	30	0.882353	2.464324	0	0.656639	51	0.864407	6.819472	0	0.583358	FALSE	120	326
LV_ Basal-Sep.Wall FS.Lt Basal-Sep FS ZS(HC)[]	none	30	0.882353	8.18934	0	0.584705	55	0.932203	23.202031	0	0.582629	FALSE	249	327
LV_ MAPSE septal.MAPSE sept. ZS(GA)[]	none	28	0.823529	5.283566	0	0.575423	51	0.864407	7.095244	0	0.581615	FALSE	276	328
LV_ CO/KG.Lt CO/KG % (GA)[%]	centile	28	0.823529	5.133239	0.4	0.536983	50	0.847458	3.681261	0.285714	0.580604	FALSE	331	329
RV_ ED Area.Rt ED Area ZS(BPD)[]	none	30	0.882353	2.846941	0	0.645155	55	0.932203	31.496199	0	0.578411	FALSE	148	330
RV_ Frac. Area change.Rt FRAC % (GA)[%]	centile	28	0.823529	8.478269	0.235294	0.530437	51	0.864407	4.631092	0.25	0.578238	FALSE	336	331
RV_ Basal-Sep.Wall FS.Rt Basal-Sep FS % (GA)[%]	centile	28	0.823529	5.502102	0.392157	0.534068	51	0.864407	2.810883	0.607143	0.576452	FALSE	332	332

parameter	qc_rule	intra_n_usable	intra_usable_rate	intra_rel_mdc95	intra_max_flip_rate	intra_score	inter_n_usable	inter_usable_rate	inter_rel_mdc95	inter_max_flip_rate	inter_score	possible_copy_forward_inter	intra_rank	inter_rank
RV_ ED Area.Rt ED Area ZS(FL)[]	none	29	0.852941	3.510608	0	0.61515	55	0.932203	40.710364	0	0.575692	FALSE	191	333
RV_ ED Area.Rt ED Area ZS(HC)[]	none	30	0.882353	2.660356	0	0.650455	55	0.932203	64.315889	0	0.572226	FALSE	133	334
LV_ MAPSE septal.MAPSE sept. % (AUA)[%]	centile	30	0.882353	4.774069	0.037037	0.606748	54	0.915254	21.570559	0.033333	0.572016	FALSE	220	335
LV_ Basal-Sep.Wall FS.Lt Basal-Sep FS ZS(AUA)[]	none	30	0.882353	8.312086	0	0.584131	54	0.915254	29.807657	0	0.570611	FALSE	254	336
LV_ MAPSE lateral.MAPSE lat. % (GA)[%]	centile	28	0.823529	3.983019	0.27451	0.564586	51	0.864407	4.643094	0.357143	0.567372	FALSE	288	337
LV_ Basal-Sep.Wall FS.Lt Basal-Sep FS ZS(GA)[]	none	28	0.823529	10.044009	0	0.547983	51	0.864407	15.813823	0	0.555993	FALSE	321	338
RV_ ED Area.Rt ED Area ZS(GA)[]	none	28	0.823529	3.391173	0	0.602857	51	0.864407	21.60015	0	0.549902	FALSE	224	339
LV_ EDV.Lt EDV % (FL)[%]	centile	17	0.5	2.161914	0.242424	0.452263	33	0.559322	1.302014	0.235294	0.529892	FALSE	354	340
LV_ CO.Lt CO ZS(FL)[]	none	17	0.5	2.08941	0	0.479475	33	0.559322	2.215269	0	0.504067	FALSE	346	341
LV_ CO/KG.Lt CO/KG ZS(FL)[]	none	17	0.5	2.143649	0	0.477241	32	0.542373	2.083589	0	0.500905	FALSE	350	342
LV_ EF.Lt EF ZS(FL)[]	none	17	0.5	8.006783	0	0.394411	33	0.559322	2.337167	0	0.499523	FALSE	376	343
LV_ SV.Lt SV ZS(FL)[]	none	17	0.5	2.13539	0	0.477576	33	0.559322	2.707234	0	0.487558	FALSE	349	344
LV_ SV/KG.Lt SV/KG ZS(FL)[]	none	17	0.5	2.128977	0	0.477837	32	0.542373	2.532936	0	0.484407	FALSE	348	345
LV_ EDV.Lt EDV % (AC)[%]	centile	19	0.558824	1.932639	0.178571	0.497951	28	0.474576	1.522026	0.210526	0.474838	FALSE	342	346
LV_ CO.Lt CO ZS (AC)[]	none	19	0.558824	1.664635	0	0.529526	28	0.474576	2.121678	0	0.465424	FALSE	337	347
LV_ CO/KG.Lt CO/KG ZS (AC)[]	none	19	0.558824	1.648136	0	0.530461	27	0.457627	2.001949	0	0.46206	FALSE	335	348
LV_ EF.Lt EF % (FL)[%]	centile	17	0.5	2.314029	0.424242	0.428275	33	0.559322	3.902313	0	0.461255	FALSE	363	349
LV_ SV.Lt SV % (FL)[%]	centile	17	0.5	2.638254	0.30303	0.42964	33	0.559322	2.880432	0.235294	0.459213	FALSE	362	350
LV_ EF.Lt EF ZS (AC)[]	none	19	0.558824	2.348596	0	0.498865	28	0.474576	2.351735	0	0.456629	FALSE	341	351
LV_ SV/KG.Lt SV/KG % (FL)[%]	centile	17	0.5	3.551336	0.375	0.400386	32	0.542373	2.908927	0.176471	0.455869	FALSE	372	352
LV_ SV.Lt SV ZS (AC)[]	none	19	0.558824	1.483867	0	0.540451	28	0.474576	2.485288	0	0.452056	FALSE	329	353
LV_ SV/KG.Lt SV/KG ZS (AC)[]	none	19	0.558824	1.412445	0	0.545219	27	0.457627	2.40731	0	0.446208	FALSE	324	354
LV_ CO.Lt CO % (FL)[%]	centile	17	0.5	3.537292	0.393939	0.398764	33	0.559322	3.24564	0.352941	0.438581	FALSE	373	355
LV_ SV.Lt SV % (AC)[%]	centile	19	0.558824	3.237662	0.357143	0.438089	28	0.474576	2.734845	0.105263	0.433861	FALSE	360	356
LV_ CO/KG.Lt CO/KG % (FL)[%]	centile	17	0.5	4.599418	0.46875	0.374561	32	0.542373	3.38741	0.294118	0.432945	FALSE	381	357
LV_ EDV.Lt EDV ZS(FL)[]	none	17	0.5	3.378167	0	0.441362	33	0.559322	6.593272	0	0.432339	FALSE	357	358
LV_ CO.Lt CO % (AC)[%]	centile	19	0.558824	3.890664	0.25	0.4362	28	0.474576	2.870715	0.105263	0.430102	FALSE	361	359
LV_ SV/KG.Lt SV/KG % (AC)[%]	centile	19	0.558824	4.07421	0.37037	0.421205	27	0.457627	2.675338	0.105263	0.427121	FALSE	366	360
LV_ EDV.Lt EDV ZS (AC)[]	none	19	0.558824	2.561952	0	0.49171	28	0.474576	4.085417	0	0.415944	FALSE	343	361
LV_ EDV.Lt EDV % (EFW)[%]	centile	16	0.470588	1.79075	0.210526	0.457572	19	0.322034	1.26452	0.25	0.412655	FALSE	353	362
LV_ CO/KG.Lt CO/KG % (AC)[%]	centile	19	0.558824	5.260396	0.481481	0.395157	27	0.457627	3.140631	0.157895	0.409628	FALSE	374	363
LV_ EF.Lt EF % (AC)[%]	centile	19	0.558824	3.879409	0.357143	0.425675	28	0.474576	3.859193	0.105263	0.40908	FALSE	364	364
LV_ EF.Lt EF ZS (EFW)[]	none	16	0.470588	2.513639	0	0.449136	19	0.322034	1.838868	0	0.401918	FALSE	355	365
LV_ CO.Lt CO ZS (EFW)[]	none	16	0.470588	2.085946	0	0.464914	19	0.322034	2.028849	0	0.39308	FALSE	352	366
LV_ CO/KG.Lt CO/KG ZS (EFW)[]	none	16	0.470588	1.827104	0	0.476782	19	0.322034	2.087617	0	0.390567	FALSE	351	367
LV_ SV.Lt SV ZS (EFW)[]	none	16	0.470588	1.700225	0	0.48343	19	0.322034	2.381329	0	0.379314	FALSE	345	368
LV_ SV/KG.Lt SV/KG ZS (EFW)[]	none	16	0.470588	1.596401	0	0.489354	19	0.322034	2.434142	0	0.377494	FALSE	344	369
LV_ SV.Lt SV % (EFW)[%]	centile	16	0.470588	2.904775	0.368421	0.400891	19	0.322034	2.259381	0.125	0.37124	FALSE	371	370
LV_ SV/KG.Lt SV/KG % (EFW)[%]	centile	16	0.470588	3.659167	0.263158	0.394831	19	0.322034	2.600366	0.125	0.359617	FALSE	375	371
LV_ EDV.Lt EDV % (HC)[%]	centile	13	0.382353	1.89013	0.285714	0.401007	14	0.237288	1.462994	0.230769	0.357971	FALSE	370	372
LV_ EF.Lt EF ZS(HC)[]	none	13	0.382353	1.625872	0	0.443507	14	0.237288	1.89696	0	0.35672	FALSE	356	373
LV_ CO.Lt CO % (EFW)[%]	centile	16	0.470588	3.697531	0.263158	0.394129	19	0.322034	2.479717	0.25	0.350969	FALSE	377	374
LV_ CO/KG.Lt CO/KG % (EFW)[%]	centile	16	0.470588	4.766732	0.368421	0.367815	19	0.322034	2.966415	0.1875	0.343114	FALSE	382	375
LV_ EF.Lt EF % (EFW)[%]	centile	16	0.470588	3.7089	0.368421	0.383398	19	0.322034	3.769071	0.0625	0.338641	FALSE	380	376
LV_ CO.Lt CO % (HC)[%]	centile	13	0.382353	4.456534	0.357143	0.328769	14	0.237288	1.822234	0.230769	0.337299	FALSE	385	377
LV_ CO/KG.Lt CO/KG ZS(HC)[]	none	13	0.382353	2.196114	0	0.416328	14	0.237288	2.505598	0	0.332747	FALSE	368	378
LV_ SV/KG.Lt SV/KG ZS(HC)[]	none	13	0.382353	1.706578	0	0.438965	14	0.237288	2.723071	0	0.326082	FALSE	358	379
LV_ CO.Lt CO ZS(HC)[]	none	13	0.382353	1.977248	0	0.425529	14	0.237288	2.759188	0	0.32505	FALSE	365	380
LV_ SV/KG.Lt SV/KG % (HC)[%]	centile	13	0.382353	3.527187	0.357143	0.343817	14	0.237288	2.101828	0.230769	0.324523	FALSE	384	381
LV_ SV.Lt SV % (HC)[%]	centile	13	0.382353	4.322692	0.214286	0.344898	14	0.237288	2.281586	0.230769	0.317459	FALSE	383	382
LV_ CO/KG.Lt CO/KG % (HC)[%]	centile	13	0.382353	4.508138	0.357143	0.328082	14	0.237288	2.194811	0.307692	0.313078	FALSE	387	383
LV_ SV.Lt SV ZS(HC)[]	none	13	0.382353	1.707494	0	0.438915	14	0.237288	3.943137	0	0.299564	FALSE	359	384
LV_ EDV.Lt EDV ZS (EFW)[]	none	16	0.470588	4.549055	0	0.407378	19	0.322034	11.180224	0	0.293857	FALSE	369	385
LV_ EF.Lt EF % (HC)[%]	centile	13	0.382353	2.270762	0.285714	0.384901	14	0.237288	3.842809	0.076923	0.293548	FALSE	379	386
LV_ EDV.Lt EDV ZS(HC)[]	none	13	0.382353	9.788871	0	0.328252	14	0.237288	14.951743	0	0.24372	FALSE	386	387

Supplementary File S6

Functional_subset: Functional subset ranked (EF/FRAC/Strain/TAPSE/Volumes/Flow)

parameter	qc_rule	intra_n_usable	intra_usable_rate	intra_rel_mdc95	intra_max_flip_rate	intra_score	inter_n_usable	inter_usable_rate	inter_rel_mdc95	inter_max_flip_rate	inter_score	possible_copy_forward_inter	intra_rank	inter_rank
LV_ EDV[ml]	positive	30	0.882353	1.125847	0	0.729337	56	0.949153	0.615759	0	0.822138	FALSE	62	14
RV_ ED Area.Rt ED Area % (EFW)[%]	centile	30	0.882353	1.671337	0.145455	0.676369	55	0.932203	0.801776	0.166667	0.771438	FALSE	95	19
RV_ Global Strain.Rt GLS %[%]	centile	30	0.882353	0.795571	0.339286	0.730018	56	0.949153	0.865128	0.2	0.769039	FALSE	59	20
RV_ FreeWall Strain.Rt FWS %[%]	centile	30	0.882353	0.760566	0.285714	0.739805	56	0.949153	0.853795	0.266667	0.763683	FALSE	54	21
LV_ FreeWall Strain.Lt FWS %[%]	centile	30	0.882353	0.926004	0.303571	0.718503	56	0.949153	0.899334	0.3	0.755176	FALSE	63	22
LV_ ESV[ml]	positive	30	0.882353	0.929099	0	0.748527	56	0.949153	1.231309	0	0.753843	FALSE	52	23
LV_ Global Strain.Lt GLS %[%]	centile	30	0.882353	0.783748	0.357143	0.729709	56	0.949153	1.098928	0.133333	0.751816	FALSE	60	24
LV_ Sept.Wall Strain.Lt SWS %[%]	centile	30	0.882353	0.707239	0.357143	0.739759	56	0.949153	1.148314	0.166667	0.744102	FALSE	55	26
RV_ Sept.Wall Strain.Rt SWS %[%]	centile	30	0.882353	0.883888	0.214286	0.732075	56	0.949153	1.042182	0.366667	0.733778	FALSE	57	29
LV_ ED Length.Lt ED Length ZS (EFW)[]	none	30	0.882353	1.585392	0	0.695892	55	0.932203	1.612752	0	0.719197	FALSE	76	37
RV_ TAPSE lateral.TAPSE lat. ZS(FL)[]	none	29	0.852941	1.863671	0	0.666151	56	0.949153	1.773326	0	0.718807	FALSE	107	38
RV_ TAPSE lateral.TAPSE lat. ZS (AC)[]	none	30	0.882353	1.907687	0	0.678743	56	0.949153	1.905353	0	0.712253	FALSE	93	41
LV_ ED Area.Lt ED Area % (EFW)[%]	centile	30	0.882353	2.04699	0.109091	0.661544	55	0.932203	1.285842	0.3	0.711092	FALSE	113	42
RV_ TAPSE lateral.TAPSE lat. ZS(BPD)[]	none	30	0.882353	1.8154	0	0.683252	55	0.932203	1.792584	0	0.709338	FALSE	83	43
RV_ TAPSE lateral.TAPSE lat. ZS(HC)[]	none	30	0.882353	1.813092	0	0.683369	55	0.932203	1.818592	0	0.708017	FALSE	82	44
LV_ Frac. Area change.Lt FRAC ZS (EFW)[]	none	30	0.882353	4.417508	0	0.615011	55	0.932203	1.858262	0	0.706047	FALSE	192	46
RV_ TAPSE lateral.TAPSE lat. ZS (EFW)[]	none	30	0.882353	1.883955	0	0.679875	55	0.932203	1.863604	0	0.705786	FALSE	90	47
LV_ Frac. Area change.Lt FRAC ZS(HC)[]	none	30	0.882353	4.461099	0	0.614422	55	0.932203	1.876969	0	0.705137	FALSE	196	48
LV_ Frac. Area change.Lt FRAC ZS (AC)[]	none	30	0.882353	4.441982	0	0.614679	56	0.949153	2.07217	0	0.704777	FALSE	194	49
LV_ Frac. Area change.Lt FRAC ZS(BPD)[]	none	30	0.882353	4.430305	0	0.614837	55	0.932203	1.891523	0	0.704437	FALSE	193	50
RV_ Sept.Wall Strain[%]	strain_raw	22	0.647059	1.484057	0	0.584556	50	0.847458	1.235116	0	0.70269	FALSE	251	52
RV_ Frac. Area change.Rt FRAC ZS (AC)[]	none	30	0.882353	2.044992	0	0.67254	56	0.949153	2.13132	0	0.702318	FALSE	101	53
RV_ TAPSE lateral.TAPSE lat. ZS(AUA)[]	none	30	0.882353	1.856156	0	0.681225	54	0.915254	1.836719	0	0.698635	FALSE	89	59
LV_ Frac. Area change.Lt FRAC ZS(FL)[]	none	29	0.852941	4.420842	0	0.60026	55	0.932203	2.0422	0	0.697586	FALSE	227	61
LV_ EF[%]	percent_0_100	22	0.647059	0.734184	0	0.654185	45	0.762712	0.851033	0	0.697451	FALSE	122	62
LV_ Frac. Area change[%]	percent_0_100	22	0.647059	0.77258	0	0.649189	45	0.762712	0.871831	0	0.69505	FALSE	136	66
RV_ Frac. Area change.Rt FRAC ZS (EFW)[]	none	30	0.882353	2.027562	0	0.673296	55	0.932203	2.103561	0	0.694986	FALSE	99	67
RV_ Frac. Area change.Rt FRAC ZS(HC)[]	none	30	0.882353	2.045597	0	0.672514	55	0.932203	2.106572	0	0.694861	FALSE	102	68
RV_ Frac. Area change.Rt FRAC ZS(BPD)[]	none	30	0.882353	2.046866	0	0.672459	55	0.932203	2.114489	0	0.694534	FALSE	103	69
RV_ Frac. Area change.Rt FRAC ZS(FL)[]	none	29	0.852941	2.064942	0	0.656979	55	0.932203	2.129092	0	0.693934	FALSE	119	71
LV_ MAPSE lateral.MAPSE lat. ZS (EFW)[]	none	30	0.882353	2.562281	0	0.653464	55	0.932203	2.182077	0	0.691806	FALSE	126	73
RV_ Global Strain[%]	strain_raw	22	0.647059	2.187958	0	0.549002	52	0.881356	1.682834	0	0.689774	FALSE	320	79
LV_ Global Strain[%]	strain_raw	23	0.676471	1.066105	0	0.631836	50	0.847458	1.411869	0	0.689575	FALSE	165	80
LV_ Frac. Area change.Lt FRAC ZS(AUA)[]	none	30	0.882353	4.450077	0	0.61457	54	0.915254	2.06267	0	0.688232	FALSE	195	82
RV_ ED Length.Rt ED Length ZS (EFW)[]	none	30	0.882353	1.421576	0	0.706358	55	0.932203	2.300425	0	0.687298	FALSE	69	84
LV_ Basal-Sep.Wall FS.Lt Basal-Sep FS % (EFW)[%]	centile	30	0.882353	1.826696	0.254545	0.65723	55	0.932203	1.646279	0.3	0.687257	FALSE	118	85
RV_ FreeWall Strain[%]	strain_raw	22	0.647059	1.855231	0	0.563623	50	0.847458	1.455728	0	0.686613	FALSE	290	86
RV_ Frac. Area change.Rt FRAC ZS(AUA)[]	none	30	0.882353	2.031495	0	0.673125	54	0.915254	2.110648	0	0.686218	FALSE	100	87
RV_ Basal-Lat.Wall FS.Rt Basal-Lat FS ZS (EFW)[]	none	30	0.882353	2.632389	0	0.651297	55	0.932203	2.369338	0	0.684819	FALSE	130	88
LV_ EF.Lt EF ZS(AUA)[]	none	30	0.882353	7.684258	0	0.587237	54	0.915254	2.227835	0	0.681549	FALSE	243	93
LV_ FreeWall Strain[%]	strain_raw	23	0.676471	1.365925	0	0.607302	50	0.847458	1.623798	0	0.67618	FALSE	218	102
RV_ TAPSE lateral.TAPSE lat. ZS(GA)[]	none	28	0.823529	1.733639	0	0.65809	51	0.864407	1.799215	0	0.675101	FALSE	116	105
RV_ ED Length.Rt ED Length % (EFW)[%]	centile	30	0.882353	2.792033	0.236364	0.623024	55	0.932203	1.887651	0.3	0.674623	FALSE	177	107
LV_ Sept.Wall Strain[%]	strain_raw	24	0.705882	1.207653	0	0.634129	48	0.813559	1.384818	0	0.674507	FALSE	155	124

parameter	qc_rule	intra_n_usable	intra_usable_rate	intra_rel_mdc95	intra_max_flip_rate	intra_score	inter_n_usable	inter_usable_rate	inter_rel_mdc95	inter_max_flip_rate	inter_score	possible_copy_forward_inter	intra_rank	inter_rank
RV_ TAPSE septal.TAPSE sept. ZS(FL)[]	none	29	0.852941	2.727612	0	0.633778	55	0.932203	2.77582	0	0.672039	FALSE	157	126
LV_ MAPSE septal.MAPSE sept. % (EFW)[%]	centile	30	0.882353	2.545367	0.218182	0.632182	55	0.932203	1.857063	0.366667	0.669439	FALSE	163	136
LV_ ED Length.Lt ED Length % (EFW)[%]	centile	30	0.882353	3.011302	0.272727	0.613622	55	0.932203	1.855342	0.4	0.66619	FALSE	199	141
4CV Circ (2Dist).4CV C % (EFW)[%]	centile	30	0.882353	0.226163	0	0.867397	55	0.932203	0	0	0.666102	TRUE	10	142
4CV Circ (2Dist).4CV C ZS (EFW)[]	none	30	0.882353	0.889339	0	0.752891	55	0.932203	0	0	0.666102	TRUE	50	142
4CV Trv. Width ED.4CV TW ED ZS (EFW)[]	none	30	0.882353	4.078418	0	0.619941	55	0.932203	0	0	0.666102	TRUE	181	142
4CV Area (2Dist).4CV A ZS (EFW)[]	none	30	0.882353	0.684872	0	0.778583	55	0.932203	0	0	0.666102	TRUE	42	142
4CV Length ED.4CV L ED ZS (EFW)[]	none	30	0.882353	1.007234	0	0.740456	55	0.932203	0	0	0.666102	TRUE	53	142
4CV Area (2Dist).4CV A % (EFW)[%]	centile	30	0.882353	0.20025	0	0.87444	55	0.932203	0	0	0.666102	TRUE	7	142
RV_ TAPSE septal.TAPSE sept. ZS (AC)[]	none	30	0.882353	2.821843	0	0.645838	55	0.932203	3.002055	0	0.66605	FALSE	146	157
LV_ EF.Lt EF ZS(BPD)[]	none	30	0.882353	7.726619	0	0.587013	51	0.864407	2.038448	0	0.66385	FALSE	244	161
RV_ TAPSE septal.TAPSE sept. ZS(BPD)[]	none	30	0.882353	2.661065	0	0.650434	54	0.915254	2.776163	0	0.663555	FALSE	134	162
LV_ Frac. Area change.Lt FRAC ZS(GA)[]	none	28	0.823529	4.849648	0	0.580145	51	0.864407	2.055004	0	0.663136	FALSE	264	163
4CV Trv. Width ED.4CV TW ED % (EFW)[%]	centile	30	0.882353	0.36621	0	0.833957	55	0.932203	0	0.033333	0.662768	TRUE	25	164
4CV Length ED.4CV L ED % (EFW)[%]	centile	30	0.882353	0.304642	0	0.847774	55	0.932203	0	0.033333	0.662768	TRUE	18	164
LV_ EDV.Lt EDV % (AUA)[%]	centile	30	0.882353	2.701629	0.259259	0.623311	54	0.915254	2.037368	0.266667	0.662653	FALSE	175	173
RV_ Frac. Area change.Rt FRAC ZS(GA)[]	none	28	0.823529	2.021143	0	0.644165	51	0.864407	2.076052	0	0.66224	FALSE	149	174
RV_ TAPSE septal.TAPSE sept. ZS(HC)[]	none	30	0.882353	2.731007	0	0.648386	54	0.915254	2.847635	0	0.661587	FALSE	139	177
RV_ Frac. Area change[%]	percent_0_100	18	0.529412	1.195642	0	0.546885	43	0.728814	1.037268	0	0.660748	FALSE	322	178
LV_ EDV.Lt EDV % (GA)[%]	centile	28	0.823529	2.370193	0.176471	0.612805	51	0.864407	1.610269	0.25	0.660444	FALSE	201	179
RV_ TAPSE septal.TAPSE sept. ZS (EFW)[]	none	30	0.882353	2.833417	0	0.645522	54	0.915254	2.944817	0	0.659026	FALSE	147	183
LV_ CO[ml/min]	positive	22	0.647059	1.892895	0	0.561799	45	0.762712	1.251453	0	0.659019	FALSE	292	184
LV_ EDV.Lt EDV % (BPD)[%]	centile	30	0.882353	2.344819	0.215686	0.639196	51	0.864407	1.726357	0.233333	0.655586	FALSE	152	188
LV_ EDV.Lt EDV ZS(AUA)[]	none	30	0.882353	2.094675	0	0.670431	54	0.915254	3.086429	0	0.655512	FALSE	104	189
LV_ EF.Lt EF ZS(GA)[]	none	28	0.823529	8.268992	0	0.554919	51	0.864407	2.257478	0	0.654998	FALSE	310	191
RV_ Basal-Sep.Wall FS.Rt Basal-Sep FS ZS (EFW)[]	none	30	0.882353	3.329678	0	0.633562	55	0.932203	3.526862	0	0.654463	FALSE	158	193
LV_ Basal-Lat.Wall FS.Lt Basal-Lat FS % (EFW)[%]	centile	30	0.882353	2.726941	0.327273	0.615776	55	0.932203	2.642561	0.233333	0.652581	FALSE	189	201
LV_ SV[ml]	positive	22	0.647059	1.701264	0	0.571608	45	0.762712	1.339709	0	0.652317	FALSE	284	203
RV_ TAPSE septal.TAPSE sept. ZS(AUA)[]	none	30	0.882353	2.755293	0	0.647693	53	0.898305	2.98251	0	0.649592	FALSE	142	207
LV_ FreeWall Strain.Lt FWS ZS[]	zscore	27	0.794118	2.293432	0.4	0.578513	55	0.932203	2.429363	0.333333	0.649408	FALSE	270	208
RV_ TAPSE lateral[mm]	tapse_mm	16	0.470588	1.036064	0	0.531752	45	0.762712	1.435362	0	0.645603	FALSE	334	215
LV_ Basal-Lat.Wall FS.Lt Basal-Lat FS ZS (EFW)[]	none	30	0.882353	4.989465	0	0.60796	55	0.932203	4.047799	0	0.645344	FALSE	215	217
RV_ Basal-Lat.Wall FS.Rt Basal-Lat FS % (EFW)[%]	centile	30	0.882353	4.415979	0.327273	0.582305	55	0.932203	2.969219	0.233333	0.643544	FALSE	260	220
RV_ TAPSE lateral.TAPSE lat. % (AC)[%]	centile	30	0.882353	4.909959	0.303571	0.578502	56	0.949153	3.41473	0.266667	0.638515	FALSE	271	230
LV_ Frac. Area change.Lt FRAC % (AC)[%]	centile	30	0.882353	4.090097	0.410714	0.578689	56	0.949153	4.016266	0.166667	0.63765	FALSE	269	232
LV_ ED Area.Lt ED Area ZS (EFW)[]	none	30	0.882353	2.679959	0	0.649873	55	0.932203	4.707186	0	0.636189	FALSE	135	234
RV_ TAPSE lateral.TAPSE lat. % (FL)[%]	centile	29	0.852941	5.350686	0.285714	0.560885	56	0.949153	3.566239	0.275862	0.63459	FALSE	294	235
LV_ Frac. Area change.Lt FRAC % (HC)[%]	centile	30	0.882353	4.131074	0.345455	0.584587	55	0.932203	4.078665	0.133333	0.631529	FALSE	250	241
RV_ TAPSE lateral.TAPSE lat. % (EFW)[%]	centile	30	0.882353	5.134868	0.327273	0.57365	55	0.932203	3.381649	0.266667	0.630725	FALSE	283	242
LV_ Frac. Area change.Lt FRAC % (FL)[%]	centile	29	0.852941	2.904852	0.309091	0.597998	55	0.932203	4.109631	0.137931	0.630592	FALSE	229	243
LV_ Sept.Wall Strain.Lt SWS ZS[]	zscore	29	0.852941	2.899912	0.482143	0.580823	56	0.949153	3.61159	0.310345	0.63028	FALSE	263	244
RV_ TAPSE septal.TAPSE sept. % (AC)[%]	centile	30	0.882353	6.556734	0.436364	0.550473	55	0.932203	3.621573	0.233333	0.629319	FALSE	317	245
RV_ TAPSE septal.TAPSE sept. % (FL)[%]	centile	29	0.852941	6.824102	0.4	0.537595	55	0.932203	3.791085	0.206897	0.6289	FALSE	330	246
RV_ TAPSE lateral.TAPSE lat. % (HC)[%]	centile	30	0.882353	5.756856	0.254545	0.574921	55	0.932203	3.483562	0.266667	0.62865	FALSE	278	247
RV_ Global Strain.Rt GLS ZS[]	zscore	25	0.735294	2.109592	0.415094	0.554772	53	0.898305	2.881821	0.24	0.628197	FALSE	312	250
LV_ Frac. Area change.Lt FRAC % (BPD)[%]	centile	30	0.882353	4.159512	0.345455	0.584158	55	0.932203	4.092784	0.166667	0.627977	FALSE	253	251
RV_ TAPSE lateral.TAPSE lat. % (BPD)[%]	centile	30	0.882353	5.564463	0.272727	0.574838	55	0.932203	3.545102	0.266667	0.627442	FALSE	280	253

parameter	qc_rule	intra_n_usable	intra_usable_rate	intra_rel_mdc95	intra_max_flip_rate	intra_score	inter_n_usable	inter_usable_rate	inter_rel_mdc95	inter_max_flip_rate	inter_score	possible_copy_forward_inter	intra_rank	inter_rank
RV_ FreeWall Strain.Rt FWS ZS[]	zscore	25	0.735294	2.108065	0.415094	0.554835	53	0.898305	2.927239	0.24	0.627005	FALSE	311	254
RV_ TAPSE septal.TAPSE sept. ZS(GA)[]	none	28	0.823529	2.836431	0	0.616028	50	0.847458	3.005963	0	0.62358	FALSE	187	258
RV_ Sept.Wall Strain.Rt SWS ZS[]	zscore	29	0.852941	3.228488	0.321429	0.588924	56	0.949153	3.796995	0.344828	0.623479	FALSE	240	259
RV_ TAPSE lateral.TAPSE lat. % (AUA)[%]	centile	30	0.882353	5.462842	0.259259	0.577143	54	0.915254	3.339915	0.266667	0.623128	FALSE	273	260
LV_ Frac. Area change.Lt FRAC % (EFW)[%]	centile	30	0.882353	4.223665	0.272727	0.590478	55	0.932203	4.202984	0.2	0.622981	FALSE	238	261
RV_ TAPSE septal.TAPSE sept. % (HC)[%]	centile	30	0.882353	6.788523	0.388889	0.553645	54	0.915254	3.746662	0.233333	0.618564	FALSE	314	268
RV_ Frac. Area change.Rt FRAC % (AC)[%]	centile	30	0.882353	9.033657	0.232143	0.557828	56	0.949153	4.986756	0.233333	0.618057	FALSE	299	269
RV_ TAPSE septal.TAPSE sept. % (EFW)[%]	centile	30	0.882353	6.568931	0.407407	0.553283	54	0.915254	3.59323	0.266667	0.618045	FALSE	315	270
LV_ Frac. Area change.Lt FRAC % (AUA)[%]	centile	30	0.882353	4.092111	0.314815	0.588248	54	0.915254	4.201525	0.166667	0.617861	FALSE	241	271
LV_ MAPSE lateral.MAPSE lat. % (EFW)[%]	centile	30	0.882353	3.85491	0.327273	0.59084	55	0.932203	3.798351	0.333333	0.61613	FALSE	237	274
RV_ TAPSE septal.TAPSE sept. % (AUA)[%]	centile	30	0.882353	6.69166	0.377358	0.555445	53	0.898305	3.609237	0.2	0.615935	FALSE	308	275
RV_ TAPSE septal.TAPSE sept. % (BPD)[%]	centile	30	0.882353	6.791665	0.37037	0.555476	54	0.915254	3.929967	0.233333	0.61543	FALSE	307	276
LV_ MAPSE septal.MAPSE sept. ZS (EFW)[]	none	30	0.882353	4.744144	0	0.610813	55	0.932203	7.164928	0	0.615092	FALSE	207	278
RV_ ED Area.Rt ED Area ZS (EFW)[]	none	30	0.882353	5.44323	0	0.603257	55	0.932203	7.484209	0	0.613248	FALSE	223	281
LV_ EF.Lt EF % (AUA)[%]	centile	30	0.882353	4.409205	0.314815	0.583643	54	0.915254	4.361662	0.2	0.612231	FALSE	256	283
RV_ Basal-Sep.Wall FS.Rt Basal-Sep FS % (EFW)[%]	centile	30	0.882353	5.78741	0.418182	0.558291	55	0.932203	2.933188	0.566667	0.611134	FALSE	297	287
RV_ Frac. Area change.Rt FRAC % (EFW)[%]	centile	30	0.882353	9.154705	0.236364	0.556931	55	0.932203	4.99483	0.233333	0.609493	FALSE	303	288
RV_ Frac. Area change.Rt FRAC % (BPD)[%]	centile	30	0.882353	9.140413	0.236364	0.556986	55	0.932203	5.022526	0.233333	0.609186	FALSE	302	290
RV_ Frac. Area change.Rt FRAC % (HC)[%]	centile	30	0.882353	9.191185	0.236364	0.55679	55	0.932203	5.136417	0.233333	0.607953	FALSE	304	294
RV_ Frac. Area change.Rt FRAC % (FL)[%]	centile	29	0.852941	9.078901	0.236364	0.542521	55	0.932203	5.066263	0.241379	0.607902	FALSE	326	295
LV_ EDV.Lt EDV ZS(GA)[]	none	28	0.823529	1.765398	0	0.656409	51	0.864407	4.401962	0	0.606251	FALSE	121	299
RV_ Frac. Area change.Rt FRAC % (AUA)[%]	centile	30	0.882353	9.248791	0.222222	0.557983	54	0.915254	5.057606	0.233333	0.600327	FALSE	298	306
LV_ EF.Lt EF % (GA)[%]	centile	28	0.823529	4.443283	0.333333	0.551916	51	0.864407	3.843074	0.178571	0.596938	FALSE	316	309
LV_ Frac. Area change.Lt FRAC % (GA)[%]	centile	28	0.823529	4.142355	0.392157	0.550334	51	0.864407	3.860091	0.178571	0.596649	FALSE	318	310
RV_ TAPSE septal[mm]	tapse_mm	12	0.352941	2.424018	0	0.393292	39	0.661017	1.441278	0	0.594357	FALSE	378	311
LV_ Basal-Sep.Wall FS.Lt Basal-Sep FS ZS (EFW)[]	none	30	0.882353	7.674802	0	0.587287	55	0.932203	14.378863	0	0.592111	FALSE	242	312
RV_ TAPSE lateral.TAPSE lat. % (GA)[%]	centile	28	0.823529	5.576275	0.313725	0.541217	51	0.864407	3.737174	0.25	0.591642	FALSE	327	313
LV_ Global Strain.Lt GLS ZS[]	zscore	24	0.705882	2.212906	0.54902	0.522537	51	0.864407	3.974378	0.25	0.587615	FALSE	339	318
LV_ EF.Lt EF % (BPD)[%]	centile	30	0.882353	4.560669	0.27451	0.585659	51	0.864407	4.45552	0.2	0.585524	FALSE	248	320
RV_ TAPSE septal.TAPSE sept. % (GA)[%]	centile	28	0.823529	6.781968	0.4	0.523166	50	0.847458	3.723863	0.25	0.583405	FALSE	338	324
LV_ EDV.Lt EDV ZS(BPD)[]	none	30	0.882353	2.464324	0	0.656639	51	0.864407	6.819472	0	0.583358	FALSE	120	326
RV_ Frac. Area change.Rt FRAC % (GA)[%]	centile	28	0.823529	8.478269	0.235294	0.530437	51	0.864407	4.631092	0.25	0.578238	FALSE	336	331
LV_ EDV.Lt EDV % (FL)[%]	centile	17	0.5	2.161914	0.242424	0.452263	33	0.559322	1.302014	0.235294	0.529892	FALSE	354	340
LV_ EF.Lt EF ZS(FL)[]	none	17	0.5	8.006783	0	0.394411	33	0.559322	2.337167	0	0.499523	FALSE	376	343
LV_ EDV.Lt EDV % (AC)[%]	centile	19	0.558824	1.932639	0.178571	0.497951	28	0.474576	1.522026	0.210526	0.474838	FALSE	342	346
LV_ EF.Lt EF % (FL)[%]	centile	17	0.5	2.314029	0.424242	0.428275	33	0.559322	3.902313	0	0.461255	FALSE	363	349
LV_ EF.Lt EF ZS (AC)[]	none	19	0.558824	2.348596	0	0.498865	28	0.474576	2.351735	0	0.456629	FALSE	341	351
LV_ EDV.Lt EDV ZS(FL)[]	none	17	0.5	3.378167	0	0.441362	33	0.559322	6.593272	0	0.432339	FALSE	357	358
LV_ EDV.Lt EDV ZS (AC)[]	none	19	0.558824	2.561952	0	0.49171	28	0.474576	4.085417	0	0.415944	FALSE	343	361
LV_ EDV.Lt EDV % (EFW)[%]	centile	16	0.470588	1.79075	0.210526	0.457572	19	0.322034	1.26452	0.25	0.412655	FALSE	353	362
LV_ EF.Lt EF % (AC)[%]	centile	19	0.558824	3.879409	0.357143	0.425675	28	0.474576	3.859193	0.105263	0.40908	FALSE	364	364
LV_ EF.Lt EF ZS (EFW)[]	none	16	0.470588	2.513639	0	0.449136	19	0.322034	1.838868	0	0.401918	FALSE	355	365
LV_ CO.Lt CO ZS (EFW)[]	none	16	0.470588	2.085946	0	0.464914	19	0.322034	2.028849	0	0.39308	FALSE	352	366
LV_ CO/KG.Lt CO/KG ZS (EFW)[]	none	16	0.470588	1.827104	0	0.476782	19	0.322034	2.087617	0	0.390567	FALSE	351	367
LV_ SV.Lt SV ZS (EFW)[]	none	16	0.470588	1.700225	0	0.48343	19	0.322034	2.381329	0	0.379314	FALSE	345	368
LV_ SV/KG.Lt SV/KG ZS (EFW)[]	none	16	0.470588	1.596401	0	0.489354	19	0.322034	2.434142	0	0.377494	FALSE	344	369
LV_ SV.Lt SV % (EFW)[%]	centile	16	0.470588	2.904775	0.368421	0.400891	19	0.322034	2.259381	0.125	0.37124	FALSE	371	370
LV_ SV/KG.Lt SV/KG % (EFW)[%]	centile	16	0.470588	3.659167	0.263158	0.394831	19	0.322034	2.600366	0.125	0.359617	FALSE	375	371
LV_ EDV.Lt EDV % (HC)[%]	centile	13	0.382353	1.89013	0.285714	0.401007	14	0.237288	1.462994	0.230769	0.357971	FALSE	370	372
LV_ EF.Lt EF ZS(HC)[]	none	13	0.382353	1.625872	0	0.443507	14	0.237288	1.89696	0	0.35672	FALSE	356	373
LV_ CO.Lt CO % (EFW)[%]	centile	16	0.470588	3.697531	0.263158	0.394129	19	0.322034	2.479717	0.25	0.350969	FALSE	377	374

parameter	qc_rule	intra_n_usable	intra_usable_rate	intra_rel_mdc95	intra_max_flip_rate	intra_score	inter_n_usable	inter_usable_rate	inter_rel_mdc95	inter_max_flip_rate	inter_score	possible_copy_forward_inter	intra_rank	inter_rank
LV_ CO/KG.Lt CO/KG % (EFW)[%]	centile	16	0.470588	4.766732	0.368421	0.367815	19	0.322034	2.966415	0.1875	0.343114	FALSE	382	375
LV_ EF.Lt EF % (EFW)[%]	centile	16	0.470588	3.7089	0.368421	0.383398	19	0.322034	3.769071	0.0625	0.338641	FALSE	380	376
LV_ EDV.Lt EDV ZS (EFW)[]	none	16	0.470588	4.549055	0	0.407378	19	0.322034	11.180224	0	0.293857	FALSE	369	385
LV_ EF.Lt EF % (HC)[%]	centile	13	0.382353	2.270762	0.285714	0.384901	14	0.237288	3.842809	0.076923	0.293548	FALSE	379	386
LV_ EDV.Lt EDV ZS(HC)[]	none	13	0.382353	9.788871	0	0.328252	14	0.237288	14.951743	0	0.24372	FALSE	386	387

Supplementary File S6

Intra_Top50: Intra Top50

parameter	qc_rule	intra_n_usable	intra_usable_rate	intra_rel_mdc95	intra_max_flip_rate	intra_score	inter_n_usable	inter_usable_rate	inter_rel_mdc95	inter_max_flip_rate	inter_score	possible_copy_forward_inter	intra_rank	inter_rank
4CV Trv. Width ED[mm]	positive	30	0.882353	0.039218	0	0.926081	56	0.949153	0	0	0.674576	TRUE	1	108
4CV Circ (2Dist).4CV C % (AC)[%]	centile	30	0.882353	0.045903	0	0.923621	56	0.949153	0	0	0.674576	TRUE	2	108
4CV Circ (2Dist)[mm]	positive	30	0.882353	0.056392	0	0.919824	56	0.949153	0	0	0.674576	TRUE	3	108
4CV Length ED[mm]	positive	30	0.882353	0.079295	0	0.911789	56	0.949153	0	0	0.674576	TRUE	4	108
4CV Area (2Dist)[mm¸]	none	30	0.882353	0.104454	0	0.903346	56	0.949153	0	0	0.674576	TRUE	5	108
4CV Area (2Dist).4CV A % (AC)[%]	centile	30	0.882353	0.189421	0	0.877474	56	0.949153	0	0	0.674576	TRUE	6	108
4CV Area (2Dist).4CV A % (EFW)[%]	centile	30	0.882353	0.20025	0	0.87444	55	0.932203	0	0	0.666102	TRUE	7	142
4CV Length ED.4CV L ED % (GA)[%]	centile	28	0.823529	0.108445	0	0.872631	51	0.864407	0	0.035714	0.628632	TRUE	8	248
4CV Circ (2Dist).4CV C % (HC)[%]	centile	30	0.882353	0.222098	0	0.868482	55	0.932203	0	0	0.666102	TRUE	9	142
4CV Circ (2Dist).4CV C % (EFW)[%]	centile	30	0.882353	0.226163	0	0.867397	55	0.932203	0	0	0.666102	TRUE	10	142
4CV Trv. Width ED.4CV TW ED % (AC)[%]	centile	30	0.882353	0.24131	0	0.863417	56	0.949153	0	0	0.674576	TRUE	11	108
4CV Length ED.4CV L ED % (BPD)[%]	centile	30	0.882353	0.25373	0	0.860224	55	0.932203	0	0.033333	0.662768	TRUE	12	164
4CV Area (2Dist).4CV A % (HC)[%]	centile	30	0.882353	0.278282	0	0.854097	55	0.932203	0	0.033333	0.662768	TRUE	13	164
4CV Area (2Dist).4CV A % (BPD)[%]	centile	30	0.882353	0.29097	0	0.851021	55	0.932203	0	0.033333	0.662768	TRUE	14	164
4CV Circ (2Dist).4CV C % (AUA)[%]	centile	30	0.882353	0.293899	0	0.85032	54	0.915254	0.006128	0.033333	0.951858	FALSE	15	2
4CV Length ED.4CV L ED % (AUA)[%]	centile	30	0.882353	0.292921	0.018519	0.848702	54	0.915254	0.011686	0.033333	0.949674	FALSE	16	3
4CV Area (2Dist).4CV A % (AUA)[%]	centile	30	0.882353	0.303273	0	0.848096	54	0.915254	0.002829	0.033333	0.953166	FALSE	17	1
4CV Length ED.4CV L ED % (EFW)[%]	centile	30	0.882353	0.304642	0	0.847774	55	0.932203	0	0.033333	0.662768	TRUE	18	164
4CV Length ED.4CV L ED % (AC)[%]	centile	30	0.882353	0.316738	0	0.844957	56	0.949153	0	0	0.674576	TRUE	19	108
LV_ ES Length[cm]	positive	30	0.882353	0.333237	0	0.841198	56	0.949153	0.377065	0	0.865049	FALSE	20	6
4CV Length ED.4CV L ED % (HC)[%]	centile	30	0.882353	0.338164	0	0.840094	55	0.932203	0	0.033333	0.662768	TRUE	21	164
4CV Circ (2Dist).4CV C % (GA)[%]	centile	28	0.823529	0.218699	0	0.839984	51	0.864407	0	0	0.632203	TRUE	22	237
4CV Circ (2Dist).4CV C % (BPD)[%]	centile	30	0.882353	0.346481	0	0.838247	55	0.932203	0	0.033333	0.662768	TRUE	23	164
4CV Trv. Width ED.4CV TW ED % (HC)[%]	centile	30	0.882353	0.35845	0	0.83563	55	0.932203	0	0.033333	0.662768	TRUE	24	164
4CV Trv. Width ED.4CV TW ED % (EFW)[%]	centile	30	0.882353	0.36621	0	0.833957	55	0.932203	0	0.033333	0.662768	TRUE	25	164
4CV Trv. Width ED.4CV TW ED % (BPD)[%]	centile	30	0.882353	0.372159	0	0.832688	55	0.932203	0	0.033333	0.662768	TRUE	26	164
4CV Trv. Width ED.4CV TW ED % (AUA)[%]	centile	30	0.882353	0.378531	0	0.83134	54	0.915254	0.012515	0.033333	0.94935	FALSE	27	4
LV_ ED Length[cm]	positive	30	0.882353	0.407216	0	0.825426	56	0.949153	0.317558	0	0.878168	FALSE	28	5
RV_ ED Length[cm]	positive	30	0.882353	0.429296	0	0.821034	56	0.949153	0.406536	0	0.858963	FALSE	29	7
4CV Length ED.4CV L ED % (FL)[%]	centile	29	0.852941	0.377314	0	0.816891	56	0.949153	0	0.034483	0.671128	TRUE	30	129
4CV Trv. Width ED.4CV TW ED % (GA)[%]	centile	28	0.823529	0.317619	0	0.815343	51	0.864407	0	0.035714	0.628632	TRUE	31	248
4CV Circ (2Dist).4CV C ZS(HC)[]	none	30	0.882353	0.510102	0	0.806059	55	0.932203	0	0	0.666102	TRUE	32	142
4CV Circ (2Dist).4CV C % (FL)[%]	centile	29	0.852941	0.43605	0	0.805012	56	0.949153	0	0.034483	0.671128	TRUE	33	129
RV_ ES Length[cm]	positive	30	0.882353	0.521714	0	0.804038	56	0.949153	0.408071	0	0.858653	FALSE	34	8
LV_ ES Area[cm¸]	none	30	0.882353	0.524116	0	0.803624	56	0.949153	0.721839	0	0.806886	FALSE	35	16
4CV Area (2Dist).4CV A % (FL)[%]	centile	29	0.852941	0.445525	0	0.803187	56	0.949153	0	0.034483	0.671128	TRUE	36	129
4CV Trv. Width ED.4CV TW ED % (FL)[%]	centile	29	0.852941	0.459742	0	0.800492	56	0.949153	0	0.034483	0.671128	TRUE	37	129
LV_ ED Area[cm¸]	none	30	0.882353	0.589294	0	0.792861	56	0.949153	0.427659	0	0.854755	FALSE	38	9
4CV Area (2Dist).4CV A ZS(HC)[]	none	30	0.882353	0.595428	0	0.791893	55	0.932203	0	0	0.666102	TRUE	39	142
4CV Circ (2Dist).4CV C ZS(GA)[]	none	28	0.823529	0.446866	0	0.788224	51	0.864407	0	0	0.632203	TRUE	40	237

parameter	qc_rule	intra_n_usable	intra_usable_rate	intra_rel_mdc95	intra_max_flip_rate	intra_score	inter_n_usable	inter_usable_rate	inter_rel_mdc95	inter_max_flip_rate	inter_score	possible_copy_forward_inter	intra_rank	inter_rank
4CV Area (2Dist).4CV A ZS(BPD)[]	none	30	0.882353	0.653458	0	0.783094	55	0.932203	0	0	0.666102	TRUE	41	142
4CV Area (2Dist).4CV A ZS (EFW)[]	none	30	0.882353	0.684872	0	0.778583	55	0.932203	0	0	0.666102	TRUE	42	142
LV_ MAPSE septal.MAPSE sept. ZS(AUA)[]	none	30	0.882353	0.688301	0	0.778101	54	0.915254	0.529221	0	0.819198	FALSE	43	15
RV_ ES Area[cm¸]	none	30	0.882353	0.747478	0	0.770078	56	0.949153	0.566861	0	0.829864	FALSE	44	13
4CV Length ED.4CV L ED ZS(GA)[]	none	28	0.823529	0.571981	0	0.766221	51	0.864407	0	0	0.632203	TRUE	45	237
RV_ ED Area[cm¸]	none	30	0.882353	0.781799	0	0.765669	56	0.949153	0.45648	0	0.849211	FALSE	46	10
4CV Length ED.4CV L ED ZS(BPD)[]	none	30	0.882353	0.837321	0	0.758885	55	0.932203	0	0	0.666102	TRUE	47	142
4CV Circ (2Dist).4CV C ZS(BPD)[]	none	30	0.882353	0.859313	0	0.75631	55	0.932203	0	0	0.666102	TRUE	48	142
4CV Length ED.4CV L ED ZS(HC)[]	none	30	0.882353	0.871595	0	0.754898	55	0.932203	0	0	0.666102	TRUE	49	142
4CV Circ (2Dist).4CV C ZS (EFW)[]	none	30	0.882353	0.889339	0	0.752891	55	0.932203	0	0	0.666102	TRUE	50	142

Supplementary File S6

Intra_Bottom50: Intra Bottom50

parameter	qc_rule	intra_n_usable	intra_usable_rate	intra_rel_mdc95	intra_max_flip_rate	intra_score	inter_n_usable	inter_usable_rate	inter_rel_mdc95	inter_max_flip_rate	inter_score	possible_copy_forward_inter	intra_rank	inter_rank
LV_ CO/KG.Lt CO/KG % (HC)[%]	centile	13	0.382353	4.508138	0.357143	0.328082	14	0.237288	2.194811	0.307692	0.313078	FALSE	387	383
LV_ EDV.Lt EDV ZS(HC)[]	none	13	0.382353	9.788871	0	0.328252	14	0.237288	14.951743	0	0.24372	FALSE	386	387
LV_ CO.Lt CO % (HC)[%]	centile	13	0.382353	4.456534	0.357143	0.328769	14	0.237288	1.822234	0.230769	0.337299	FALSE	385	377
LV_ SV/KG.Lt SV/KG % (HC)[%]	centile	13	0.382353	3.527187	0.357143	0.343817	14	0.237288	2.101828	0.230769	0.324523	FALSE	384	381
LV_ SV.Lt SV % (HC)[%]	centile	13	0.382353	4.322692	0.214286	0.344898	14	0.237288	2.281586	0.230769	0.317459	FALSE	383	382
LV_ CO/KG.Lt CO/KG % (EFW)[%]	centile	16	0.470588	4.766732	0.368421	0.367815	19	0.322034	2.966415	0.1875	0.343114	FALSE	382	375
LV_ CO/KG.Lt CO/KG % (FL)[%]	centile	17	0.5	4.599418	0.46875	0.374561	32	0.542373	3.38741	0.294118	0.432945	FALSE	381	357
LV_ EF.Lt EF % (EFW)[%]	centile	16	0.470588	3.7089	0.368421	0.383398	19	0.322034	3.769071	0.0625	0.338641	FALSE	380	376
LV_ EF.Lt EF % (HC)[%]	centile	13	0.382353	2.270762	0.285714	0.384901	14	0.237288	3.842809	0.076923	0.293548	FALSE	379	386
RV_ TAPSE septal[mm]	tapse_mm	12	0.352941	2.424018	0	0.393292	39	0.661017	1.441278	0	0.594357	FALSE	378	311
LV_ CO.Lt CO % (EFW)[%]	centile	16	0.470588	3.697531	0.263158	0.394129	19	0.322034	2.479717	0.25	0.350969	FALSE	377	374
LV_ EF.Lt EF ZS(FL)[]	none	17	0.5	8.006783	0	0.394411	33	0.559322	2.337167	0	0.499523	FALSE	376	343
LV_ SV/KG.Lt SV/KG % (EFW)[%]	centile	16	0.470588	3.659167	0.263158	0.394831	19	0.322034	2.600366	0.125	0.359617	FALSE	375	371
LV_ CO/KG.Lt CO/KG % (AC)[%]	centile	19	0.558824	5.260396	0.481481	0.395157	27	0.457627	3.140631	0.157895	0.409628	FALSE	374	363
LV_ CO.Lt CO % (FL)[%]	centile	17	0.5	3.537292	0.393939	0.398764	33	0.559322	3.24564	0.352941	0.438581	FALSE	373	355
LV_ SV/KG.Lt SV/KG % (FL)[%]	centile	17	0.5	3.551336	0.375	0.400386	32	0.542373	2.908927	0.176471	0.455869	FALSE	372	352
LV_ SV.Lt SV % (EFW)[%]	centile	16	0.470588	2.904775	0.368421	0.400891	19	0.322034	2.259381	0.125	0.37124	FALSE	371	370
LV_ EDV.Lt EDV % (HC)[%]	centile	13	0.382353	1.89013	0.285714	0.401007	14	0.237288	1.462994	0.230769	0.357971	FALSE	370	372
LV_ EDV.Lt EDV ZS (EFW)[]	none	16	0.470588	4.549055	0	0.407378	19	0.322034	11.180224	0	0.293857	FALSE	369	385
LV_ CO/KG.Lt CO/KG ZS(HC)[]	none	13	0.382353	2.196114	0	0.416328	14	0.237288	2.505598	0	0.332747	FALSE	368	378
RV_ Basal-Sep.Wall FS[%]	percent_0_100	14	0.411765	2.604507	0	0.416855	40	0.677966	1.297066	0	0.613118	FALSE	367	282
LV_ SV/KG.Lt SV/KG % (AC)[%]	centile	19	0.558824	4.07421	0.37037	0.421205	27	0.457627	2.675338	0.105263	0.427121	FALSE	366	360
LV_ CO.Lt CO ZS(HC)[]	none	13	0.382353	1.977248	0	0.425529	14	0.237288	2.759188	0	0.32505	FALSE	365	380
LV_ EF.Lt EF % (AC)[%]	centile	19	0.558824	3.879409	0.357143	0.425675	28	0.474576	3.859193	0.105263	0.40908	FALSE	364	364
LV_ EF.Lt EF % (FL)[%]	centile	17	0.5	2.314029	0.424242	0.428275	33	0.559322	3.902313	0	0.461255	FALSE	363	349
LV_ SV.Lt SV % (FL)[%]	centile	17	0.5	2.638254	0.30303	0.42964	33	0.559322	2.880432	0.235294	0.459213	FALSE	362	350
LV_ CO.Lt CO % (AC)[%]	centile	19	0.558824	3.890664	0.25	0.4362	28	0.474576	2.870715	0.105263	0.430102	FALSE	361	359
LV_ SV.Lt SV % (AC)[%]	centile	19	0.558824	3.237662	0.357143	0.438089	28	0.474576	2.734845	0.105263	0.433861	FALSE	360	356
LV_ SV.Lt SV ZS(HC)[]	none	13	0.382353	1.707494	0	0.438915	14	0.237288	3.943137	0	0.299564	FALSE	359	384
LV_ SV/KG.Lt SV/KG ZS(HC)[]	none	13	0.382353	1.706578	0	0.438965	14	0.237288	2.723071	0	0.326082	FALSE	358	379
LV_ EDV.Lt EDV ZS(FL)[]	none	17	0.5	3.378167	0	0.441362	33	0.559322	6.593272	0	0.432339	FALSE	357	358
LV_ EF.Lt EF ZS(HC)[]	none	13	0.382353	1.625872	0	0.443507	14	0.237288	1.89696	0	0.35672	FALSE	356	373
LV_ EF.Lt EF ZS (EFW)[]	none	16	0.470588	2.513639	0	0.449136	19	0.322034	1.838868	0	0.401918	FALSE	355	365
LV_ EDV.Lt EDV % (FL)[%]	centile	17	0.5	2.161914	0.242424	0.452263	33	0.559322	1.302014	0.235294	0.529892	FALSE	354	340
LV_ EDV.Lt EDV % (EFW)[%]	centile	16	0.470588	1.79075	0.210526	0.457572	19	0.322034	1.26452	0.25	0.412655	FALSE	353	362
LV_ CO.Lt CO ZS (EFW)[]	none	16	0.470588	2.085946	0	0.464914	19	0.322034	2.028849	0	0.39308	FALSE	352	366
LV_ CO/KG.Lt CO/KG ZS (EFW)[]	none	16	0.470588	1.827104	0	0.476782	19	0.322034	2.087617	0	0.390567	FALSE	351	367
LV_ CO/KG.Lt CO/KG ZS(FL)[]	none	17	0.5	2.143649	0	0.477241	32	0.542373	2.083589	0	0.500905	FALSE	350	342
LV_ SV.Lt SV ZS(FL)[]	none	17	0.5	2.13539	0	0.477576	33	0.559322	2.707234	0	0.487558	FALSE	349	344
LV_ SV/KG.Lt SV/KG ZS(FL)[]	none	17	0.5	2.128977	0	0.477837	32	0.542373	2.532936	0	0.484407	FALSE	348	345
RV_ Basal-Apical FS[%]	percent_0_100	16	0.470588	1.775925	0	0.47939	45	0.762712	1.242376	0	0.659738	FALSE	347	181
LV_ CO.Lt CO ZS(FL)[]	none	17	0.5	2.08941	0	0.479475	33	0.559322	2.215269	0	0.504067	FALSE	346	341
LV_ SV.Lt SV ZS (EFW)[]	none	16	0.470588	1.700225	0	0.48343	19	0.322034	2.381329	0	0.379314	FALSE	345	368
LV_ SV/KG.Lt SV/KG ZS (EFW)[]	none	16	0.470588	1.596401	0	0.489354	19	0.322034	2.434142	0	0.377494	FALSE	344	369
LV_ EDV.Lt EDV ZS (AC)[]	none	19	0.558824	2.561952	0	0.49171	28	0.474576	4.085417	0	0.415944	FALSE	343	361
LV_ EDV.Lt EDV % (AC)[%]	centile	19	0.558824	1.932639	0.178571	0.497951	28	0.474576	1.522026	0.210526	0.474838	FALSE	342	346
LV_ EF.Lt EF ZS (AC)[]	none	19	0.558824	2.348596	0	0.498865	28	0.474576	2.351735	0	0.456629	FALSE	341	351
RV_ Basal-Apical FS.Rt BASE-AP FS ZS[]	zscore	25	0.735294	2.739372	0.566038	0.518013	53	0.898305	3.240085	0.44	0.59949	FALSE	340	307
LV_ Global Strain.Lt GLS ZS[]	zscore	24	0.705882	2.212906	0.54902	0.522537	51	0.864407	3.974378	0.25	0.587615	FALSE	339	318
RV_ TAPSE septal.TAPSE sept. % (GA)[%]	centile	28	0.823529	6.781968	0.4	0.523166	50	0.847458	3.723863	0.25	0.583405	FALSE	338	324

Supplementary File S6

Inter_Top50: Inter Top50

parameter	qc_rule	intra_n_usable	intra_usable_rate	intra_rel_mdc95	intra_max_flip_rate	intra_score	inter_n_usable	inter_usable_rate	inter_rel_mdc95	inter_max_flip_rate	inter_score	possible_copy_forward_inter	intra_rank	inter_rank
4CV Area (2Dist).4CV A % (AUA)[%]	centile	30	0.882353	0.303273	0	0.848096	54	0.915254	0.002829	0.033333	0.953166	FALSE	17	1
4CV Circ (2Dist).4CV C % (AUA)[%]	centile	30	0.882353	0.293899	0	0.85032	54	0.915254	0.006128	0.033333	0.951858	FALSE	15	2
4CV Length ED.4CV L ED % (AUA)[%]	centile	30	0.882353	0.292921	0.018519	0.848702	54	0.915254	0.011686	0.033333	0.949674	FALSE	16	3
4CV Trv. Width ED.4CV TW ED % (AUA)[%]	centile	30	0.882353	0.378531	0	0.83134	54	0.915254	0.012515	0.033333	0.94935	FALSE	27	4
LV_ ED Length[cm]	positive	30	0.882353	0.407216	0	0.825426	56	0.949153	0.317558	0	0.878168	FALSE	28	5
LV_ ES Length[cm]	positive	30	0.882353	0.333237	0	0.841198	56	0.949153	0.377065	0	0.865049	FALSE	20	6
RV_ ED Length[cm]	positive	30	0.882353	0.429296	0	0.821034	56	0.949153	0.406536	0	0.858963	FALSE	29	7
RV_ ES Length[cm]	positive	30	0.882353	0.521714	0	0.804038	56	0.949153	0.408071	0	0.858653	FALSE	34	8
LV_ ED Area[cm¸]	none	30	0.882353	0.589294	0	0.792861	56	0.949153	0.427659	0	0.854755	FALSE	38	9
RV_ ED Area[cm¸]	none	30	0.882353	0.781799	0	0.765669	56	0.949153	0.45648	0	0.849211	FALSE	46	10
4CV Trv. Width ED.4CV TW ED ZS(AUA)[]	none	30	0.882353	3.102651	0	0.638674	54	0.915254	0.409973	0	0.84132	FALSE	154	11
4CV Length ED.4CV L ED ZS(AUA)[]	none	30	0.882353	1.114588	0	0.730339	54	0.915254	0.448819	0	0.833714	FALSE	58	12
RV_ ES Area[cm¸]	none	30	0.882353	0.747478	0	0.770078	56	0.949153	0.566861	0	0.829864	FALSE	44	13
LV_ EDV[ml]	positive	30	0.882353	1.125847	0	0.729337	56	0.949153	0.615759	0	0.822138	FALSE	62	14
LV_ MAPSE septal.MAPSE sept. ZS(AUA)[]	none	30	0.882353	0.688301	0	0.778101	54	0.915254	0.529221	0	0.819198	FALSE	43	15
LV_ ES Area[cm¸]	none	30	0.882353	0.524116	0	0.803624	56	0.949153	0.721839	0	0.806886	FALSE	35	16
4CV Area (2Dist).4CV A ZS(AUA)[]	none	30	0.882353	1.122562	0	0.729628	54	0.915254	0.753774	0	0.785707	FALSE	61	17
RV_ ED Area.Rt ED Area % (AC)[%]	centile	30	0.882353	1.366391	0.142857	0.695925	56	0.949153	0.820479	0.2	0.774299	FALSE	75	18
RV_ ED Area.Rt ED Area % (EFW)[%]	centile	30	0.882353	1.671337	0.145455	0.676369	55	0.932203	0.801776	0.166667	0.771438	FALSE	95	19
RV_ Global Strain.Rt GLS %[%]	centile	30	0.882353	0.795571	0.339286	0.730018	56	0.949153	0.865128	0.2	0.769039	FALSE	59	20
RV_ FreeWall Strain.Rt FWS %[%]	centile	30	0.882353	0.760566	0.285714	0.739805	56	0.949153	0.853795	0.266667	0.763683	FALSE	54	21
LV_ FreeWall Strain.Lt FWS %[%]	centile	30	0.882353	0.926004	0.303571	0.718503	56	0.949153	0.899334	0.3	0.755176	FALSE	63	22
LV_ ESV[ml]	positive	30	0.882353	0.929099	0	0.748527	56	0.949153	1.231309	0	0.753843	FALSE	52	23
LV_ Global Strain.Lt GLS %[%]	centile	30	0.882353	0.783748	0.357143	0.729709	56	0.949153	1.098928	0.133333	0.751816	FALSE	60	24
LV_ ED Area.Lt ED Area % (AC)[%]	centile	30	0.882353	1.607381	0.125	0.682087	56	0.949153	1.009411	0.233333	0.750306	FALSE	87	25
LV_ Sept.Wall Strain.Lt SWS %[%]	centile	30	0.882353	0.707239	0.357143	0.739759	56	0.949153	1.148314	0.166667	0.744102	FALSE	55	26
RV_ ED Area.Rt ED Area % (AUA)[%]	centile	30	0.882353	2.11645	0.185185	0.651009	54	0.915254	0.916676	0.266667	0.739655	FALSE	132	27
RV_ ED Area.Rt ED Area % (FL)[%]	centile	29	0.852941	2.232714	0.181818	0.632024	55	0.932203	1.07844	0.206897	0.737864	FALSE	164	28
RV_ Sept.Wall Strain.Rt SWS %[%]	centile	30	0.882353	0.883888	0.214286	0.732075	56	0.949153	1.042182	0.366667	0.733778	FALSE	57	29
RV_ ED Area.Rt ED Area % (BPD)[%]	centile	30	0.882353	2.290414	0.218182	0.640924	55	0.932203	1.09708	0.3	0.726843	FALSE	151	30
LV_ ED Area.Lt ED Area % (FL)[%]	centile	29	0.852941	2.153902	0.145455	0.638752	55	0.932203	1.221435	0.206897	0.725476	FALSE	153	31
LV_ ED Length.Lt ED Length ZS(BPD)[]	none	30	0.882353	1.488056	0	0.701945	55	0.932203	1.523228	0	0.724629	FALSE	72	32
RV_ ED Area.Rt ED Area % (HC)[%]	centile	30	0.882353	2.658978	0.2	0.630497	55	0.932203	1.068943	0.366667	0.72277	FALSE	167	33
LV_ ED Length.Lt ED Length ZS(FL)[]	none	29	0.852941	1.621904	0	0.679031	55	0.932203	1.565827	0	0.721997	FALSE	91	34
4CV Circ (2Dist).4CV C ZS(AUA)[]	none	30	0.882353	1.075385	0	0.733912	54	0.915254	1.434154	0	0.721955	FALSE	56	35
LV_ ED Length.Lt ED Length ZS(HC)[]	none	30	0.882353	1.484868	0	0.702151	55	0.932203	1.603283	0	0.719754	FALSE	71	36
LV_ ED Length.Lt ED Length ZS (EFW)[]	none	30	0.882353	1.585392	0	0.695892	55	0.932203	1.612752	0	0.719197	FALSE	76	37
RV_ TAPSE lateral.TAPSE lat. ZS(FL)[]	none	29	0.852941	1.863671	0	0.666151	56	0.949153	1.773326	0	0.718807	FALSE	107	38
LV_ ED Length.Lt ED Length ZS (AC)[]	none	30	0.882353	1.666307	0	0.691197	56	0.949153	1.773601	0	0.718793	FALSE	79	39
LV_ CO.Lt CO ZS(AUA)[]	none	30	0.882353	1.435048	0	0.705444	54	0.915254	1.520502	0	0.716326	FALSE	70	40
RV_ TAPSE lateral.TAPSE lat. ZS (AC)[]	none	30	0.882353	1.907687	0	0.678743	56	0.949153	1.905353	0	0.712253	FALSE	93	41
LV_ ED Area.Lt ED Area % (EFW)[%]	centile	30	0.882353	2.04699	0.109091	0.661544	55	0.932203	1.285842	0.3	0.711092	FALSE	113	42

parameter	qc_rule	intra_n_usable	intra_usable_rate	intra_rel_mdc95	intra_max_flip_rate	intra_score	inter_n_usable	inter_usable_rate	inter_rel_mdc95	inter_max_flip_rate	inter_score	possible_copy_forward_inter	intra_rank	inter_rank
RV_ TAPSE lateral.TAPSE lat. ZS(BPD)[]	none	30	0.882353	1.8154	0	0.683252	55	0.932203	1.792584	0	0.709338	FALSE	83	43
RV_ TAPSE lateral.TAPSE lat. ZS(HC)[]	none	30	0.882353	1.813092	0	0.683369	55	0.932203	1.818592	0	0.708017	FALSE	82	44
LV_ ED Length.Lt ED Length ZS(AUA)[]	none	30	0.882353	1.540191	0	0.698645	54	0.915254	1.665702	0	0.707681	FALSE	73	45
LV_ Frac. Area change.Lt FRAC ZS (EFW)[]	none	30	0.882353	4.417508	0	0.615011	55	0.932203	1.858262	0	0.706047	FALSE	192	46
RV_ TAPSE lateral.TAPSE lat. ZS (EFW)[]	none	30	0.882353	1.883955	0	0.679875	55	0.932203	1.863604	0	0.705786	FALSE	90	47
LV_ Frac. Area change.Lt FRAC ZS(HC)[]	none	30	0.882353	4.461099	0	0.614422	55	0.932203	1.876969	0	0.705137	FALSE	196	48
LV_ Frac. Area change.Lt FRAC ZS (AC)[]	none	30	0.882353	4.441982	0	0.614679	56	0.949153	2.07217	0	0.704777	FALSE	194	49
LV_ Frac. Area change.Lt FRAC ZS(BPD)[]	none	30	0.882353	4.430305	0	0.614837	55	0.932203	1.891523	0	0.704437	FALSE	193	50

Supplementary File S6

Inter_Bottom50: Inter Bottom50

parameter	qc_rule	intra_n_usable	intra_usable_rate	intra_rel_mdc95	intra_max_flip_rate	intra_score	inter_n_usable	inter_usable_rate	inter_rel_mdc95	inter_max_flip_rate	inter_score	possible_copy_forward_inter	intra_rank	inter_rank
LV_ EDV.Lt EDV ZS(HC)[]	none	13	0.382353	9.788871	0	0.328252	14	0.237288	14.951743	0	0.24372	FALSE	386	387
LV_ EF.Lt EF % (HC)[%]	centile	13	0.382353	2.270762	0.285714	0.384901	14	0.237288	3.842809	0.076923	0.293548	FALSE	379	386
LV_ EDV.Lt EDV ZS (EFW)[]	none	16	0.470588	4.549055	0	0.407378	19	0.322034	11.180224	0	0.293857	FALSE	369	385
LV_ SV.Lt SV ZS(HC)[]	none	13	0.382353	1.707494	0	0.438915	14	0.237288	3.943137	0	0.299564	FALSE	359	384
LV_ CO/KG.Lt CO/KG % (HC)[%]	centile	13	0.382353	4.508138	0.357143	0.328082	14	0.237288	2.194811	0.307692	0.313078	FALSE	387	383
LV_ SV.Lt SV % (HC)[%]	centile	13	0.382353	4.322692	0.214286	0.344898	14	0.237288	2.281586	0.230769	0.317459	FALSE	383	382
LV_ SV/KG.Lt SV/KG % (HC)[%]	centile	13	0.382353	3.527187	0.357143	0.343817	14	0.237288	2.101828	0.230769	0.324523	FALSE	384	381
LV_ CO.Lt CO ZS(HC)[]	none	13	0.382353	1.977248	0	0.425529	14	0.237288	2.759188	0	0.32505	FALSE	365	380
LV_ SV/KG.Lt SV/KG ZS(HC)[]	none	13	0.382353	1.706578	0	0.438965	14	0.237288	2.723071	0	0.326082	FALSE	358	379
LV_ CO/KG.Lt CO/KG ZS(HC)[]	none	13	0.382353	2.196114	0	0.416328	14	0.237288	2.505598	0	0.332747	FALSE	368	378
LV_ CO.Lt CO % (HC)[%]	centile	13	0.382353	4.456534	0.357143	0.328769	14	0.237288	1.822234	0.230769	0.337299	FALSE	385	377
LV_ EF.Lt EF % (EFW)[%]	centile	16	0.470588	3.7089	0.368421	0.383398	19	0.322034	3.769071	0.0625	0.338641	FALSE	380	376
LV_ CO/KG.Lt CO/KG % (EFW)[%]	centile	16	0.470588	4.766732	0.368421	0.367815	19	0.322034	2.966415	0.1875	0.343114	FALSE	382	375
LV_ CO.Lt CO % (EFW)[%]	centile	16	0.470588	3.697531	0.263158	0.394129	19	0.322034	2.479717	0.25	0.350969	FALSE	377	374
LV_ EF.Lt EF ZS(HC)[]	none	13	0.382353	1.625872	0	0.443507	14	0.237288	1.89696	0	0.35672	FALSE	356	373
LV_ EDV.Lt EDV % (HC)[%]	centile	13	0.382353	1.89013	0.285714	0.401007	14	0.237288	1.462994	0.230769	0.357971	FALSE	370	372
LV_ SV/KG.Lt SV/KG % (EFW)[%]	centile	16	0.470588	3.659167	0.263158	0.394831	19	0.322034	2.600366	0.125	0.359617	FALSE	375	371
LV_ SV.Lt SV % (EFW)[%]	centile	16	0.470588	2.904775	0.368421	0.400891	19	0.322034	2.259381	0.125	0.37124	FALSE	371	370
LV_ SV/KG.Lt SV/KG ZS (EFW)[]	none	16	0.470588	1.596401	0	0.489354	19	0.322034	2.434142	0	0.377494	FALSE	344	369
LV_ SV.Lt SV ZS (EFW)[]	none	16	0.470588	1.700225	0	0.48343	19	0.322034	2.381329	0	0.379314	FALSE	345	368
LV_ CO/KG.Lt CO/KG ZS (EFW)[]	none	16	0.470588	1.827104	0	0.476782	19	0.322034	2.087617	0	0.390567	FALSE	351	367
LV_ CO.Lt CO ZS (EFW)[]	none	16	0.470588	2.085946	0	0.464914	19	0.322034	2.028849	0	0.39308	FALSE	352	366
LV_ EF.Lt EF ZS (EFW)[]	none	16	0.470588	2.513639	0	0.449136	19	0.322034	1.838868	0	0.401918	FALSE	355	365
LV_ EF.Lt EF % (AC)[%]	centile	19	0.558824	3.879409	0.357143	0.425675	28	0.474576	3.859193	0.105263	0.40908	FALSE	364	364
LV_ CO/KG.Lt CO/KG % (AC)[%]	centile	19	0.558824	5.260396	0.481481	0.395157	27	0.457627	3.140631	0.157895	0.409628	FALSE	374	363
LV_ EDV.Lt EDV % (EFW)[%]	centile	16	0.470588	1.79075	0.210526	0.457572	19	0.322034	1.26452	0.25	0.412655	FALSE	353	362
LV_ EDV.Lt EDV ZS (AC)[]	none	19	0.558824	2.561952	0	0.49171	28	0.474576	4.085417	0	0.415944	FALSE	343	361
LV_ SV/KG.Lt SV/KG % (AC)[%]	centile	19	0.558824	4.07421	0.37037	0.421205	27	0.457627	2.675338	0.105263	0.427121	FALSE	366	360
LV_ CO.Lt CO % (AC)[%]	centile	19	0.558824	3.890664	0.25	0.4362	28	0.474576	2.870715	0.105263	0.430102	FALSE	361	359
LV_ EDV.Lt EDV ZS(FL)[]	none	17	0.5	3.378167	0	0.441362	33	0.559322	6.593272	0	0.432339	FALSE	357	358
LV_ CO/KG.Lt CO/KG % (FL)[%]	centile	17	0.5	4.599418	0.46875	0.374561	32	0.542373	3.38741	0.294118	0.432945	FALSE	381	357
LV_ SV.Lt SV % (AC)[%]	centile	19	0.558824	3.237662	0.357143	0.438089	28	0.474576	2.734845	0.105263	0.433861	FALSE	360	356
LV_ CO.Lt CO % (FL)[%]	centile	17	0.5	3.537292	0.393939	0.398764	33	0.559322	3.24564	0.352941	0.438581	FALSE	373	355
LV_ SV/KG.Lt SV/KG ZS (AC)[]	none	19	0.558824	1.412445	0	0.545219	27	0.457627	2.40731	0	0.446208	FALSE	324	354
LV_ SV.Lt SV ZS (AC)[]	none	19	0.558824	1.483867	0	0.540451	28	0.474576	2.485288	0	0.452056	FALSE	329	353
LV_ SV/KG.Lt SV/KG % (FL)[%]	centile	17	0.5	3.551336	0.375	0.400386	32	0.542373	2.908927	0.176471	0.455869	FALSE	372	352
LV_ EF.Lt EF ZS (AC)[]	none	19	0.558824	2.348596	0	0.498865	28	0.474576	2.351735	0	0.456629	FALSE	341	351
LV_ SV.Lt SV % (FL)[%]	centile	17	0.5	2.638254	0.30303	0.42964	33	0.559322	2.880432	0.235294	0.459213	FALSE	362	350
LV_ EF.Lt EF % (FL)[%]	centile	17	0.5	2.314029	0.424242	0.428275	33	0.559322	3.902313	0	0.461255	FALSE	363	349
LV_ CO/KG.Lt CO/KG ZS (AC)[]	none	19	0.558824	1.648136	0	0.530461	27	0.457627	2.001949	0	0.46206	FALSE	335	348
LV_ CO.Lt CO ZS (AC)[]	none	19	0.558824	1.664635	0	0.529526	28	0.474576	2.121678	0	0.465424	FALSE	337	347
LV_ EDV.Lt EDV % (AC)[%]	centile	19	0.558824	1.932639	0.178571	0.497951	28	0.474576	1.522026	0.210526	0.474838	FALSE	342	346
LV_ SV/KG.Lt SV/KG ZS(FL)[]	none	17	0.5	2.128977	0	0.477837	32	0.542373	2.532936	0	0.484407	FALSE	348	345
LV_ SV.Lt SV ZS(FL)[]	none	17	0.5	2.13539	0	0.477576	33	0.559322	2.707234	0	0.487558	FALSE	349	344
LV_ EF.Lt EF ZS(FL)[]	none	17	0.5	8.006783	0	0.394411	33	0.559322	2.337167	0	0.499523	FALSE	376	343
LV_ CO/KG.Lt CO/KG ZS(FL)[]	none	17	0.5	2.143649	0	0.477241	32	0.542373	2.083589	0	0.500905	FALSE	350	342
LV_ CO.Lt CO ZS(FL)[]	none	17	0.5	2.08941	0	0.479475	33	0.559322	2.215269	0	0.504067	FALSE	346	341
LV_ EDV.Lt EDV % (FL)[%]	centile	17	0.5	2.161914	0.242424	0.452263	33	0.559322	1.302014	0.235294	0.529892	FALSE	354	340
RV_ ED Area.Rt ED Area ZS(GA)[]	none	28	0.823529	3.391173	0	0.602857	51	0.864407	21.60015	0	0.549902	FALSE	224	339
LV_ Basal-Sep.Wall FS.Lt Basal-Sep FS ZS(GA)[]	none	28	0.823529	10.044009	0	0.547983	51	0.864407	15.813823	0	0.555993	FALSE	321	338
